# π-Extended diphosphonium-bridged ladder stilbenes: water-soluble fluorophores with up to eight annulated rings

**DOI:** 10.1039/d5sc03752b

**Published:** 2025-09-29

**Authors:** Sebastian Senn, Jean-Marc Mörsdorf, Maria-Sophie Bertrams, Christoph Kerzig, Joachim Ballmann

**Affiliations:** a Anorganisch-Chemisches Institut Universität Heidelberg Im Neuenheimer Feld 276 D-69120 Heidelberg Germany jean-marc.moersdorf@aci.uni-heidelberg.de; b Department of Chemistry, Johannes Gutenberg University Mainz Duesbergweg 10-14 D-55128 Mainz Germany ckerzig@uni-mainz.de

## Abstract

Diphosphapentalene-derived *P*-heterocyclic materials with two directly fused phospholes are fairly scarce, at least in comparison to their simpler congeners containing only one phosphole entity. To fill that void, π-conjugated naphtho-fused phospholo[3,2-*b*]phosphole dications were prepared *via in situ* oxidation of the corresponding diphosphines. In the case of one specific naphtho-annulation pattern, a hitherto unprecedented bis-(Δ^2^-phosphetene) dication was formed selectively and isolated as a colorless powder. DFT modelling studies revealed that this bis-(Δ^2^-phosphetenium) salt is produced *via* single electron transfer steps, while all the phospholo[3,2-*b*]phosphole salts may either be generated *via* their *P*-diylidic counterparts or *via* similar radical mechanisms. Exploiting this knowledge, the dicationic phospholo[3,2-*b*]phosphole isomer of the bis-(Δ^2^-phosphetenium) salt was isolated as well. In view of the high fluorescence quantum yields of these naphtho-fused phospholo[3,2-*b*]phosphole salts in aqueous solution, linearly π-extended anthraceno-fused derivatives were developed in order to bathochromically shift their emissions into the biological window. While detailed optoelectronic studies confirmed our expectations, the utmost remarkable observation is that even the anthraceno-fused materials were found to be sufficiently soluble in water, despite the fact that these fluorophores comprise up to eight fused rings.

## Abstract 2

Synthetic procedures accompanied by DFT-based mechanistic studies for the preparation of novel fluorophores with efficient red emission and attractive redox potentials are presented, paving the way for applications in optoelectronics and imaging.

## Graphical abstract

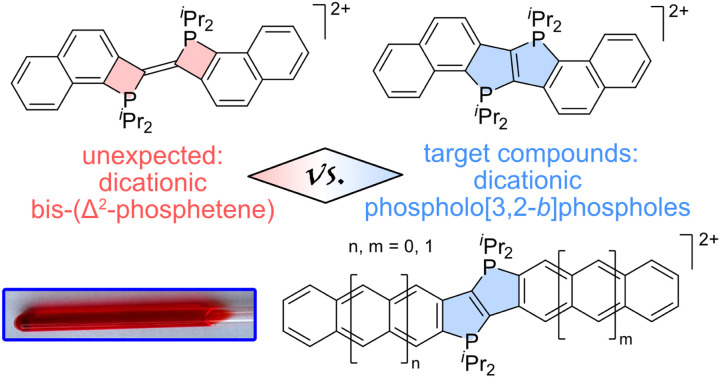

## Introduction

**Scheme 1:**
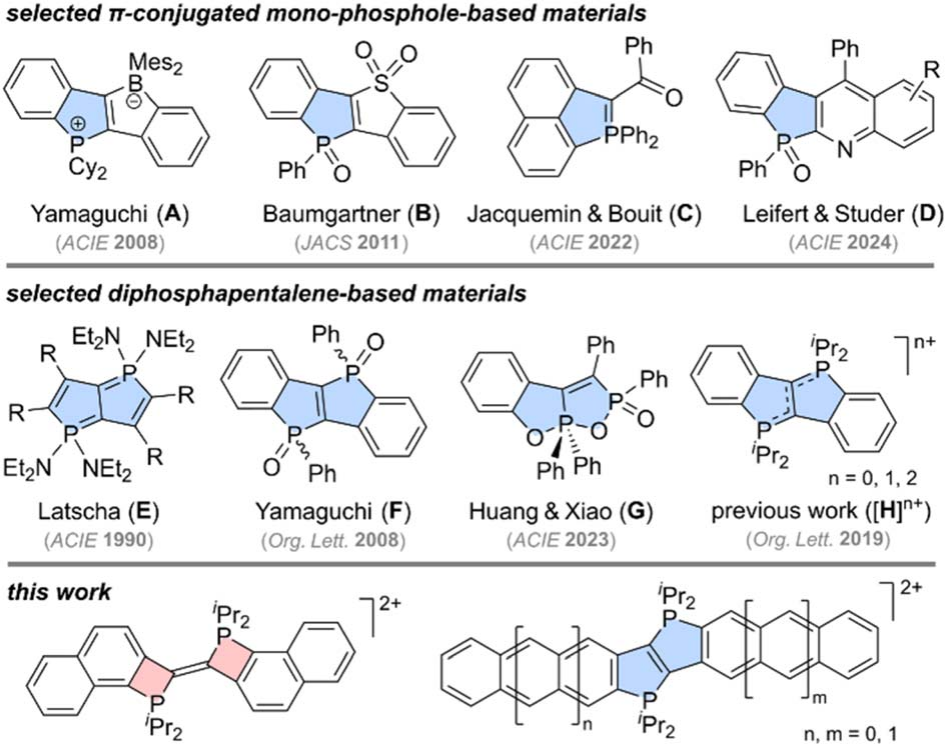
Selection of π-conjugated mono-phosphole-based (top, A–D) and diphosphapentalene-derived materials (middle, E–H) together with the most important structural motifs presented herein (bottom).

Cyclic π-conjugated organophosphorus materials comprising 4-(phosphetenes),^1^ 5-(phospholes),^2^ 6-(phosphinines)^3^ and 7-membered rings (phosphepines)^4^ are known for their manifold applications, not only in organic electronics^5^ and (bio)imaging,^6^ but also in (photo)catalysis.^7^ Despite the seemingly endless possibilities to fuse regular arenes to *P*-containing rings with different ring sizes, π-extended phospholes are targeted most frequently due to the availability of various synthetic protocols for their preparation^8^ and due to the fact that phospholes are readily converted to P(v)-oxides, P(v)-sulphides and P(v)-phospholium cations, which are known to be intrinsically air-stable.^9^ The latter property is certainly of crucial importance for most applications, while the presence of two exocyclic P(v)-substituents is commonly considered beneficial considering that hyperconjugative interactions^10^ between the exocyclic C–P bonds and the endocyclic π-systems are easily exploited in order to fine-tune the electronic properties of the ensuing materials. Hence, π-extended phosphole oxides and phospholium salts have attracted considerable attention over the past years,^11^ which also led to the development of novel synthetic routes for their preparation.^12^ A decade ago, phosphines or phospholes had to be isolated, for example for the preparation of A^13^ and B^14^, while methods that avoid these potentially air-sensitive intermediates only emerged quite recently.^12^ Along these lines, a straightforward one-pot procedure for the preparation of remarkably robust *P*-ylides (C) was reported by Jacquemin and Bouit,^15^ while Leifert and Studer demonstrated that quinoline-fused phosphole oxides, such as D, may be assembled directly from phosphine oxides *via* a new radical cyclization protocol.^16^ These particularly elegant synthetic routes to π-extended mono-phosphole-derived materials, however, are rarely applicable if two mutually fused phospholes need to be incorporated into a given π-framework. Hence, 6π phospholo–phospholes and 8π diphosphapentalenes are best regarded as a compound class of their own, despite the fact that only a few members of this class have been discovered so far. Prior to 2008, solely carboxylate-substituted diphosphapentalenes, such as E (see Scheme 1),^17^ were known, which changed when Yamaguchi's group prepared the first bis-(PhP=O)-bridged ladder stilbenes and succeeded in separating the *cis*- and *trans*-isomer of F.^18^ Separating isomeric mixtures by column chromatography,^19^ however, may be tedious, which is considered a major drawback, in particular if the *P*-oxides are introduced in the last step. Starting from simple phosphinous and phosphinic acids, Huang and Xiao recently managed to prepare dioxaphosphorane-fused diphosphacycles, such as G,^20^ which were shown to reversibly ring-open upon addition of OH^−^. Targeting more robust diphosphacycles, we have set our focus on bis-(R_2_P^+^)-bridged ladder stilbenes (H with *n* = 2) and their use as novel triplet–triplet annihilators in aqueous solution.^21^ In previous studies, the dication of H (*n* = 2) has been prepared *via* oxidation of the corresponding *P*-diylidic diphosphapentalene (H with *n* = 0), which also led to the isolation of the intermediate radical cation (H with *n* = 1).^22*a*^ In contrast to F, no stereochemical peculiarities are to be expected in compounds of type H, which prompted us to exploit this advantage by seeking for π-extended derivatives with bathochromically shifted absorption and fluorescence bands. Herein, the envisioned naphtho- and anthraceno-fused diphosphonium-bridged ladder stilbenes (see Scheme 1) have been prepared without isolating the aforementioned (air-sensitive) *P*-diylidic species. Over the course of our study, an unexpected and hitherto unprecedented bis-(Δ^2^-phosphetene) dication (see Scheme 1) was obtained selectively, which paved the way for a comparative DFT-aided mechanistic analysis. This analysis revealed that the thermodynamically favoured phospholo[3,2-*b*]phosphole dications may be formed *via* three different mechanisms (depending on the reaction conditions), while the formation of the bis-(Δ^2^-phosphetenium) salt relies on a commencing SET (single electron transfer) step to initiate the conversion. In the following, these findings are discussed in more depth and presented in conjunction with detailed photophysical studies.

## Results and discussion

**Scheme 2:**
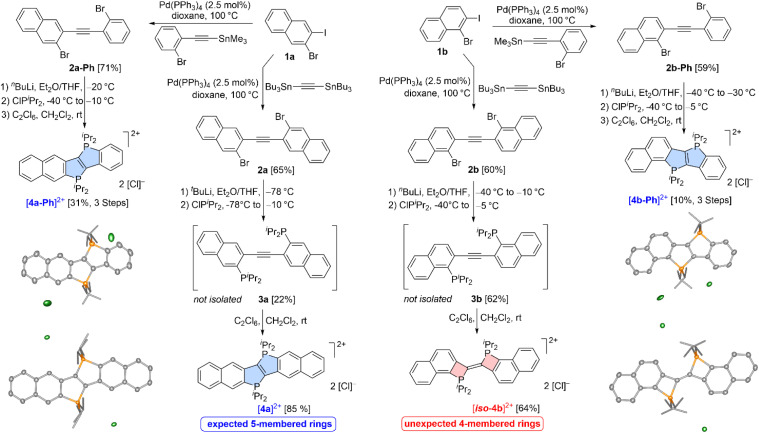
Synthesis of [4a]^2+^, [4a-Ph]^2+^, [iso-4b]^2+^ and [4b-Ph]^2+^ (with [X]^−^ = [Cl]^−^ or [PF_6_]^−^) together with the crystallographically determined molecular structures of the chloride salts (co-crystallized solvents, disorder and hydrogen atoms omitted for clarity, thermal ellipsoids drawn at the 50% probability level).

Given that H has been prepared previously starting from 2,2′-dibromotolane,^22*a*^ similar π-extended dibromo acetylenes were targeted at first. Although many excellent emitters containing the Ph_2_P^+^ unit to connect aromatic systems were reported previously,^22*b*–*e*^ we decided to focus on ^*i*^Pr_2_P^+^-bridged systems as we observed a slightly higher emission quantum yield and, more importantly, a much higher photostability for the ^*i*^Pr_2_P^+^-containing system in a comparative study as well as an improved water solubility (see ref. 22a, Table S9 and related text). Starting from two different regioisomeric bromo-iodonaphthalenes (1a and 1b, see Scheme 2), the di(bromonaphthyl) acetylenes (2a and 2b) were obtained *via* Stille coupling with Bu_3_SnC≡CSnBu_3_. Exchange of the bromides in 2a and 2b for lithium, followed by treatment of the resulting dilithiated intermediates with ClP^*i*^Pr_2_, led to the expected diphosphines, 3a and 3b, which were detected by ^31^P{^1^H} NMR spectroscopy (*δ*(^31^P) = 6.6 ppm for 3a, *δ*(^31^P) = 2.0 ppm for 3b), but not isolated. Upon oxidation of 3a with C_2_Cl_6_ at room temperature, the expected bis-(^*i*^Pr_2_P^+^)-bridged ladder stilbene ([4a]^2+^ with *δ*(^31^P) = 55.9 ppm) was obtained as an air-stable red- orange powder exhibiting a bright orange fluorescence (*vide infra*). Similarly, the bis-[PF_6_]^−^ salt of [4a]^2+^ was obtained upon oxidation of 3a with Fc[PF_6_].

Treatment of 3b with C_2_Cl_6_, however, led to an air-sensitive, nearly colourless powder featuring a significantly downfield-shifted singlet (*δ*(^31^P) = 97.5 ppm) in the ^31^P{^1^H} NMR spectrum. These observations suggested that a significantly different species was produced, which was also obtained upon oxidation of 3b with Fc[PF_6_]. Single crystal X-ray diffraction (scXRD) unambiguously confirmed that an unexpected bis-(Δ^2^-phosphetene) dication ([*iso*-4b]^2+^, see Scheme 2) was generated. In [*iso*-4b]^2+^, two 4-membered phosphetenium cations are interconnected *via* a shared C=C bond (*d*_C=C_ = 1.336(4) Å), thus forging a planar, but not fully ring-fused bis-(^*i*^Pr_2_P^+^)-containing ladder stilbene. To the best of our knowledge, bis-(Δ^2^-phosphetenes) akin to [*iso*-4b]^2+^ have not been reported previously, while uncharged mono-phosphetenes with an exocyclic double bond are well-known.^1*c*,*e*,23^ To address the question whether each naphthalene unit in 3a and 3b is needed to dictate the reaction outcome ([4a]^2+^*vs.* [*iso*-4b]^2+^), one naphthyl unit in 2a and 2b was replaced for a phenyl entity (*cf.*2a-Ph and 2b-Ph). Employing the aforementioned methodology *via* dilithiation, phosphination and consecutive oxidative cyclization (*vide supra*), [4a-Ph]^2+^ (*δ*(^31^P) = 58.2 and 56.2 ppm) and [4b-Ph]^2+^ (*δ*(^31^P) = 58.3 and 66.3 ppm) were obtained as orange powders and found to exhibit ^31^P{^1^H} NMR shifts akin to the one observed for [4a]^2+^ (*δ*(^31^P) = 55.9 ppm). These NMR data strongly suggested that 5-membered rings ([4a-Ph]^2+^ and [4b-Ph]^2+^) were produced, which was confirmed by scXRD (see Scheme 2). On this basis, it is clear that the bis-(Δ^2^-phosphetene) dication [*iso*-4b]^2+^ is truly exceptional and that the specific naphtho-annulation pattern present in 3b is needed at both ends of the central alkyne to selectively forge the 4-membered rings in [*iso*-4b]^2+^.

**Fig. 1:**
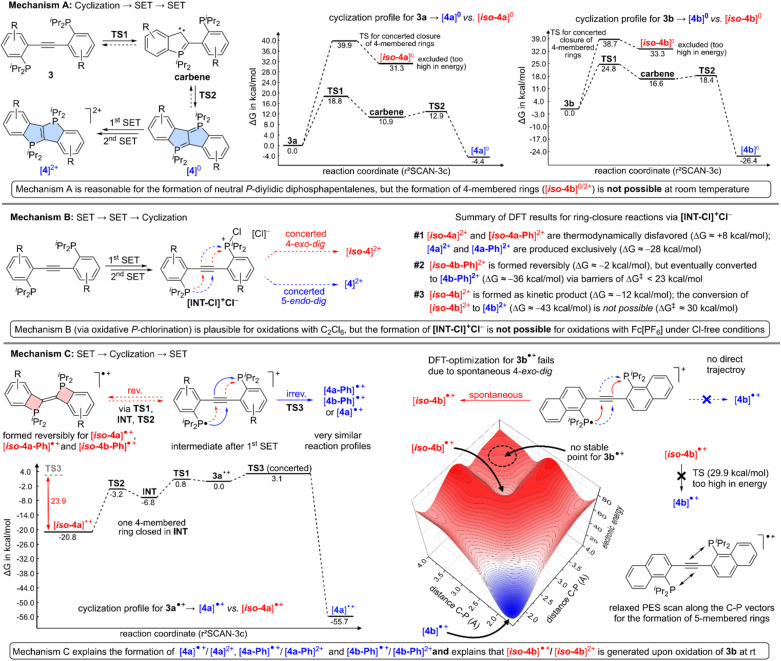
Mechanistic proposals (mechanism A, B and C) for the formation of [4a]^2+^, [4a-Ph]^2+^, [4b-Ph]^2+^ and [*iso*-4b]^2+^ based on DFT calculations (r^2^SCAN-3c, D4, def2-mTZVPP, CPCM for CH_2_Cl_2_). Additional energy profiles and relaxed PES scans are provided in the SI.

To further elucidate these observations, DFT modelling studies (r^2^SCAN-3c, D4, def2-mTZVPP, CPCM for CH_2_Cl_2_)^24^ were carried out. Given that [H]^0^ (see Scheme 1) has been previously isolated in form of its *P*-diylide and oxidized *via* two consecutive SET steps, we at first assumed that a similar cyclization → SET → SET mechanism (*cf.* mechanism A in Fig. 1) may be operative here. In such a scenario, *P*-diylidic diphosphapentalenes are formed *via* carbene intermediates,^25^ which leads to 5-membered rings in all cases ([4a]^0^, [4b]^0^, [4a-Ph]^0^ and [4b-Ph]^0^). The formation of neutral P-diylidic bis-(Δ^2^-phosphetenes), however, is thermodynamically and kinetically prohibited (activation barriers of >35 kcal mol^−1^). Hence, mechanism A was excluded as it fails to explain the formation of [*iso*-4b]^0^ (and thus the formation of its experimentally observed dication [*iso*-4b]^2+^), at least at room temperature. Yet, it is noted that the barriers along the way to each *P*-diylidic diphosphapentalene are very reasonable, which suggested that this mechanistic pathway may be exploited in the absence of an oxidant (*vide infra*). In the search for a mechanism, which actually explains that [*iso*-4b]^2+^ is produced selectively in our experiments, two alternative mechanisms were considered, namely a SET → SET → cyclization (mechanism B) and a SET → cyclization → SET sequence (mechanism C). In mechanism B, a chlorophosphorus(v) chloride intermediate ([INT-Cl]^+^Cl^−^, see Fig. 1) is expected to form after two consecutive SET steps, which has been proposed previously for similar cyclization reactions.^26^ Starting from [INT-Cl]^+^Cl^−^, each 5-*endo-dig* cyclization is thermodynamically favoured and predicted to occur for [4a]^2+^, [4a-Ph]^2+^ and [4b-Ph]^2+^ (see Fig. 1 and SI for details). Upon oxidation of 3b to its chlorophosphorus(v) chloride intermediate, however, the kinetic product [*iso*-4b]^2+^ is expected to form at room temperature, while the thermodynamic product [4b]^2+^ is inaccessible given that a barrier of approximately 30 kcal mol^−1^ was calculated for the conversion of [*iso*-4b]^2+^ to [4b]^2+^. Hence, mechanism B is in line with the experimental findings and considered plausible, but only if a chlorine synthon (such as C_2_Cl_6_) was used to actually produce [INT-Cl]^+^Cl^−^ in the first place.

Considering that the use of Fc[PF_6_] in non-chlorinated solvents also led to [4a]^2+^, [4a-Ph]^2+^, [4b-Ph]^2+^ and [*iso*-4b]^2+^, yet another mechanism was studied *in silico*, namely the aforementioned SET → cyclization → SET sequence (mechanism C). In this scenario, radical cations (3˙^+^) are produced in the first SET step, which were identified as local minima in the case of 3a˙^+^, 3a-Ph˙^+^ and 3b-Ph˙^+^. For these three radical cations, a cyclization to 4-membered and 5-membered rings is plausible as shown for 3a˙^+^ in Fig. 1. In the case of 3a˙^+^, [*iso*-4a]˙^+^ is formed reversibly given that less than 25 kcal mol^−1^ are required to open the 4-membered rings, while the formation of [4a]˙^+^ is energetically favoured by approximately 55 kcal mol^−1^ suggesting that [4a]˙^+^ is produced irreversibly at room temperature. While very similar reaction profiles are also found for 3a-Ph˙^+^ and 3b-Ph˙^+^, an entirely different situation was encountered during the optimization of 3b˙^+^, which was found to spontaneously cyclize to [*iso*-4b]˙^+^. To further support this finding, a relaxed PES (potential energy surface) scan along both C–P vectors that are involved in a 5-*endo-dig* cyclization was carried out. The latter scan confirmed that [*iso*-4b]˙^+^ is formed in a barrier-free manner, while no energetically reasonable trajectory interconnecting the starting point (approximate geometry of 3b˙^+^) and [4b]˙^+^ was found, despite the fact that [4b]˙^+^ is thermodynamically favoured over [*iso*-4b]˙^+^ by approximately 45 kcal mol^−1^. For the thermal conversion of [*iso*-4b]˙^+^ to [4b]˙^+^, an activation barrier of 29.9 kcal mol^−1^ was calculated, which is prohibitively high for a reaction at room temperature.

The finding that [*iso*-4b]˙^+^ is formed *via* a barrier-free pathway upon 1e^−^-oxidation of 3b also inferred that a twofold oxidation of 3b (*cf.* mechanism B) is only possible if the second SET step is faster than intramolecular radical cyclization, which is unlikely for most 1e^−^ oxidants. For C_2_Cl_6_, however, it is known that 
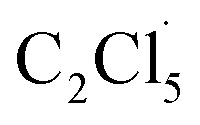
, which is formed after the first SET, is more oxidizing than its parent reagent (C_2_Cl_6_),^27^ thus rendering two consecutive SETs possible. Hence, it is concluded that mechanism B is plausible for highly oxidizing chlorine-synthons (such as C_2_Cl_6_), while mechanism C is considered more likely for prototypical 1e^−^ oxidants (such as Fc[PF_6_]).

**Scheme 3:**
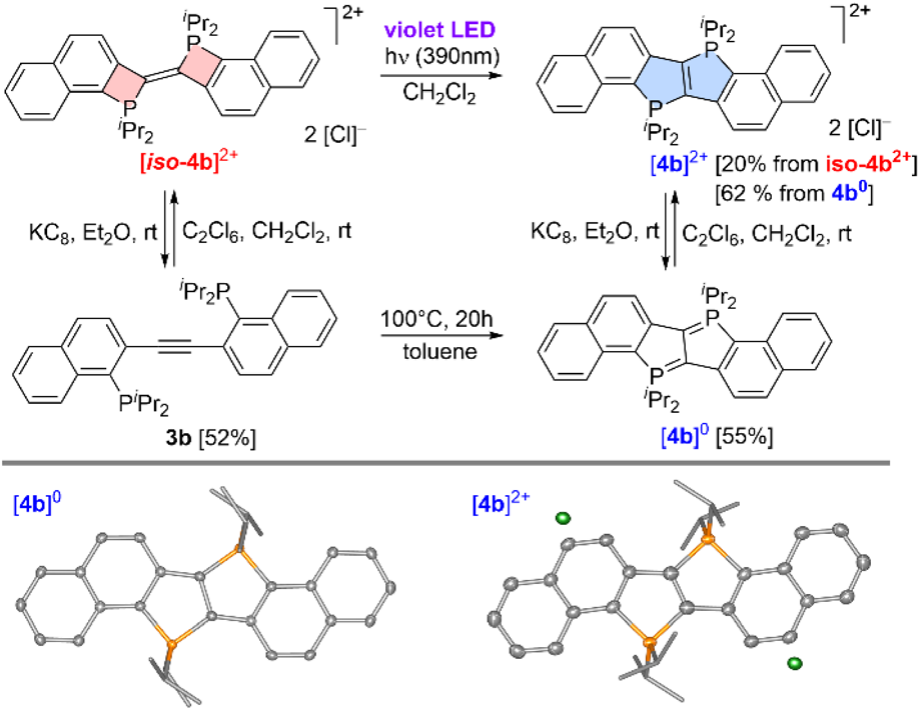
Photochemical and thermal conversion of [*iso*-4b]^2+^ to [4b]^2+^ (top) together with the crystallographically determined molecular structures of [4b]^0^ and [4b]^2+^ (bottom, co-crystallized solvents and hydrogen atoms omitted, thermal ellipsoids drawn at the 50% probability level).

Despite the good agreement between mechanisms B and C with all our experimental findings, we were puzzled by the fact that mechanism A predicts the formation of *P*-diylidic diphosphapentalenes, not only for [4a]^0^, [4a-Ph]^0^ and [4b-Ph]^0^, but also for [4b]^0^. To elucidate whether [4b]^0^ may be prepared *via* mechanism A, [*iso*-4b]^2+^ was reduced to 3b as shown in Scheme 3.

The finding that [*iso*-4b]^0^ was not detected during this reduction, is in line with the prediction that *P*-diylidic bis-(Δ^2^-phosphetenes) are thermodynamically disfavoured relative to the corresponding diphosphines (*cf.* mechanism A). Upon heating of 3b in the absence of C_2_Cl_6_, however, a fairly selective transformation to [4b]^0^ (*δ*(^31^P) = 32.3 ppm) set in at 60 °C, which is in line with mechanism A. In a subsequent step, the thus obtained *P*-diylide [4b]^0^ was oxidized using C_2_Cl_6_, which led to the originally envisioned dicationic phospholo[3,2-*b*]phosphole dication [4b]^2+^ (*δ*(^31^P) = 65.2 ppm). Alternatively, the latter product ([4b]^2+^) may as well be prepared directly from [*iso*-4b]^2+^*via* irradiation with violet light, while vast decomposition set in upon heating of [*iso*-4b]^2+^ to temperatures of ≥70 °C. Taken together, these findings strongly suggested that mechanism A is operative in the absence of an oxidant (as exploited for the thermal conversion of 3b to [4b]^0^), while one (for Fc[PF_6_]) or two (for C_2_Cl_6_) SET steps are needed for the oxidative cyclization of 3b to [*iso*-4b]^2+^ (see Scheme 2) at room temperature. Furthermore, we were able to show that the position of the alkyne on the naphthalene scaffold (connected to C-1 *vs.* C-2) plays a decisive role in the cyclization process. For the C-1 alkyne-linked naphthalene derivative 2d-Naph (see SI, Scheme S6), treatment with PhICl_2_ did not result in cyclization but instead led to chlorination at the phosphine moieties, indicating pronounced steric influences on this reaction.

**Scheme 4:**
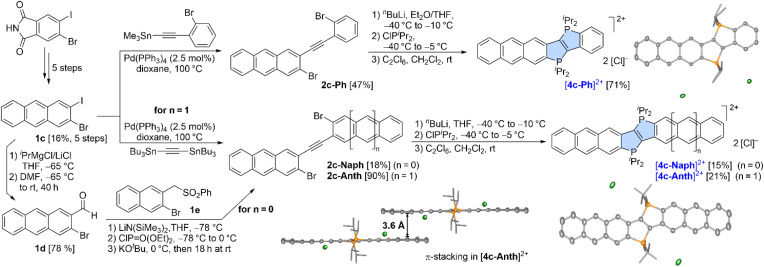
Synthesis of [4c-Ph]^2+^, [4c-Naph]^2+^ and [4c-Anth]^2+^ together with the crystallographically determined molecular structures of [4c-Ph]^2+^ and [4c-Anth]^2+^ (co-crystallized solvent, disorder and hydrogen atoms omitted for clarity, thermal ellipsoids drawn at the 50% probability level).

With the knowledge that 4-*exo-dig* cyclizations are to be expected for annulation patterns akin to the one in 3b, we set out to further expand the π-system in [4a]^2+^ and [4a-Ph]^2+^ by targeting linearly fused anthraceno analogues.^28^ For this purpose, 2-bromo-3-iodo-anthracene (1c, see Scheme 4) was prepared in five steps starting from 5-bromo-6-iodo-phthalimide (see SI for details). With 1c at hand, 2c-Ph and 2c-Anth were obtained in analogy to 2a as shown in Scheme 4. For 2c-Naph, however, a different method had to be established given that multiple attempts to prepare the required stannylated 2-bromo-3-naphthyl acetylene met with failure. Thus, the envisioned dibromo naphthyl-anthracenyl acetylene was eventually assembled *via* a Julia–Lythgoe-type alkyne synthesis starting from 1d and an appropriately substituted sulfone (1e). With the dibromides 2c-Ph, 2c-Naph and 2c-Anth available, the target compounds [4c-Ph]^2+^, [4c-Naph]^2+^ and [4c-Anth]^2+^ were produced without difficulties (see Scheme 4) and isolated as red-orange powders.

In the molecular structure of [4c-Anth]^2+^, colinear π-stacking layers were found (see Scheme 4), which are also present in [4a]^2+^, but absent in all other phospholo[3,2-*b*]phosphole dications reported herein. This finding, however, seems to be related to crystal packing effects given that all compounds [4]^2+^ are nearly planar (with respect to the π-system) and therefore supposedly suited to engage in π-stacking interactions.

**Fig. 2:**
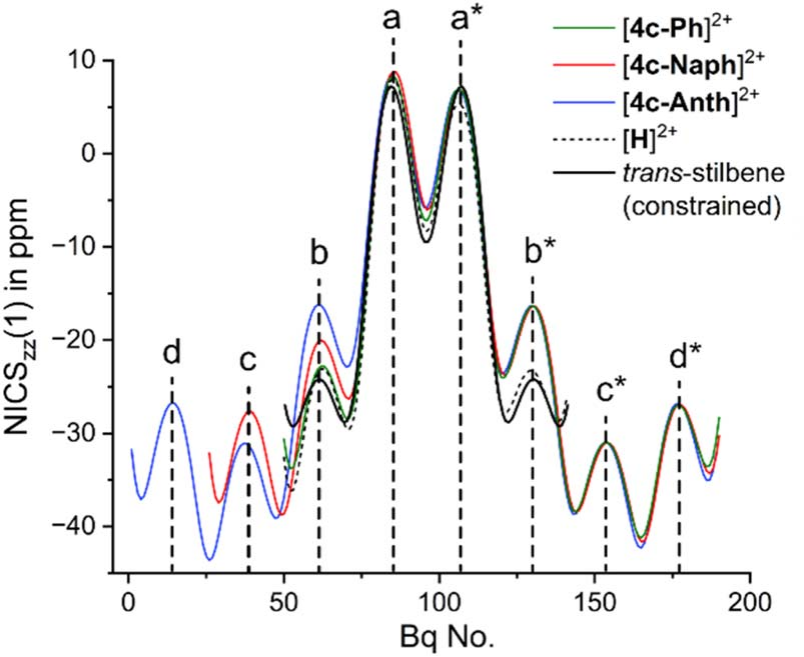
NICS_zz_(1) *XY*-scans for [H]^2+^, [4c-Ph]^2+^, [4c-Naph]^2+^, [4c-Anth]^2+^and for a geometrically constrained *trans*-stilbene. The positive NICS_zz_(1) values for the *a* and a* are not indicative of antiaromaticity given that almost identical values were found for the latter *trans*-stilbene.

To gauge the extent of electron delocalization within the π-system of our new chromophores, AICD calculations (anisotropy of the induced current density)^29^ and NICS_zz_(1) *XY*-scans (nucleus-independent chemical shifts)^30^ were carried out (B3LYP, GD3, def2-TZVPP, SCRF for water). In all cases, diatropic ring currents indicative of aromaticity were found for the arenes on each side of the dicationic phospholo[3,2-*b*]phosphole core, while global ring currents involving the ^*i*^Pr_2_P^+^-bridges were clearly absent (see SI for details). Hence, the individual π-systems at both ends of each molecule are conjugated *via* the central dicationic core, but independent in terms of aromaticity. In [4c-Ph]^2+^, [4c-Naph]^2+^ and [4c-Anth]^2+^, for example, negative NICS_zz_(1) values were calculated for the b*, c* and d* rings, while positive NICS_zz_(1) values of +7(±2) ppm were found for the *a* and a* rings (see Fig. 2). These positive values for the *a* and a* rings, which are commonly seen as a sign for antiaromaticity,^31^ are misleading in the present case: In a geometrically constrained *trans*-stilbene, which was constructed by replacing the ^*i*^Pr_2_P^+^moieties in [H]^2+^ for hydrogen atoms, almost identical NICS_zz_(1) values were calculated, suggesting that all the π-extended derivatives of [H]^2+^ are best interpreted as diphosphonium-bridged ladder stilbenes.

Due to the dicationic charge of all these compounds, energetically low-lying LUMOs are to be expected and indeed found *in silico* (see SI for details). A closer inspection revealed that the linearly annulated derivatives ([4a-Ph]^2+^, [4a]^2+^, [4c-Ph]^2+^, [4c-Naph]^2+^ and [4c-Anth]^2+^) exhibit LUMO energies in the narrow range of −3.48 ± 0.11 eV, while slightly lower energies of −3.70 ± 0.11 eV were calculated for the non-linear compounds ([4b-Ph]^2+^ and [4b]^2+^). For that reason, each series (linear *vs.* non-linear) is expected to display similar reduction potentials, which was confirmed by cyclic voltammetry (see SI for details). As expected, reduction of the non-linear derivatives (to their radical cations) was observed at less negative potentials (*E*_1/2_ = −0.71 ± 0.05 V *vs.* Fc/Fc^+^ for [4b-Ph]^2+^ and [4b]^2+^) in comparison to the corresponding reduction waves for the linear compounds ([4a-Ph]^2+^, [4a]^2+^, [4c-Ph]^2+^, [4c-Naph]^2+^ and [4c-Anth]^2+^ with *E*_1/2_ = −0.92 ± 0.10 V *vs.* Fc/Fc^+^). From exemplary measurements in an extended positive potential range, oxidation waves were observed for [H]Cl_2,_ [4a-Ph]Cl_2_ and [4c-Ph]Cl_2_ (*E*_1/2_ = +0.47 ± 0.05 V, *E*_1/2_ = +0.51 ± 0.05 V and *E*_1/2_ = +0.49 ± 0.05 V *vs.* Fc/Fc^+^, respectively). Comparative measurements with triflate salts, however, unambiguously showed that these signals do not arise from oxidation of the π-conjugated ladder-stilbene framework, but rather from electrochemical oxidation of the chloride counterion (see SI, Fig. S153).

**Fig. 3:**
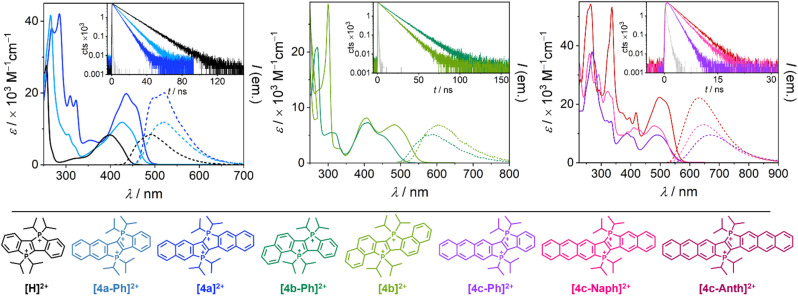
Absorption and emission spectra of the chromophores as their chloride salts dissolved in pure water. The decay traces of the excited states are shown in the insets. Details for the measurements are provided in the SI. Strongly diluted dye solutions (lifetime measurements: *c* < 25 μM; emission spectra: *c* < 105 μM) were used for emission spectroscopy to avoid self-quenching or filter effects.

To further substantiate our findings, photophysical measurements and TD-DFT calculations (B3LYP, def2-TZVPP, GD3, solvation-corrected for H_2_O) were carried out. In all cases, the Cl^−^ salts, which are soluble in water in micromolar concentrations, were found to exhibit high molar absorption coefficients in the visible and ultraviolet spectral range (see Fig. 3). The lowest energy absorption in each compound is interpreted as a predominant π → π*-transition involving the HOMO and the LUMO, which is in line with our TD-DFT analysis (see SI for details). All new chromophores emit from an excited singlet state with fluorescence lifetimes (*τ*_0_) in the low nanosecond range (see Table 1 and SI).

**Table 1:** Photophysical characteristics of the chromophores (see Fig. 3 for spectra, decay traces and structures) in aqueous solution. Details for the measurements are given in the SI

	[H]^2+^^a^	[4a-Ph]^2+^	[4a]^2+^	[4b-Ph]^2+^	[4b]^2+^	[4c-Ph]^2+^	[4c-Naph]^2+^	[4c-Anth]^2+^
*ε*/10^3^ M^−1^ cm^−1^ (nm)	29.6 ± 1.0 (256)	41.6 ± 0.3 (265)	42.1 ± 1.1 (285)	20.9 ± 0.7 (270)	28.6 ± 0.0 (298)	37.9 ± 0.2 (269)	24.4 ± 0.3 (322)	53.2 ± 0.4 (336)
	1.8 ± 0.0 (321)	8.6 ± 0.0 (305)	19.3 ± 0.5 (310)	7.3 ± 0.2 (408)	8.2 ± 0.2 (408)	10.1 ± 0.1 (389)	12.0 ± 0.1 (410)	17.2 ± 0.3 (418)
	8.4 ± 0.0 (400)	11.8 ± 0.1 (426)	19.4 ± 0.6 (435)	4.9 ± 0.1 (450)	6.9 ± 0.1 (484)	9.6 ± 0.0 (489)	12.5 ± 0.2 (479)	22.3 ± 0.4 (498)
*E*_00_/eV (nm)	2.81 (441)	2.63 (470)	2.61 (475)	2.46 (503)	2.30 (540)	2.19 (566)	2.23 (555)	2.23 (557)
Δ*E*_calc_/eV (nm)	2.95 (420)	2.76 (449)	2.70 (459)	2.50 (495)	2.31 (537)	2.30 (539)	2.35 (527)	2.29 (542)
*f*_calc_	0.2167	0.3839	0.6105	0.1194	0.1858	0.3700	0.5390	0.8911
*τ*_0_/ns (*λ*_max_/nm)	14.7 ± 0.09 (494)	9.1 ± 0.05 (520)	6.4 ± 0.05 (519)	14.1 ± 0.08 (583)	10.5 ± 0.00 (605)	1.3 ± 0.00 (670)	2.2 ± 0.04 (646)	2.6 ± 0.04 (631)
*ϕ*_FL_ d	0.87 ± 0.019	0.81 ± 0.020^b^	0.88 ± 0.000^b^	0.34 ± 0.005^c^	0.36 ± 0.026^c^	0.09 ± 0.010^c^	0.19 ± 0.008^c^	0.27 ± 0.014^c^
*k*_FL_ e/10^7^ s^−1^	5.9	8.9	14	2.4	3.4	6.9	8.6	10
*k*_nr_ f/10^7^ s^−1^	0.9	2.1	1.9	4.7	6.1	70	37	28

a Ref. 21.

b Fluorescein in 0.1 M NaOH as a reference compound.^33^

c Ruthenium-based complex ([Ru(bpy)_3_]^2+^) in H_2_O as a reference compound.^33^

d Relative quantum yields, see SI for details.

e *k*_FL_ = *ϕ*_FL_/*τ*_0_.

f *k*_nr_ = (1 − *ϕ*_FL_)/*τ*_0_.

In the series [H]^2+^ → [4a]^2+^ → [4c-Anth]^2+^, the π-system is extended linearly and in a symmetric fashion, which is accompanied by a red-shift of the low-energy absorption bands and the fluorescence emission bands. It has also been demonstrated that the optical properties of ladder stilbenes can be further modified by incorporation of different heteroatoms into the scaffold (see Table S10 in the SI).^22*b*,*c*,32^ For related π-extended phospholium salts, similar bathochromic shifts have been reported recently.^22*b*–*d*^ In our case, this trend is reflected in the energies of the 0-0 transitions *E*_00_ (see Table 1 and SI for details), which were determined from the intersections of the normalized absorption and emission spectra. The calculated energies for the S_0_ → S_1_ excitation (see Table 1) were found to be in good agreement with the *E*_00_ values, thus confirming this trend. In [4a-Ph]^2+^ and [4a]^2+^, almost identical *E*_00_ energies were determined experimentally (Δ*E*_00_ = 0.02 eV), but the molar absorption coefficient at the low-energy absorption band is higher in [4a]^2+^ by a factor of ∼1.5. The calculated oscillator strengths for the lowest energy absorptions in [4a-Ph]^2+^ and [4a]^2+^ are in line with this finding. Furthermore, a decrease in the excited-state lifetimes (increase in *k*_FL_) was observed in the series [H]^2+^ → [4a-Ph]^2+^ → [4a]^2+^, which is consistent with the Strickler–Berg equation in the simplified version (constant of radiative decay is proportional to the energy of the lowest transition squared and the corresponding oscillator strength, *k*_rad_ ∼ *ν*^2^*f*).^34^ Within the series of anthraceno-fused compounds ([4c-Ph]^2+^, [4c-Naph]^2+^ and [4c-Anth]^2+^) similar trends were evident: Compared to [H]^2+^ and [4a]^2+^, the *E*_00_ energies are generally lower in all the anthraceno-fused compounds. The molar absorption coefficients for the lowest energy transition were found to increase within the series [4c-Ph]^2+^ → [4c-Naph]^2+^ → [4c-Anth]^2+^, while the *E*_00_ energy remains essentially identical (Δ*E*_00_ = 0.04 eV). The lifetimes increase when going from [4c-Ph]^2+^ to [4c-Anth]^2+^, which is opposite to the trend observed for the linearly naphtho-fused compounds ([4a-Ph]^2+^ → [4a]^2+^). This observation might be related to the degeneracies of the orbitals involved in the S_1_→S_0_ transition: In [4c-Anth]^2+^, two anthracene units are fused to the central dicationic core, whereas the symmetry for [4c-Naph]^2+^ and [4c-Ph]^2+^ is lower. As a result, the degeneracies of the orbitals involved in the transition may differ, which eventually affects *k*_FL_ and therefore *τ*_0_ as well. Moreover, strongly divergent intersystem crossing rates could contribute to such *τ*_0_ differences of seemingly similar compounds. In general, red emitters show significantly lower emission quantum yields compared to their congeners emitting at higher energies as a result of the energy gap law.^35^ In consequence, higher rate constants for the non-radiative decay (*k*_nr_, see Table 1) are commonly observed for red emitters. A quantum yield *ϕ*_FL_ close to 30% for [4c-Anth]^2+^, whose broad emission band is centred around 630 nm, is therefore highly appreciated, in particular in aqueous solution. For the non-symmetric red emitters, a closer look at the frontier orbitals indicates the admixture of charge transfer contributions in addition to pronounced π → π* transitions. Hence, we carried out additional spectroscopic measurements with [4c-Ph]^2+^, [4c-Naph]^2+^ and [4c-Anth]^2+^ for investigating solvatochromic effects (see Fig. S74–S76 and Tables S5–S7 in the SI). The absorption and emission spectra and hence the derived parameters (*E*_00_ and Stokes shift) only change to a small extent (<10%) when going from water *via* polar organic solvents to DCM, substantiating the absence of significant charge transfer characters for the first excited singlet states. The non-linear naphtho-fused chromophores [4b-Ph]^2+^ and [4b]^2+^ differ from their linear congeners, not only in terms of their redox potentials (*vide supra*), but also in their photophysical characteristics: For [4b-Ph]^2+^ and [4b]^2+^, comparatively low molar absorption coefficients and red-shifted absorption and emission bands are found. While our TD-DFT analysis correctly predicted these findings (*cf.* lower oscillator strengths for [4b-Ph]^2+^ and [4b]^2+^, see Table 1), the origin of this effect is not obvious, although it is well-known that the electronic transitions of linear and non-linear π-systems may differ significantly: For π-extended pentalenes, for example, the HOMO–LUMO transitions are allowed in linear structures, but symmetry-forbidden (and therefore less intense) in non-linear derivatives.^36^ For all the diphosphonium bridged ladder stilbenes presented herein, however, the HOMO–LUMO transitions are symmetry-allowed, regardless of the annulation pattern (see SI for details). In the non-linear derivatives, however, significantly lower transition electric dipole moments were calculated (4.9 debye for [4b-Ph]^2+^ and 8.3 debye for [4b]^2+^) in comparison to their linear counterparts (14.4 debye for [4a-Ph]^2+^ and 23.4 debye for [4a]^2+^, see SI for details). In consequence, lower molar absorption coefficients are to be expected for the non-linear chromophores. Finally, the lifetimes of the non-linear derivatives are longer than those of the linear compounds, which is in line with the Strickler–Berg equation, taking the lower *E*_00_ energies and molar absorption coefficients of the non-linear derivatives into account.^34^

## Conclusions

In summary, several π-extended phospholo[3,2-*b*]phosphole dications with different naphtho and anthraceno ring-fusion patterns have been prepared in order to establish a correlation between their structures and their optoelectronic properties. It was found that the annulation pattern (linear *vs.* non-linear) is of crucial importance, not only from a photophysical perspective, but also from a synthetic point of view: For the specific naphtho-annulation pattern present in 3b, a dicationic bis-(Δ^2^-phosphetene) ([*iso*-4b]^2+^) and its phospholo[3,2-*b*]phosphole isomer ([4b]^2+^) have been prepared, each in a selective manner. On this basis, three distinct mechanistic pathways for the cyclization of diphosphinotolanes have been explored *in silico*, which revealed that the formation of uncharged diphosphapentalenes (such as [4b]^0^) *via* mechanism A plays a role in the absence of oxidants, while SET steps play a crucial role in the presence of suitable oxidants (mechanism B for C_2_Cl_6_ or mechanism C for Fc[PF_6_]). While these findings are expected to guide the way to new phospholo[3,2-*b*]phosphole dications, it was also shown that all our new chromophores are best interpreted as diphosphonium-bridged ladder stilbenes (no antiaromaticity within the phospholium rings). Detailed photophysical studies in water revealed that both linearly fused centrosymmetric compounds ([4a]^2+^ and [4c-Anth]^2+^) display fairly high fluorescence quantum yields, while π-stacking interactions were found in their crystalline samples. Hence, [4a]^2+^ and [4c-Anth]^2+^ seem to be particularly suited for sensing applications (*e.g.* intercalation into DNA), which will be a subject of future studies.

## Author contributions

S. S. and J.-M. M. conducted the synthetic work and M.-S. B. conducted the photophysical measurements. J. B. carried out the DFT work and the crystallographic analysis. C. K. and J. B. supervised the project. All authors contributed to writing and manuscript preparation.

## Conflicts of interest

There are no conflicts to declare.

## Supplementary Material

SC-OLF-D5SC03752B-s001

SC-OLF-D5SC03752B-s002

SC-OLF-D5SC03752B-s003

## Data Availability

CCDC 2431371–2431379 and 2470828 contain the supplementary crystallographic data for this paper.^37*a*–*j*^ The data supporting this article have been included as part of the supplementary information (SI). Supplementary information: experimental details, computational data, details on photophysical measurements, electrochemical measurements, crystallographic data. See DOI: https://doi.org/10.1039/d5sc03752b.
