# Supplementary material for: π-Extended diphosphonium-bridged ladder stilbenes: water-soluble fluorophores with up to eight annulated rings
Source: Chem Sci. 2025 Sep 29;16(43):20517–26. doi: 10.1039/d5sc03752b (PMC12499890; doi:10.1039/d5sc03752b)
Supplement: SC-016-D5SC03752B-s002 [file SC-016-D5SC03752B-s002.pdf]

Supplementary Information (SI) for Chemical Science.
This journal is © The Royal Society of Chemistry 2025

Electronic Supplementary Information

π-Extended Diphosphonium-Bridged Ladder Stilbenes: Water-Soluble Fluorophores with up to Eight Annulated Rings

Sebastian Senn,‡[a] Jean-Marc Mörsdorf,‡* [a] Maria-Sophie Bertrams,[b] Christoph Kerzig,*[b] and Joachim Ballmann[◆]

◆ J.B. deceased on June 7, 2025

Abstract: Diphosphapentalene-derived *P*-heterocyclic materials with two directly fused phospholes are fairly scarce, at least in comparison to their simpler congeners containing only one phosphole entity. To fill that void, *π*-conjugated naphtho-fused phospholo[3,2-*b*]phosphole dications were prepared via *in-situ* oxidation of the corresponding diphosphines. In the case of one specific naphtho-annulation pattern, a hitherto unprecedented bis-(Δ^2-phosphetene) dication was formed selectively and isolated as a colorless powder. DFT-modelling studies revealed that this bis-(Δ^2-phosphetenium) salt is produced via single electron transfer steps, while all the phospholo[3,2-*b*]phosphole salts may either be generated via their *P*-diylidic counterparts or via similar radical mechanisms. Exploiting this knowledge, the dicationic phospholo[3,2-*b*]phosphole isomer of the bis-(Δ^2-phosphetenium) salt was isolated as well. In view of the high fluorescence quantum yields of these naphtho-fused phospholo[3,2-*b*]phosphole salts in aqueous solution, linearly *π*-extended anthraceno-fused derivatives were developed in order to batho-chromically shift their emissions into the biological window. While detailed optoelectronic studies confirmed our expectations, the utmost remarkable observation is that even the anthraceno-fused materials were found to be sufficiently soluble in water, despite the fact that these fluorophores comprise up to eight fused rings.

Table of Contents

1) Experimental Procedures 2
2) Optical spectroscopy 55
3) Steady-state absorption and emission measurements 56
4) Time-resolved measurements 69
5) Details on DFT Calculations 71
6) Electrochemical Measurements 132
7) X-Ray Crystal Structure Determinations 135
8) Additional Compounds and Spectra 145
9) References 148

1) Experimental Procedures

1.1) General Remarks

All experiments were conducted under an atmosphere of dry and oxygen-free argon by using standard Schlenk techniques or in a glovebox (MBraun). Argon 5.0 was used and further dried by passing over a column of phosphorus pentoxide. Glassware was heated to 130°C overnight and evacuated while still hot. Dichloromethane, diethylether, hexane, pentane, THF and toluene were purified by a MBraun Solvent Purification System. Deuterated solvents were dried over sodium (benzene-d_6, THF-d_8, toluene-d_8) or over calcium hydride (CD_2Cl_2) and distilled prior to use. Chlorodiisopropylphosphine was purified by simple distillation and stored in Teflon-valve ampules. Bis(tributylstannyl)acetylen was prepared according to literature[1]. All other chemicals were purchased from commercial suppliers and used as received. One and two dimensional ^{1}H, ^{13}C and ^{31}P NMR spectra were recorded on a Bruker Avance DRX 300, a Bruker Avance II 400 MHz or on a Bruker Avance 600 III spectrometer. Unless noted otherwise, all X-nuclei spectra were recorded with ^{1}H broadband or composite pulse decoupling. Residual (undeuterated) solvent served as reference for ^{1}H and ^{13}C NMR spectra. Chemical shifts *d* are given in parts per million (ppm), coupling constants *J* in Hertz (Hz). Signal multiplicities are stated by common abbreviations (e.g. s – singlet, d – doublet, dd – doublet of doublets). Mass spectra were recorded at the Department of Organic Chemistry at Heidelberg University on a Bruker Autoflex Speed MALDI-TOF for MALDI HR MS, on a The Bruker timsTOFfleX for ESI HR MS, on a JEOL AccuTOF GCx for EI HR MS and on a JEOL JMS-700 magnetic sector by liquid injection FD ionization (LIFDI) technique. Elemental analyses were carried out at the Department of Inorganic Chemistry at Heidelberg University on an Elementar vario MICRO Cube.

1.2) Synthesis of ((2-bromophenyl)ethynyl)trimethylstannane

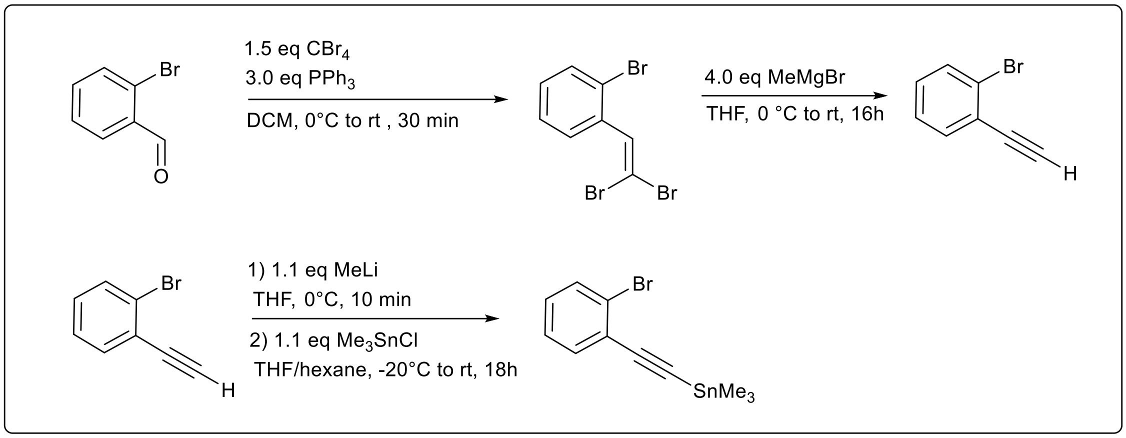

Scheme S1. Synthetic route for the synthesis of ((2-bromophenyl)ethynyl)trimethylstannane.

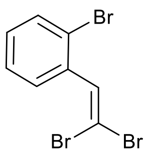

1-Bromo-2-(2,2-dibromovinyl)benzene was synthesized according to a modified procedure of *Florent* and coworkers[2]. 2-Bromobenzaldehyde (37.2 g, 201 mmol, 1.0 eq) and CBr_4 (100 g, 302 mmol, 1.5 eq) were dissolved in dry DCM (300 mL). A solution of PPh_3 (158 g, 603 mmol, 3.0 eq) in dry DCM (300 mL) was added dropwise at 0 °C and the mixture was stirred for 10 min at this temperature. The reaction was allowed to warm up to rt over 30 min. The suspension was diluted with ice-cold n-hexane and was filtered over silica. The filtration cake washed two times with ice-cold n-hexane. Column chromatography (PE) afforded 1-bromo-2-(2,2-dibromovinyl)benzene as a yellow oil (38.8 g, 114 mmol, 57%). **^{1}H NMR** (400 MHz, $CDCl_3$): δ(in ppm) = 7.61–7.58 (m, 2H), 7.51 (s, 1H), 7.34 (td, $J_{H\text{–}H}$ = 7.6, 1.3 Hz, 1H), 7.21 (td, $J_{H\text{–}H}$ = 7.7, 1.7 Hz, 1H). *These data are in accordance with the literature*[2].

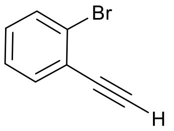

1-Bromo-2-ethynylbenzene was synthesized according to a modified procedure of *Hashmi* and coworkers [3]. 1-bromo-2-(2,2-dibromovinyl)benzene (37.3 g, 109 mmol, 1.0 eq) was dissolved in dry THF (400 mL) in an 1 L Schlenk-flask equipped with a dropping funnel and was cooled to 0 °C. A 3 M ethereal solution of MeMgBr (146 mL, 438 mmol, 4.0 eq) was added dropwise over 1 h. The reaction mixture was stirred at 0 °C for 15 min and at rt for 16 h. The mixture was poured onto ice and was extracted with dichloromethane (15 × 100 mL). The combined organic layer was dried (Na_2SO_4), filtrated over silica gel, and the solvent was removed under reduced pressure. 1-bromo-2-ethynylbenzene was obtained as a brown oil (17.8 g, 98.3 mmol, 90%). **^{1}H NMR** (400 MHz, $CDCl_3$): δ(in ppm) = 7.59 (dd, $J_{H\text{–}H}$ = 8.0, 1.3 Hz, 1H), 7.53 (dd, $J_{H\text{–}H}$ = 7.6, 1.8 Hz, 1H), 7.31–7.25 (m, 1H), 7.23–7.18 (m, 1H), 3.38 (s, 1H). *These data are in accordance with the literature*[3].

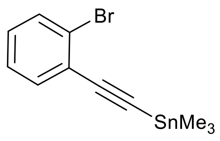

1-Bromo-2-ethynylbenzene (8.10 g, 44.7 mmol, 1.0 eq) was dissolved in deoxygenated and dry THF (250 mL). The mixture was cooled to 0 °C and an ethereal 1.6 M methyllithium solution (1.08 g, 49.2 mmol 1.1 eq) was added via cannula over 10 min. The mixture was stirred and allowed to warm up to rt over 90 min. The reaction mixture was cooled to –20 °C and a solution of Me_3SnCl (10.1 g, 50.6 mmol, 1.1 eq) in dry hexane (50 mL) was added via cannula at once. The mixture was stirred at rt for 18 h and the solvent was removed under reduced pressure. PE was added and the suspension was filtrated. The solvent was removed under reduced pressure and purification by two-fold vacuum distillation (4.5×10^{-2} mbar, 180 °C) afforded compound **1b** as a yellow oil (2.83 g, 8.23 mmol, 19%). **^{1}H NMR** (400 MHz, $CDCl_3$): δ (in ppm) = 7.56 (dd, J_{H-H} = 7.7, 0.9 Hz, 1H), 7.49 (dd, J_{H-H} = 7.7, 1.7 Hz, 1H), 7.23 (td, J_{H-H} = 7.6, 1.2 Hz, 1H), 7.13 (td, J_{H-H} = 7.7, 1.8 Hz, 1H), 0.38 (s, 9H). **^{119}Sn{^{1}H} NMR** (149 MHz, $CDCl_3$): δ (in ppm) = –61.1. **^{13}C{^{1}H} NMR** (101 MHz, $CDCl_3$): δ (in ppm) = 133.8 (CH, 1C), 132.4 (CH, 1C), 129.3 (CH, 1C), 127.0 (CH, 1C), 125.8 (C_q, 1C), 125.7 (C_q, 1C), 106.9 (C_q, 1C), 99.6 (C_q, 1C), –7.5 (CH_3, 3C). **EI HR-MS** (pos): m/z calcd for $C_{11}H_{13}BrSn$ $[M]^+$ 343.9217, found 343.9302.

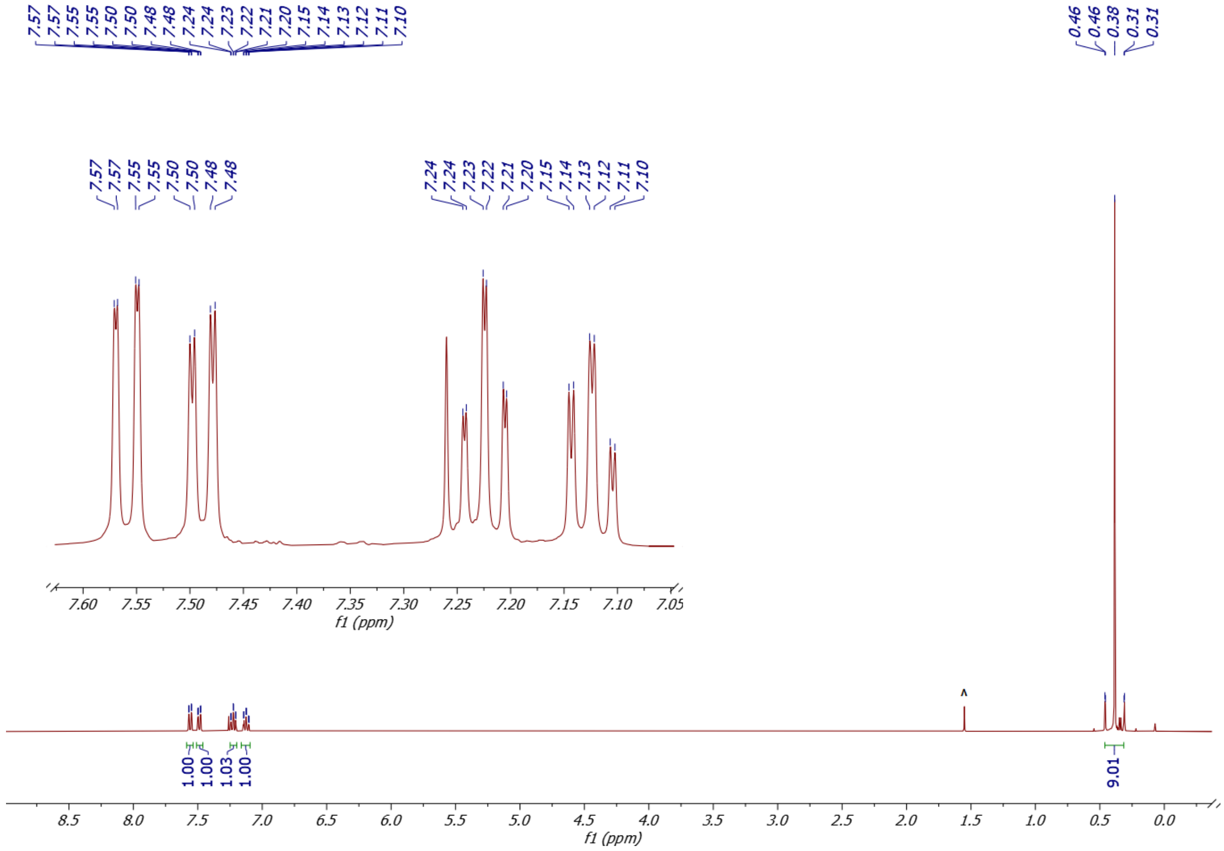

Figure S1. **^{1}H NMR** (400 MHz, $CDCl_3$, 298K) spectrum of ((2-bromophenyl)ethynyl)trimethylstannane (traces of impurities of water(^) are marked).

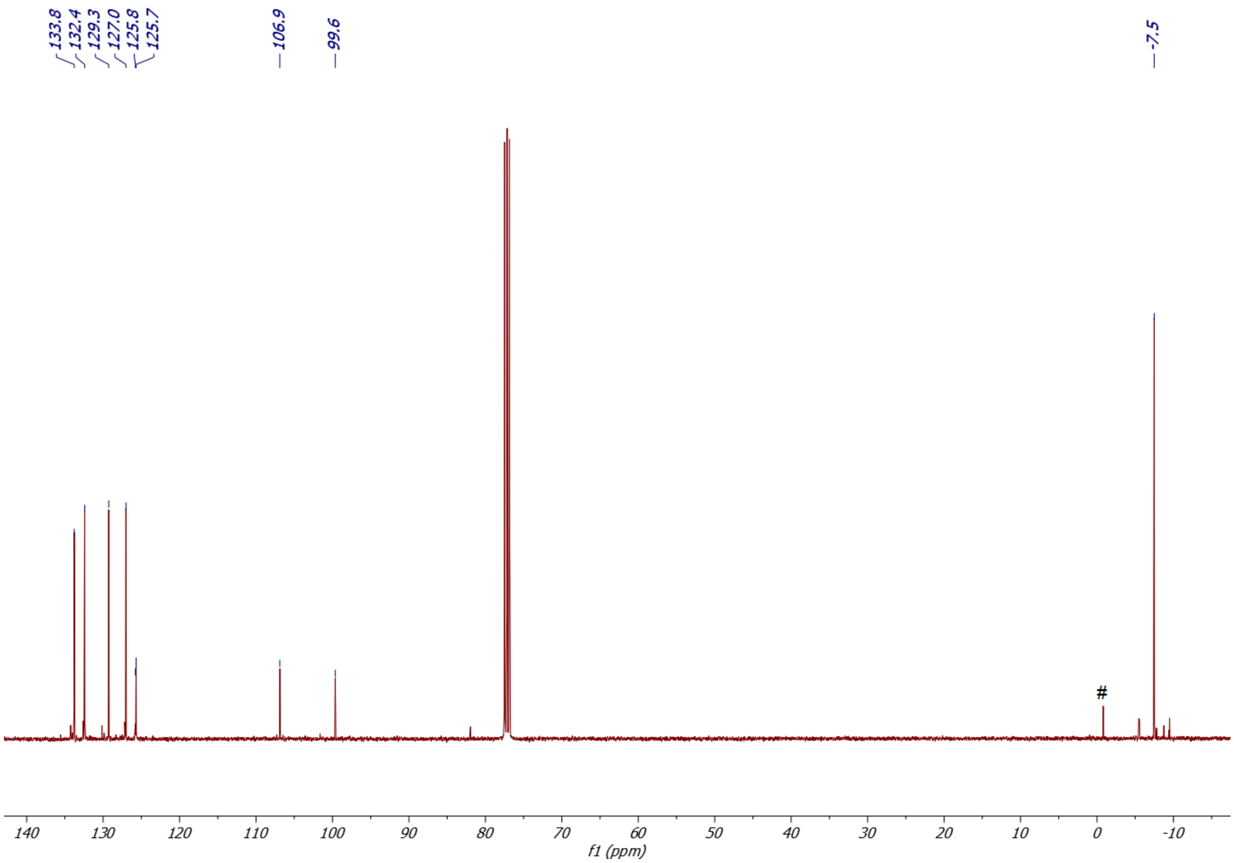

Figure S2. **^{13}C {^{1}H} NMR** (101 MHz, $CDCl_3$, 298K) spectrum of ((2-bromophenyl)ethynyl)trimethylstannane (traces of silicon grease (#) are marked).

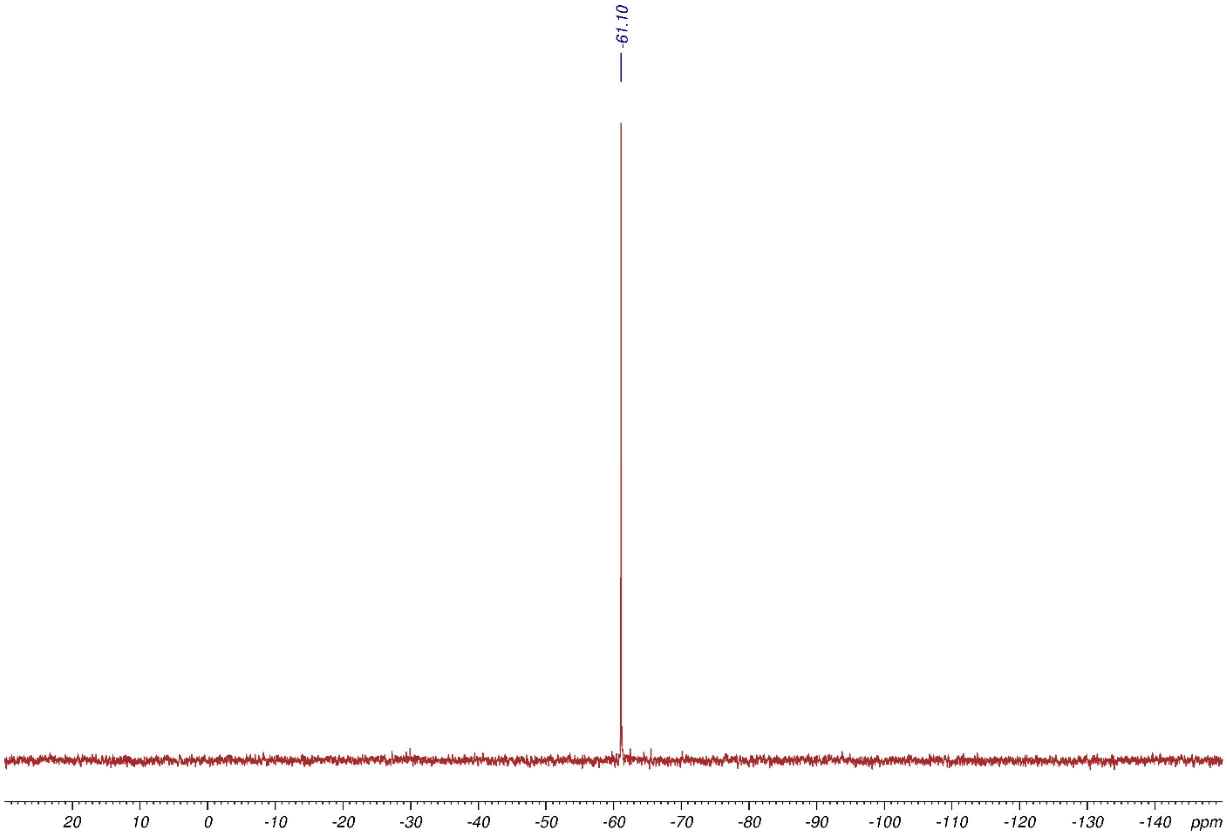

Figure S3. **^{119}Sn{^{1}H} NMR** (149 MHz, $CDCl_3$, 298K) spectrum of ((2-bromophenyl)ethynyl)trimethylstannane.

1.3) Synthesis of $[4a\text{-}Ph]^{2+}$ and $[4a]^{2+}$ from 1a

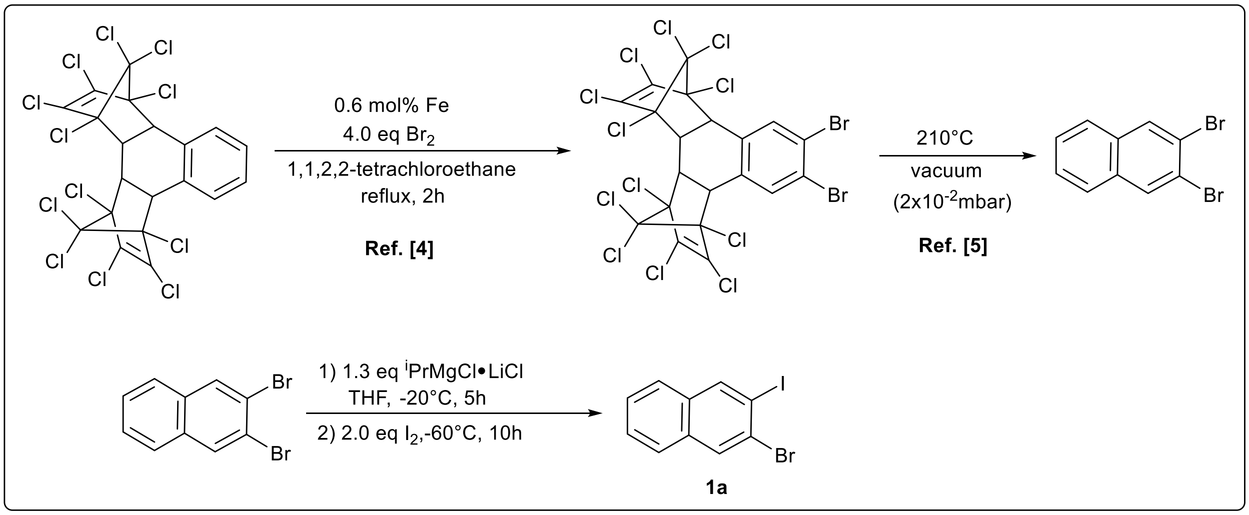

Scheme S2. Synthetic route for the synthesis of compound **1a**.

The precursor 2,3-dibromonaphthalene was synthesized and purified according to literature[4,5] (see Scheme S2) and obtained with a combined yield of 65% (over 2 steps). Compound **1a** and all compounds based on **1a** were synthesized as follows:

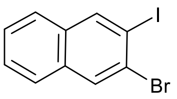

1a

For the synthesis of **1a** the method described by Schlosser and coworkers[6] was modified in the following way: A 1.3 M THF solution of ^{i}PrMgCl·LiCl (35.4 ml, 44.9 mmol, 1.3 eq) was added dropwise at -20°C to 2,3-dibromonaphthalene (9.87 g, 34.51 mmol, 1.0 eq) dissolved in dry THF (50 ml). The mixture was stirred for 5h at this temperature, then cooled to -60°C. I_2 (17.5g, 69.1 mmol, 2.0 eq) dissolved in dry THF (30 ml) was added dropwise. The mixture was warmed to room temperature over 10h, then the solvent was removed in vacuum. 200 ml of Et_2O were added to the residue, followed by 40ml of 4.0 M HCl (aq.). The mixture was stirred for 30 min, the aqueous phase was discarded and the organic phase was dried over $MgSO_4$. After the solvent was removed, crude **1a** was purified by column chromatography (SiO_2, PE/DCM, 25:1) and isolated as colorless crystalline solid (75%). **^{1}H NMR** (600 MHz, $CDCl_3$): δ(in ppm) = 8.41 (s, 1H), 8.14 (s, 1H), 7.73-7.69 (m, 2H), 7.52-7.49 (m, 2H). *These data are in accordance with the literature*[5].

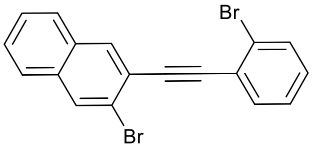

2a-Ph

1a (3.76 g, 11.3 mmol, 1.0 eq), $Pd(PPh_3)_4$ (326 mg, 0.28 mol, 2.5 mol%) and ((2-bromophenyl)ethynyl)trimethylstannane (4.08 g, 11.9 mmol, 1.0 eq) were stirred in dry 1,4-dioxane (60 mL) under an argon atmosphere at 100 °C for 18 h. After the mixture cooled to room temperature, the solvent was removed under reduced pressure. Purification of the crude product, performed by column chromatography (SiO_2, PE/EA, 15:1 + 1% Et_3N) yielded **2a-Ph** as a beige solid (3.11 g, 8.04 mmol, 71%). **^{1}H NMR** (600 MHz, $CDCl_3$): δ(in ppm) = 8.13 (d, $J_{H\text{-}H}$ = 2H), 7.81-7.80 (m, 1H), 7.75-7.73 (m, 1H), 7.67-7.64 (m, 1H), 7.54-7.50 (m, 2H), 7.34-7.32 (m, 1H), 7.23-7.20 (m, 1H). **^{13}C NMR {^{1}H}** (151 MHz, $CDCl_3$): δ(in ppm) = 133.93 (s, C_q, 1C,), 133.83 (s, CH, 1C), 133.71 (s, CH, 1C), 132.69 (s, CH, 1C), 131.83 (s, C_q, 1C), 131.23 (s, CH, 1C), 129.87 (s, CH, 1C),127.89 (s, CH, 1C), 127.84 (s, CH, 1C), 127.21 (s, CH, 1C), 127.06 (s, CH, 1C), 127.01 (s, CH, 1C), 125.68 (s, C_q, 1C), 125.34 (s, C_q, 1C), 122.50 (s, C_q,1C), 121.83 (s, C_q, 1C), 92.75 (s, C_q, 1C), 92.06 (s, C_q, 1C). **EI HR MS** (pos): m/z calcd for $[C_{18}H_{10}Br_2]^+$: 383.9144, found: 383.9145 $[M]^+$.

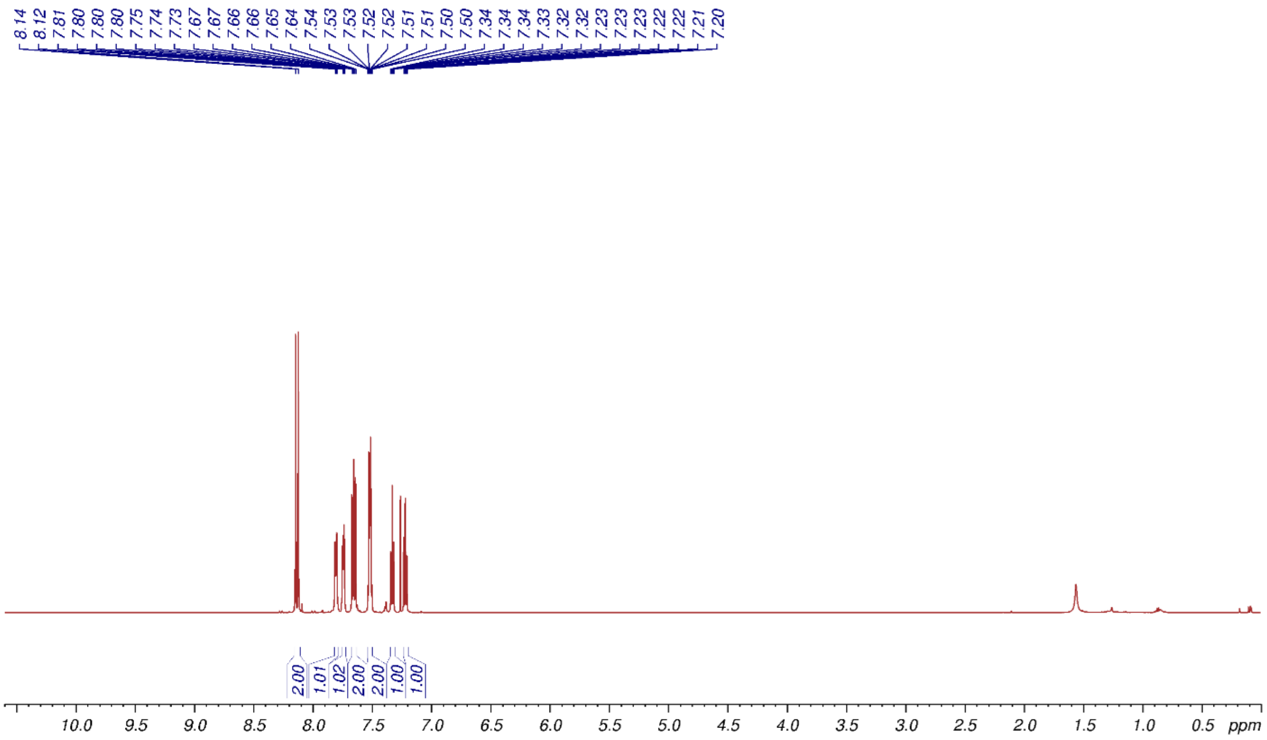

Figure S4. **^{1}H NMR** (600 MHz, $CDCl_3$, 298K) of **2a-Ph**.

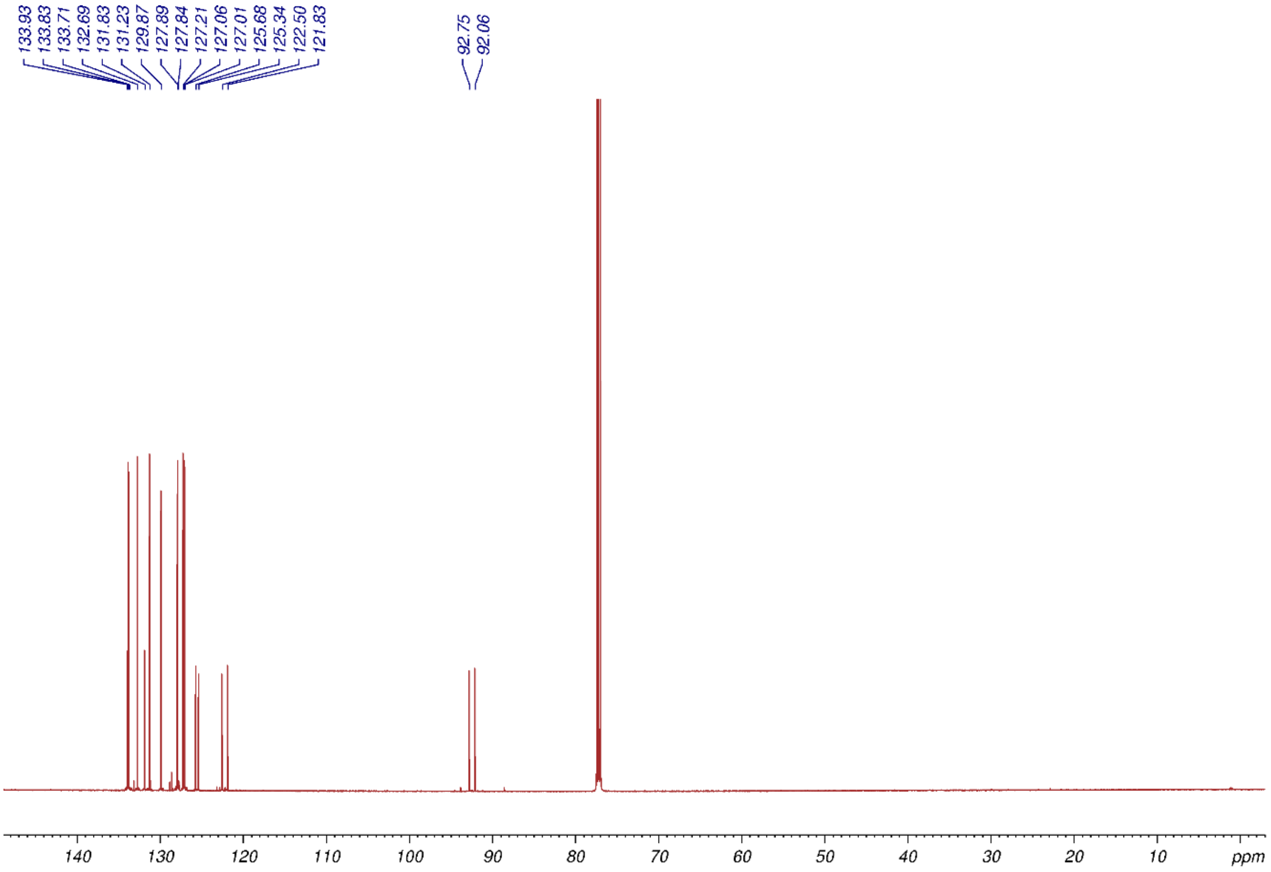

Figure S5. **$^{13}C\{^1H\}$ NMR** (151 MHz, $CDCl_3$, 298K) of **2a-Ph**.

1a (3.23 g, 7.40 mmol), $Pd(PPh_3)_4$ (214 mg, 0.19 mol), and 1,2-bis(tributylstannyl)ethyne (2.23 g, 3.69 mmol, 1.94 mL) were stirred in dry 1,4-dioxane (60 mL) under an argon atmosphere at 100 °C for 15 h. After the mixture cooled to room temperature, the solvent was removed under reduced pressure. Purification of the crude product, performed by column chromatography (SiO_2, PE/DCM, 20:1 + 1% Et_3N) yielded compound **2a** as an beige solid (2.10 g, 4.81 mmol, 65%). **^{1}H NMR** (600 MHz, $CDCl_3$): δ (in ppm) = 8.19 (s, 2H), 8.15 (s, 2H), 7.83-7.82 (m, 2H), 7.77-7.75 (m, 2H), 7.55-7.51 (m, 4H). **^{13}C{^{1}H} NMR** (151 MHz, $CDCl_3$): δ (in ppm) = 134.0 (s, C_q, 2C), 133.8 (s, CH, 2C), 131.9 (s, C_q, 2C), 131.3 (s, CH, 2C), 127.9 (s, CH, 2C), 127.9 (s, CH, 2C), 127.1 (s, CH, 2C), 127.0 (s, CH, 2C), 122.6 (s, C_q, 2C), 121.9 (s, C_q, 2C), 92.4 (s, C_q, 2C). **MALDI HR-MS** (pos): *m/z* calcd for $[C_{22}H_{12}Br_2]^+$: 433.9300, found: 433.9305 $[M]^+$.

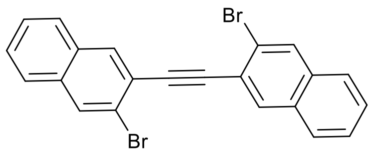

2a

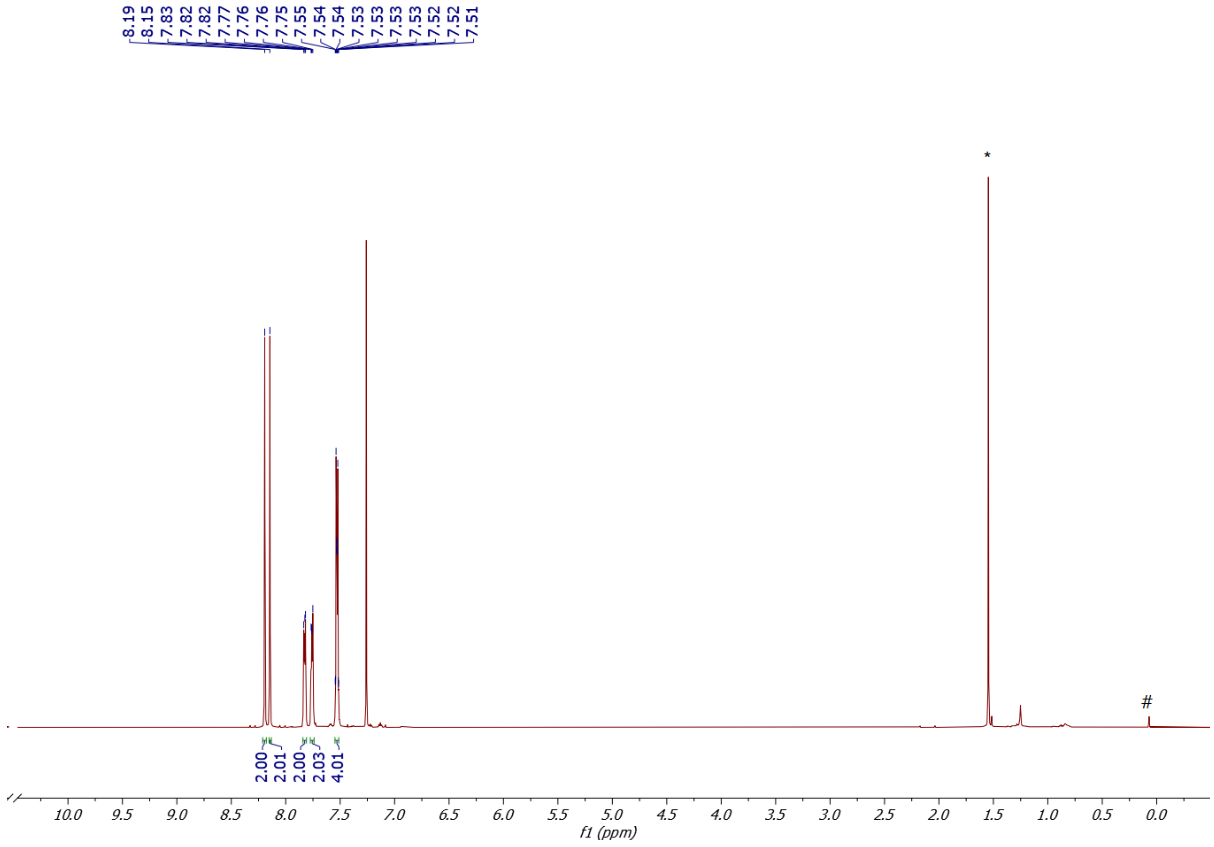

Figure S6. **^{1}H NMR** (600 MHz, $CDCl_3$, 298K) of **2a** (traces of impurities of water (*) and silicon grease (#) are marked).

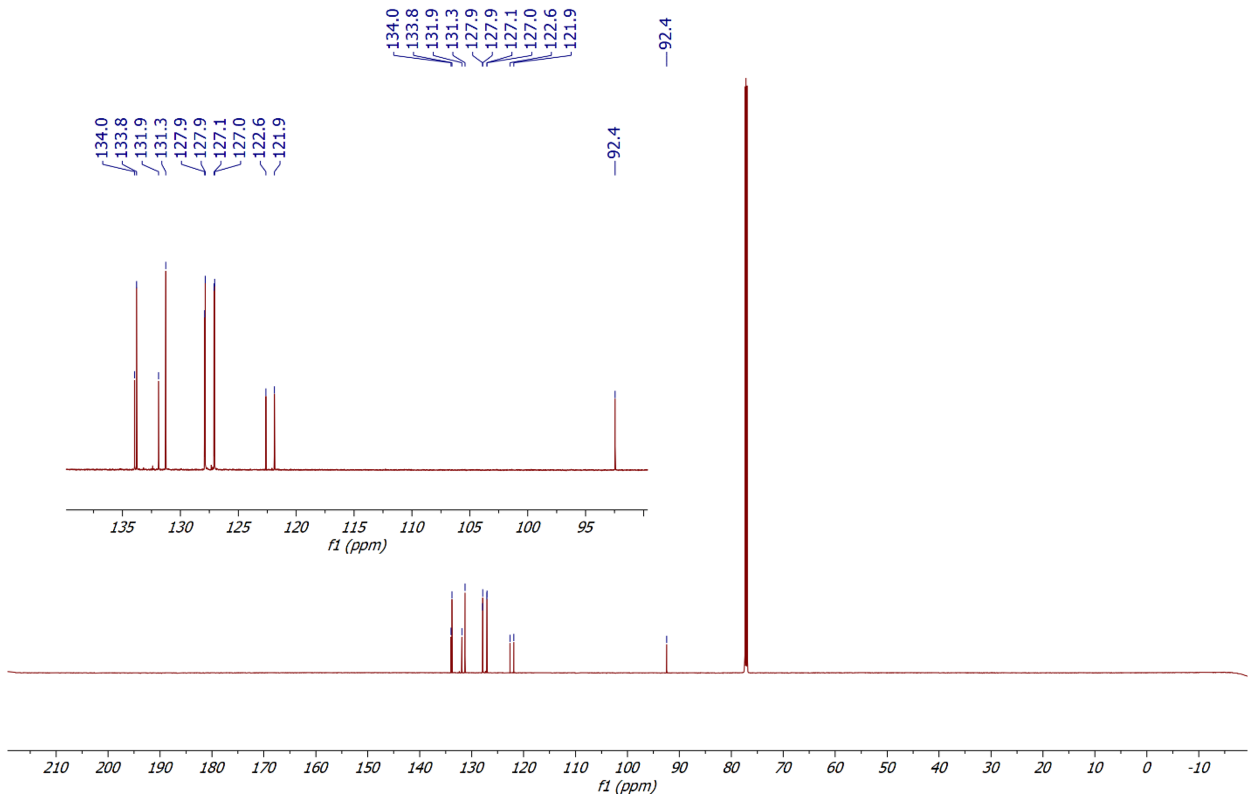

Figure S7. **$^{13}C\{^1H\}$ NMR** (151 MHz, $CDCl_3$, 298K) of **2a**.

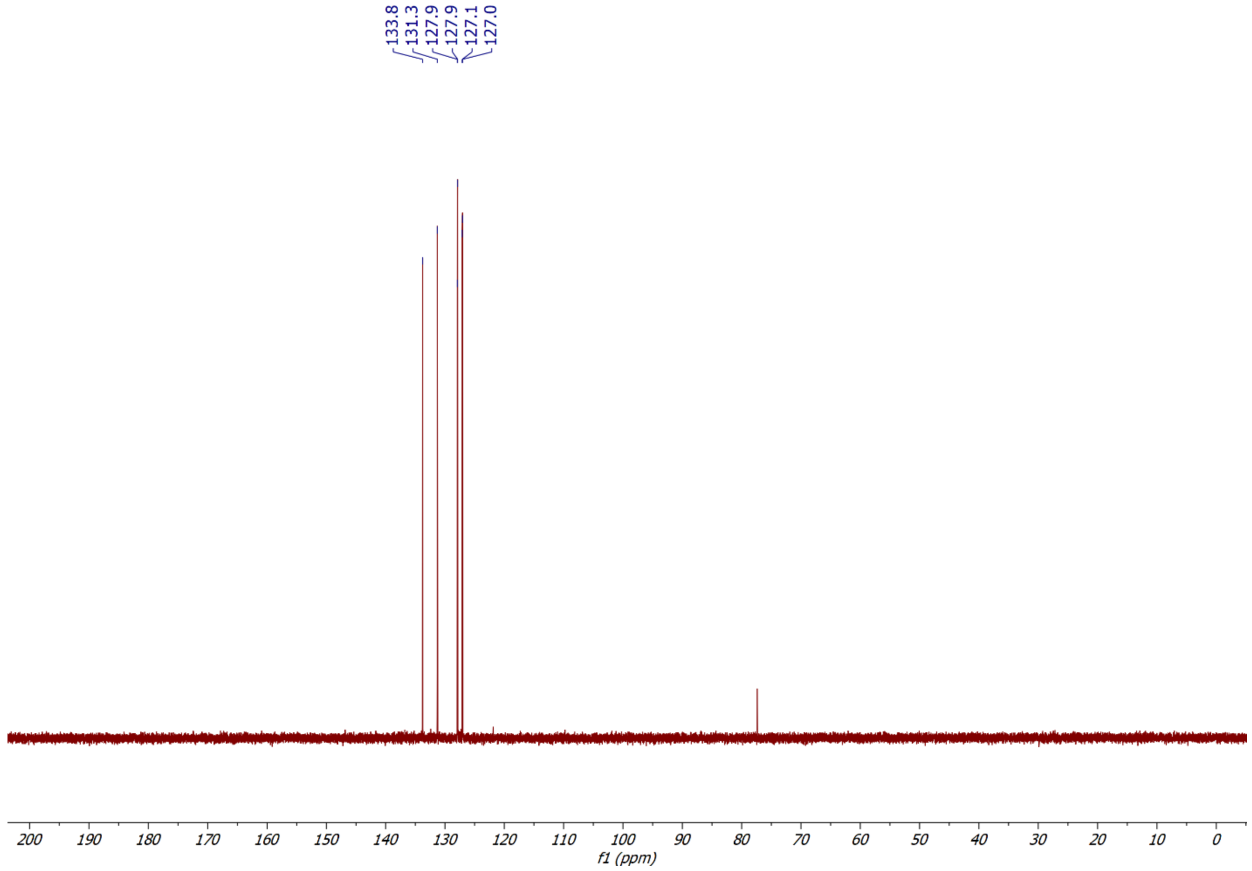

Figure S8. **^{13}C DEPT-135$\{^1H\}$ NMR** (151 MHz, $CDCl_3$, 298K) of **2a**.

2a (2.00 g, 5.18 mmol, 1.00 equiv.) was dissolved in diethyl ether (150 ml) under an argon atmosphere and cooled to -20°C. Then a 2.5 M solution of *n*-BuLi in hexane (4.14 ml, 110.4 mmol, 2.00 equiv.) was added. The mixture was stirred 30 min at this temperature. Then the reaction solution was warmed to 0 °C and stirred 10 min. Afterwards the reaction solution was cooled to -40 °C and *i*Pr_2PCl (1.73 ml, 10.9 mmol, 2.10 equiv.) were added. The reaction mixture was stirred at -20 °C for 2 days. After cold filtration over a silica plug and washing with pentane a green power (1.15 g, 2.49 mmol, 48%) was obtained. Due to high air sensitivity and temperature instability no further purification for **3a-Ph** was performed. **^{1}H NMR** (600 MHz, C_6D_6): δ (in ppm) = 8.40 (d, J_{H-H} = 2.5 Hz, 1H), 7.98-7.95 (m, 2H), 7.57 (dd, J_{H-H} = 8.1 Hz, J_{H-P} = 0.6 Hz 1H), 7.41 (dd, J_{H-H} = 8.1 Hz, J_{H-P} = 0.7 Hz, 1H), 7.33-7.31 (m, 1H), 7.18 (dt, J_{H-H} = 6.8 Hz, J_{H-P} = 1.3 Hz, 1H), 7.14 (dt, J_{H-H} = 7.5 Hz, J_{H-P} = 1.3 Hz, 1H), 7.06-7.01 (m, 2H). 2.46 (sept, J_{H-H} = 6.5 Hz, 2H), 2.17 (sept, J_{H-H} = 6.9 Hz, 2H), 1.25 (d, J_{H-H} = 7.0 Hz, 3H), 1.23 (d, J_{H-H} = 7.2 Hz, 3H), 1.21 (d, J_{H-H} = 7.2 Hz, 3H), 1.19 (d, J_{H-H} = 7.0 Hz, 3H), 1.04 (d, J_{H-H} = 7.0 Hz, 3H), 1.02 (d, J_{H-H} = 7.0 Hz, 3H), 0.99 (d, J_{H-H} = 6.9 Hz, 3H), 0.98 (d, J_{H-H} = 6.9 Hz, 3H). **^{31}P{^{1}H} NMR** (243 MHz, C_6D_6): δ (in ppm) = 7.60 (s, 1P), 3.60 (s, 1P). *Due to low thermal stability and large signal broadening, it was not possible to measure a reasonably resolved and pure ^{13}C{^{1}H} NMR spectrum*.

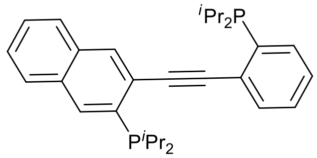

3a-Ph

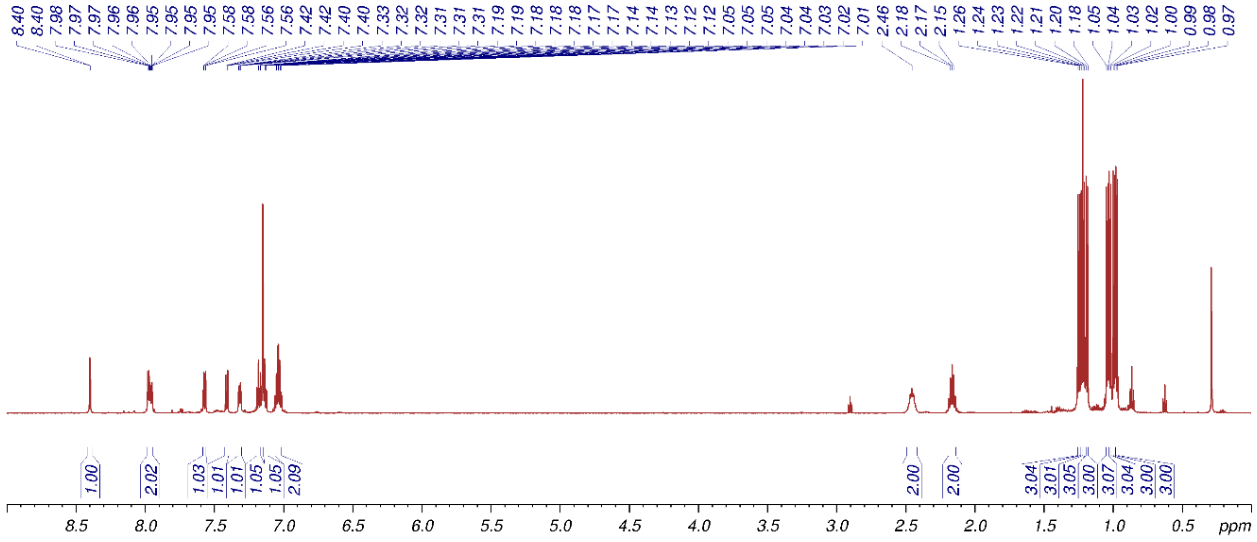

Figure S9. **^{1}H NMR** (600 MHz, $CDCl_3$, 298K) of **3a-Ph**.

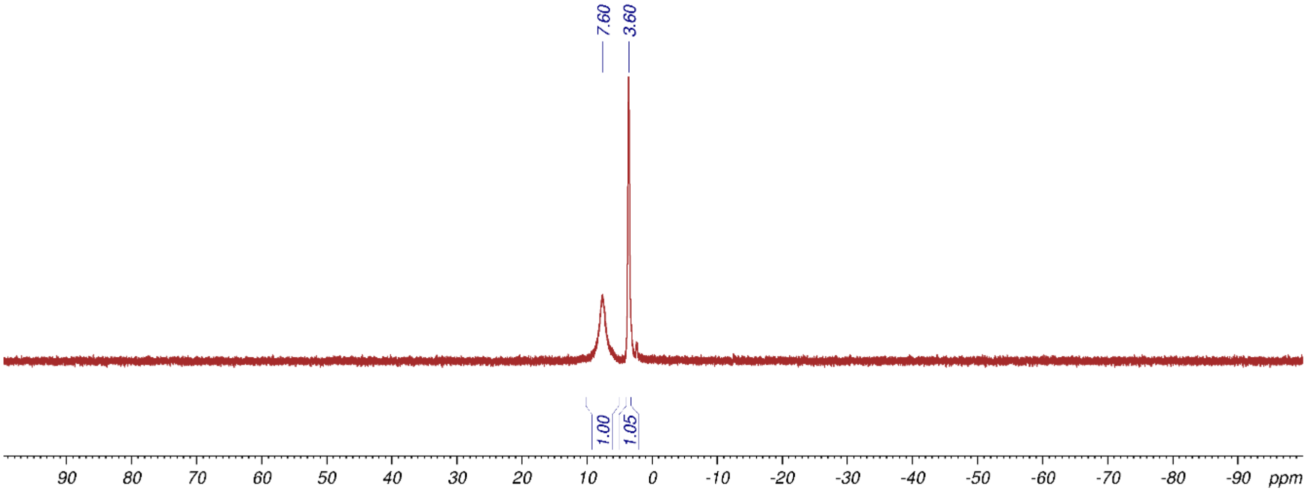

Figure S10. **^{31}P{^{1}H} NMR** (243 MHz, C_6D_6, 298K) of **3a-Ph**.

3a (1.96 g, 4.49 mmol, 1.0 eq) was dissolved in a Et_2O/ THF solution (1:1, 200 ml) under an argon atmosphere. The reaction mixture was cooled to -78 °C followed by the dropwise addition of 1.7 M *t*-BuLi solution in pentane (10.6 ml, 18.0 mmol, 4.0 eq). After the addition was completed the reaction solution was stirred for 2 hours at -78 °C. Then *i*Pr_2PCl (1.50 g, 9.44 mmol, 2.0 eq.) were added and the mixture was stirred for 3 days at -20 °C. The reaction solution was filtered over a silica plug. The filtrate was evaporated under vacuum and the precipitate was washed with pentane. The product was obtained as a yellow powder (0.51 g, 0.99 mmol, 22%). Due to high air sensitivity and temperature instability no further purification for **3a** was performed. **^{1}H NMR** (600 MHz, C_6D_6): δ(in ppm) = 8.47 (d, $J_{H\text{-}H}$ = 2.3 Hz, 2H), 7.99 (d, $J_{H\text{-}H}$ = 4.4 Hz, 2H), 7.60 (d, $J_{H\text{-}H}$ = 8.0 Hz, 2H), 7.47 (d, $J_{H\text{-}H}$ = 8.0 Hz, 2H), 7.20 (dt, $J_{H\text{-}H}$ = 8.1 Hz, $J_{H\text{-}P}$ = 1.2 Hz, 2H), 7.16 (dt, $J_{H\text{-}H}$ = 8.2 Hz, $J_{H\text{-}H}$ = 1.2 Hz, 2H), 2.45 (sept, $J_{H\text{-}H}$ = 6.8 Hz, 4H), 1.28 (d, $J_{H\text{-}H}$ = 7.0 Hz, 6H), 1.25 (d, $J_{H\text{-}H}$ = 7.0 Hz, 6H), 1.07 (d, $J_{H\text{-}H}$ = 7.0 Hz, 6H), 1.05 (d, $J_{H\text{-}H}$ = 7.0 Hz, 6H). **^{31}P{^{1}H} NMR** (243 MHz, C_6D_6): δ(in ppm) = 6.57 (s). *Due to low thermal stability and large signal broadening, it was not possible to measure a reasonably resolved and pure ^{13}C{^{1}H} NMR spectrum*.

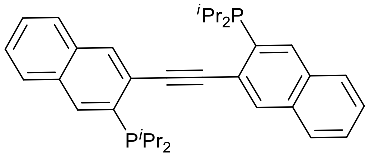

3a

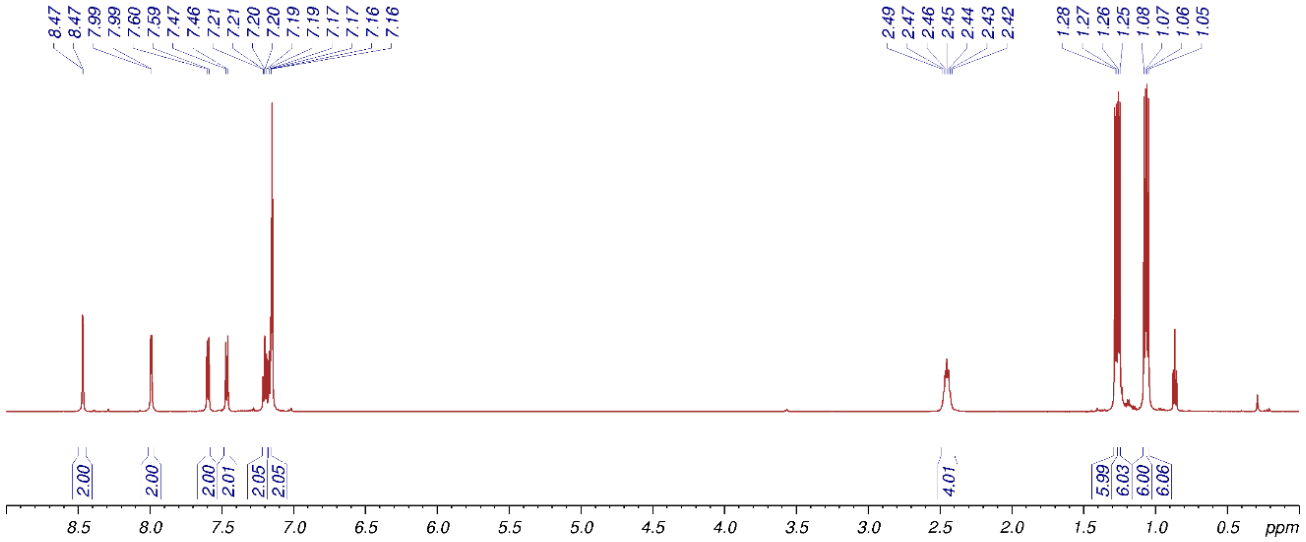

Figure S11. **^{1}H NMR** (600 MHz, $CDCl_3$, 298K) of **3a**.

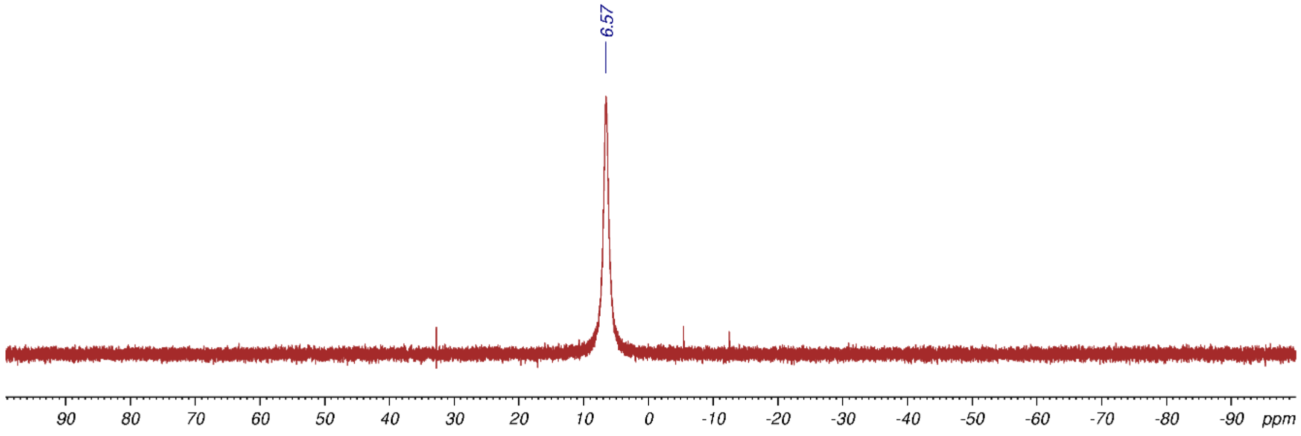

Figure S12. **^{31}P{^{1}H} NMR** (243 MHz, C_6D_6, 298K) of **3a**.

3a-Ph (0.50 g, 1.09 mmol, 1.00 equiv.) and C_2Cl_6 (0.26 g, 1.09 mmol, 1.00 equiv.) were placed in a flask under an argon atmosphere and dissolved in 50 ml DCM. The reaction mixture was stirred overnight at room temperature. The precipitation was filtered off and washed with a mixture of DCM and NEt_3 (10 ml DCM and three drops NEt_3). After washing with additional DCM and drying under high vacuum, the product was obtained as a green powder (0.45 g, 0.97 mmol, 90%). **IR** $\tilde{\upsilon}$ [cm^{-1}] = 3388, 3056, 2966, 2861, 2330, 2087, 1616, 1500, 1454, 1435,1391, 1370, 1359, 1337, 1253, 1224, 1157, 1133, 973, 919, 683, 649, 618. **^{1}H NMR** (600 MHz,CD_3OD): δ (in ppm) = 8.84 (dd, J_{H-H} = 10.4 Hz, J_{H-P} = 1.6 Hz, 1H), 8.41 (d, J_{H-P} = 3.4 Hz, 1H), 8.25 (t, J_{H-H} = 8.0 Hz, 1H), 8.18 (d, J_{H-H} = 8.4 Hz, 2H), 8.04 (t, J_{H-H} = 8.0 Hz, 1H), 8.00-7.98 (m, 1H), 7.90-7.82 (m, 3H), 3.79-3.73 (m, 2H), 3.73-3.67 (m, 2H), 1.47 (dd, J_{H-P} = 20.0 Hz, J_{H-H} = 7.0 Hz, 12H), 1.42-1.34 (m, 12H). **^{13}C{^{1}H} NMR** (151 MHz, D_2O): δ (in ppm) = 146.82 (dd, J_{C-P} = 64.2 Hz, J_{C-P} = 9.9 Hz, C_q, 1C,), 144.89 (dd, J_{C-P} = 64.9 Hz, J_{C-P} = 11.8 Hz, C_q, 1C), 138.92 (dd, J_{C-P} = 14.3 Hz, J_{C-P} = 13.8 Hz, C_q, 1C), 138.04 (d, J_{C-P} = 10.3 Hz, CH, 1C), 136.92 (d, J_{C-P} = 1.9 Hz, CH, 1C), 135.47 (d, J_{C-P} = 1.4 Hz, CH, 1C), 134.19 (d, J_{C-P} = 10.4 Hz, CH, 1C), 133.07 (dd, J_{C-P} = 14.6 Hz, J_{C-P} = 11.6 Hz, C_q, 1C), 132.29 (d, J_{C-P} = 11.2 Hz, CH, 1C), 131.46 (s, CH, 1C), 130.46 (s, CH, 1C), 129.82 (d, J_{C-P} = 11.0 Hz, CH, 1C), 128.36 (d, J_{C-P} = 6.5 Hz, CH, 1C), 127.17 (d, J_{C-P} = 7.0 Hz, CH, 1C), 119.73 (dd, J_{C-P} = 84.1 Hz, J_{C-P} = 8.7 Hz, C_q, 1C), 119.42 (dd, J_{C-P} = 84.6 Hz, J_{C-P} = 11.8 Hz, C_q, 1C), 115.46 (dd, J_{C-P} = 86.7 Hz, J_{C-P} = 8.7 Hz, C_q, 1C), 22.92 (d, J_{C-P} = 36.9 Hz, CH, 2C), 22.59 (d, J_{C-P} = 35.7 Hz, CH, 2C), 15.37 (dd, J_{C-P} = 26.5 Hz, J_{C-P} = 2.9 Hz, CH_3, 4C), 14.71 (dd, J_{C-P} = 17.8 Hz, J_{C-P} = 2.7 Hz, CH_3, 4C).**^{31}P{^{1}H} NMR** (243 MHz, D_2O): δ (in ppm) = 58.15 (d, J_{P-P} = 24.8 Hz, 1P), 56.21 (d, J_{P-P} = 24.8 Hz, 1P). **ESI HR MS** (pos): m/z calcd for [$C_{30}H_{38}P_2$+OH]$^+$: 477.2471, found: 477.2473 [$C_{30}H_{38}P_2$+OH]$^+$.

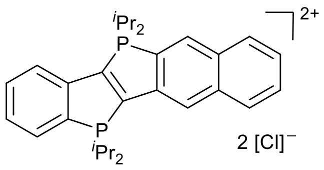

[4a-Ph]$^{2+}$

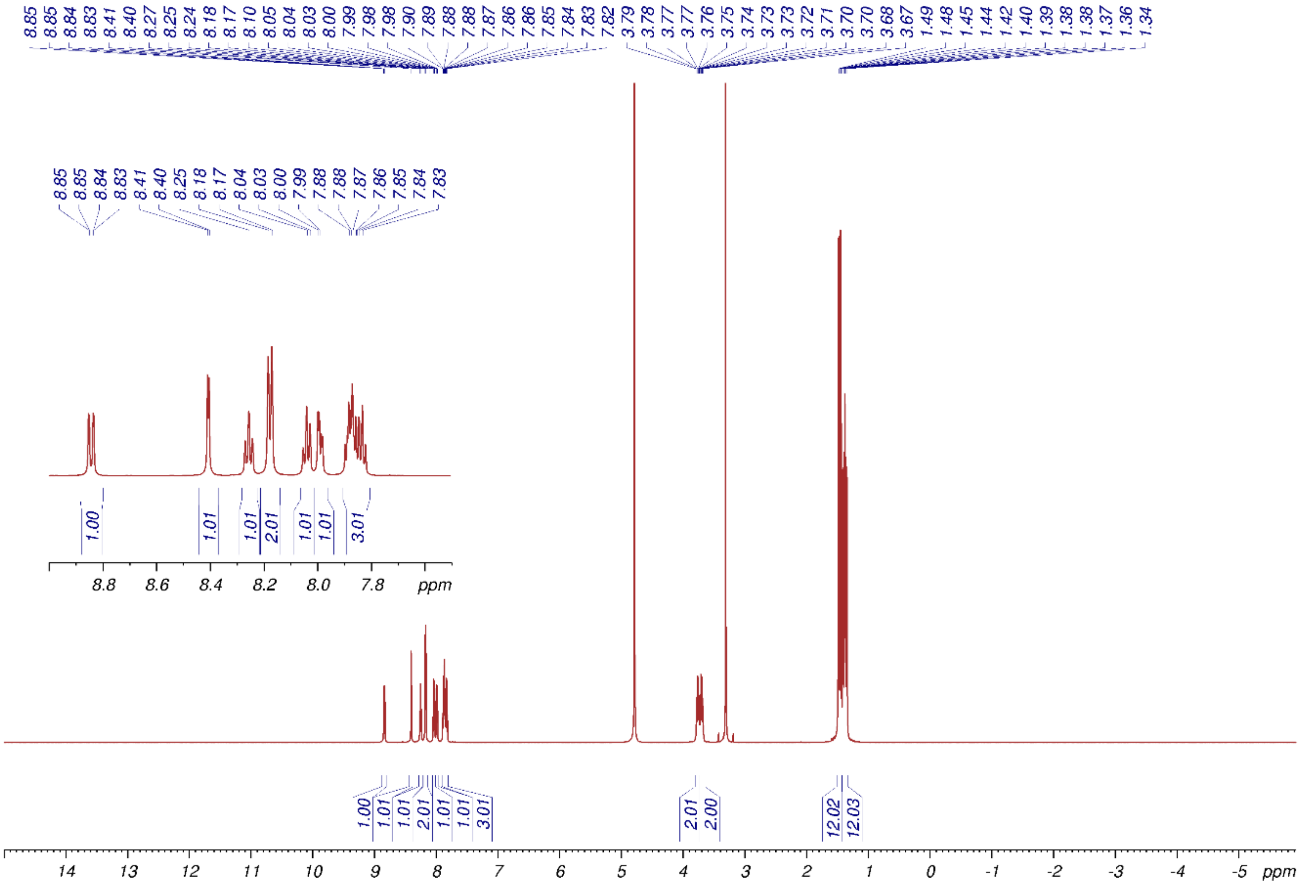

Figure S13. **^{1}H NMR** (600 MHz, CD_3OD, 298K) of [**4a-Ph**]$^{2+}$.

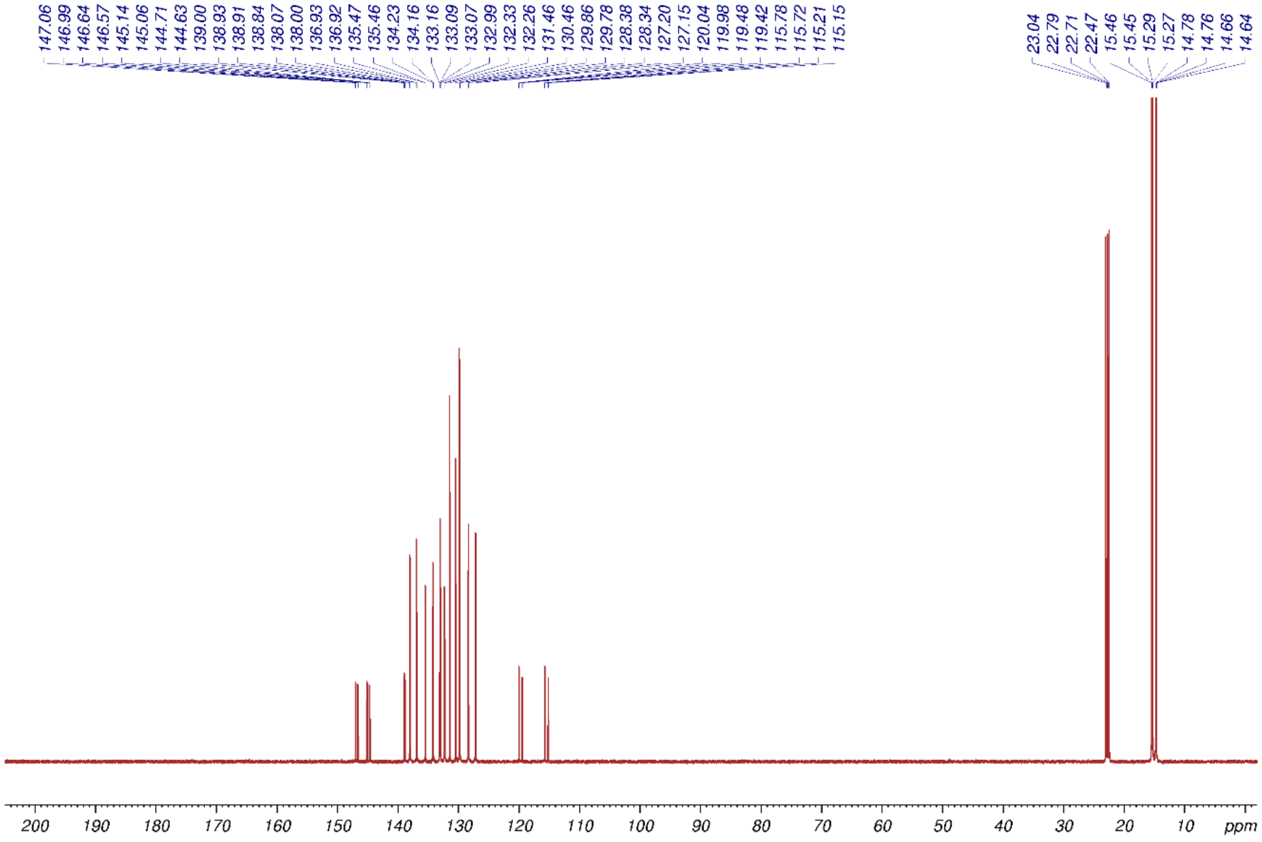

Figure S14. **^{13}C{^{1}H} NMR** (151 MHz, D_2O, 298K) of [**4a-Ph]$^{2+}$**.

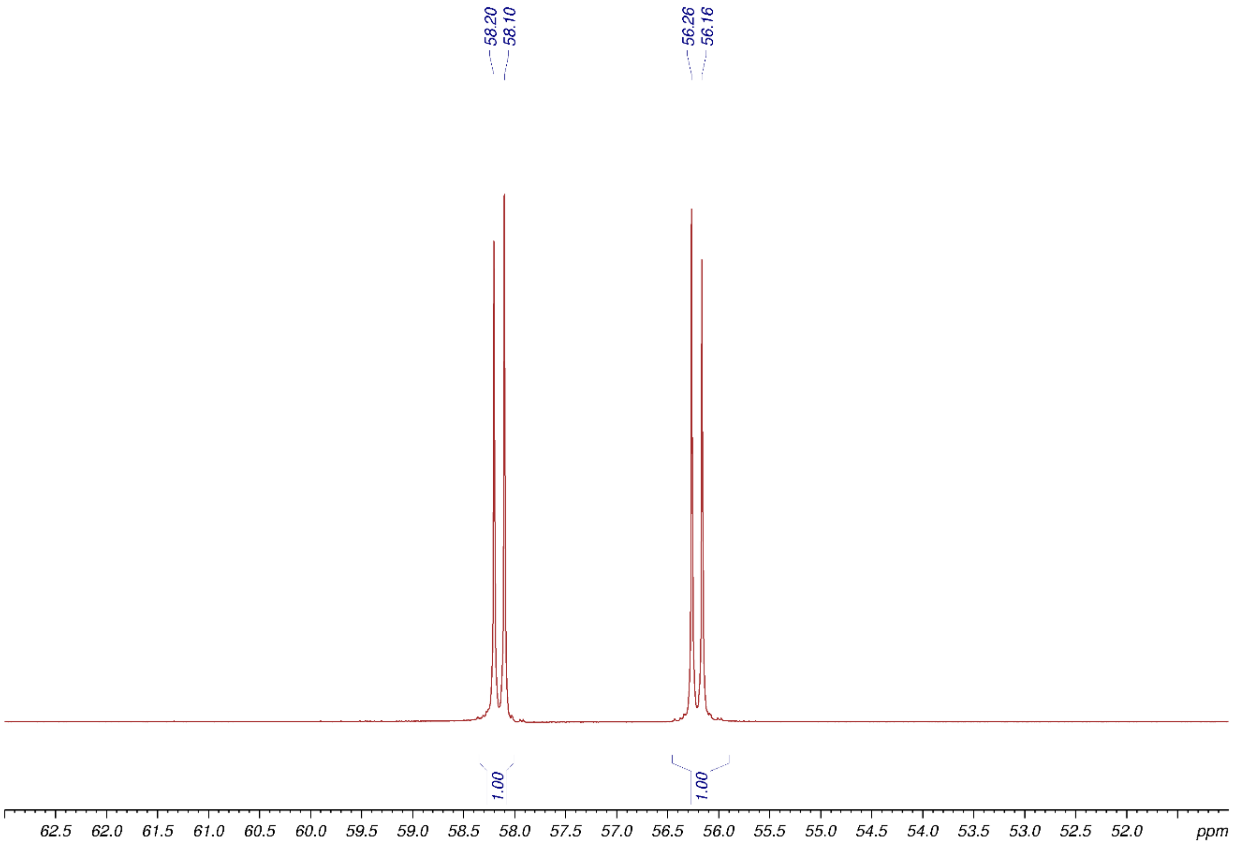

Figure S15. **^{31}P{^{1}H} NMR** (243 MHz, D_2O, 298K) of **[4a-Ph]$^{2+}$**.

4a (0.30 g, 0.59 mmol, 1.00 equiv.) and C_2Cl_6 (0.14 g, 0.59 mmol, 1.00 equiv.) were placed in a flask under an argon atmosphere and dissolved in 50 ml DCM. The reaction mixture was stirred overnight at room temperature. The precipitation was filtered off and washed with a mixture of DCM and NEt_3 (10 ml DCM and three drops NEt_3). After washing with additional DCM and drying under high vacuum, the product was obtained as a yellow powder (0.26 g, 0.49 mmol, 85%). **IR** $\tilde{\upsilon}$ [cm^{-1}] = 3385, 2956, 2852, 2331, 2163, 2107, 1875, 1620, 1495, 1440, 1410, 1223, 1150, 1132, 1018, 921, 860, 753, 705, 679, 616. **^{1}H NMR** (600 MHz, D_2O): δ (in ppm) = 8.87-8.85 (m, 2H), 8.40 (d, $J_{H\text{-}P}$ = 2.8 Hz, 2H), 8.20-8.17 (m, 4H), 7.90 (dt, $J_{H\text{-}H}$ = 8.1 Hz, $J_{H\text{-}P}$ = 1.2 Hz, 2H), 7.86 (dt, $J_{H\text{-}H}$ = 8.1 Hz, $J_{H\text{-}P}$ = 1.2 Hz, 2H), 3.81-3.73 (m, 4H), 1.51 (dd, $J_{H\text{-}P}$ = 19.3 Hz, $J_{H\text{-}H}$ = 7.0 Hz, 12H), 1.42 (dd, $J_{H\text{-}P}$ = 19.7 Hz, $J_{H\text{-}H}$ = 6.9 Hz, 12H). **^{13}C{^{1}H} NMR** (151 MHz, D_2O): δ (in ppm) = 145.25 (dd, $J_{C\text{-}P}$ = 74.6 Hz, $J_{C\text{-}P}$ = 7.2 Hz, C_q, 2C), 138.03 (dd, $J_{C\text{-}P}$ = 6.0 Hz, $J_{C\text{-}P}$ = 5.4 Hz, CH, 2C), 135.53 (s, C_q, 2C), 133.41 (dd, $J_{C\text{-}P}$ = 14.0 Hz, $J_{C\text{-}P}$ = 13.2 Hz, C_q, 2C), 132.97 (dd, $J_{C\text{-}P}$ = 6.8 Hz, $J_{C\text{-}P}$ = 6.5 Hz, C_q, 2C), 131.43 (s, CH, 2C), 130.34 (s, CH, 2C), 129.82 (s, CH, 2C), 129.67 (s, CH, 2C), 128.00 (dd, $J_{C\text{-}P}$ = 4.0 Hz, $J_{C\text{-}P}$ = 3.2 Hz, CH, 2C), 115.44 (dd, $J_{C\text{-}P}$ = 92.6 Hz, $J_{C\text{-}P}$ = 5.4 Hz, C_q, 2C), 23.23-22.83 (m, CH, 4C), 15.43 (br s, CH_3, 4C), 14.64 (br s, CH_3, 4C). **^{31}P{^{1}H} NMR** (243 MHz, D_2O): δ (in ppm) = 55.92. ESI HR MS (pos): m/z calcd for $[H_{34}H_{40}P_2+OH]^+$: 527.2627, found: $[H_{34}H_{40}P_2+OH]^+$: 527.2629.

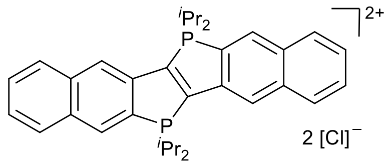

[4a]$^{2+}$

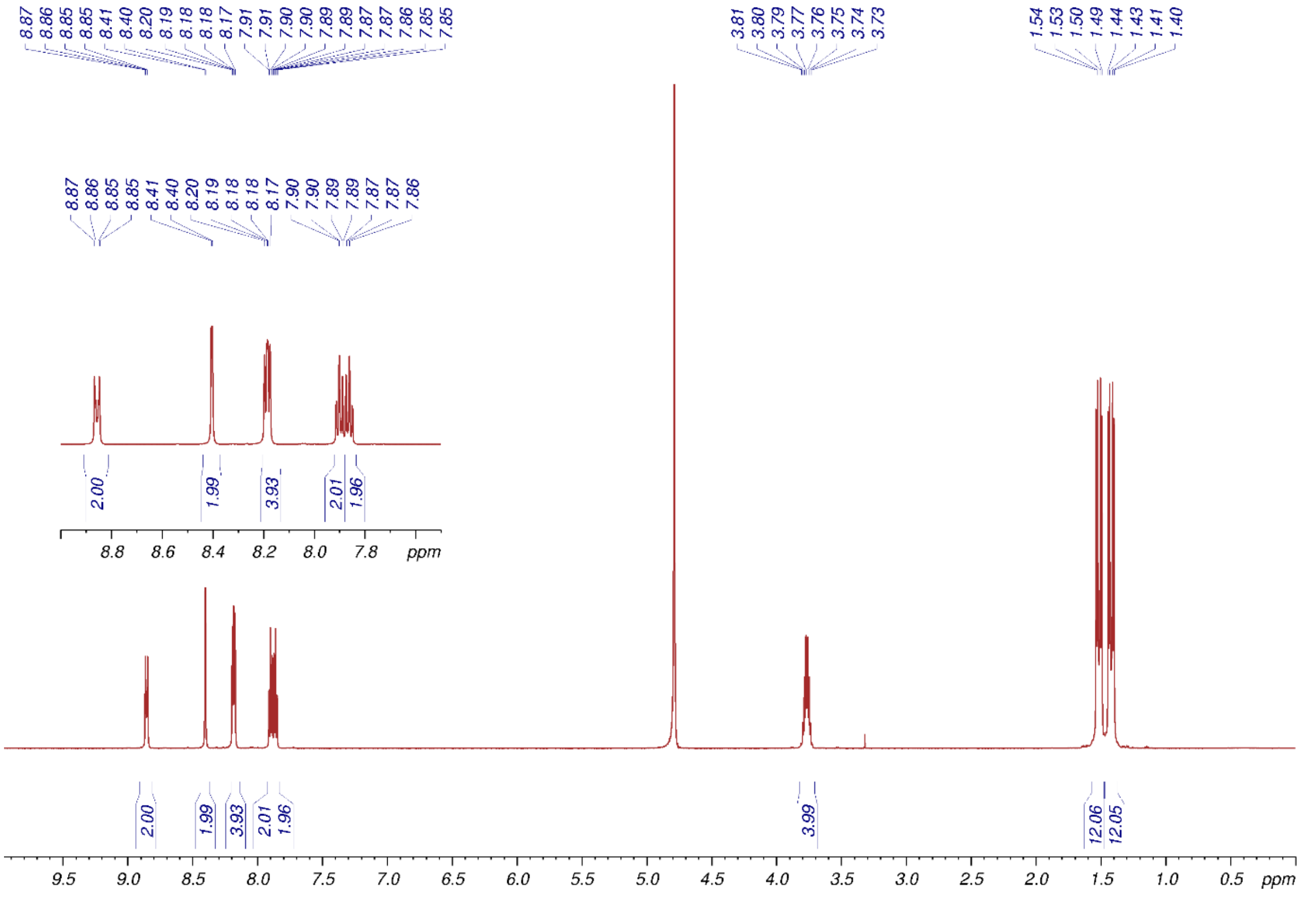

Figure S16. **^{1}H NMR** (600 MHz, D_2O, 298K) of **[4a]$^{2+}$**.

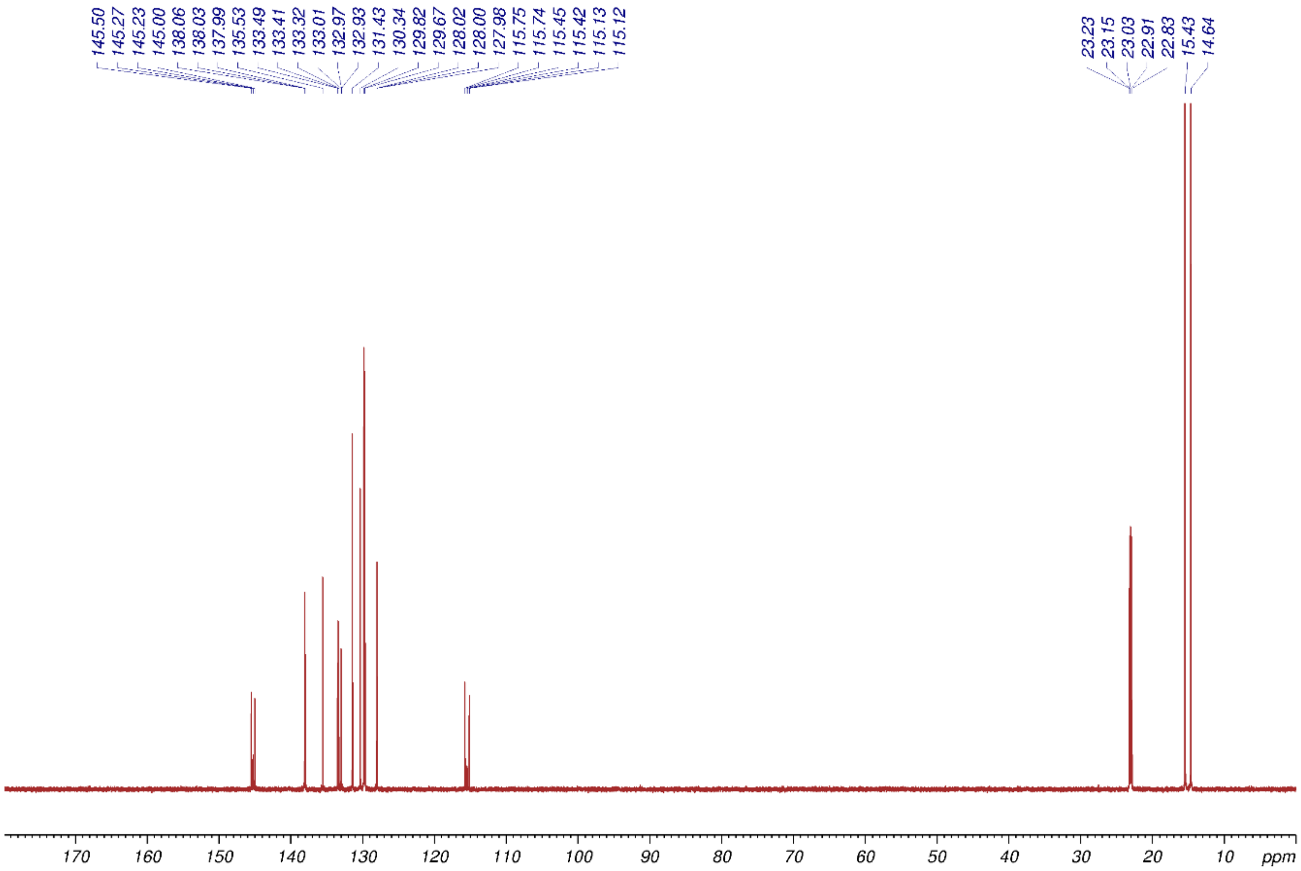

Figure S17. **^{13}C{^{1}H} NMR** (151 MHz, D_2O, 298K) spectrum of **[4a]$^{2+}$**.

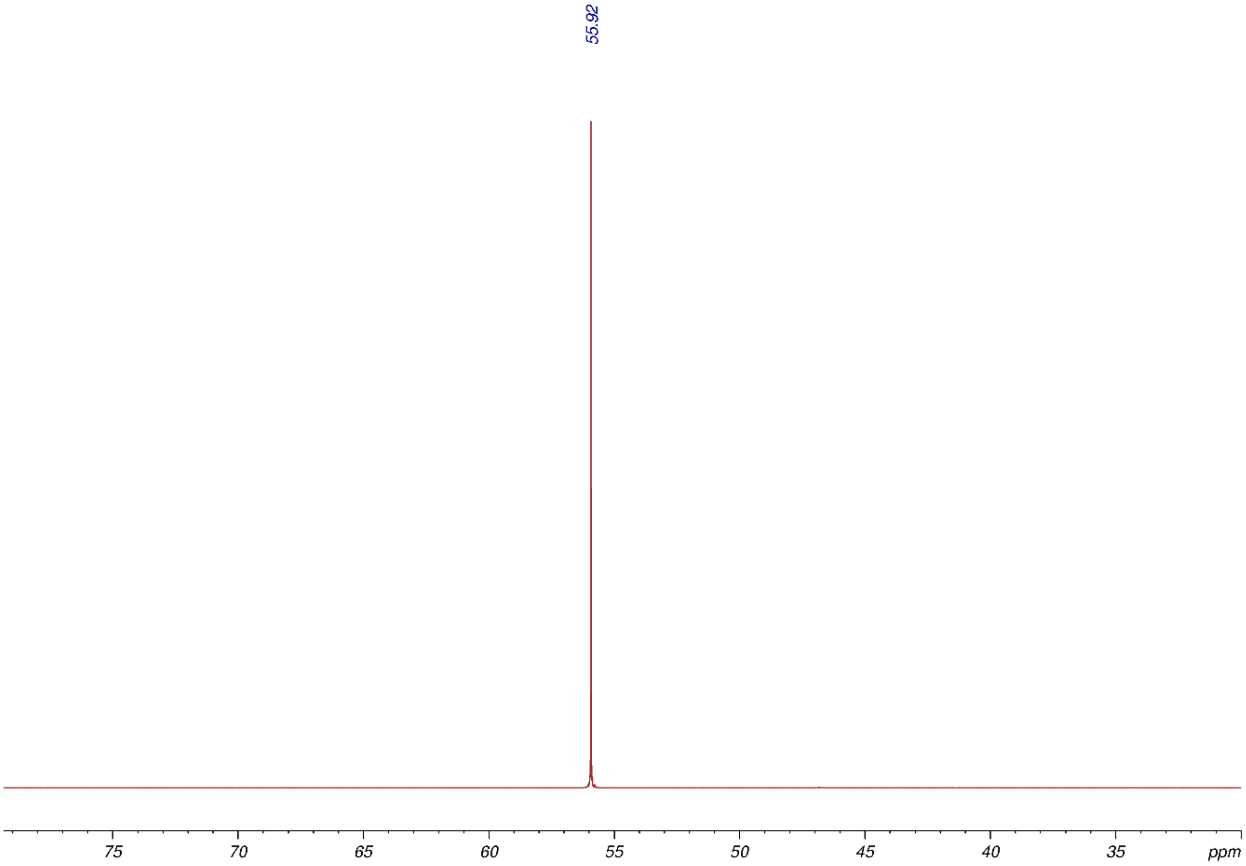

Figure S18. **^{31}P{^{1}H} NMR** (243 MHz, D_2O, 298K) spectrum of **[4a]$^{2+}$**.

1.4) Synthesis of [4b-Ph]$^{2+}$, [*iso*-4b]$^{2+}$ and [4b]^{0} from 1b

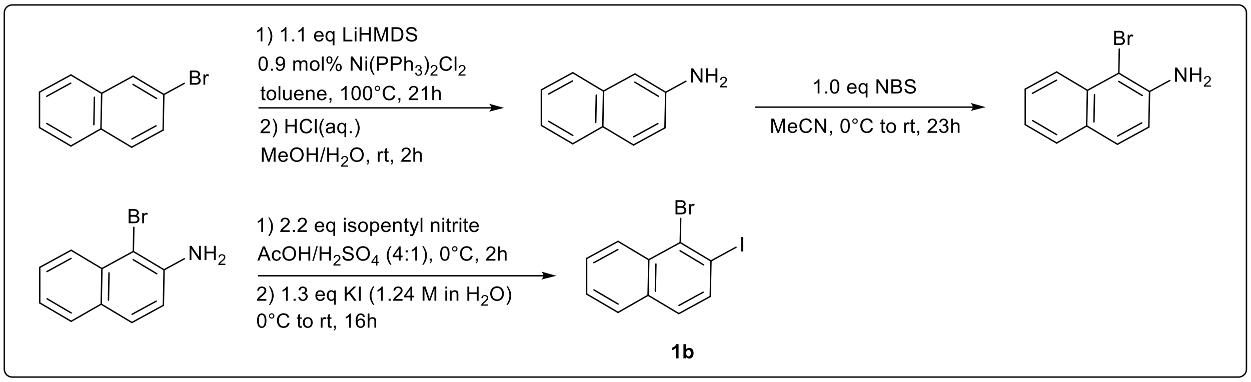

Scheme S3. Synthetic route for the synthesis of compound **1b**.

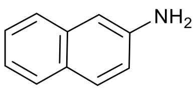

Naphthalen-2-amine was synthesized according to a modified procedure of *Fout* and coworkers[7]. Lithium bis(trimethylsilyl)amide (17.8 g, 106 mmol, 1.1 eq.), 2-bromonaphthalene (20.0 g, 96.6 mmol, 1.0 eq.), and dichloro-bis-(triphenylphosphin)-nickel (632 mg, 966 μmol, 0.9 mol%) were dissolved in dry toluene (300 mL). The reaction mixture was stirred at 100 °C for 21 h. After cooling the mixture to rt, MeOH (300 mL) and 1 M HCl (200 mL) were added. The reaction mixture was stirred at rt for 2 h and was neutralized with 1 M KOH solution. Methanol was removed under reduced pressure and the mixture was extracted with dichloromethane (10 × 100 mL). The combined organic phase was dried (Na_2SO_4), filtered and the solvent was removed under reduced pressure. Naphthalen-2-amine was obtained as an off-white solid (13.6 g, 95.0 mmol, 98%). **^{1}H NMR** (600 MHz, $CDCl_3$): δ (in ppm) = 7.69 (d, $J_{H\text{–}H}$ = 8.1 Hz, 1H), 7.66 (d, $J_{H\text{–}H}$ = 8.6 Hz, 1H), 7.59 (d, $J_{H\text{–}H}$ = 8.2 Hz, 1H), 7.36 (ddd, $J_{H\text{–}H}$ = 8.2, 6.8, 1.3 Hz, 1H), 7.22 (ddd, $J_{H\text{–}H}$ = 8.1, 6.8, 1.2 Hz, 1H), 6.99 (d, $J_{H\text{–}H}$ = 2.3 Hz, 1H), 6.95 (dd, $J_{H\text{–}H}$ = 8.6, 2.3 Hz, 1H), 3.84 (s, 2H). *These data are in accordance with the literature*[7].

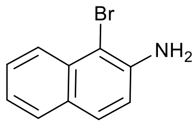

1-Bromonaphthalen-2-amine was synthesized according to a modified procedure of *Lang* and coworkers[8]. Naphthalen-2-amine (12.0 g, 83.8 mmol, 1.0 eq) was dissolved in dry acetonitrile (250 mL) and cooled to 0 °C. N-bromosuccinimide (14.9 g, 83.8 mmol, 1.0 eq) was added at once. The reaction mixture was stirred at rt for 23 h. The mixture was diluted with H_2O and ethyl acetate (100 mL each) and the phases were separated. The aqueous phase was extracted with ethyl acetate (4 × 200 mL). The combined organic layer was washed with H_2O (3 × 50 mL), brine (3 × 50 mL), 1 M NaOH solution (2 × 50 mL) and was dried over $MgSO_4$. The solvent was removed under reduced pressure and 1-bromonaphthalen-2-amine was obtained as a dark brown solid (17.2 g, 77.4 mmol, 92%). **^{1}H NMR** (600 MHz, $CDCl_3$): δ (in ppm) = 8.04 (d, $J_{H\text{–}H}$ = 8.5 Hz, 1H), 7.69 (d, $J_{H\text{–}H}$ = 8.0 Hz, 1H), 7.63 (d, $J_{H\text{–}H}$ = 8.7 Hz, 1H), 7.51 (ddd, $J_{H\text{–}H}$ = 8.4, 6.9, 1.3 Hz, 1H), 7.29 (ddd, $J_{H\text{–}H}$ = 8.0, 6.8, 1.1 Hz, 1H), 7.01 (d, $J_{H\text{–}H}$ = 8.7 Hz, 1H), 4.38 (s, 2H). *This data are in accordance with literature*[9].

1-Bromo-2-iodonaphthalene was synthesized after a modified procedure of *Haggam* and coworkers[10]. 1-Bromonaphthalen-2-amine (17.0 g, 76.6 mmol, 1.0 eq.) was dissolved in a mixture of acetic acid and sulfuric acid (4:1, 250 mL) in a three-necked flask equipped with an internal thermometer and a dropping funnel. The mixture was cooled to 0 °C and isopentyl nitrite (19.7 g, 22.6 mL, 168 mmol, 2.2 eq.) was added dropwise over 1 h while the internal temperature was kept below 7 °C. The mixture was stirred for 2 h. A solution of potassium iodine (16.5 g, 99.5 mmol, 1.3 eq.) in water (80 mL) was added over 1 h while the internal temperature was kept below 12 °C. The reaction mixture was stirred at rt for 16 h. Afterwards the mixture was poured into water, neutralized with a 3 M aq. NaOH solution and extracted with dichloromethane (15 × 100 mL). The combined organic layer was washed with H_2O (2 × 200 mL), brine (2 × 200 mL), and was dried over $MgSO_4$. The solvent was removed under reduced pressure and purification by column chromatography (PE/DCM = 20:1) afforded 1-bromo-2-iodonaphthalene as an off-white solid (6.20 g, 18.6 mmol, 24%). **Mp:** 92.3 °C. **^{1}H NMR** (600 MHz, $CDCl_3$): δ (in ppm) = 8.31 (d, $J_{H–H}$ = 8.4 Hz, 1H), 7.88 (d, $J_{H–H}$ = 8.6 Hz, 1H), 7.79 (d, $J_{H–H}$ = 7.2 Hz, 1H), 7.58–7.52 (m, 3H). **^{13}C{^{1}H} NMR** (151 MHz, $CDCl_3$): δ (in ppm) = 136.3 (CH, 1C), 133.5 (C_q, 1C), 133.5 (C_q, 1C), 130.1 (C_q, 1C), 129.1 (CH, 1C), 129.0 (CH, 1C), 128.5 (CH, 1C), 128.4 (CH, 1C), 127.1 (CH, 1C), 101.0 (C_q, 1C). **EI HR-MS** (pos): *m/z* calcd for $C_{10}H_6BrI^+$ [M]$^+$ 331.8692, found 331.8693; calcd $C_{10}H_6Br^+$ [M-I]$^+$ 204.9647, found 204.9654; calcd $C_{10}H_6^+$ [M-I-Br]$^+$ 126.0464, found 126.0465.

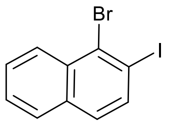

1b

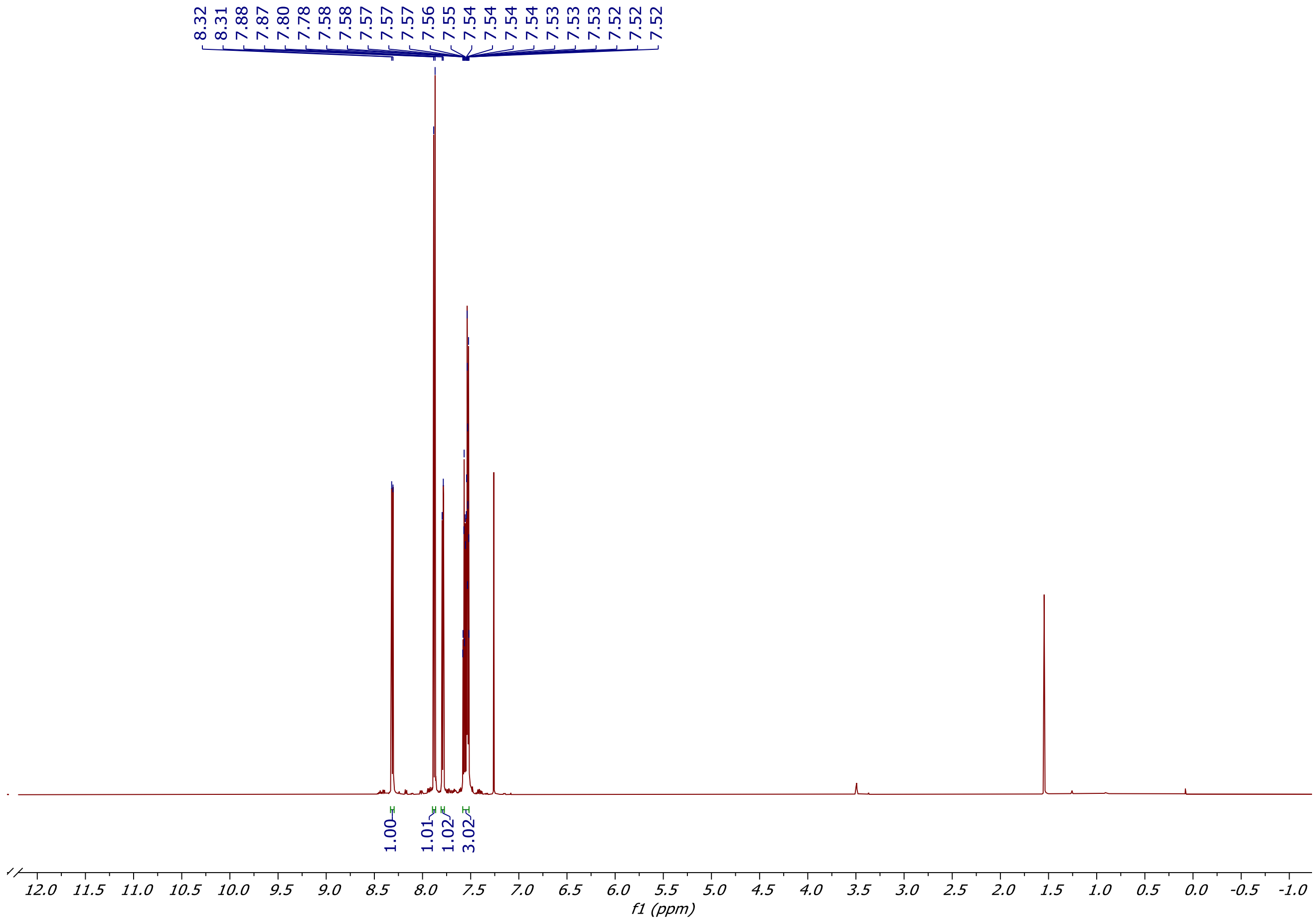

Figure S19. **^{1}H NMR** (600 MHz, $CDCl_3$, 298K) spectrum of **1b**.

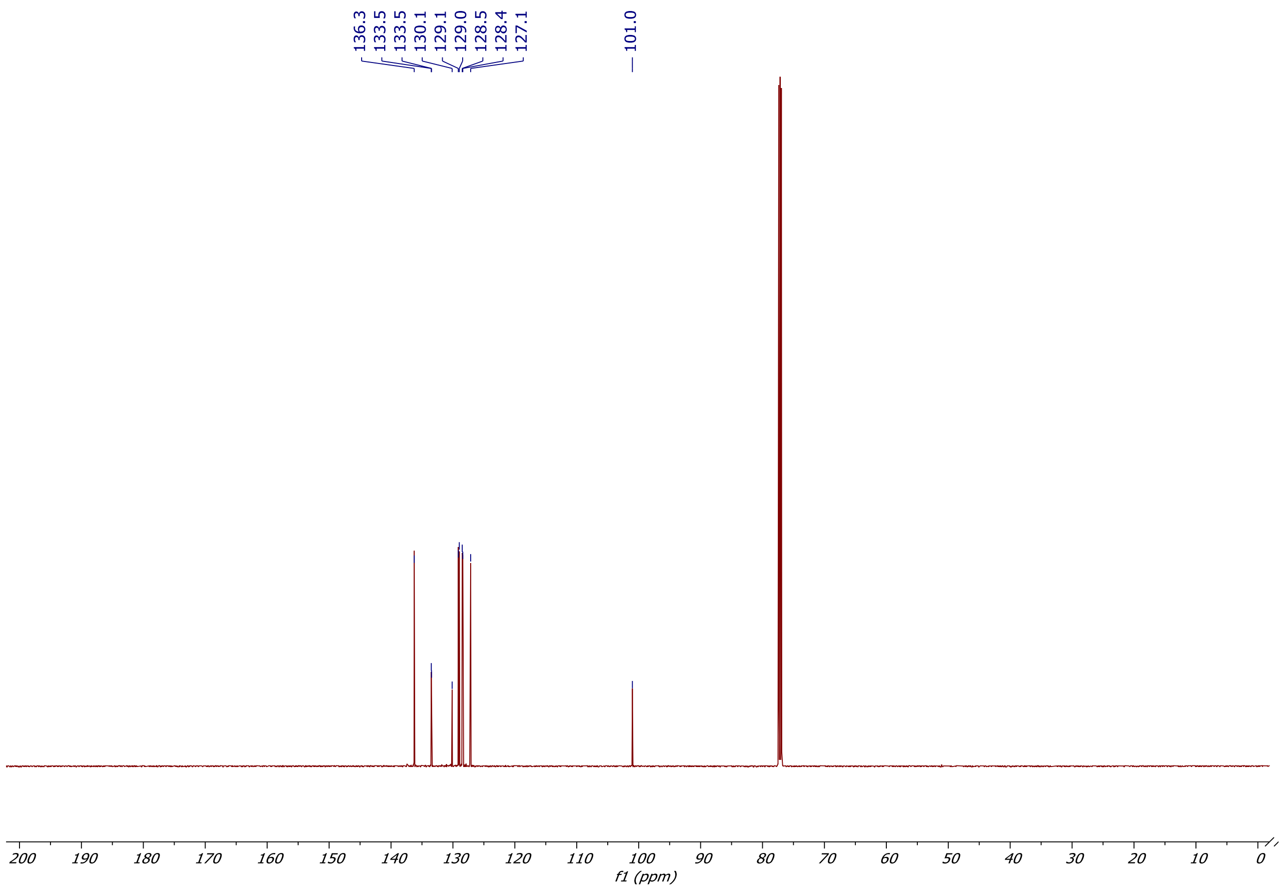

Figure S20.^{13}**C{^{1}H} NMR** (151 MHz, $CDCl_3$, 298K) spectrum of **1b**.

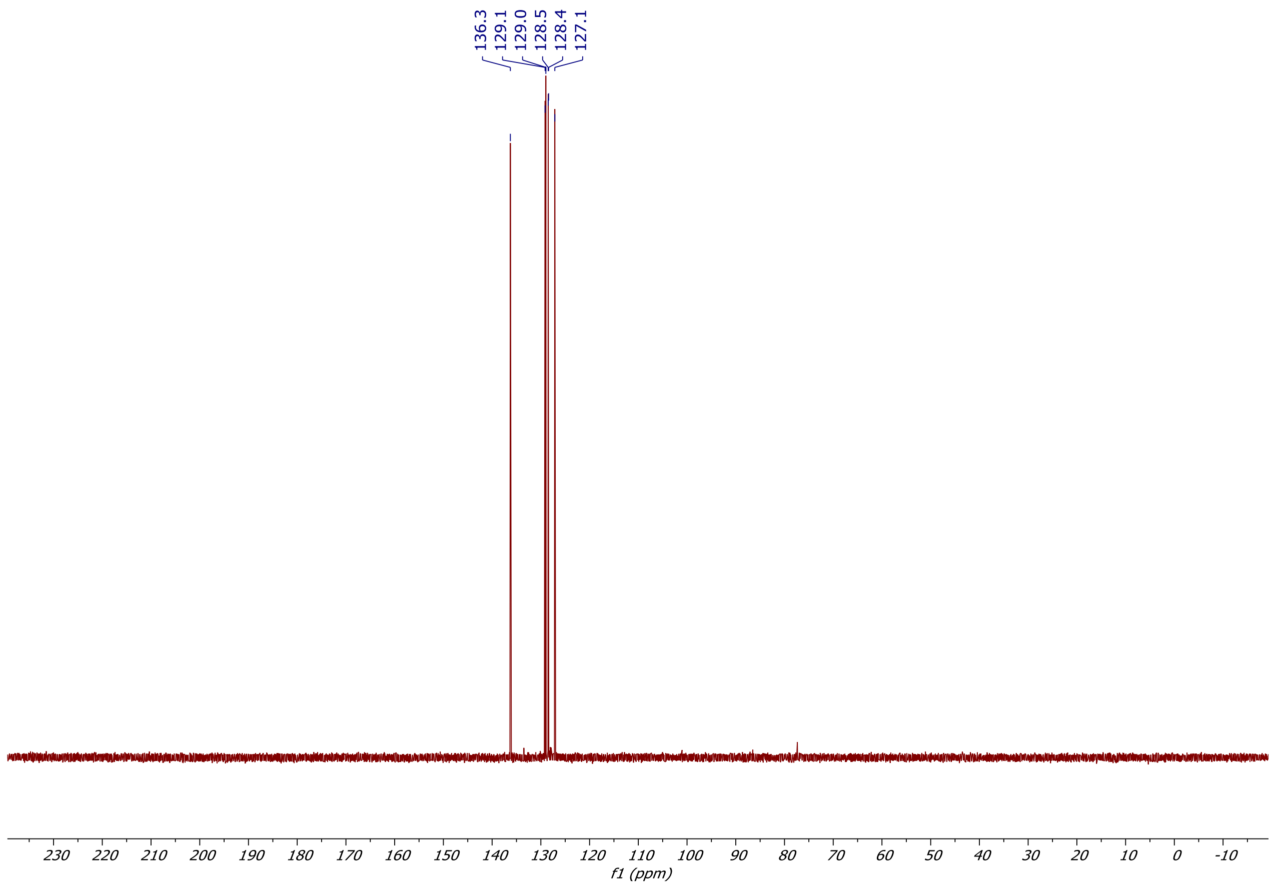

Figure S21.^{13}**C{^{1}H} DEPT-135 NMR** (151 MHz, $CDCl_3$, 298K) spectrum of **1b**.

1c (3.60 g, 10.8 mmol, 1.0 eq.), $Pd(PPh_3)_4$ (313 mg, 270 µmol, 2.5 mol%), and 1,2-bis(tributylstannyl)ethyne (3.27 g, 2.84 ml, 5.41 mmol, 2.0 eq) were stirred in dry 1,4-dioxane (70 mL) at 100 °C for 15 h. After the mixture cooled to room temperature, the solvent was removed under reduced pressure. Purification of the crude product, performed by column chromatography (SiO_2, PE/DCM, 20:1 + 1% Et_3N) afforded **2b** as an off-white solid (1.42 g, 60%). **^{1}H NMR** (600 MHz, $CDCl_3$): δ (in ppm) = 8.36–8.34 (m, 2H), 7.84–7.80 (m, 4H), 7.70 (d, $J_{H\text{–}H}$ = 8.4 Hz, 2H), 7.64 (ddd, $J_{H\text{–}H}$ = 8.4, 6.9, 1.3 Hz, 2H), 7.56 (ddd, $J_{H\text{–}H}$ = 8.0, 6.9, 1.2 Hz, 2H). **^{13}C {^{1}H} NMR** (151 MHz, $CDCl_3$): δ (in ppm) = 134.0 (C_q, 2C), 132.4 (C_q, 2C), 129.3 (CH, 2C), 128.4 (CH, 2C), 128.1 (CH, 2C), 128.0 (CH, 2C), 127.7 (CH, 2C), 127.5 (CH, 2C), 126.9 (C_q, 2C), 123.4 (C_q, 2C), 94.6 (C_q, 2C). **EI HR-MS** (pos): m/z calcd for $C_{22}H_{12}Br_2^+$ $[M]^+$ 433.9300, found 433.9319.

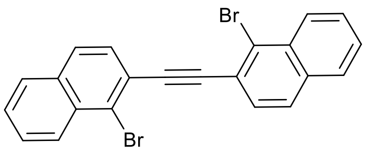

2b

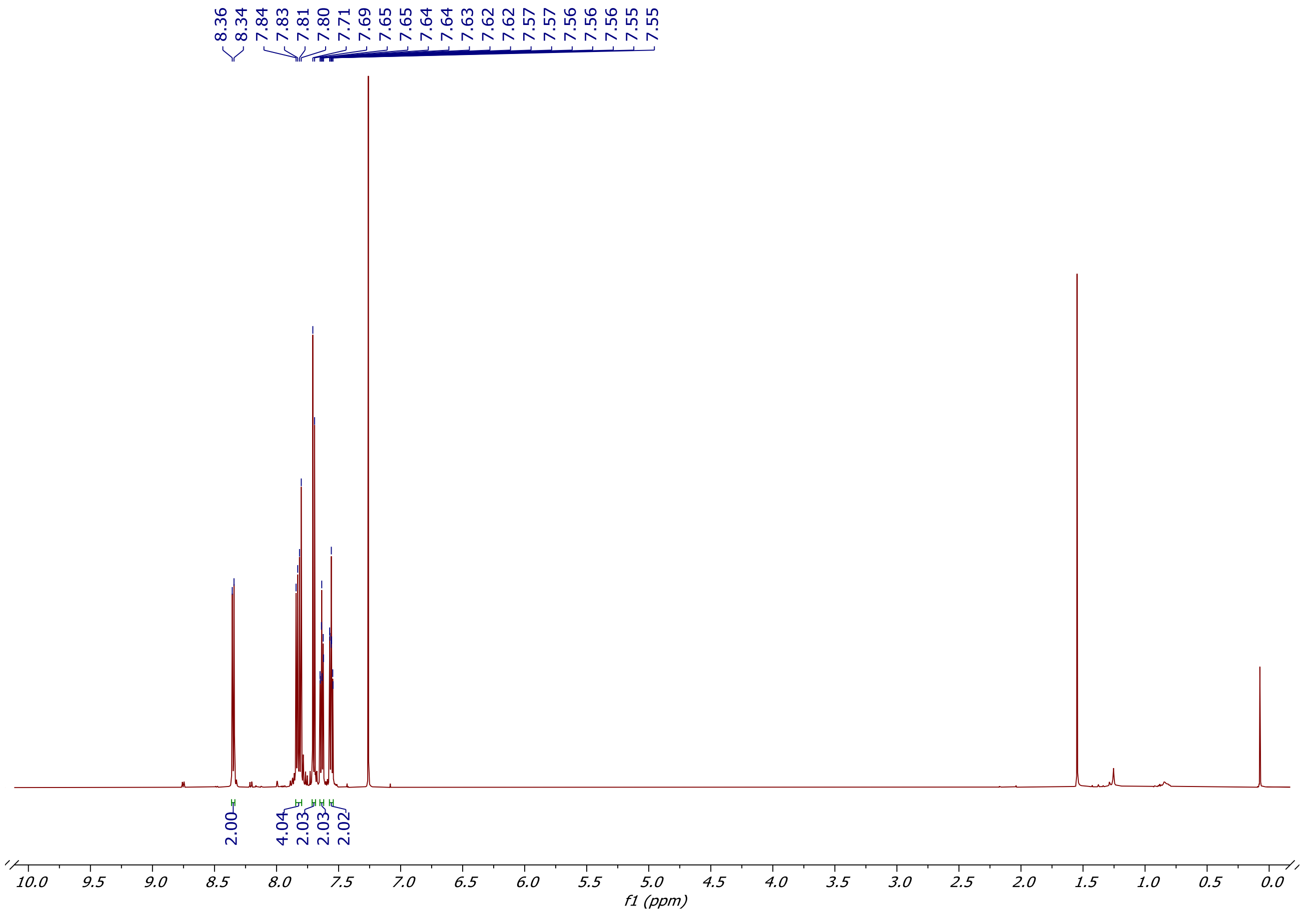

Figure S22. **^{1}H NMR** (600 MHz, $CDCl_3$, 298K) spectrum of **2b**.

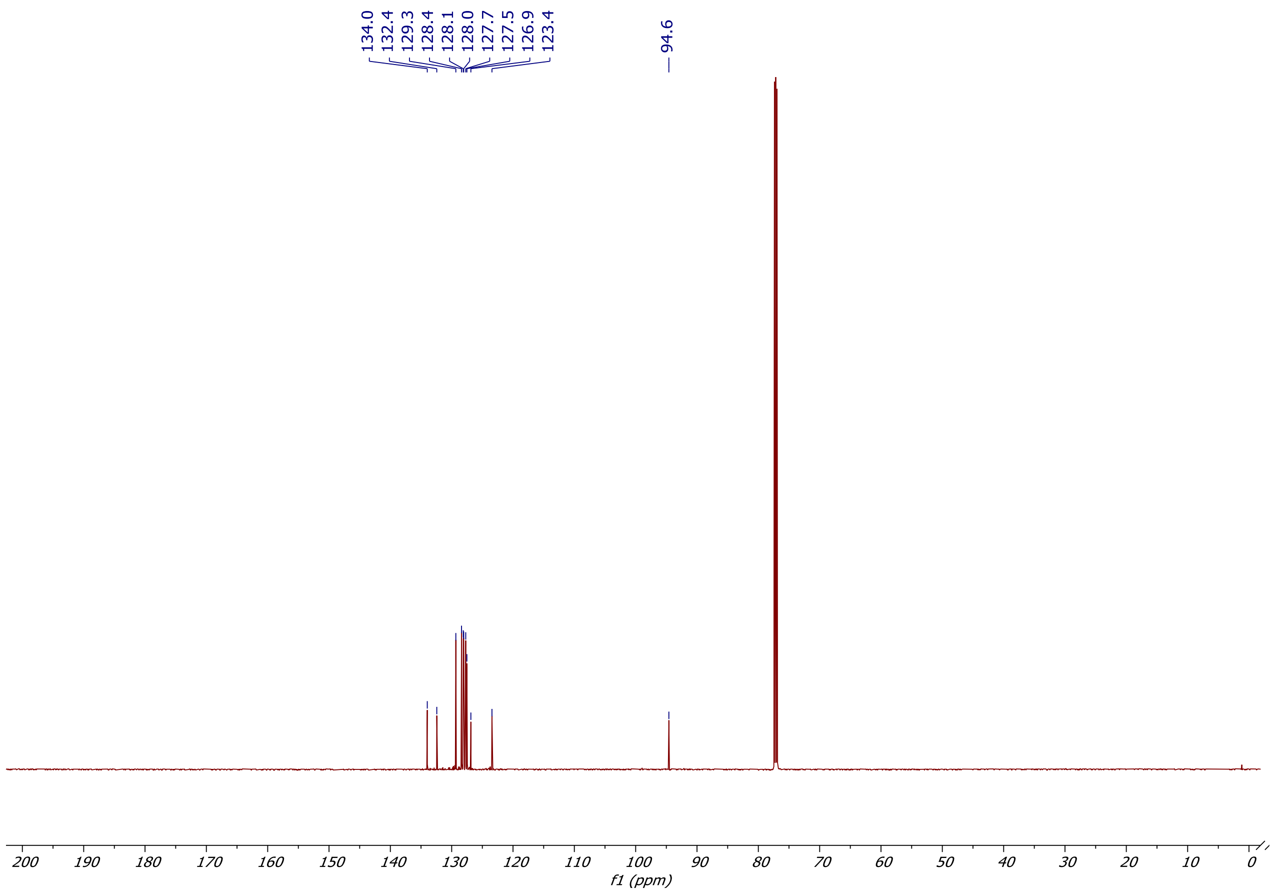

Figure S23. **^{13}C {^{1}H} NMR** (151 MHz, $CDCl_3$, 298K) spectrum of **2b**.

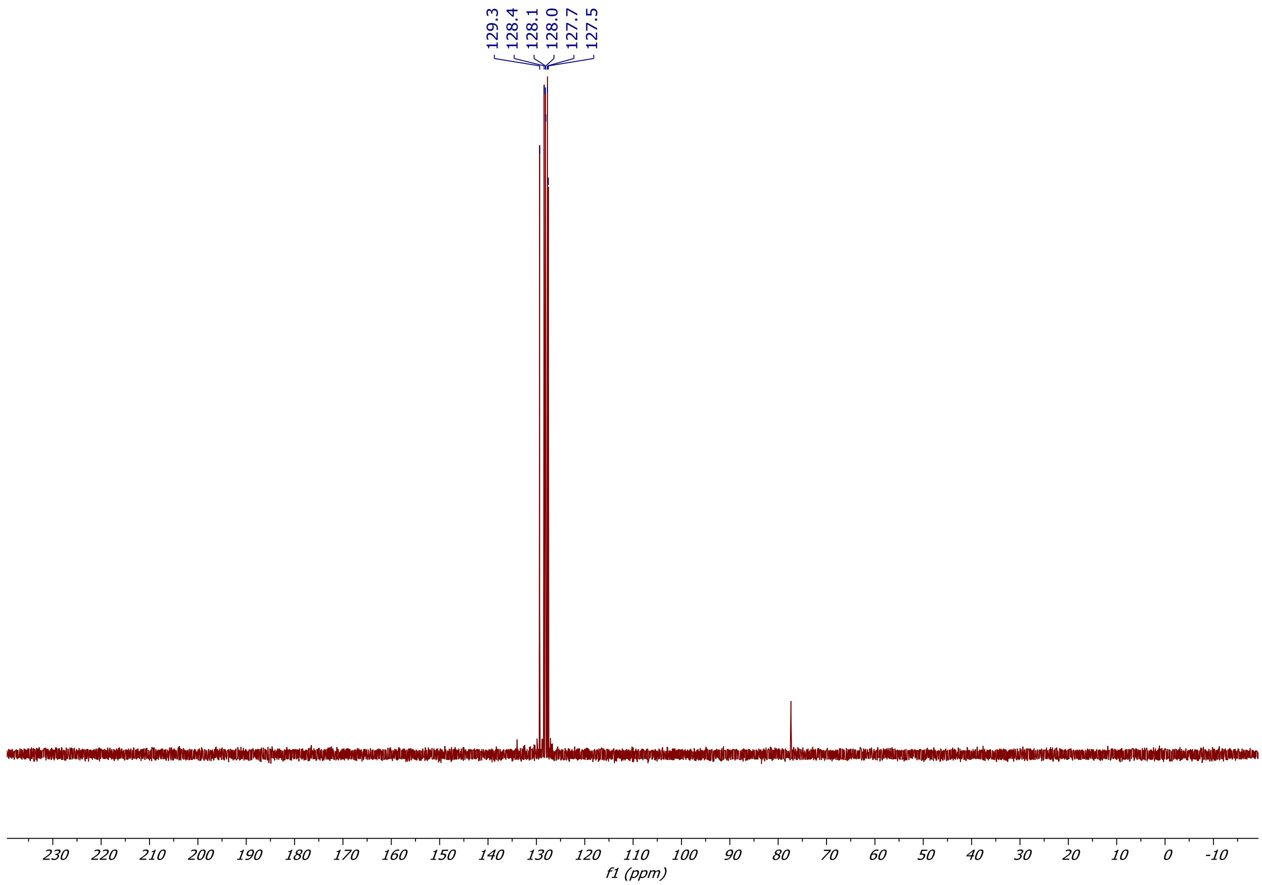

Figure S24. **^{13}C{^{1}H} DEPT-135 NMR** (151 MHz, $CDCl_3$, 298K) spectrum of **2b**.

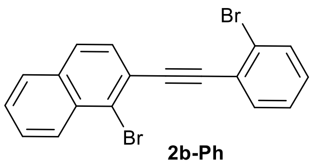

2b-Ph

1c (1.20 g, 3.60 mmol, 1.0 eq.), $Pd(PPh_3)_4$ (104 mg, 90.1 µmol, 2.5 mol%) and ((2-bromophenyl)ethynyl)trimethylstannane (1.30 g, 3.78 mmol, 1.1 eq.) were stirred in dry 1,4-dioxane (20 mL) at 100 °C for 18 h. After the mixture cooled to room temperature, the solvent was removed under reduced pressure. Purification of the crude product, performed by column chromatography (SiO_2, PE/EA, 10:1 + 1% Et_3N) afforded compound **2b-Ph** as an orange solid (819 mg, 2.12 mmol, 59%). **Mp:** 104 °C. **IR** $\tilde{\nu}$ [cm^{-1}] = 1591, 1548, 1494, 1477, 1453, 1433, 1322, 1245, 1044, 1026, 979, 940, 919, 862, 811, 767, 740, 653, 568, 527, 493, 445, 417 **^{1}H NMR** (400 MHz, $CDCl_3$): δ (in ppm) = 8.34–8.32 (m, 1H), 7.83–7.78 (m, 2H), 7.69–7.63 (m, 4H), 7.56–7.55 (m, 1H), 7.34 (td, J_{H-H} = 7.6, 1.2 Hz, 1H), 7.23–7.22 (m, 1H). **^{13}C{^{1}H} NMR** (101 MHz, $CDCl_3$): δ (in ppm) = 134.0 (C_q, 1C), 133.8 (CH, 1C), 132.7 (CH, 1C), 132.4 (C_q, 1C), 129.9 (CH, 1C), 129.3 (CH, 1C), 128.4 (CH, 1C), 128.1 (CH, 1C), 128.0 (CH, 1C), 127.7 (CH, 1C), 127.5 (CH, 1C), 127.2 (CH, 1C), 126.7 (C_q, 1C), 125.8 (C_q, 1C), 125.4 (C_q, 1C), 123.3 (C_q, 1C), 93.7 (C_q, 1C), 93.2 (C_q, 1C). **EI HR-MS** (pos): m/z calcd for $C_{18}H_{10}Br_2^+$ [M]$^+$ 383.9144, found 383.9163; calcd $C_{18}H_{10}^+$ [M-2Br]$^+$ 226.0777, found 226.0805.

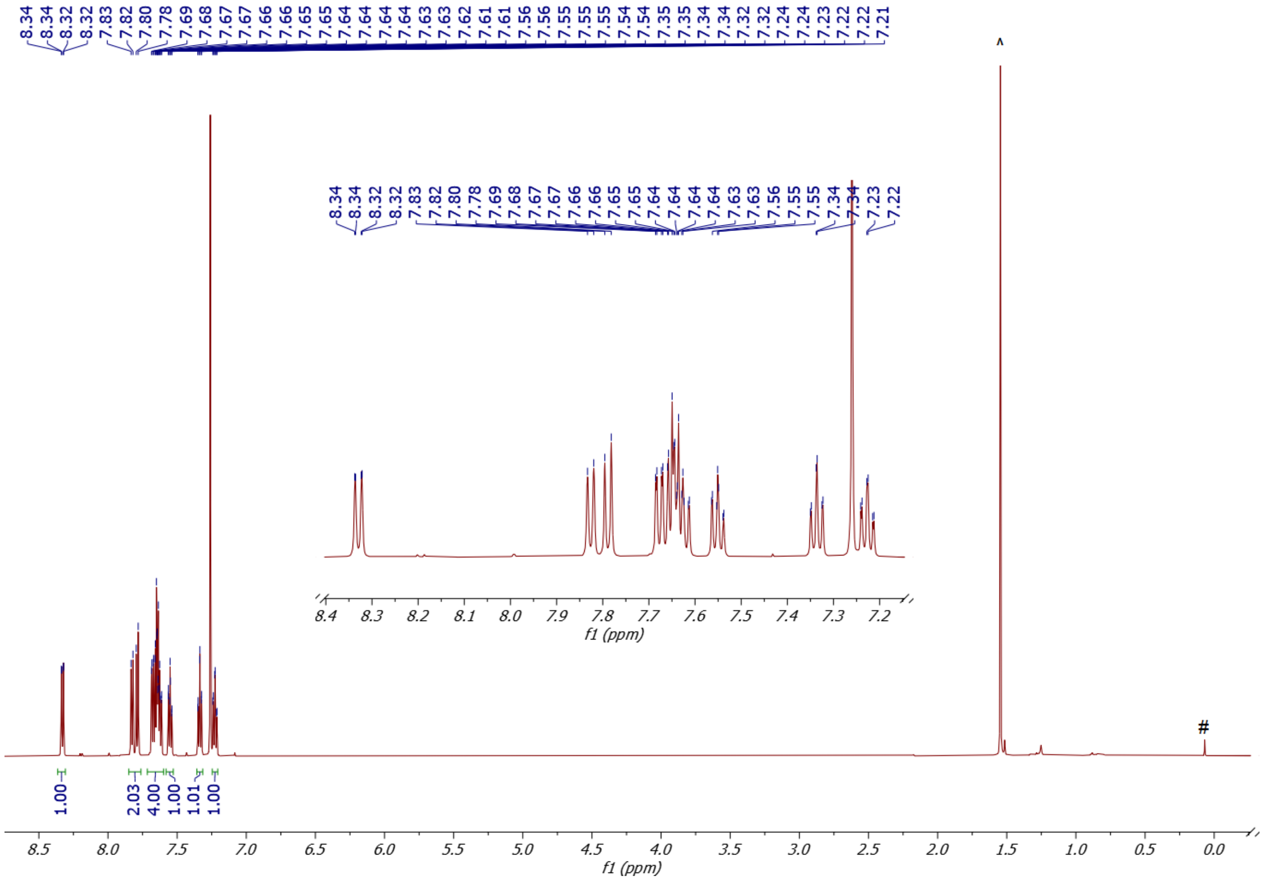

Figure S25. **^{1}H NMR** (400 MHz, $CDCl_3$, 298K) spectrum of **2b-Ph** (traces of impurities of water(^) and silicon grease(#) are marked).

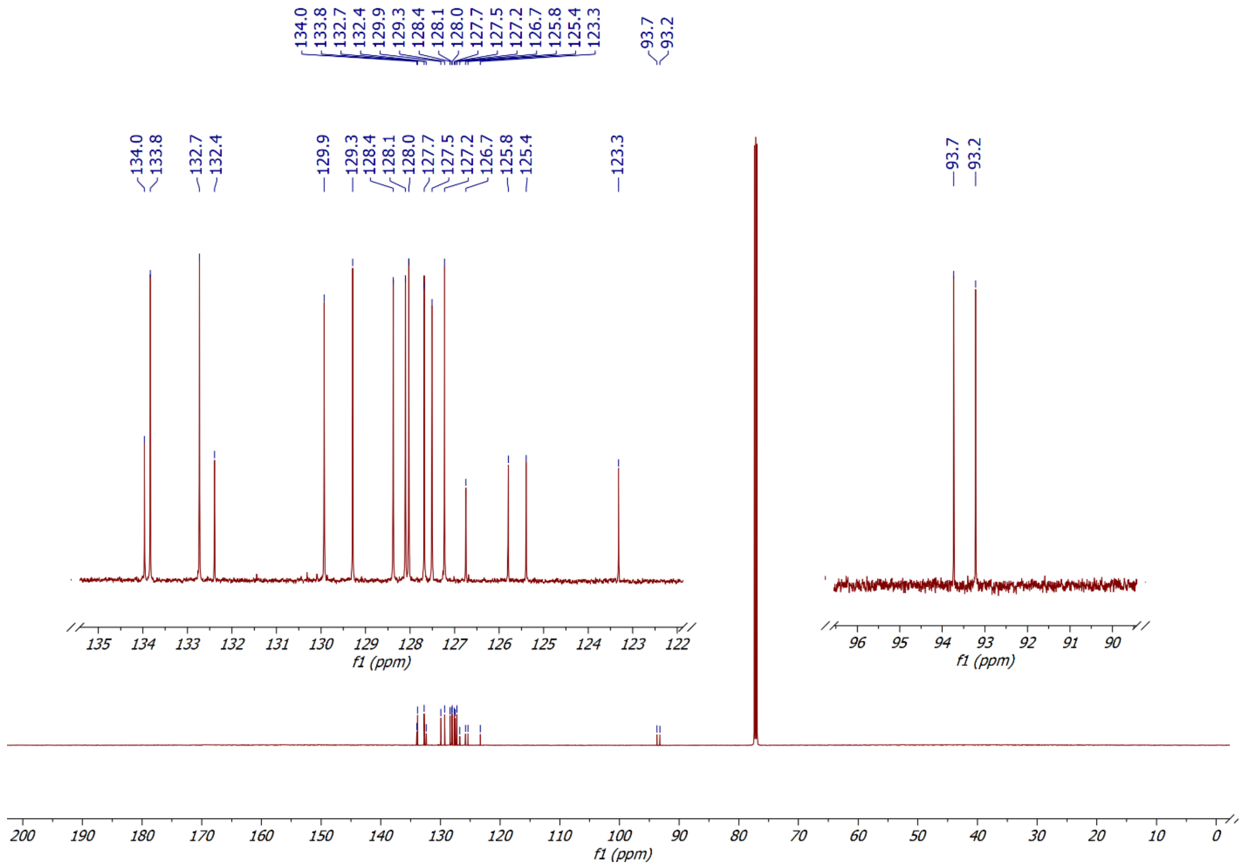

Figure S26. **$^{13}C\{^{1}H\}$ NMR** (101 MHz, $CDCl_3$, 298K) spectrum of **2b-Ph**.

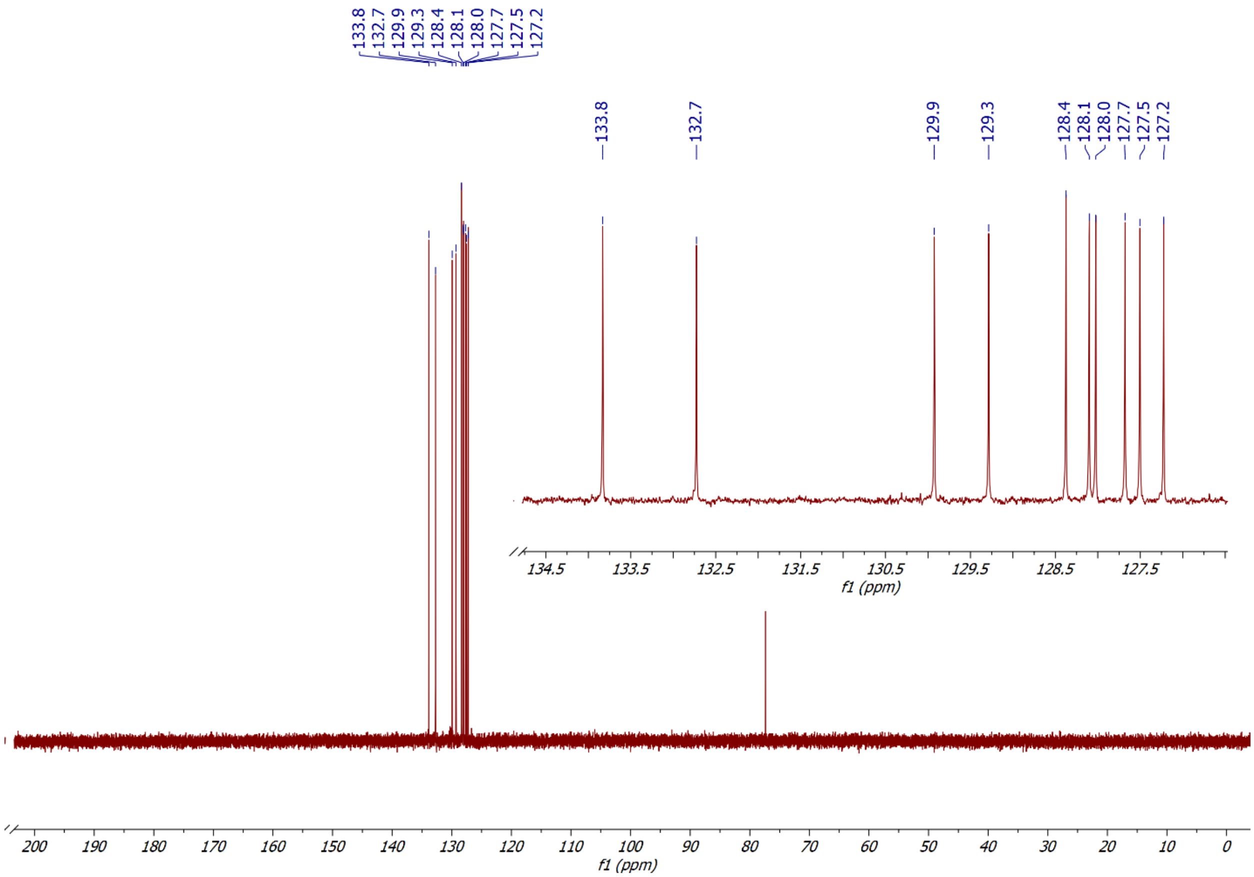

Figure S27. **$^{13}C\{^{1}H\}$ DEPT-135 NMR** (101 MHz, $CDCl_3$, 298K) spectrum of **2b-Ph**.

2b (1.50 g, 3.44 mmol, 1.0 eq.) was dissolved in a mixture of dry Et_2O and dry THF (10:1, 220 mL). A 2.5 M solution of *n*-BuLi in hexanes (2.89 ml, 7.22 mmol, 2.1 eq.) was added dropwise at –40 °C. After stirring the reaction mixture at –20 °C for 2 h, the solution was cooled to –40 °C and $^iPr_2PCl$ (1.10 g, 1.15 ml, 7.22 mmol, 2.1 eq.) was added dropwise. The mixture was stirred at –5 °C for 49 h using a cryostat. The solvent was removed under reduced pressure and the flask was brought into the glovebox. The dark green solid was dissolved in toluene and filtrated over silica. The solvent was removed under reduced pressure. The precipitate was washed with pentane and **3b** was obtained as a yellow solid (1.09 g, 2.13 mmol, 62%). **$^1H\{^{31}P\}$ NMR** (600 MHz, C_6D_6): δ (in ppm) = 9.72 (s, 2H), 7.80 (d, $J_{H\text{–}H}$ = 8.5 Hz, 2H), 7.56–7.52 (m, 4H), 7.41–7.36 (m, 2H), 7.24 (t, $J_{H\text{–}H}$ = 7.4 Hz, 2H), 3.44 (s, 4H), 1.39 (d, $J_{H\text{–}H}$ = 6.9 Hz, 12H), 0.98 (d, $J_{H\text{–}H}$ = 7.0 Hz, 12H). **$^{31}P\{^1H\}$ NMR** (162 MHz, C_6D_6): δ (in ppm) = 2.0 (s). **LIFDI** (pos): *m/z* calcd for $C_{34}H_{40}P_2^+$ $[M]^+$ 510.2600, found 510.2915. *Due to low thermal stability and large signal broadening, it was not possible to measure a reasonably resolved and pure $^{13}C\{^1H\}$ NMR spectrum*.

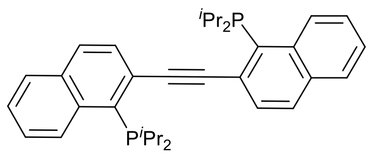

3b

2b-Ph (760 mg, 1.97 mmol, 1.0 eq.) was dissolved in a mixture of dry Et_2O and dry THF (10:1, 110 mL). A 2.5 M solution of *n*-BuLi in hexanes (1.65 ml, 4.13 mmol, 2.1 eq.) was added dropwise at –40 °C. After stirring the reaction mixture at –20 °C for 2 h, the solution was cooled to –40 °C and $^iPr_2PCl$ (658 µl, 4.13 mmol, 2.1 eq.) was added dropwise. The mixture was stirred at –5 °C for 97 h using a cryostat. The solvent was removed under reduced pressure and the flask was brought into the glovebox. The dark green solid was dissolved in toluene and filtrated over silica. The solvent was removed under reduced pressure. The precipitate was washed with pentane and **3b-Ph** was obtained as a green oil (719 mg, 1.56 mmol, 79%). **$^{31}P\{^1H\}$ NMR** (162 MHz, THF-d_8): δ (in ppm) = 3.2 (s), 1.9 (s). **LIFDI** (pos): *m/z* calcd for $C_{30}H_{38}P_2^+$ $[M]^+$ 460.2443, found 460.2919. *Due to low thermal stability and large signal broadening, it was not possible to measure a reasonably resolved and pure $^{13}C\{^1H\}$ NMR spectrum*.

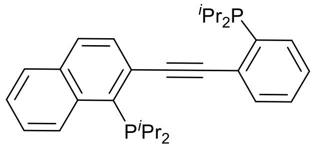

3b-Ph

3b (593 mg, 1.16 mmol, 1.0 eq.) and C_2Cl_6 (286 mg, 1.21 mmol, 1.0 eq.) were stirred in dry DCM (5 mL) at rt for 1.5 h. The solvent was removed under reduced pressure and the crude product was recrystallized from cold DCM. Compound **[*iso*-4b]$^{2+}$** was obtained as an off-white solid (434 mg, 0.75 mmol, 64%). **IR** $\tilde{\nu}$ [cm^{-1}] = 3424, 2963, 2924, 2802, 2532, 1615, 1569, 1507, 1459, 1446, 1390, 1370, 1345, 1267, 1248, 1211, 1133, 1035, 982, 877, 840, 823, 762, 725, 704, 671. **$^1H\{^{31}P\}$ NMR** (600 MHz, CD_2Cl_2): δ (in ppm) = 9.40 (d, $J_{H\text{–}H}$ = 8.7 Hz, 2H), 8.68 (d, $J_{H\text{–}H}$ = 8.7 Hz, 2H), 8.16 (d, $J_{H\text{–}H}$ = 7.7 Hz, 2H), 7.90–7.81 (m, 6H), 5.18 (hept, $J_{H\text{–}H}$ = 7.0 Hz, 4H), 1.81 (d, $J_{H\text{–}H}$ = 6.9 Hz, 12H), 1.30 (d, $J_{H\text{–}H}$ = 7.1 Hz, 12H). **$^{31}P\{^1H\}$ NMR** (162 MHz, CD_2Cl_2): δ (in ppm) = 97.5 (s). **$^{13}C\{^1H\}$ NMR** (151 MHz, CD_2Cl_2): δ (in ppm) = 150.1 (t, $J_{C\text{–}P}$ = 7.8 Hz, C_q, 2C), 142.1 (CH, 2C), 136.4 (t, $J_{C\text{–}P}$ = 4.8 Hz, C_q, 2C), 131.5 (CH, 4C), 131.0 (t, $J_{C\text{–}P}$ = 1.9 Hz, C_q, 2C), 130.8 (CH, 2C), 126.1 (CH, 2C), 126.0–125.6 (m, C_q, 2C), 124.7–124.2 (m, C_q, 2C), 120.9 (t, $J_{C\text{–}P}$ = 9.2 Hz, CH, 2C), 26.9 (t, $J_{C\text{–}P}$ = 12.2 Hz, CH, 4C), 19.0 (CH_3, 4C), 17.9 (CH_3, 4C). **LIFDI** (pos): *m/z* calcd for $C_{34}H_{40}ClP_2^+$ $[M\text{-}Cl]^+$ 545.2288, found 545.2523.

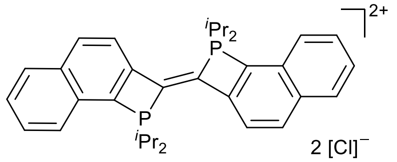

[*iso*-4b]$^{2+}$

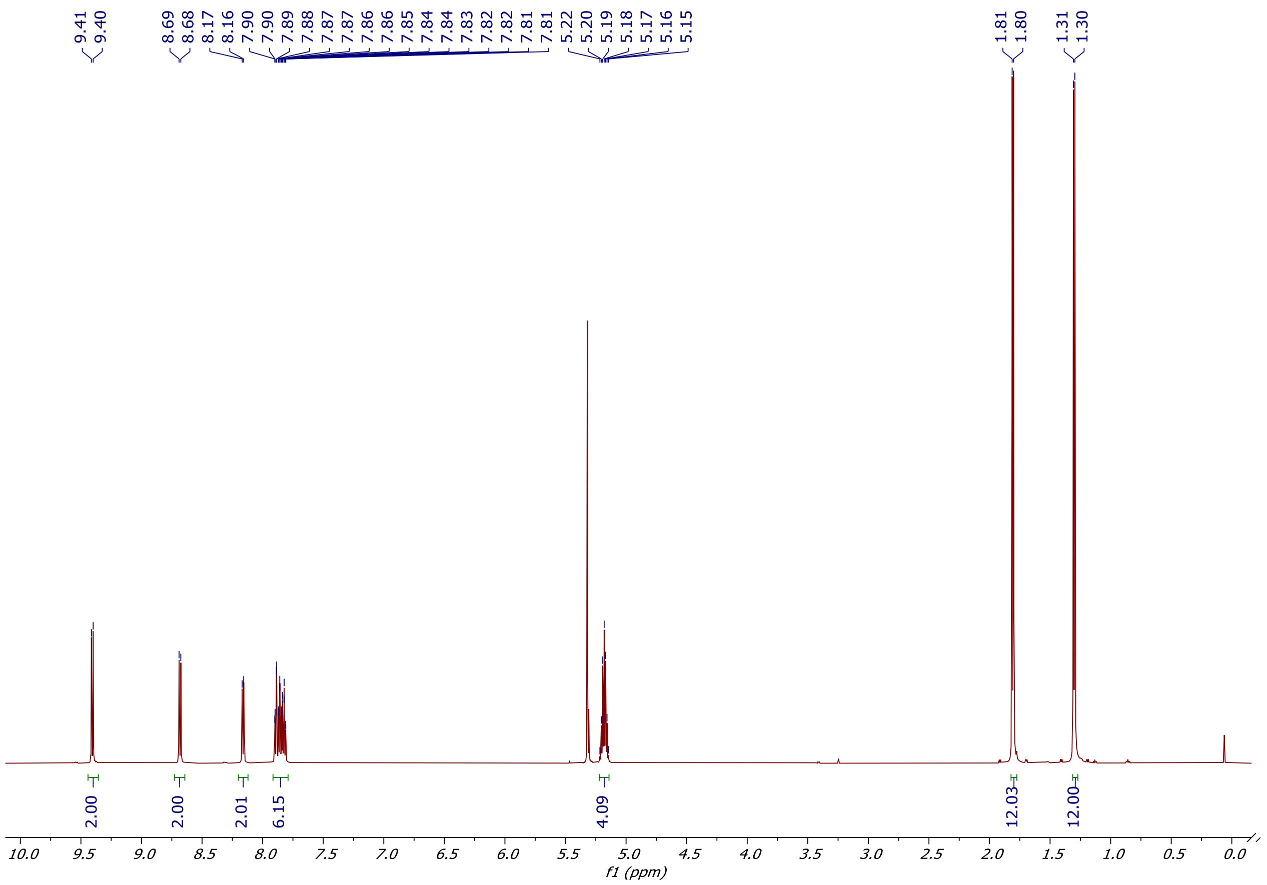

Figure S28. **^{1}H {^{31}P} NMR** (600 MHz, CD_2Cl_2, 298K) spectrum of **[*iso*-4b]$^{2+}$**.

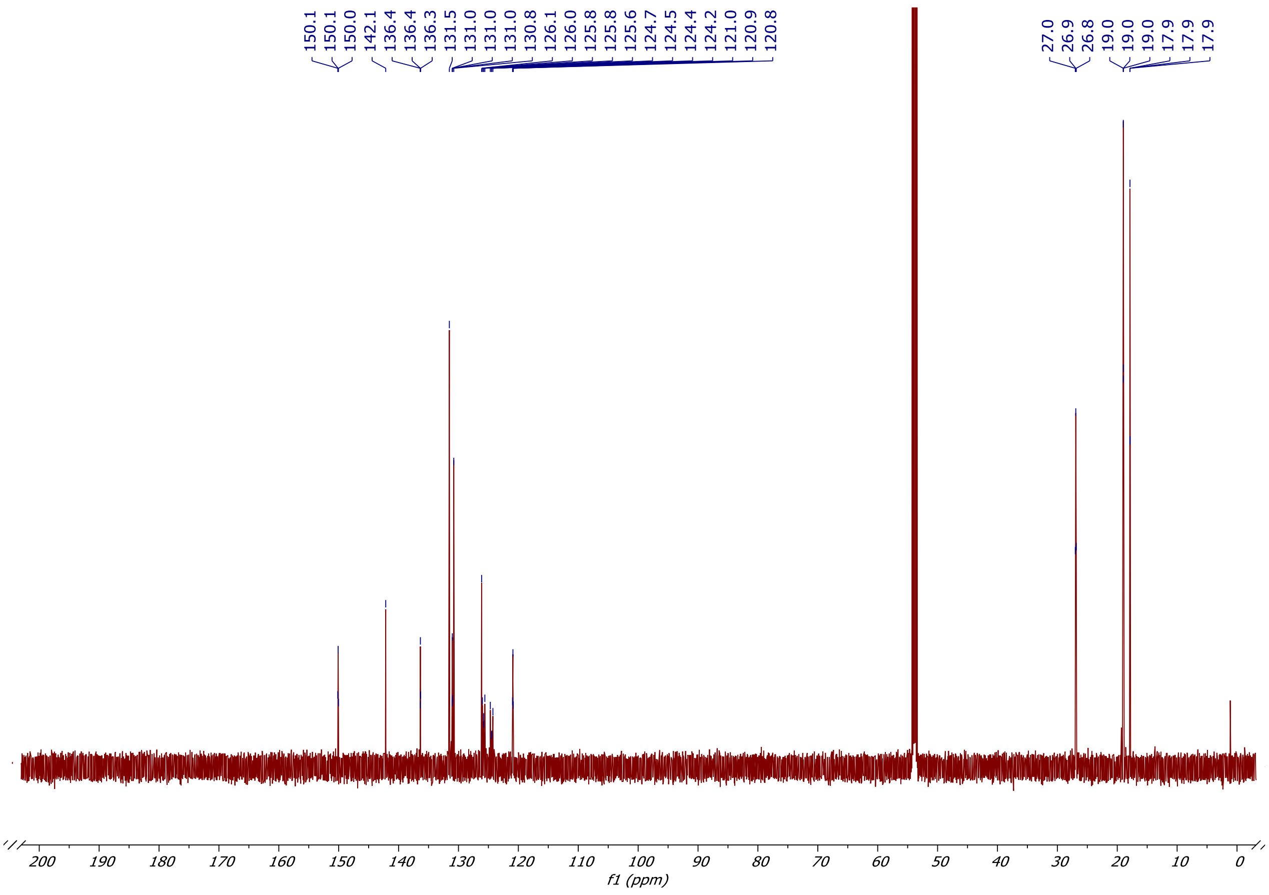

Figure S29. **^{13}C{^{1}H} NMR** (151 MHz, CD_2Cl_2, 298K) spectrum of **[*iso*-4b]$^{2+}$**.

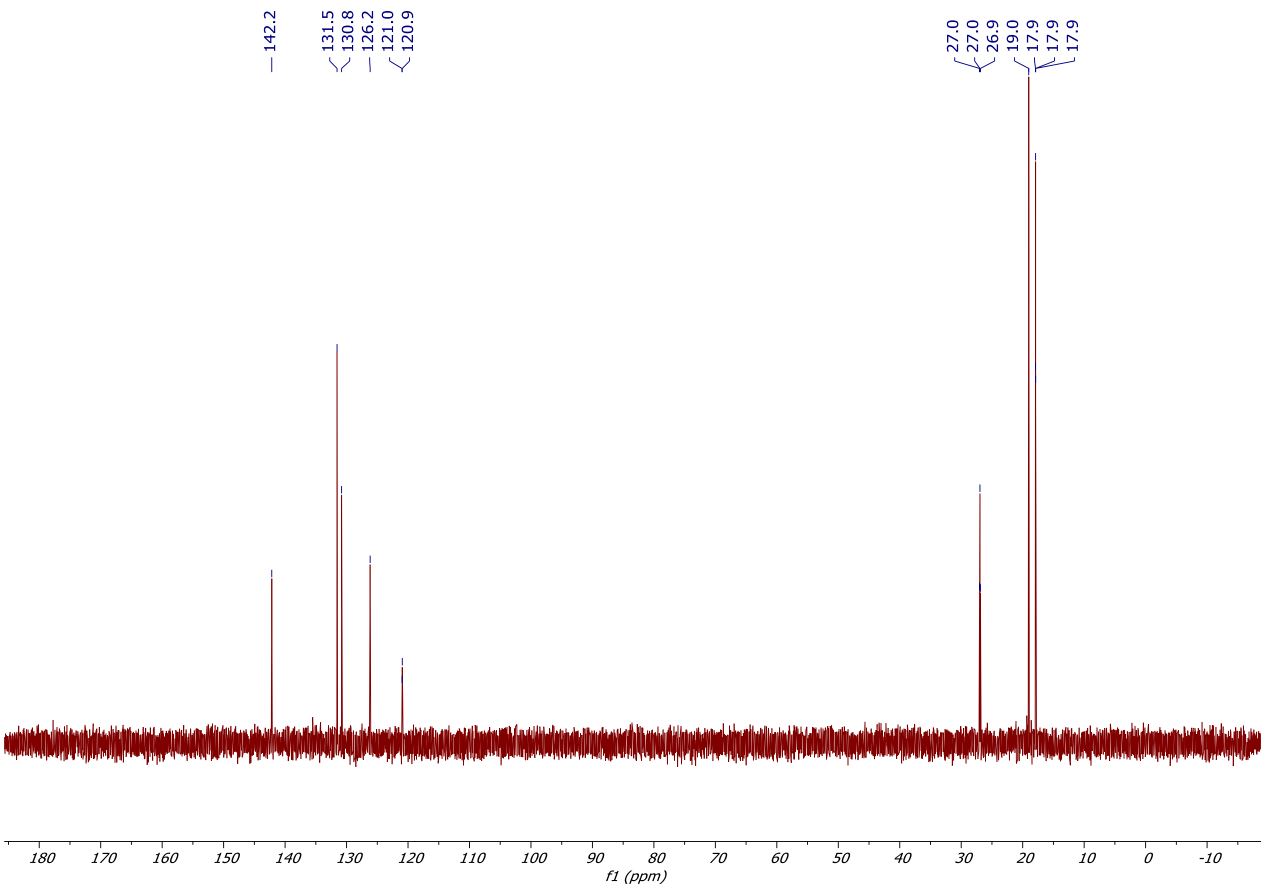

Figure S30. **^{13}C{^{1}H} DEPT-135 NMR** (151 MHz, CD_2Cl_2, 298K) spectrum of **[*iso*-4b]$^{2+}$**.

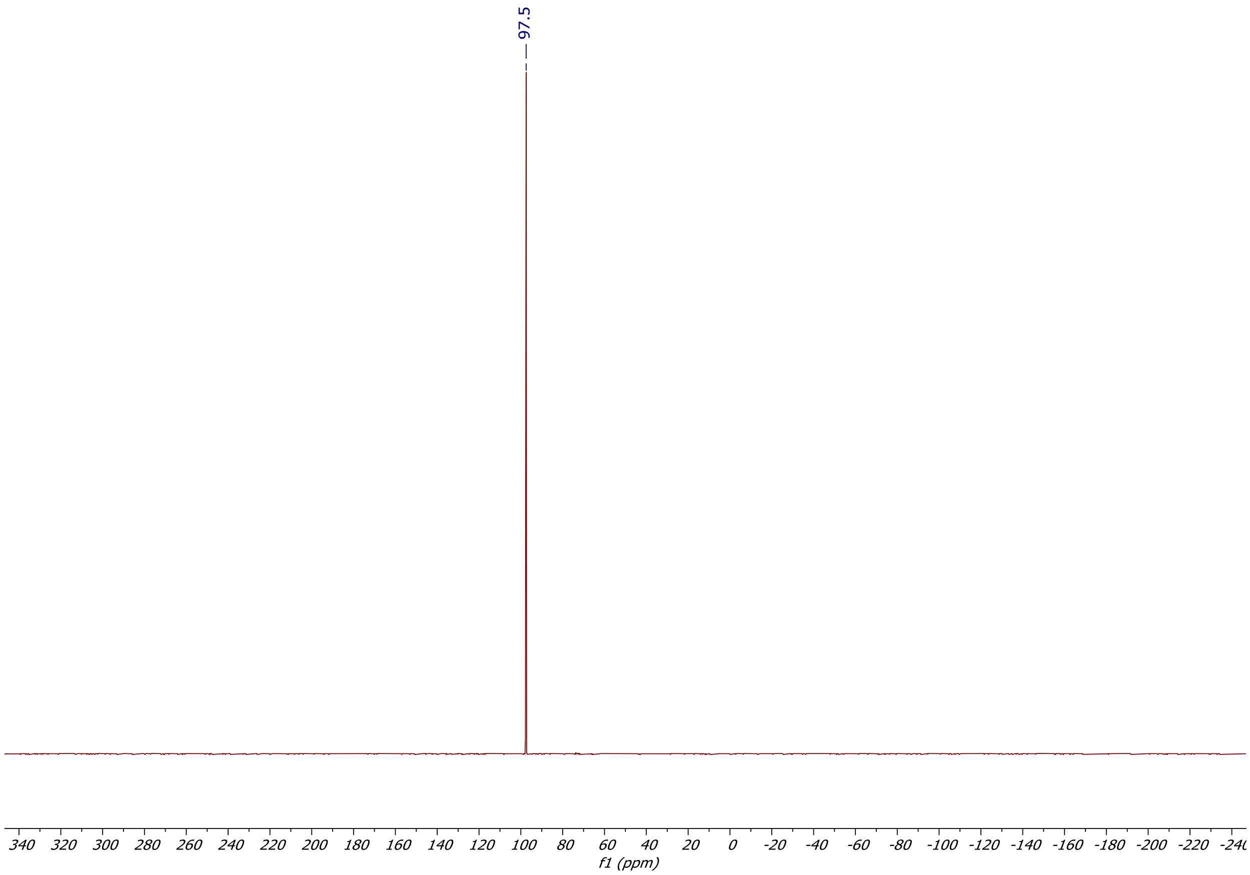

Figure S31. **^{31}P{^{1}H} NMR** (162 MHz, CD_2Cl_2, 298K) spectrum of **[*iso*-4b]$^{2+}$**.

3b-Ph (400 mg, 868 μmol, 1.0 eq.) and C_2Cl_6 (214 mg, 903 μmol, 1.0 eq.) were stirred in dry DCM (5 mL) at rt for 3 h. The precipitate was collected by filtration and washed with a mixture of DCM and NEt_3 (10 ml DCM and three drops NEt_3). After washing with additional DCM and drying under high vacuum, **[4b-Ph]$^{2+}$** was obtained as a yellow powder (98.0 mg, 184 μmol, 22%). **Mp:** 242 °C (decomp.) **IR** $\tilde{\upsilon}$ [cm^{-1}] = 3387, 3044, 2965, 2847, 2349, 2317, 1613, 1512, 1453, 1372, 1257, 1135, 1038, 988, 878, 845, 813, 783, 760, 696, 670, 665, 626, 568, 497, 428 **^{1}H NMR {^{31}P}** (400 MHz, CD_3OD): δ (in ppm) = 8.73 (d, $J_{H\text{–}H}$ = 8.4 Hz, 1H), 8.43 (d, $J_{H\text{–}H}$ = 7.5 Hz, 1H), 8.33 (d, $J_{H\text{–}H}$ = 8.3 Hz, 1H), 8.25–8.16 (m, 4H), 8.04–7.99 (m, 2H), 7.93–7.89 (m, 1H), 4.22–4.11 (m, 2H), 4.05–3.94 (m, 2H), 1.54 (d, $J_{H\text{–}H}$ = 7.0 Hz, 12H), 1.43 (d, $J_{H\text{–}H}$ = 7.0 Hz, 12H). **^{31}P NMR {^{1}H}** (162 MHz, CD_3OD): δ (in ppm) = 66.3 (d, $J_{P\text{–}P}$ = 26.1 Hz, 1P), 58.3 (d, $J_{P\text{–}P}$ = 26.2 Hz, 1P). **^{13}C NMR {^{1}H, ^{31}P}** (151 MHz, CD_3OD): δ (in ppm) = 148.6 (C_q, 1C), 148.5 (C_q, 1C), 141.0 (C_q, 1C), 140.6 (CH, 1C), 140.3 (C_q, 1C), 138.6 (CH, 1C), 136.3 (C_q, 1C), 136.0 (CH, 1C), 134.4 (C_q, 1C), 134.1 (CH, 1C), 133.1 (CH, 1C), 131.9 (CH, 1C), 131.1 (CH, 1C), 129.3 (CH, 1C), 126.4 (CH, 1C), 123.9 (CH, 1C), 121.6 (C_q, 1C), 119.3 (C_q, 1C), 25.1 (CH, 2C), 24.2 (CH, 2C), 17.0 (CH_3, 2C), 16.8 (CH_3, 2C), 16.7 (CH_3, 2C), 16.1 (CH_3, 2C). **ESI HR-MS** (pos): *m/z* calcd for $C_{30}H_{39}OP_2^+$ [M+OH]$^+$ 477.2471, found 477.2470.

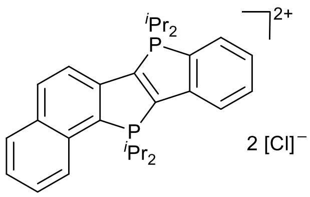

[4b-Ph]$^{2+}$

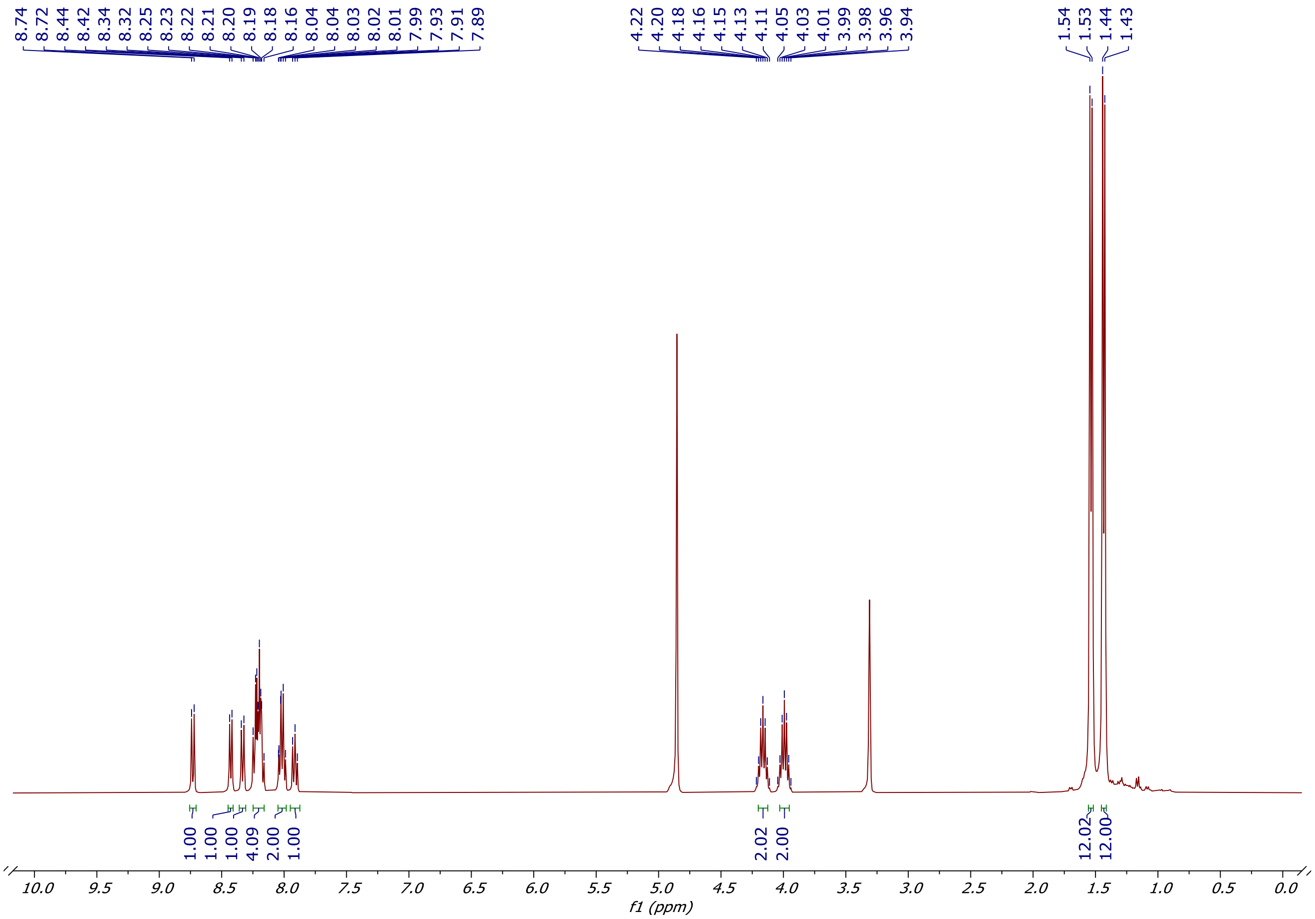

Figure S32. **^{1}H NMR {^{31}P}** (400 MHz, CD_3OD, 298K) spectrum of **[4b-Ph]$^{2+}$**.

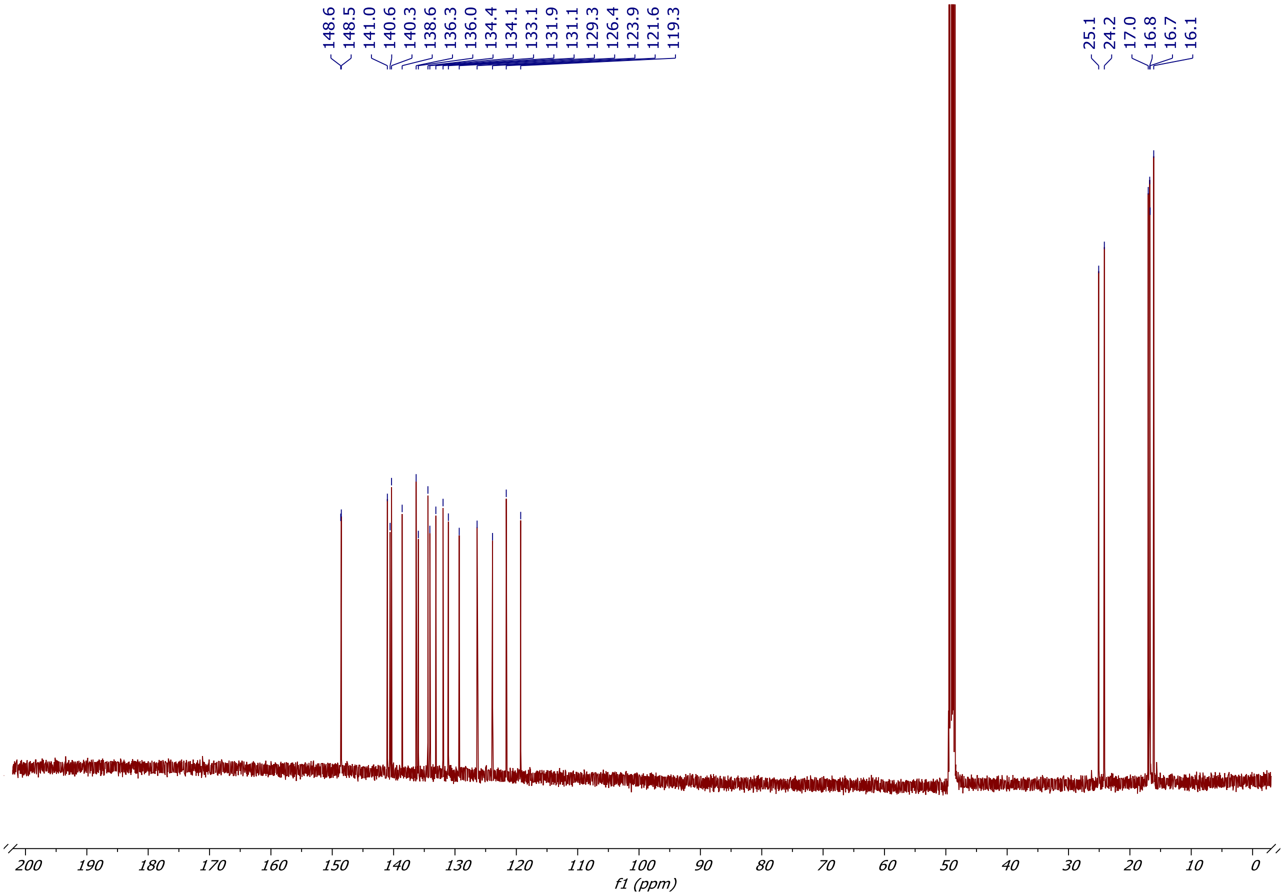

Figure S33. **^{13}C NMR {^{1}H, ^{31}P}** (151 MHz, CD_3OD, 298K) spectrum of **[4b-Ph]$^{2+}$**.

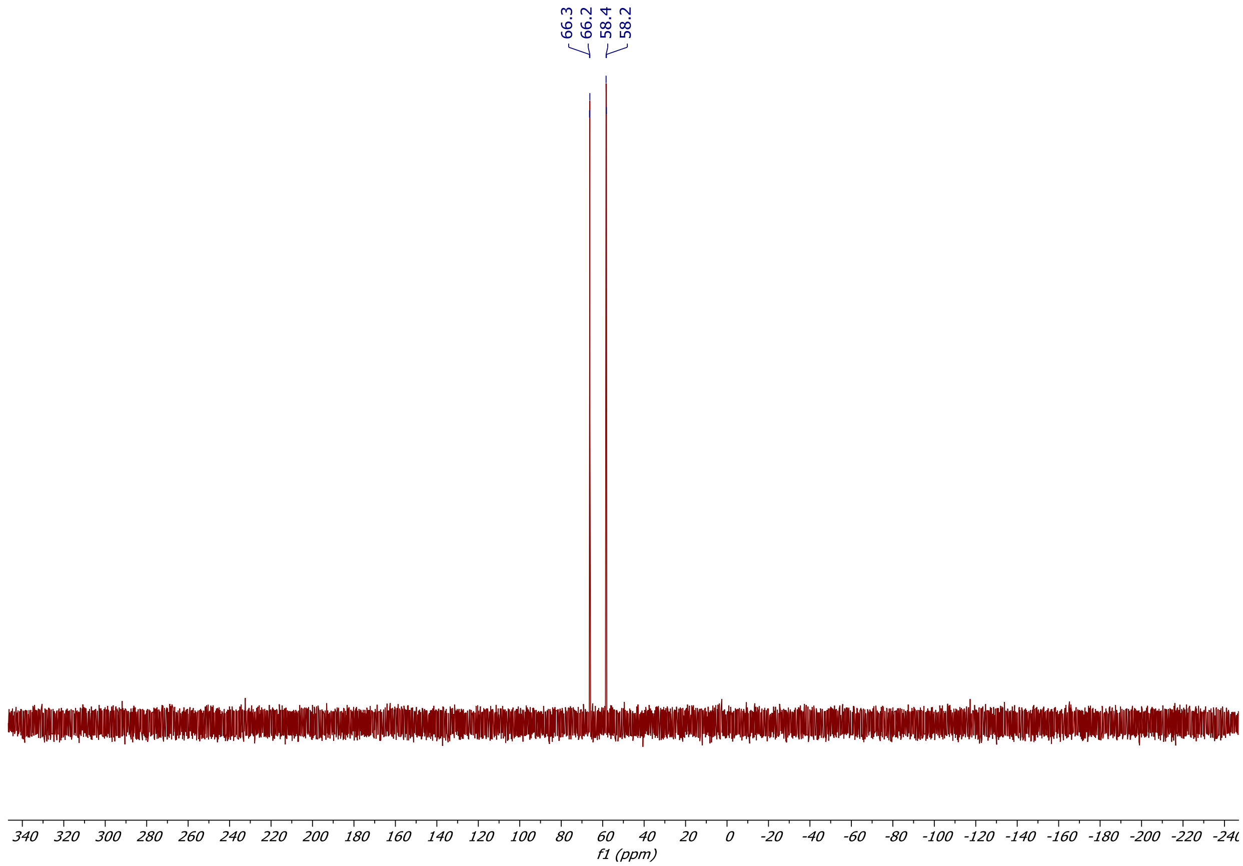

Figure S34. **^{31}P NMR {^{1}H}** (162 MHz, CD_3OD, 298K) spectrum of **[4b-Ph]$^{2+}$**.

3b (200 mg, 392 μmol) was stirred in dry toluene at 100 °C for 20 h. The solvent was removed under reduced pressure and the crude product was washed with Et_2O. **[4b]^0** was obtained as dark green solid (110 mg, 55%). **IR** $\tilde{\upsilon}$ [cm^{-1}] = 3385, 2956, 2926, 2861, 2842, 1600, 1582, 1509, 1445, 1358, 1296, 1257, 1128, 1095, 1022, 980, 931, 878, 814, 762, 739, 682. **^{1}H NMR** (600 MHz, C_6D_6): δ (in ppm) = 7.38–7.37 (m, 2H), 7.22–7.19 (m, 2H), 7.05–7.02 (m, 4H), 6.91 (s, 2H), 6.86 (s, 2H), 2.51 (s, 4H), 1.03–0.96 (m, 24H) **^{31}P{^{1}H} NMR** (243 MHz, C_6D_6): δ (in ppm) = 32.3 (s). *Due to low thermal stability and large signal broadening, it was not possible to measure a reasonably resolved and pure ^{13}C{^{1}H} NMR spectrum*.

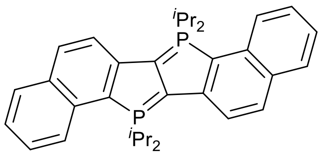

[4b]^0

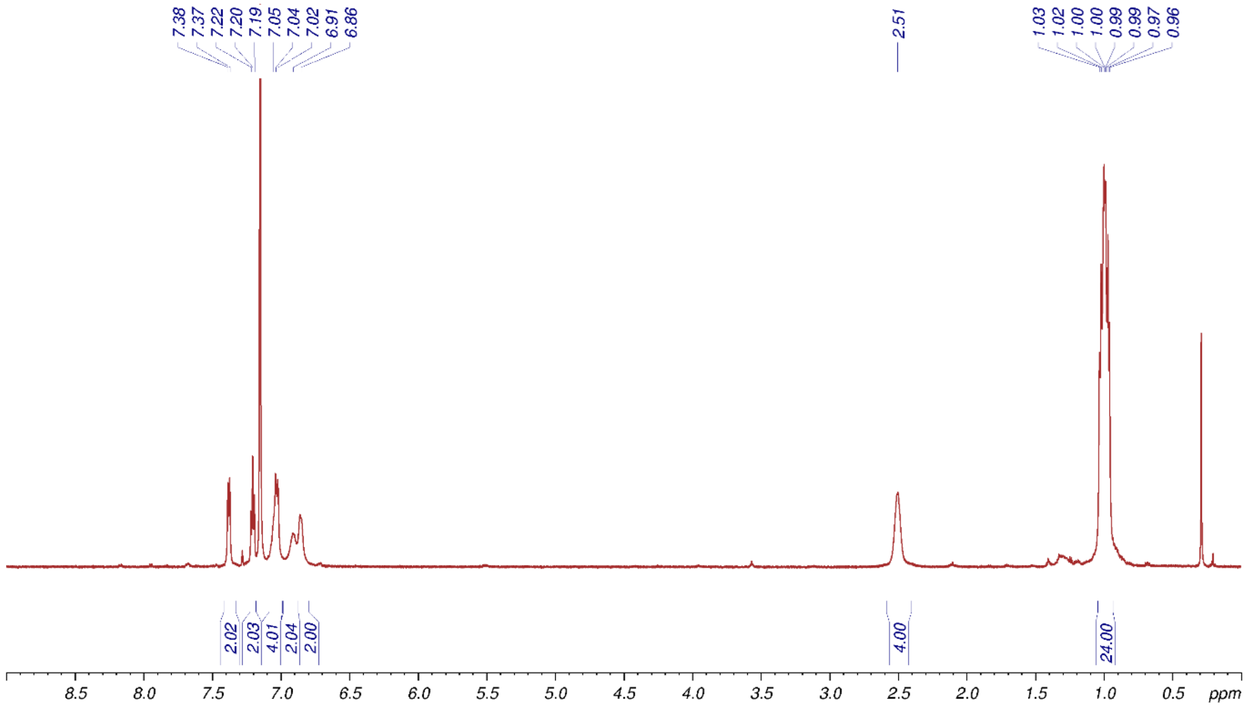

Figure S35. **^{1}H NMR** (600 MHz, C_6D_6, 298K) spectrum of **[4b]^0**.

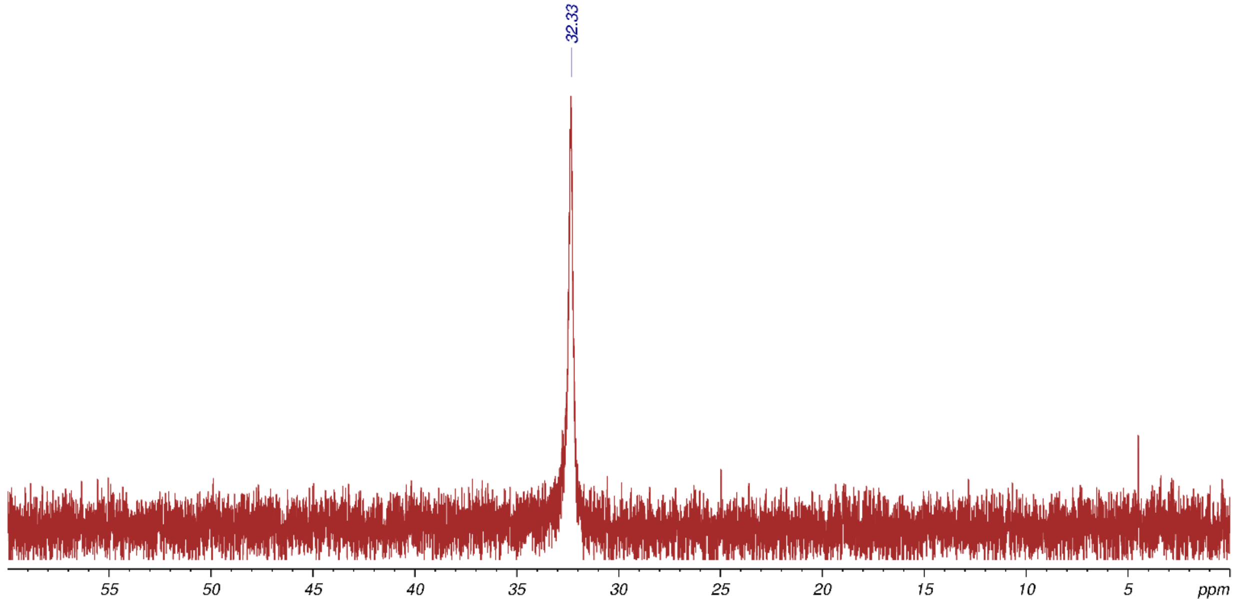

Figure S36. **^{31}P{^{1}H} NMR** (243 MHz, C_6D_6, 298K) spectrum of **[4b]^0**.

1.5) General procedure for the oxidation of 3a-Ph, 3a, 3b-Ph or 3b with Fc[PF_6]

In an argon-filled glovebox, 1.0 eq. of the diphosphine (**3a-Ph**, **3a**, **3b-Ph** or **3b**) was dissolved in PhF (0.5 mL) or in MeCN (0.5 mL) in an NMR tube. Solid Fc[PF_6] (2.0 eq) was added and the mixture was shaken on an orbital stirrer at room temperature overnight. The solvent was then decanted, and the resulting solid was dissolved in MeCN-d_3 (0.5 mL). The ^1H and $^{13}C\{^1H\}$ NMR spectra of the thus obtained bis-(hexafluorophosphate) salts were indistinguishable from the spectra of the respective bis-chloride salts, which were obtained via oxidation with C_2Cl_6 (*vide supra*). The $^{31}P\{^1H\}$ spectra confirmed the presence of [**4a**]$^{2+}$, [**4a-Ph**]$^{2+}$, [**4b-Ph**]$^{2+}$ and [***iso*-4b**]$^{2+}$, and clearly indicated that two [PF_6]$^-$ anions (per dication) were present with δ ($^{31}P\{^1H\}$) for [PF_6]$^-$ = −145 ± 1 ppm.

1.6) Synthesis of [4b]$^{2+}$

Method A: Oxidation of [4b]^0

[4b]^0 (526 mg, 1.03 mmol, 1.0 eq.) and C_2Cl_6 (254 mg, 1.07 mmol, 1.0 eq.) were stirred in dry DCM (7 mL) at rt for 22 h inside the glovebox. The crude product was collected by filtration washed with DCM. Recrystallization from MeOH/ Et_2O afforded ***[4b]$^{2+}$***as bright orange solid (370 mg, 636 μmol, 62%).

Method B: Photochemical Rearrangement of [iso-4b]$^{2+}$to [4b]$^{2+}$

[iso-4b]$^{2+}$ (200.2 mg, 0.34 mmol, 1.0 eq.) was dissoved in dry DCM (2 mL). The mixture was irradiated at 390 nm for 24h (40 W, Kessil LED) in a schlenk ampoule. The solvent was removed under reduced pressure and the residue was dissolved in 1 mL of MeOH. **[4b]$^{2+}$** was isolated via slow diffusion crystallization from MeOH/Et_2O and obtained as a bright orange solid (40.6 mg, 69.9 μmol, 20%).

Method C: Reduction of [iso-4b]$^{2+}$to [3b]

[iso-4b]$^{2+}$ (142 mg, 0.24 mmol, 1.0 eq.) was suspended in dry Et_2O (5 mL) inside an argon-filled glovebox. Solid KC_8 (79.2 mg, 0.59 mmol, 2.4 eq.) was added, and the mixture was stirred for 18 h, then filtered over Celite. The solvent was removed in vacuum, and the residue was washed with pentane to obtain **[3b]** as a colorless solid (66.3 mg, 0.13 mmol, 53%).

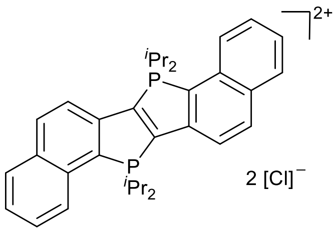

[4b]$^{2+}$

Mp: 260 °C (decomp.) **IR** $\tilde{\nu}$ [cm^{-1}] = 3397, 2960, 2838, 2349, 2337, 2312, 2031, 1577, 1508, 1457, 1371, 1304, 1256, 1143, 1038, 980, 878, 832, 800, 782, 765, 730, 694, 670, 665, 632, 566, 484, 431. **$^1H\{^{31}P\}$ NMR** (400 MHz, D_2O): δ (in ppm) = 8.63 (d, $J_{H\text{–}H}$ = 8.5 Hz, 2H), 8.27 (d, $J_{H\text{–}H}$ = 8.3 Hz, 2H), 8.10 (d, $J_{H\text{–}H}$ = 8.7 Hz, 4H), 7.96 (t, $J_{H\text{–}H}$ = 7.7 Hz, 2H), 7.86 (t, $J_{H\text{–}H}$ = 7.6 Hz, 2H), 4.06–3.99 (m, 4H), 1.55–1.42 (m, 24H) **$^{31}P\{^1H\}$ NMR** (162 MHz, D_2O): δ (in ppm) = 65.2 (s) **$^{13}C\{^1H, ^{31}P\}$ NMR** (151 MHz, D_2O): δ (in ppm) = 147.6 (C_q, 2C), 140.0 (C_q, 2C), 138.9 (CH, 2C), 134.8 (C_q, 2C), 133.2 (C_q, 2C), 131.6 (CH, 2C), 130.7 (CH, 2C), 129.8 (CH, 2C), 125.3 (CH, 2C), 123.0 (CH, 2C), 118.2 (C_q, 2C), 24.1 (CH, 4C), 16.2 (CH_3, 4C), 15.8 (CH_3, 4C). **ESI HR-MS** (pos): *m/z* calcd for $C_{34}H_{39}P_2$ [M-2Cl-H]$^+$ 509.2522, found 509.2530; calcd for $C_{34}H_{41}P_2$ [M-2Cl+H]$^+$ 511.2678, found 511.2686.

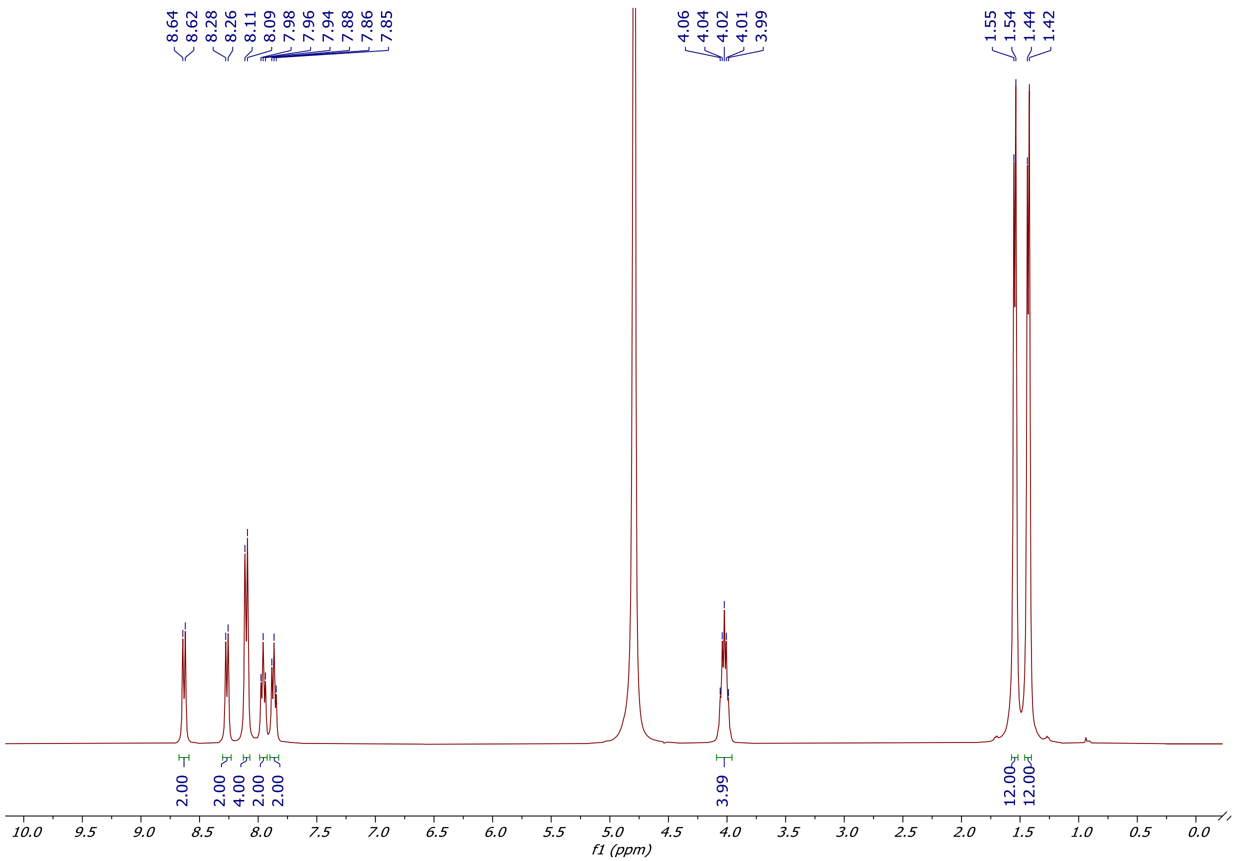

Figure S37. **^{1}H{^{31}P} NMR** (400 MHz, D_2O, 298K) spectrum of **$[4b]^{2+}$**.

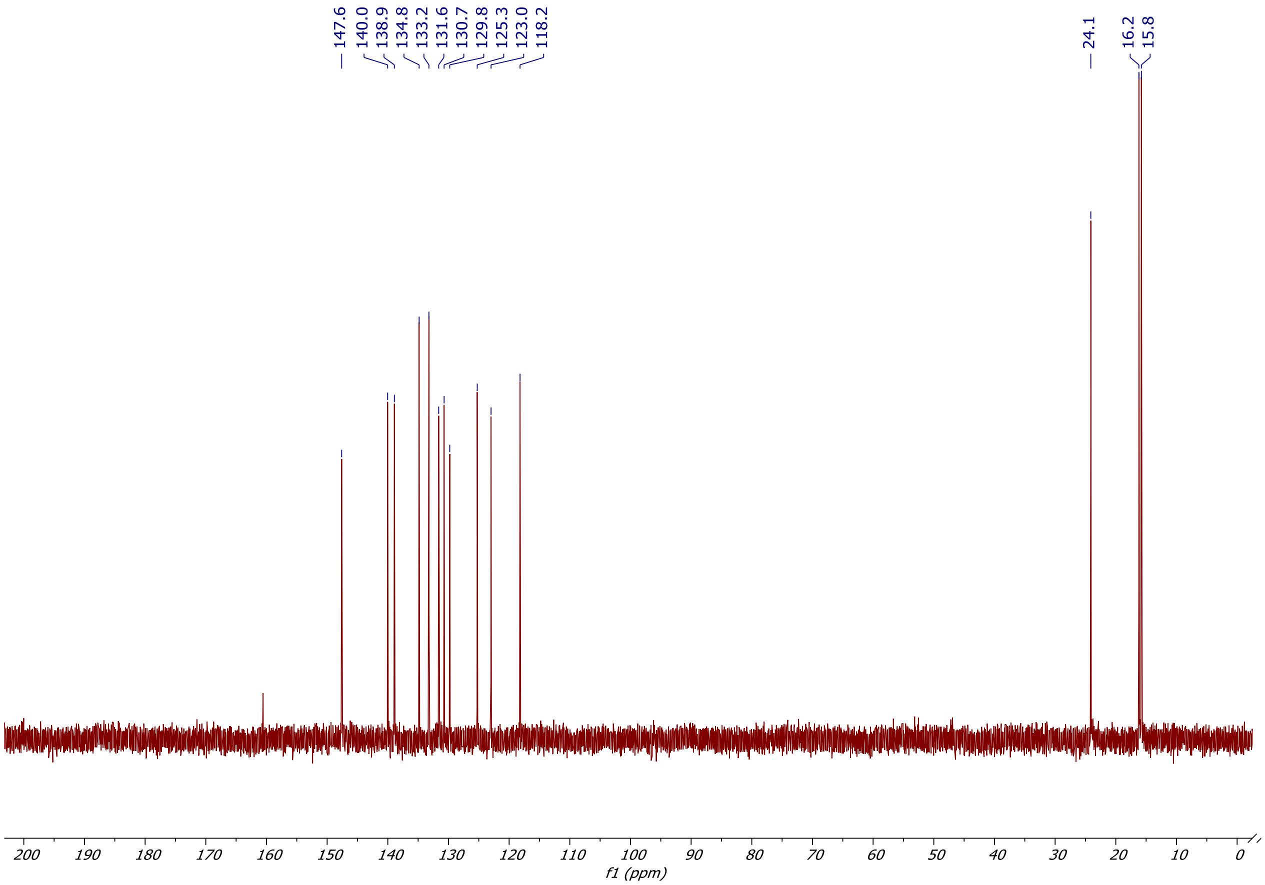

Figure S38. **^{13}C{^{1}H, ^{31}P} NMR** (151 MHz, D_2O) spectrum of **$[4b]^{2+}$**.

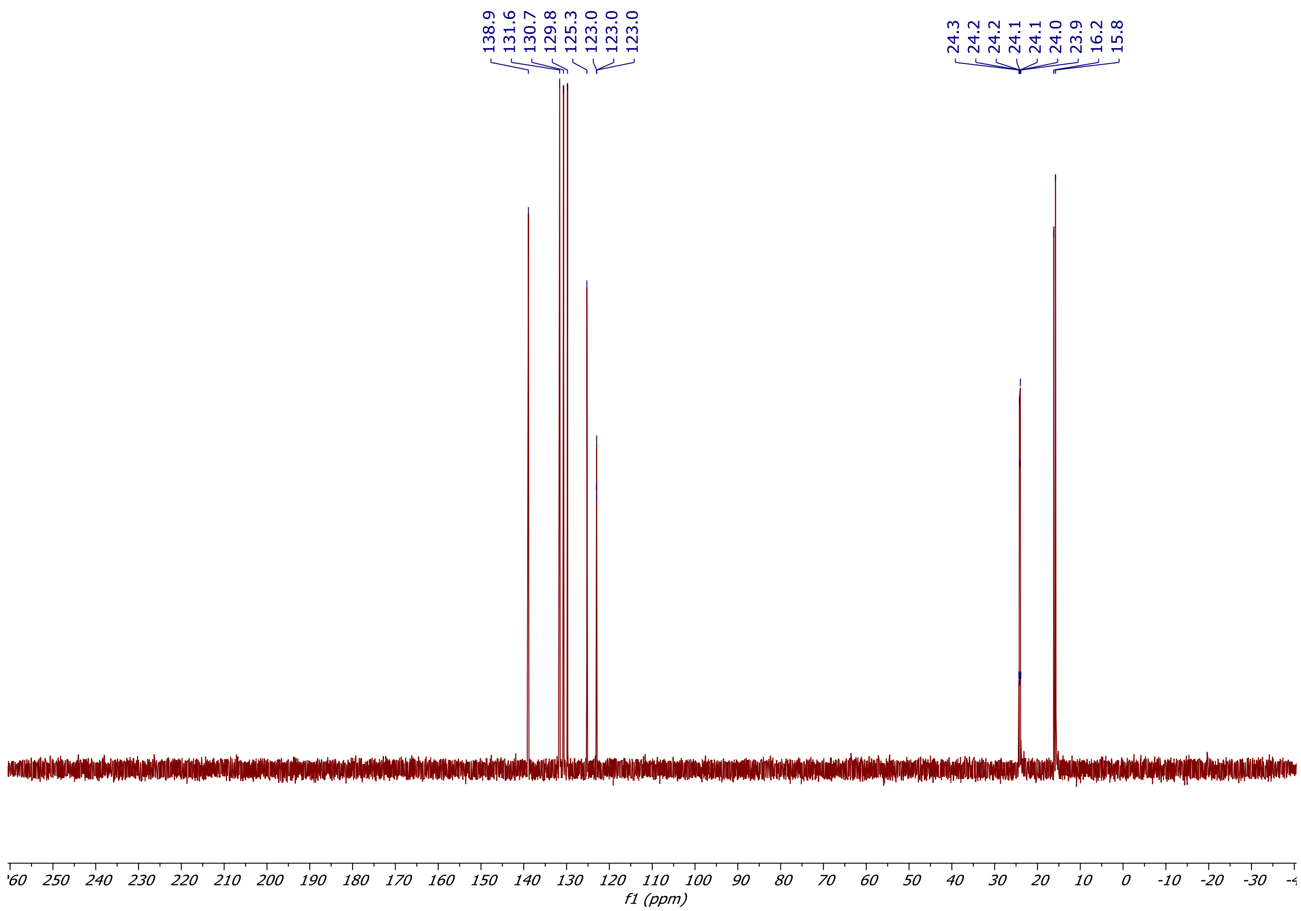

Figure S39. **$^{13}C\{^{1}H, ^{31}P\}$ DEPT-135 NMR** (151 MHz, D_2O, 298K) spectrum of **$[4b]^{2+}$**.

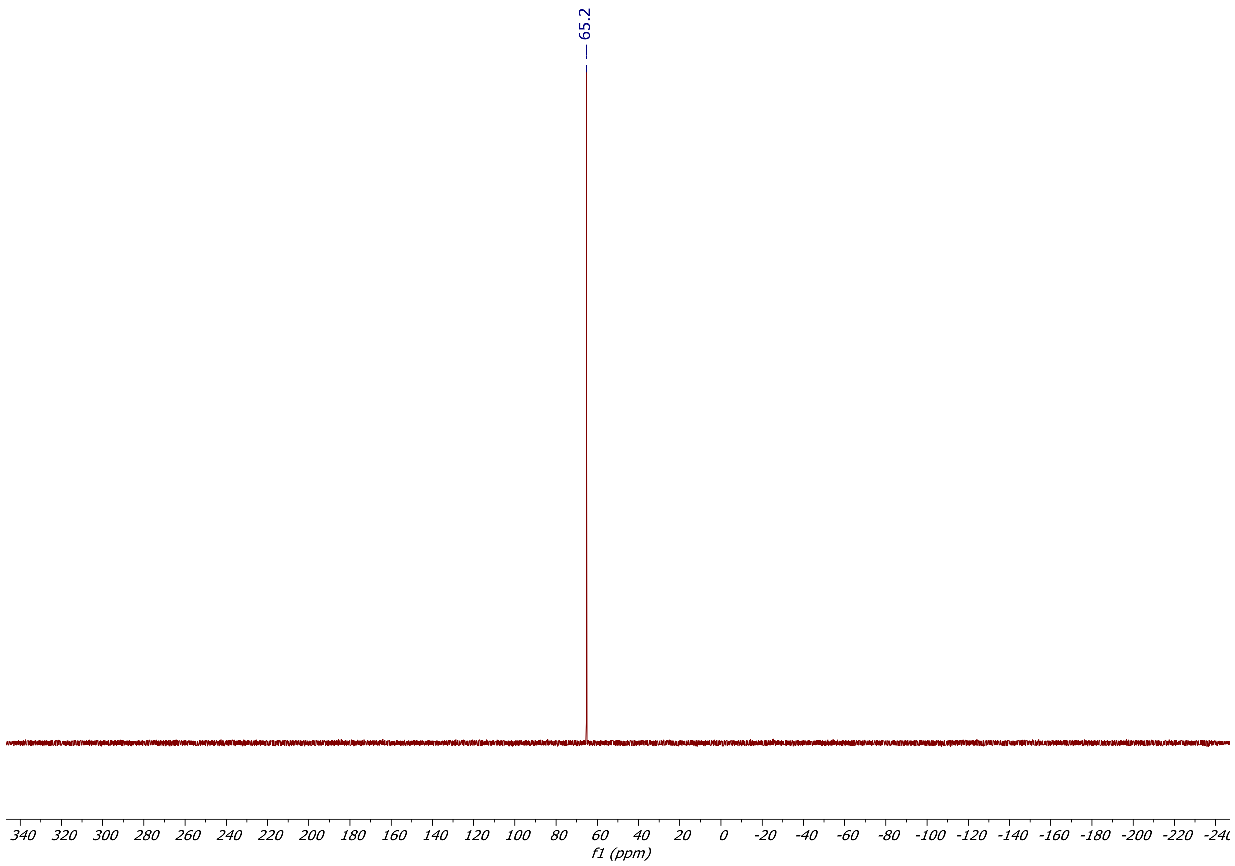

Figure S40. **$^{31}P\{^{1}H\}$ NMR** (162 MHz, D_2O, 298K) spectrum of **$[4b]^{2+}$**.

1.7) Synthesis of [4c-Ph]$^{2+}$, [4c-Naph]$^{2+}$ and [4c-Anth]$^{2+}$ from 1c, 1d, and 1e

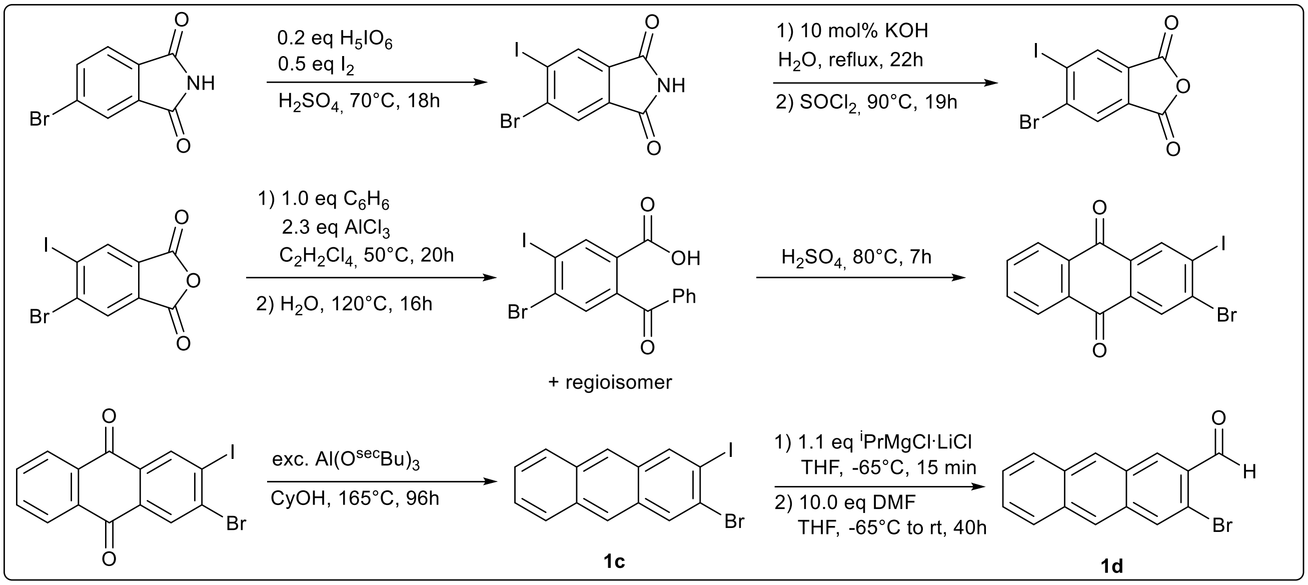

Scheme S4. Synthetic route for the synthesis of compounds **1c** and **1d**.

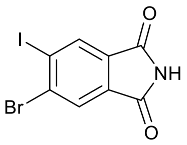

5-Bromoisoindoline-1,3-dione (99.4 g, 440 mmol,1.0 eq) and periodic acid (20.1 g, 88.0 mmol, 0.2 eq) were dissolved in sulfuric acid (500 mL). Iodine (50.2 g, 198 mmol, 0.5 eq) was added, and the mixture was stirred at 70 °C for 18 h. The mixture was cooled to rt and poured into water. The solid was collected by filtration and was washed with 2 wt% aq. K_2CO_3, sat. aq. $Na_2S_2O_3$, and water. 5-Bromo-6-iodoisoindoline-1,3-dione was obtained as an off-white solid (140 g, 398 mmol, 90%).**^{1}H NMR** (400 MHz, DMSO-*d$_6$*): δ (in ppm) = 11.56 (s, 1H), 8.29 (s, 1H), 8.10 (s, 1H). *These data are in accordance with literature*.[11]

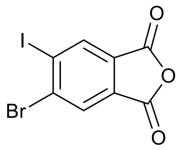

5-Bromo-6-iodoisoindoline-1,3-dione (140 g, 398 mmol, 1.0 eq) was added to an aqueous solution of 10 wt% KOH (500 mL) and heated under reflux fo r 22 h. The mixture was cooled to rt and 6 M aq. HCl-solution (ca. 75 mL) was added until pH 2 was reached. The precipitate was filtrated, washed with several volumes of water, and dried under high vacuum at 60 °C for 24 h. The so obtained crude product was then was added to a round bottom flask and heated up to 60 °C. $SOCl_2$ (250 mL) was added and the mixture was stirred at 90 °C for 19 h. Removing the solvent under reduced pressure afforded 5-bromo-6-iodoisobenzofuran-1,3-dione as a yellow powder (122 g, 346 mmol, 87% over 2 Steps). **^{1}H NMR** (600 MHz, DMSO-*d$_6$*): δ (in ppm) = 8.15 (s, 1H), 7.93 (s, 1H). *These data are in accordance with literature.*[11]

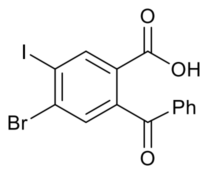

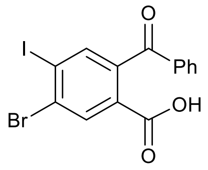

2-benzoyl-4-bromo-5-iodobenzoic acid/ 2-benzoyl-5-bromo-4-iodobenzoic acid were synthesized according to a modified procedure of *Ohashi* and coworkers[12]. 5-Bromo-6-iodoisobenzofuran-1,3-dione (118 g, 333 mmol, 1.0 eq) and benzene (26.0 g, 29.6 mL, 333 mmol, 1.0 eq) were dissolved in tetrachloroethane (800 mL) under an argon atmosphere. Dry $AlCl_3$ (100 g, 752 mmol, 2.3 eq) was added to the reaction mixture. The reaction mixture was stirred at rt for 30 min and at 50 °C for 20 h. The solvent was removed under reduced pressure and the

mixture was poured into water. The precipitate was filtrated, washed with several volumes of water, dissolved in DMF (400 mL) and stirred at 90 °C for 16 h. The solvent was decanted off. The solid was suspended in water and stirred at 120 °C for 16 h. The solvent was decanted off and removed under reduced pressure, resulting a yellow powder (103 g, 239 mmol, 73%). The thus isolated product contained a nearly 1:1 mixture of both possible regioisomers. No attempts to separate these compounds were made, since the mixture of both regioisomers could be used in next step. **Mp:** 194-194.5 °C. **^{1}H NMR** (400 MHz, DMSO-d_6): δ (in ppm) = 8.42 (s, 1H), 8.17 (s, 1H), 8.00 (s, 1H), 7.82 (s, 1H), 7.67–7.63 (m, 5H), 7.53–7.49 (m, 5H), 7.43 (s, 1H), 6.96 (s, 1H). **^{13}C{^{1}H} NMR** (151 MHz, $CDCl_3$) δ (in ppm) = 194.6, 194.4, 168.1, 167.8, 143.3, 142.4, 141.8, 139.3, 136.3, 136.3, 136.1, 134.4, 133.9, 133.9, 131.6, 131.6, 129.6, 129.5, 129.0, 128.8, 127.7, 125.0, 108.5, 102.6. **EI HR-MS** (pos): *m/z* calcd for $C_{14}H_8BrIO_3^+$ $[M]^+$ 429.8696, found 429.8666; calcd for $C_8H_3BrIO_3^+$ $[M-Ph]^+$ 352.8305, found 352.8291. *Some ^{13}C NMR signals could not be observed due to signal overlap. An assignment of the ^{13}C{^{1}H} NMR peaks is not possible given that a mixture of both possible regioisomers was obtained.*

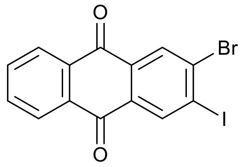

2-Bromo-3-iodoanthracene-9,10-dione was synthesized according to a modified procedure of *Ohashi* and coworkers[12]. A mixture of 2-Benzoyl-5-bromo-4-iodobenzoic acid and 2-benzoyl-4-bromo-5-iodobenzoic acid (**73**) (56.6 g, 197 mmol, 1.0 eq) was suspended in conc. H_2SO_4 (320 mL) and stirred at 80 °C for 7 h. The mixture was poured into water, the resulting solid was filtered off and washed with water. The dark solid was extracted with hot toluene (6 × 200 mL) and filtered off. Recrystallization from toluene afforded 2-bromo-3-iodoanthracene-9,10-dione as a yellow solid (23.5 g, 56.9 mmol, 43%). **Mp:** 262.9 °C **^{1}H NMR** (600 MHz, $CDCl_3$): δ (in ppm) = 8.77 (s, 1H), 8.48 (s, 1H), 8.32–8.30 (m, 2H), 7.85–7.83 (m, 2H). **^{13}C{^{1}H} NMR** (151 MHz, $CDCl_3$): δ (in ppm) = 182.0 (C_q, 1C), 181.6 (C_q, 1C), 139.5 (CH, 1C), 137.3 (C_q, 1C), 134.8 (CH, 1C), 134.8 (CH, 1C), 133.7 (C_q, 1C), 133.3 (C_q, 1C), 133.1 (C_q, 1C), 132.1 (C_q, 1C), 131.2 (CH, 1C), 127.6 (CH, 1C), 127.6 (CH, 1C), 109.6 (C_q, 1C).**EI HR-MS** (pos): *m/z* calcd for $C_{14}H_6BrIO_2^+$ $[M]^+$ 411.8590, found 411.8587; calcd for $C_{14}H_6IO_2^+$ $[M-Br]^+$ 332.9407, found 332.9431.

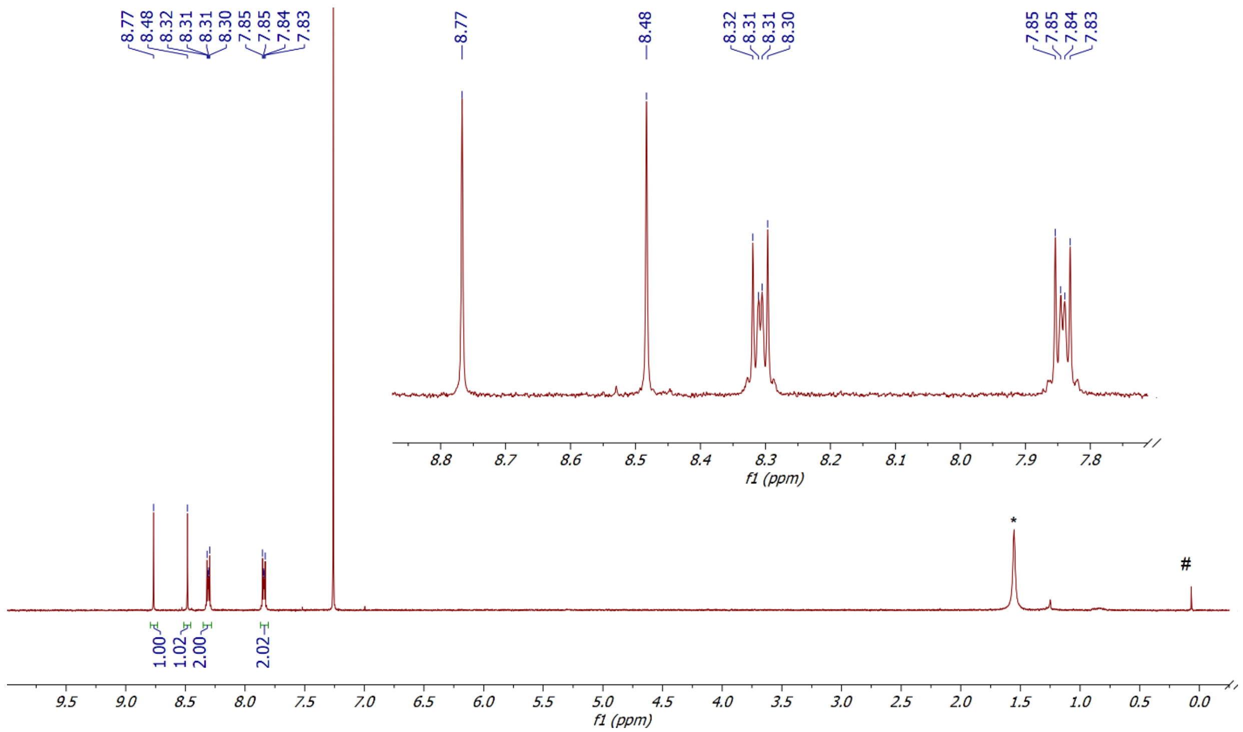

Figure S41. **^{1}H NMR** (600 MHz, $CDCl_3$, 298K) of 2-bromo-3-iodoanthracene-9,10-dione (traces of impurities of water (*) and silicon grease (#) are marked).

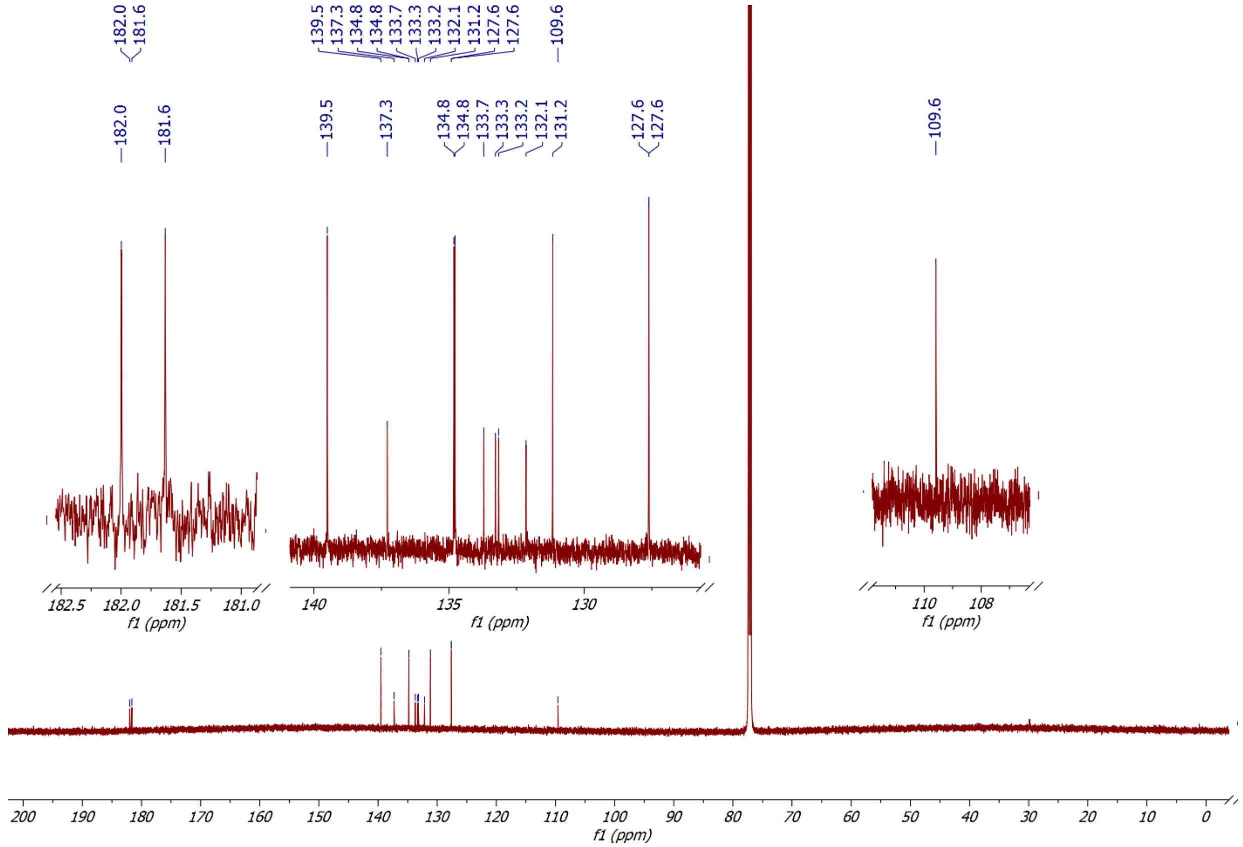

Figure S42. **$^{13}C\{^1H\}$ NMR** (151 MHz, $CDCl_3$, 298K) of 2-bromo-3-iodoanthracene-9,10-dione.

2-Bromo-3-iodoanthracene was synthesized according to a modified procedure of *Gerlach* and coworkers[13]. 2-Bromo-3-iodoanthracene-9,10-dione (20.0 g, 48.8 mmol, 1.0 eq) was added to a three-necked flask, equipped with a distillation head under argon atmosphere. Al($O^{sec}Bu$)$_3$ (179 g, 185 ml, 726 mmol) and cyclohexanol (500 mL) were added. The mixture was heated to 145 °C and progressively to 165 °C while the distillate was collected in a collection flask and was continued until the foam formation in the reaction flask stopped. The mixture was stirred at 165 °C for 96 h. After cooling to 100 °C the mixture was poured into a mixture of aq. conc. HCl, water and methanol (800 mL, 1:2:5) and stirred for 3 h. The yellow suspension was filtrated and washed with water (200 mL) and methanol (800 mL). 2-Bromo-3-iodoanthracene was obtained as a yellow solid (12.2 g, 31.8 mmol, 66%) without further purification. **Mp:** 263 °C. **^{1}H NMR** (600 MHz, $CDCl_3$): δ (in ppm) = 8.61 (s, 1H), 8.33 (s, 1H), 8.30-8.28 (m, 2H), 8.00-7.97 (m, 2H), 7.51–7.50 (m, 2H). **^{13}C{^{1}H} NMR** (151 MHz, $CDCl_3$): δ (in ppm) = 140.1 (CH, 1C), 132.5 (C_q, 1C), 132.2 (C_q, 1C), 131.5 (C_q, 1C), 131.3 (C_q, 1C), 131.0 (CH, 1C), 128.5 (CH, 1C), 128.3 (CH, 1C), 126.6 (CH, 1C), 126.4 (CH, 1C), 125.7 (CH, 1C), 125.6 (CH, 1C), 125.4 (C_q, 1C), 97.9 (C_q, 1C).**EI HR-MS** (pos): *m/z* calcd for $C_{14}H_8BrI^+$ [M]$^+$ 381.8849, found 381.8873; calcd for $C_{14}H_8Br^+$ [M-I]$^+$ 254.9804, found 254.9836; calcd for $C_{14}H_8^+$ [M-I-Br]$^+$ 176.0621, found 176.0640.

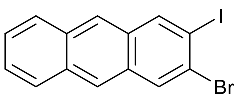

1c

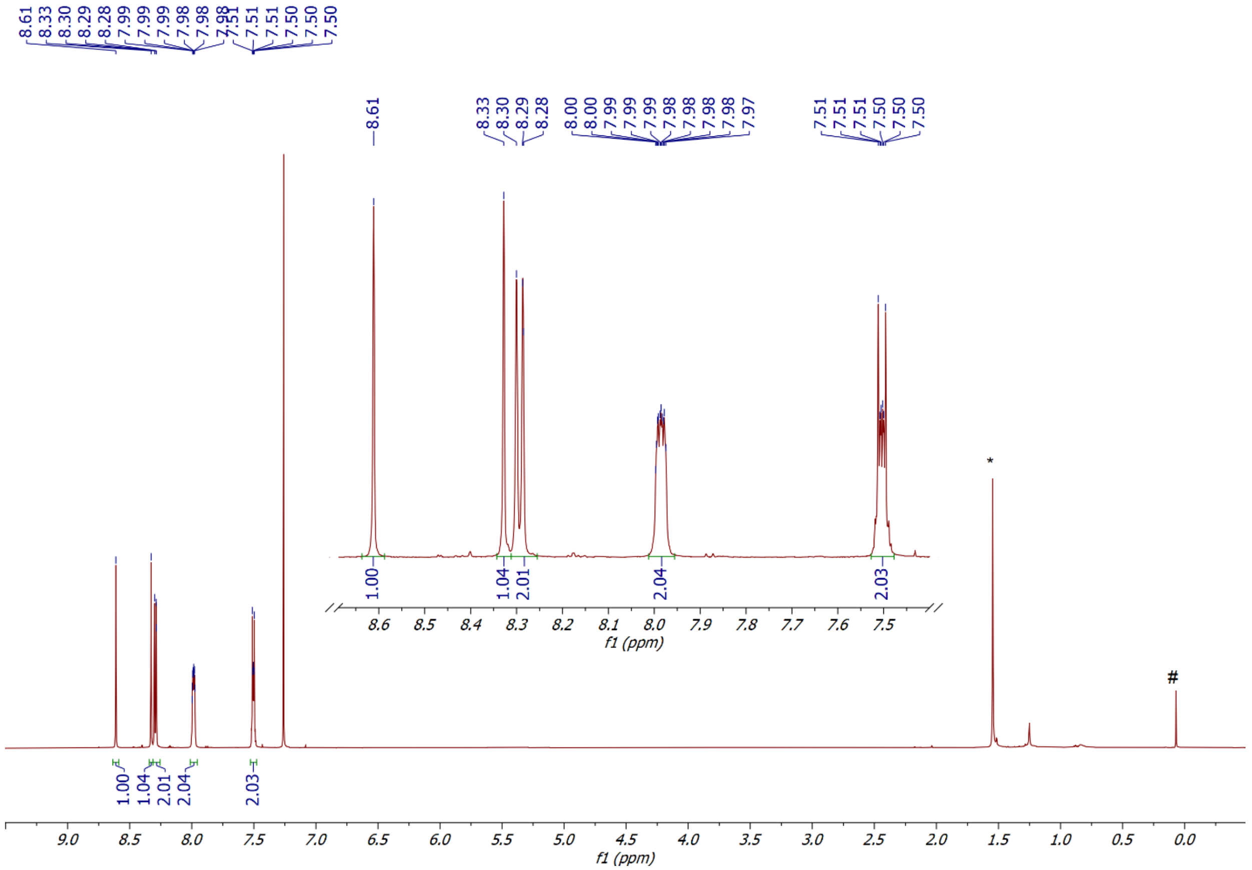

Figure S43. **^{1}H NMR** (600 MHz, $CDCl_3$, 298K) of **1c** (traces of impurities of water (*) and silicon grease (#) are marked).

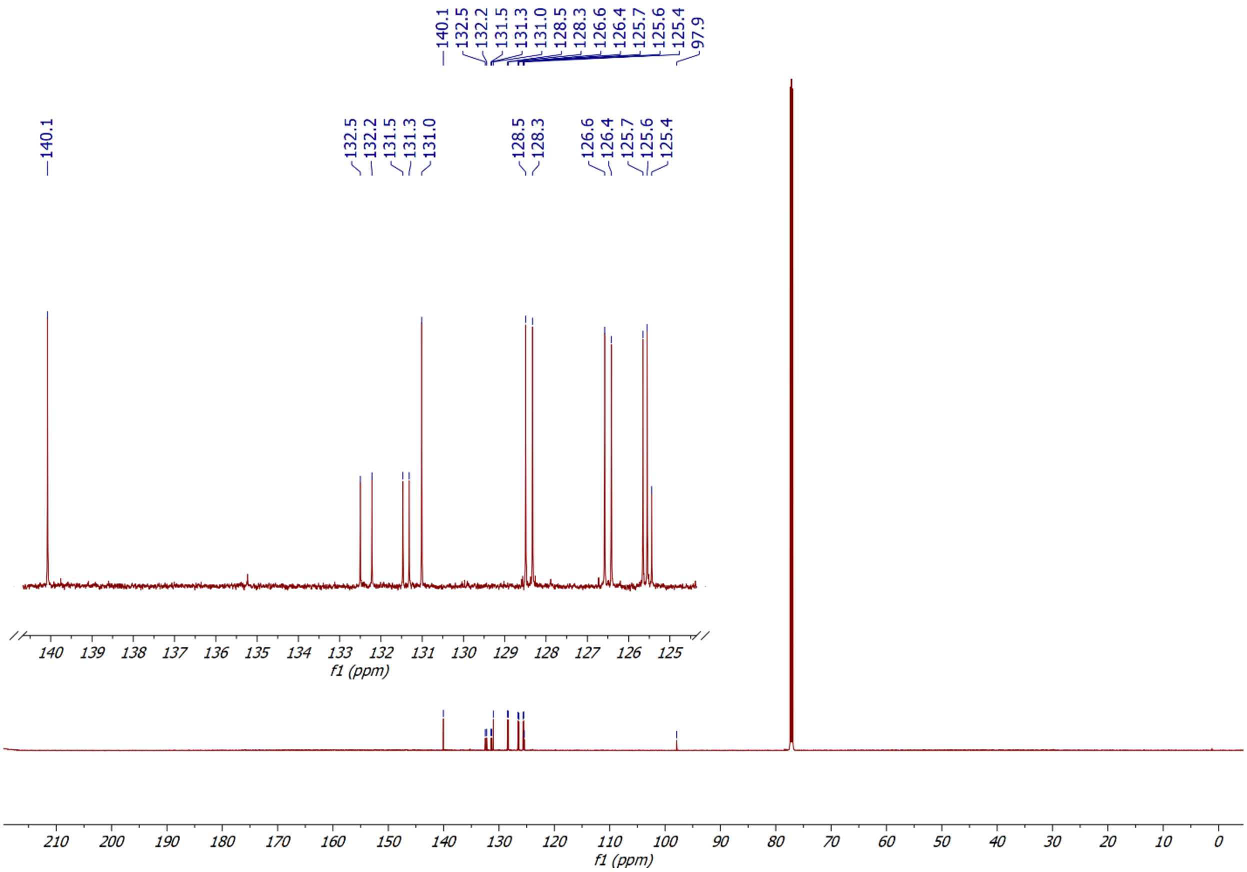

Figure S44. **^{13}C{^{1}H} NMR** (151 MHz, $CDCl_3$, 298K) of **1c**.

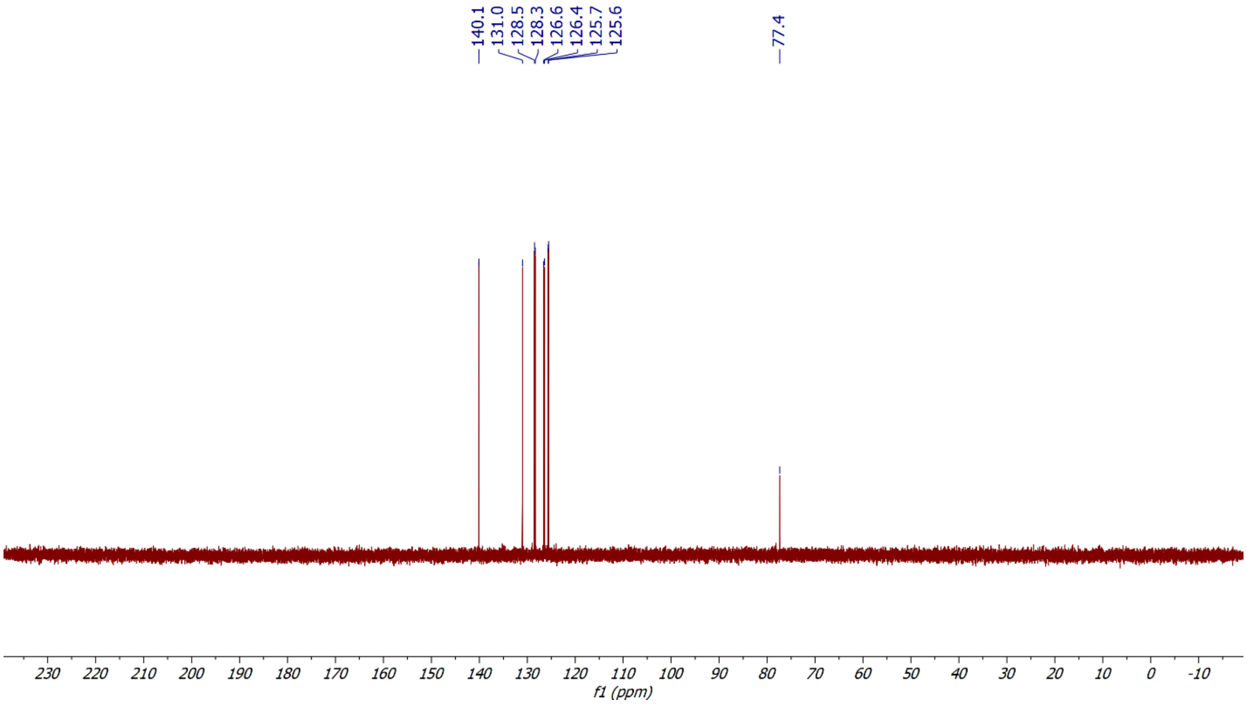

Figure S45. **^{13}C{^{1}H} DEPT-135 NMR** (151 MHz, $CDCl_3$, 298K) of **1c**.

2-Bromo-3-iodoanthracene (4.50 g, 11.8 mmol, 1.0 eq) was dissolved in dry THF (400 mL) and was cooled to –65 °C. A 1.3 M THF solution of *i*PrMgCl·LiCl (9.94 ml, 12.9 mmol, 1.1 eq) was added dropwise and the reaction mixture was stirred at –65 °C for 15 min. DMF (8.59 g, 9.10 ml, 117 mmol, 10.0 eq) was added at once and the mixture was stirred at rt for 40 h. The reaction was quenched with an aqueous saturated solution of NH_4Cl (90 mL) and the organic phase was removed under reduced pressure. The yellow precipitate was filtrated and washed with water. The crude product was purified by column chromatography (PE/DCM = 20:1, 10:1, EtOAc + 5% MeOH) and washed with toluene. 3-Bromoanthracene-2-carbaldehyde was obtained as a yellow solid (2.60 g, 9.12 mmol, 78%). **Mp:** 228.9 °C. **^{1}H NMR** (600 MHz, $CDCl_3$): δ (in ppm) = 10.53 (s, 1H), 8.67 (s, 1H), 8.61 (s, 1H), 8.36 (s, 1H), 8.30 (s, 1H), 8.03 (dd, $J_{H–H}$ = 11.9, 8.1 Hz, 2H), 7.59–7.54 (m, 2H). **^{13}C{^{1}H} NMR** (151 MHz, $CDCl_3$): δ (in ppm) = 192.1 (CH, 1C), 134.1 (C_q, 1C), 133.8 (CH, 1C), 133.6 (C_q, 1C), 132.7 (CH, 1C), 132.5 (C_q, 1C), 130.3 (CH, 1C), 130.0 (C_q, 1C), 129.2 (C_q, 1C), 128.8 (CH, 1C), 128.4 (CH, 1C), 127.8 (CH, 1C), 126.7 (CH, 1C), 125.6 (CH, 1C), 118.8 (C_q, 1C). **EI HR-MS** (pos): *m/z* calcd for $C_{15}H_9BrO^+$ [M]$^+$ 283.9831, found 283.9802; calcd $C_{14}H_8Br^+$ [M-CHO]$^+$ 254.9804, found 254.9806; calcd $C_{14}H_8^+$ [M-CHO-Br]$^+$ 176.0620, found 176.0614.

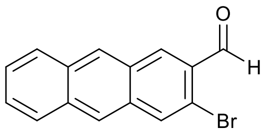

1d

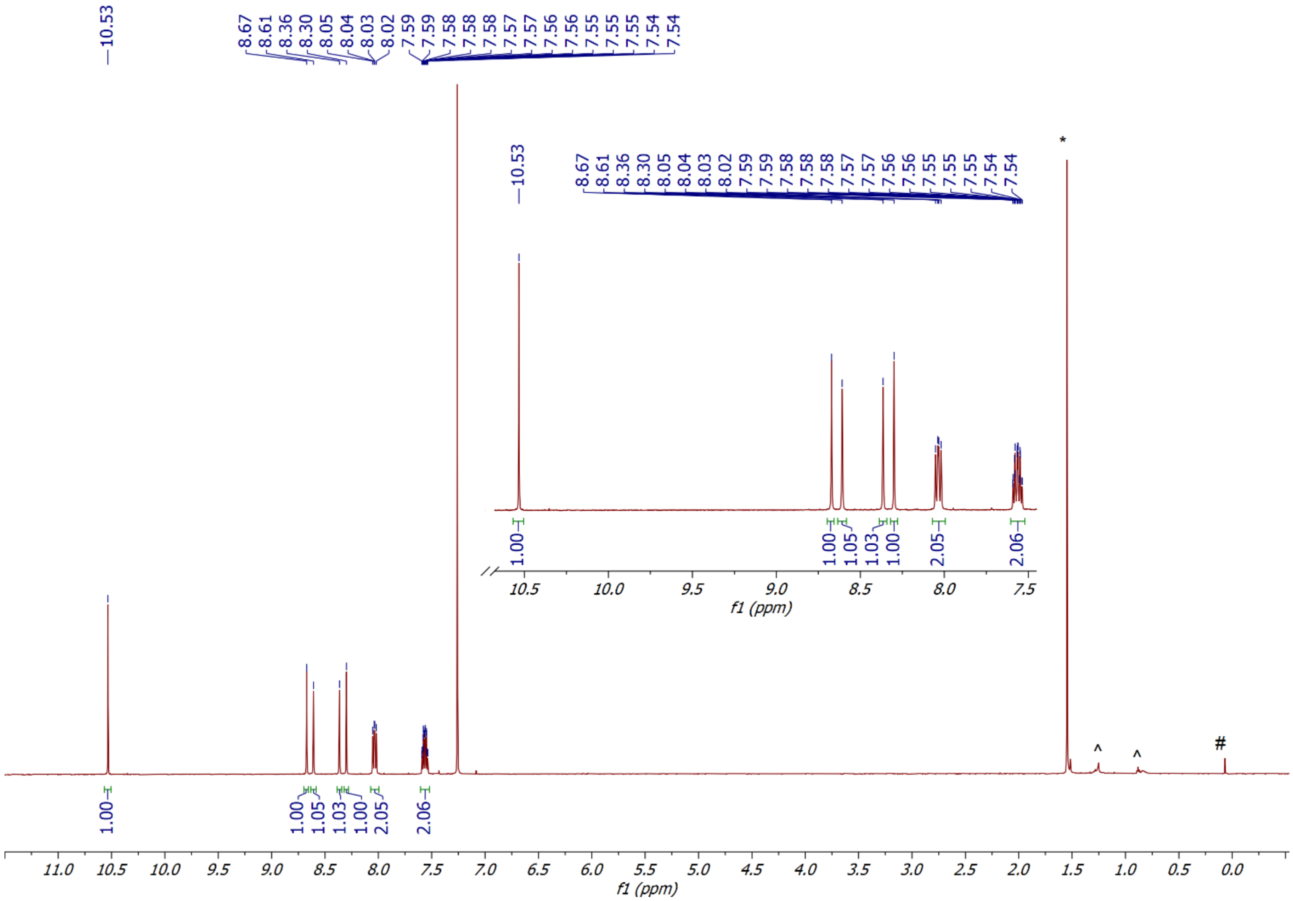

Figure S46. **^{1}H NMR** (600 MHz, $CDCl_3$, 298K) spectrum of **1d** (traces of impurities of water (*), hexanes (^) and silicon grease (#) are marked).

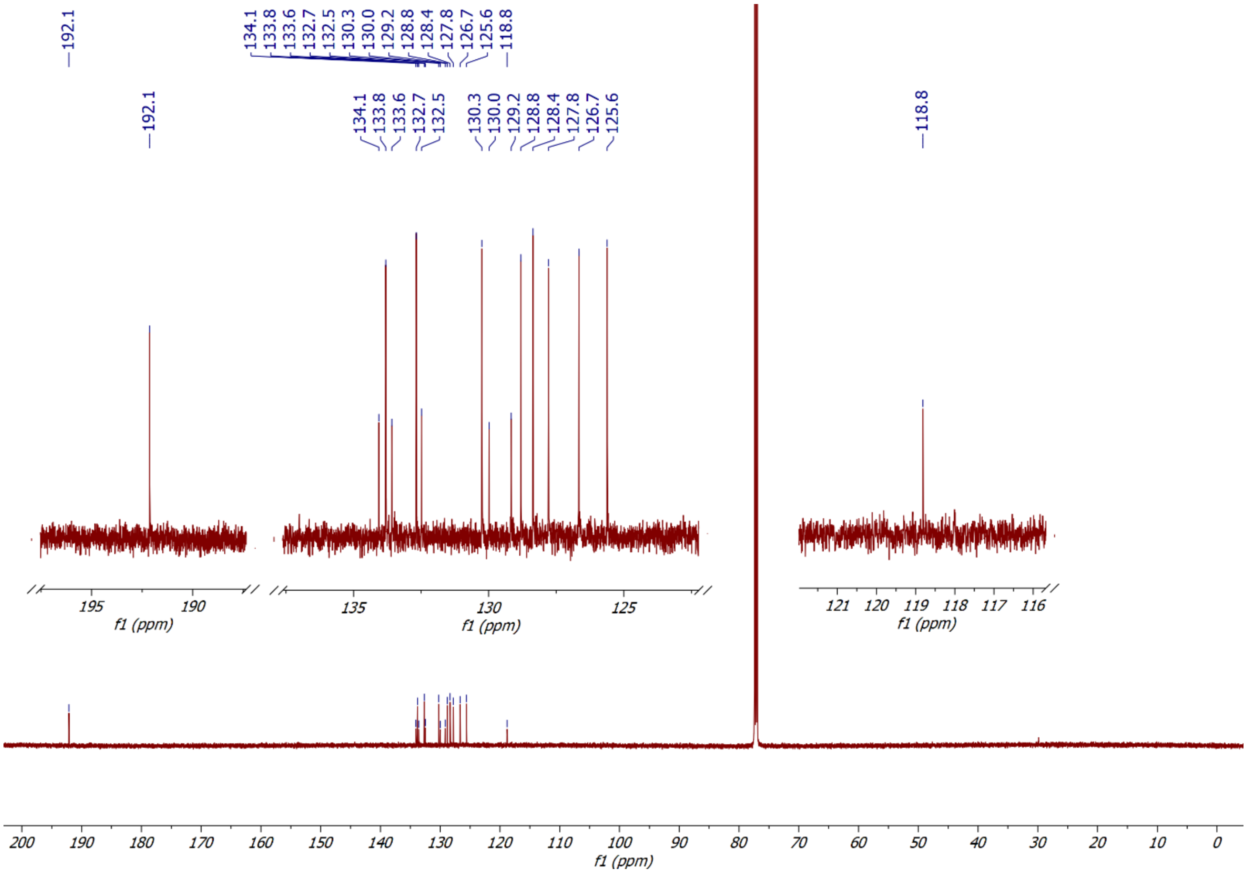

Figure S47. **$^{13}C\{^1H\}$ NMR** (151 MHz, $CDCl_3$, 298K) spectrum of **1d**.

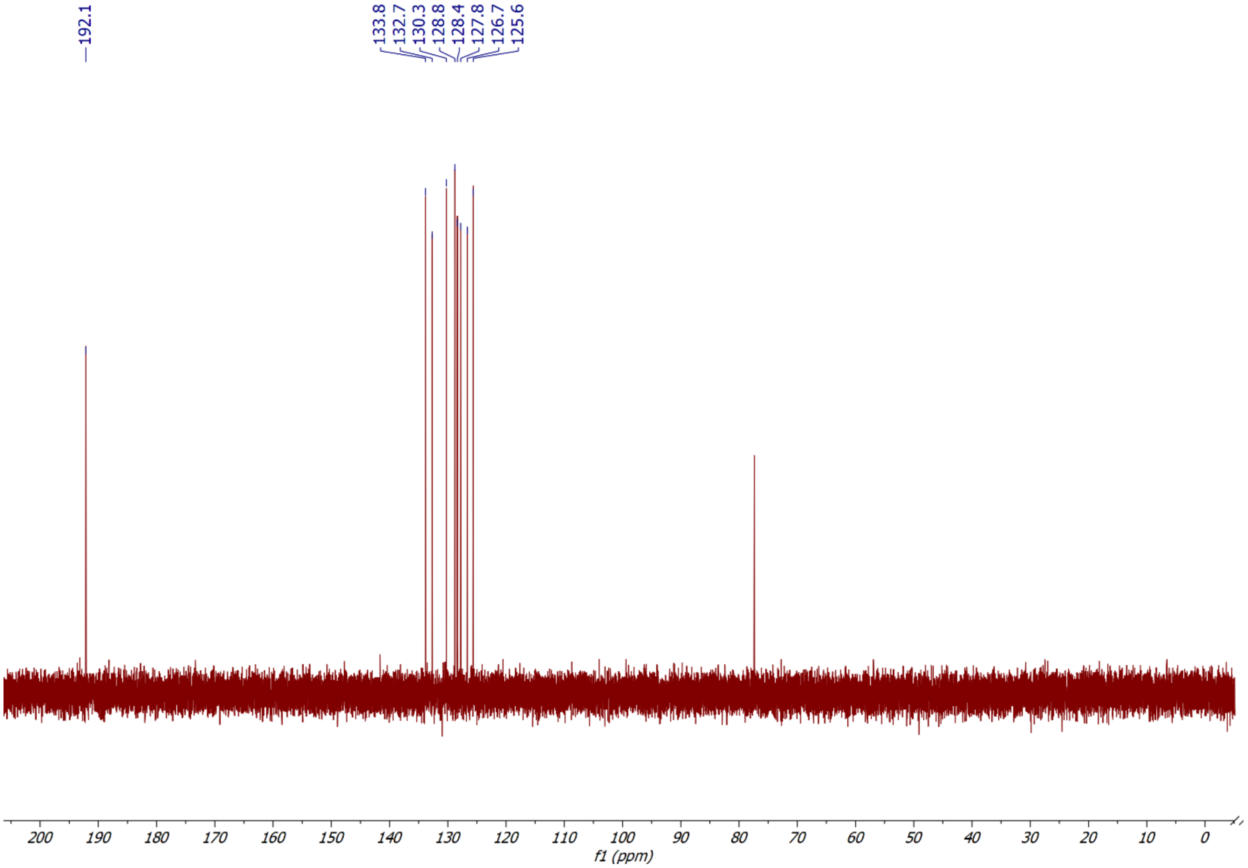

Figure S48. **$^{13}C\{^1H\}$ DEPT NMR** (151 MHz, $CDCl_3$, 298K) spectrum of **1d**.

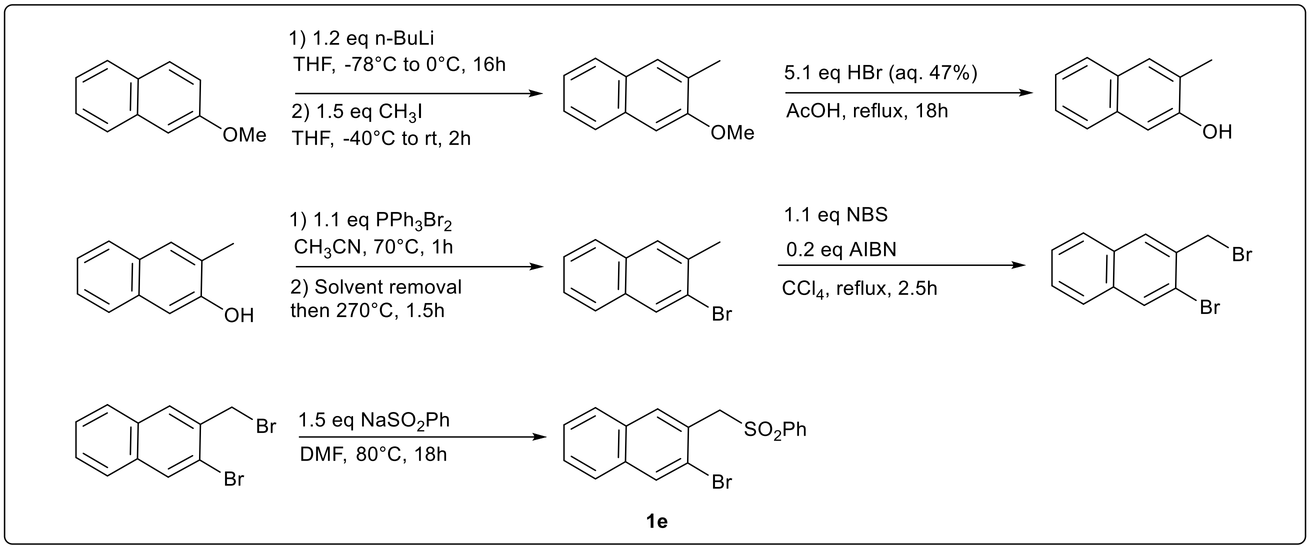

Scheme S5. Synthetic route for the synthesis of compound **1e.**

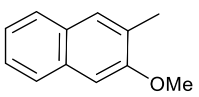

2-methoxynaphthalene (32.9 g, 0.21 mol, 1.00 eq.) was dissolved in tetrahydrofuran (200 mL) under an argon atmosphere. After the reaction mixture was cooled to -78 °C, 2.5 M *n*-BuLi solution in hexane (100 mL, 0.25 mol, 1.2 eq.) was added. The mixture was slowly warmed to 0 °C and stirred over a 18 h period. Then the red solution was cooled to -40 °C and iodomethane (19.5 mL, 0.31 mol, 1.5 eq.) was added dropwise. After the complete addition of iodomethane the solution turned yellow and was warmed to room temperature. The solvents were evaporated, and 300 ml water were added to the reaction mixture. The compound was extracted three times with 200 mL diethyl ether, the combined organic phases were dried with $MgSO_4$ and the solvent was evaporated. The product was obtained as a colorless powder (35.4 g, 204 mol, 98%).^{1}**H NMR** (600 MHz, $CDCl_3$): δ (in ppm) = 7.71 (t, J_{H-H} = 8.9 Hz, 2H), 7.58 (s, 1H), 7.41 – 7.36 (m, 1H), 7.34 – 7.29 (m, 1H), 7.08 (s, 1H), 3.95 (s, 3H), 2.38 (s, 3H). *These data are in accordance with literature*.[14]

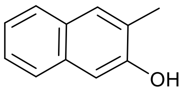

2-methoxy-3-methylnaphthalene (35.4 g, 204 mmol, 1.0 eq.) was dissolved in 300 ml acetic acid in a flask and 47% aqueous HBr (164 mL, 1.04 mol, 5.1 eq.) were added. The reaction mixture was stirred under reflux at 120 °C overnight. The volatiles were evaporated and 300 mL water and 150 mL diethyl ether were added and the resulting solution was stirred vigorously for 5 min at room temperature. The phases were separated and the aqueous phase was extracted three times with 200 mL diethyl ether. The combined organic phases were washed with 300 ml $Na_2S_2O_3$ solution, until no precipitation was observed anymore. Then the organic phase was washed with 300 mL water. After the solvents were evaporated the product was obtained as a brown powder (26.2 g, 0.16 mol, 80%) after recrystallization from hexane. ^{1}**H NMR** (400 MHz, $CDCl_3$): δ (in ppm) = 7.70 (d, J_{H-H} = 7.9 Hz, 1H), 7.64 (d, J_{H-H} = 8.0 Hz, 1H), 7.60 (s, 1H), 7.37 (t, J_{H-H} = 6.8 Hz, 1H), 7.29 (t, J_{H-H} = 7.5 Hz, 1H), 7.10 (s, 1H), 2.43 (s, 3H). *These data are in accordance with literature*.[15]

3-methylnaphthalen-2-ol (14.0 g, 88.6 mmol, 1.0 eq.) and PPh_3Br_2 (41.2 g, 97.5 mmol, 1.1 eq.) were dissolved in 100 mL acetonitrile under an argon atmosphere and heated to 70 °C. The solvent was evaporated and the reaction temperature was raised to 270 °C. After 90 min at this temperature the reaction mixture was cooled to 80 °C and 50 mL benzene and 150 mL hexane were added. The reaction solution was stirred under reflux overnight. On the following day the mixture was cooled down and filtered over a silica plug. The volatiles were evaporated. The resulting yellow powder was purified via flash column chromatography using pentane as eluent. The product was obtained as a colorless powder (10.7 g, 48.4 mmol, 55%). **^{1}H NMR** (400 MHz, $CDCl_3$): δ (in ppm) = 8.06 (s, 1H), 7.75 – 7.70 (m, 2H), 7.69 (s, 1H), 7.43-7.45 (m, 2H), 2.54 (s, 3H). *These data are in accordance with literature*. [16]

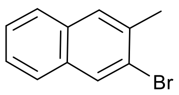

2-bromo-3-methylnaphthalene (10.1 g, 45.6 mmol, 1.0 eq.) was dissolved in 200 mL carbon tetrachloride under an argon atmosphere. NBS (8.94 g, 50.2 mmol, 1.1 eq.) and AIBN (1.50 g, 9.14 mmol, 0.2 eq.) were added and the reaction mixture was stirred under reflux for 2.5 h. After the solution cooled down the solvent was evaporated, and the residue was purified by a flash column with pentane as eluent. The product was obtained as a colorless powder (6.16 g, 20.5 mmol, 45%). **^{1}H NMR** (200 MHz, $CDCl_3$): δ (in ppm) = 8.11 (s, 1H), 7.95 (s, 1H), 7.82 – 7.73 (m, 2H), 7.57 – 7.45 (m, 2H), 4.77 (s, 2H). *These data are in accordance with literature*. [16]

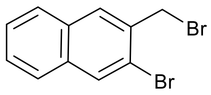

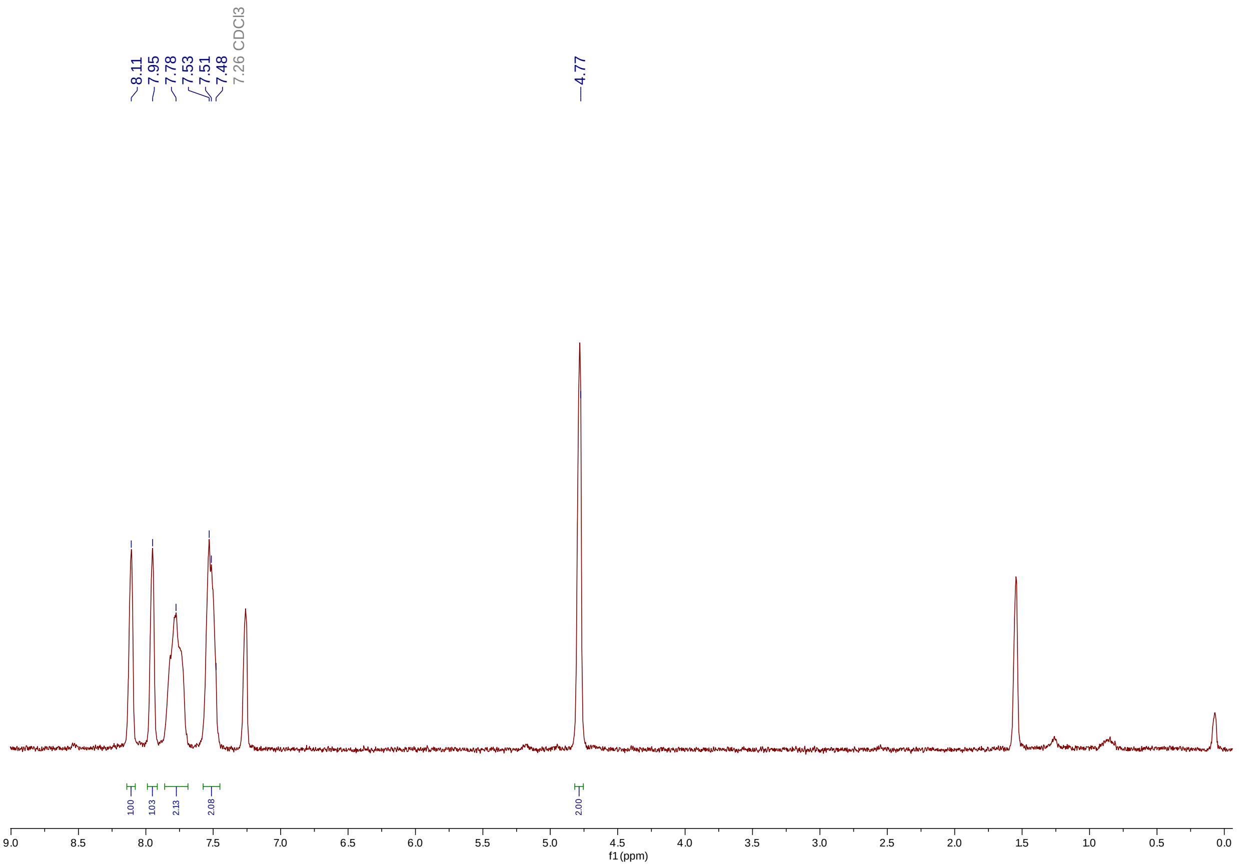

Figure S49. **^{1}H NMR** (200 MHz, $CDCl_3$, 298K) of 2-bromo-3-(bromomethyl)naphthalene.

2-bromo-3-(bromomethyl)naphthalene (5.50 g, 18.6 mmol, 1.00 eq.) and benzenesulfinic acid sodium salt (4.60 g, 27.9 mmol, 1.50 eq.) were dissolved in 50 mL DMF and stirred overnight at 80 °C. Afterwards the reaction solution was quenched with ice water. The precipitate was dissolved in DCM, followed by an aqueous workup. After the solvent was evaporated the product was obtained as a colorless powder (5.89 g, 16.3 mmol, 88%). **IR** $\tilde{\upsilon}$ [cm^{-1}] = 3045, 2927, 1584, 1495, 1481, 1449, 1404, 1298, 1258, 1242, 1193, 1154, 1125, 1024, 983, 958, 913, 868, 739, 683, 651. **^{1}H NMR** (600 MHz, $CDCl_3$): δ (in ppm) = 7.96 (d, $J_{H\text{-}H}$ = 12.2 Hz, 2H), 7.82-7.80 (m, 1H), 7.73-7.71 (m, 1H), 7.65-7.64 (m, 2H), 7.63-7.61 (m, 1H), 7.55-7.52 (m 2H), 7.44-7.41 (m, 2H), 4.75 (s, 2H). **^{13}C{^{1}H} NMR** (151 MHz, $CDCl_3$): δ (in ppm) = 138.30 (s, C_q, 1C,), 134.28 (s, C_q, 1C), 134.08 (s, CH, 1C), 132.86 (s, CH,1C), 132.12 (s, C_q, 1C), 131.70 (s, CH, 1C), 129.14 (s, CH, 2C), 129.06 (s, CH, 2C), 128.23 (s, CH, 1C), 127.93 (s, CH, 1C), 127.10 (s, CH, 1C), 126.79 (s, CH, 1C), 125.37 (s, C_q, 1C), 122.67 (s, C_q, 1C), 61.79 (s, CH_2, 1C). **MALDI HR-MS** (pos): *m/z* calcd for $[C_{17}H_{13}BrO_2S+Na]^+$: 382.9712 $[M+Na]^+$, found: 382.9715 $[M+Na]^+$.

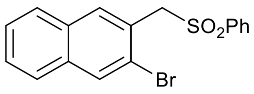

1e

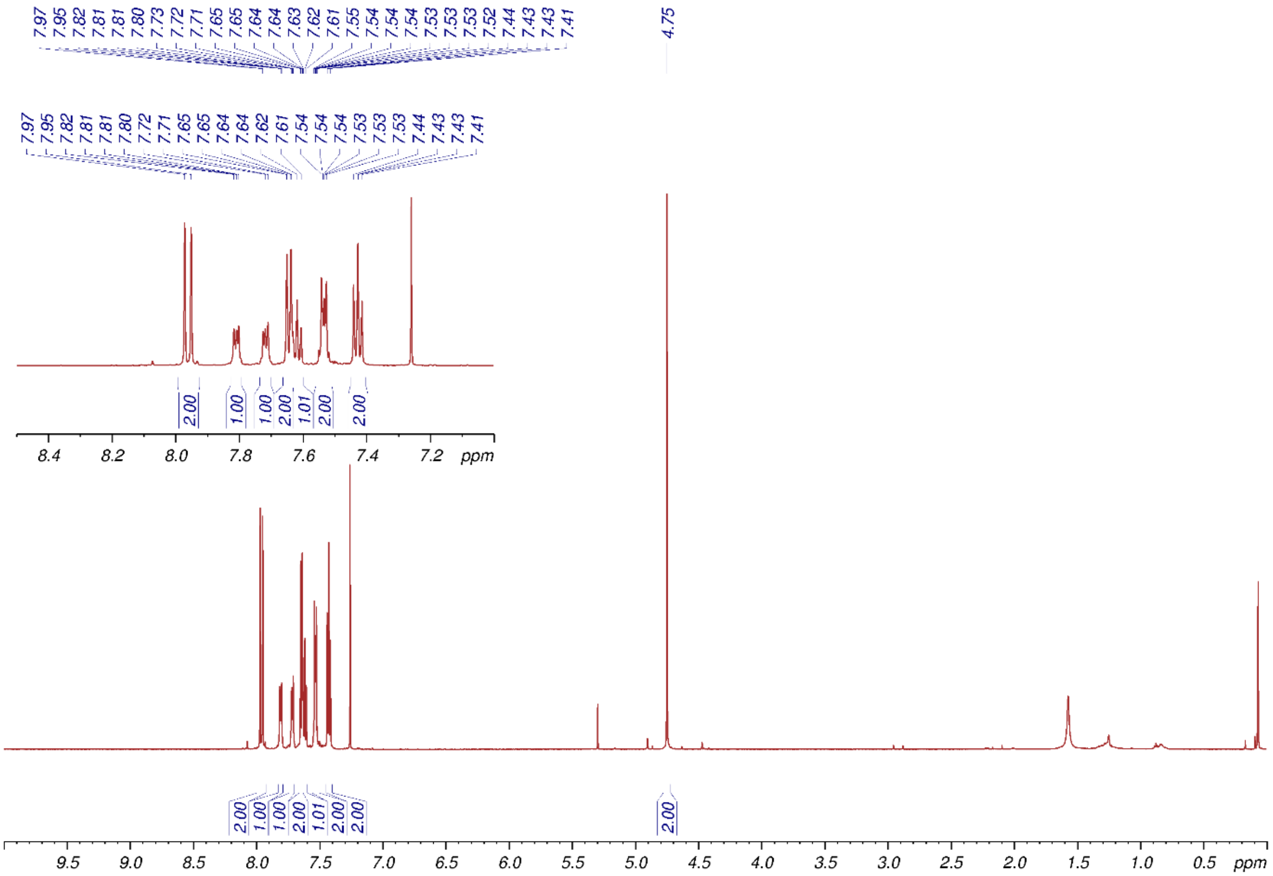

Figure S50. **^{1}H NMR** (600 MHz, $CDCl_3$, 298K) of **1e**.

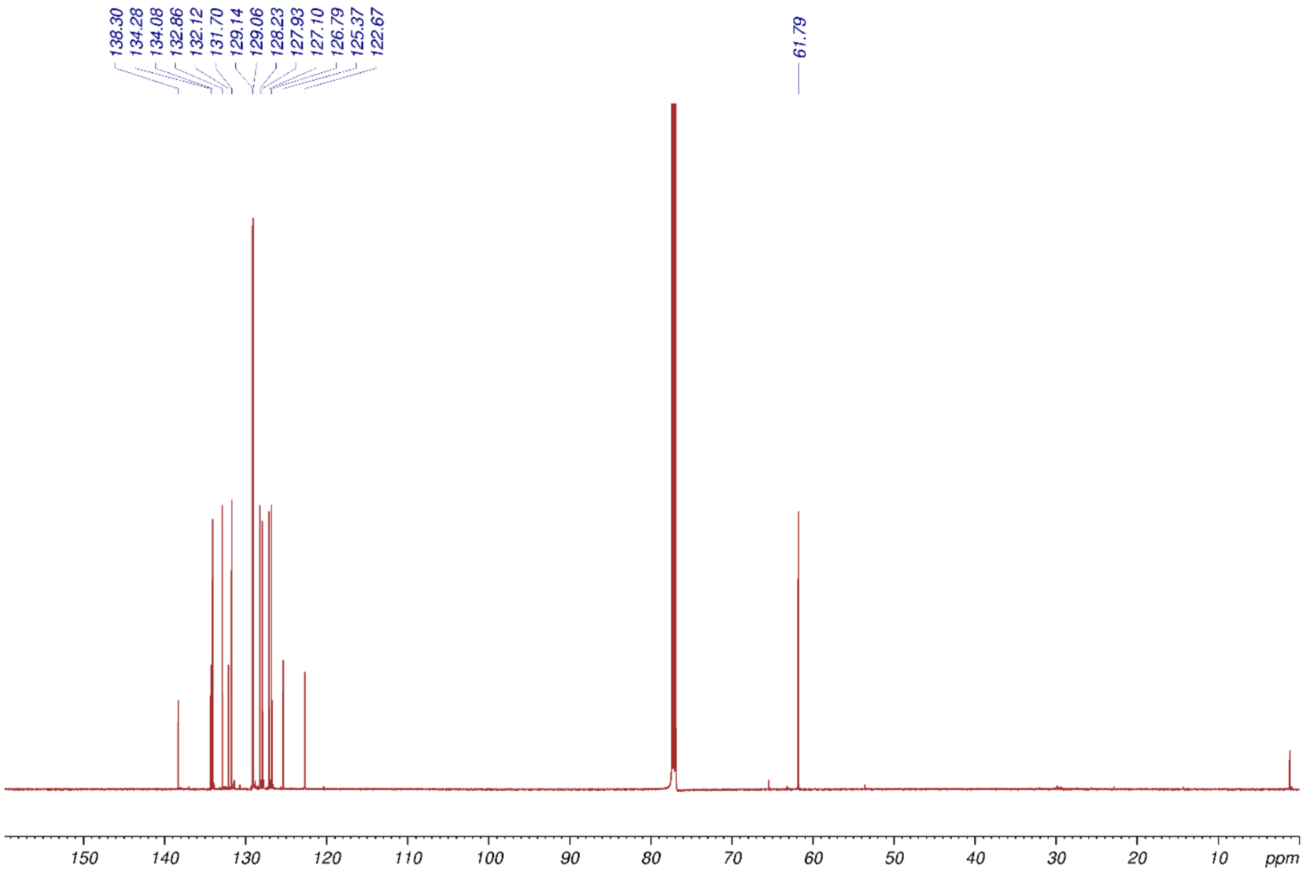

Figure S51. **^{13}C NMR {^{1}H}** (151 MHz, $CDCl_3$, 298K) of **1e**.

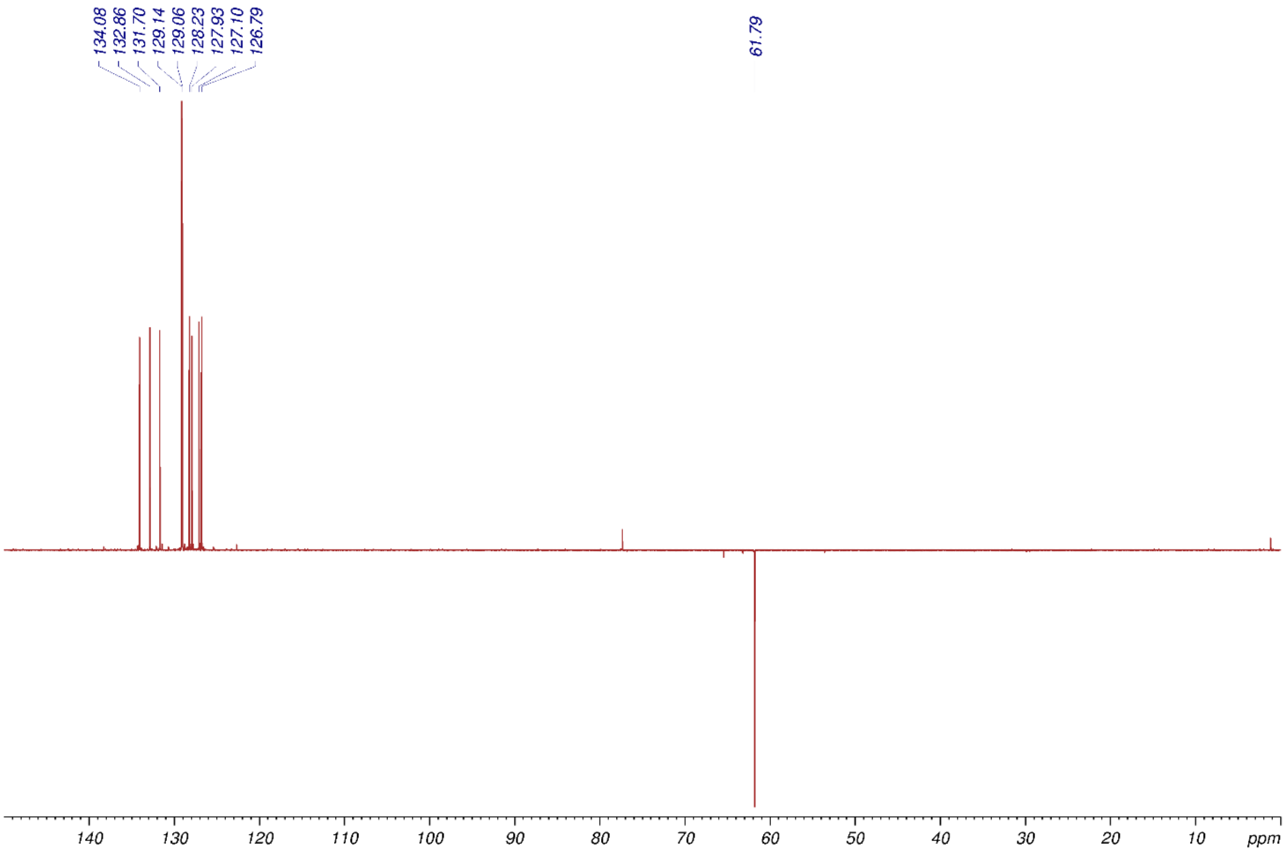

Figure S52. **^{13}C DEPT-135 NMR {^{1}H}** (151 MHz, $CDCl_3$, 298K) of **1e**.

1c (1.25 g, 3.26 mmol, 1.0 eq), $Pd(PPh_3)_4$ (113 mg, 97.9 µmol, 3.0 mol%) and **1d** (1.18 g, 3.43 mmol, 1.05 eq) were stirred in dry 1,4-dioxane (30 mL) at 100 °C for 19 h. After the mixture cooled to rt, the solvent was removed under reduced pressure and the crude red solid was washed with PE. Purification by two-fold column chromatography (SiO_2, PE/CH_2Cl_2 5:1, 3:1, 2:1, 1:1) afforded compound **2c-Ph** as a yellow solid (662 mg, 1.52 mmol, 47%). **Mp**: 198 °C. **IR** $\tilde{\nu}$ [cm^{-1}] = 1610, 1470, 1428, 1283, 1040, 1026, 996, 958, 908, 860, 739, 707, 667, 645, 596, 565, 539, 518, 474, 464, 435 **^{1}H NMR** (600 MHz, $CDCl_3$): δ (in ppm) = 8.40 (s, 1H), 8.33-8.29 (m, 3H), 8.00–7.99 (m, 2H), 7.69–7.65 (m, 2H), 7.52–7.49 (m, 2H), 7.34 (td, $J_{H\text{-}H}$ = 7.6, 1.2 Hz, 1H), 7.23 (td, $J_{H\text{-}H}$ = 7.8, 1.7 Hz, 1H). **^{13}C NMR** (151 MHz, $CDCl_3$): δ (in ppm) = 134.3 (CH, 1C), 133.8 (CH, 1C), 132.8 (C_q, 1C), 132.7 (CH, 1C), 132.3 (C_q, 1C), 131.5 (C_q, 1C), 131.2 (CH, 1C), 129.9 (CH, 1C), 129.7 (C_q, 1C), 128.6 (CH, 1C), 128.3 (CH, 1C), 127.2 (CH, 1C), 126.8 (CH, 1C), 126.7 (CH, 1C), 126.3 (CH, 1C), 125.7 (C_q, 1C), 125.5 (CH, 1C), 125.4 (C_q, 1C), 121.9 (C_q, 1C), 120.9 (C_q, 1C), 93.0 (C_q, 1C), 92.3 (C_q, 1C). **EI HR-MS** (pos): *m/z* calcd for $C_{22}H_{12}Br_2$ $[M]^+$ 433.9300, found 433.9270.

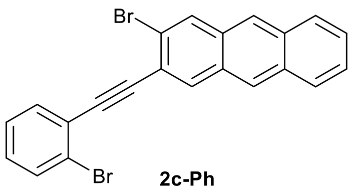

2c-Ph

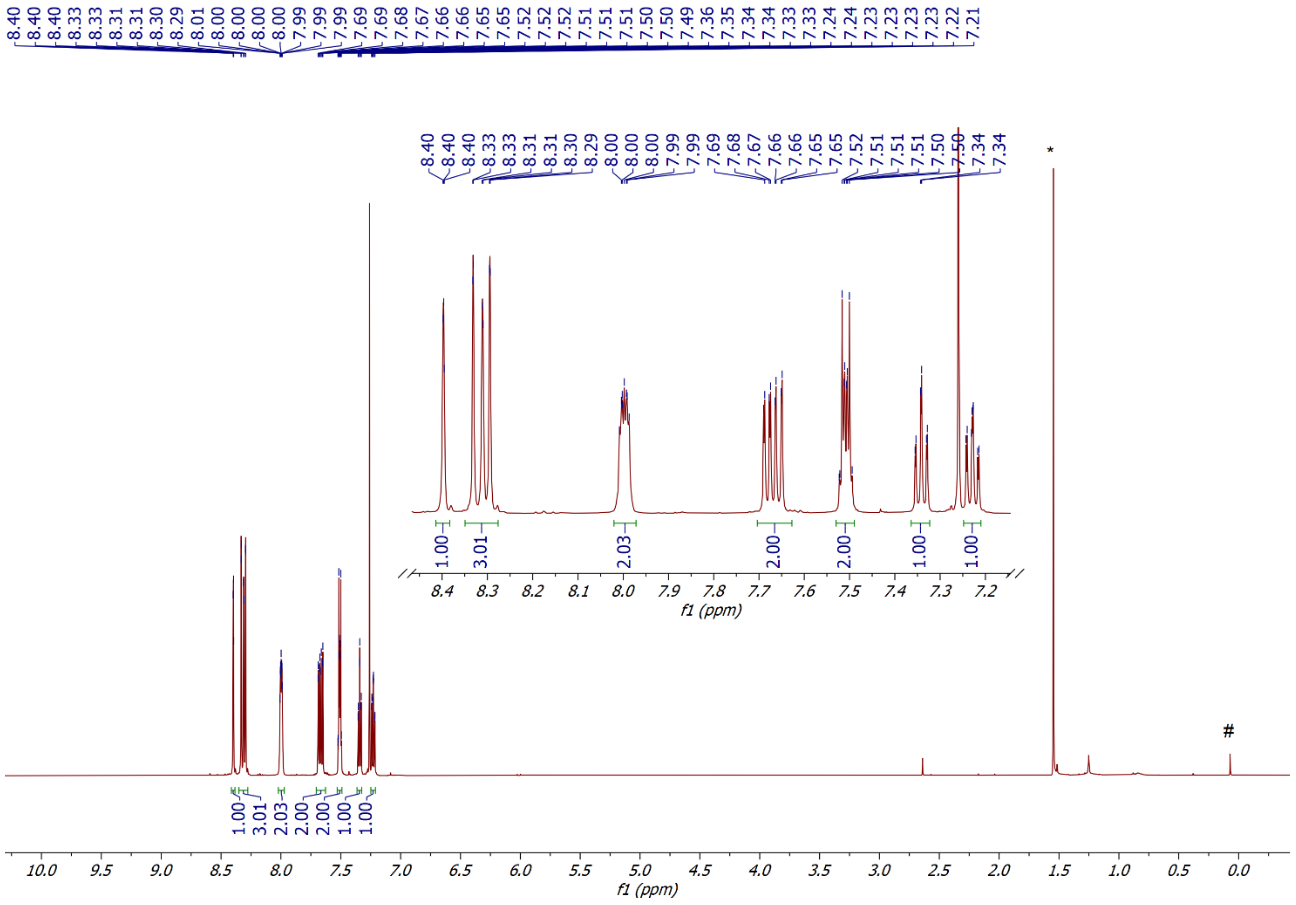

Figure S53. **^{1}H NMR** (600 MHz, $CDCl_3$, 298K) spectrum of **2c-Ph** (traces of impurities of water (*) and silicon grease (#) are marked).

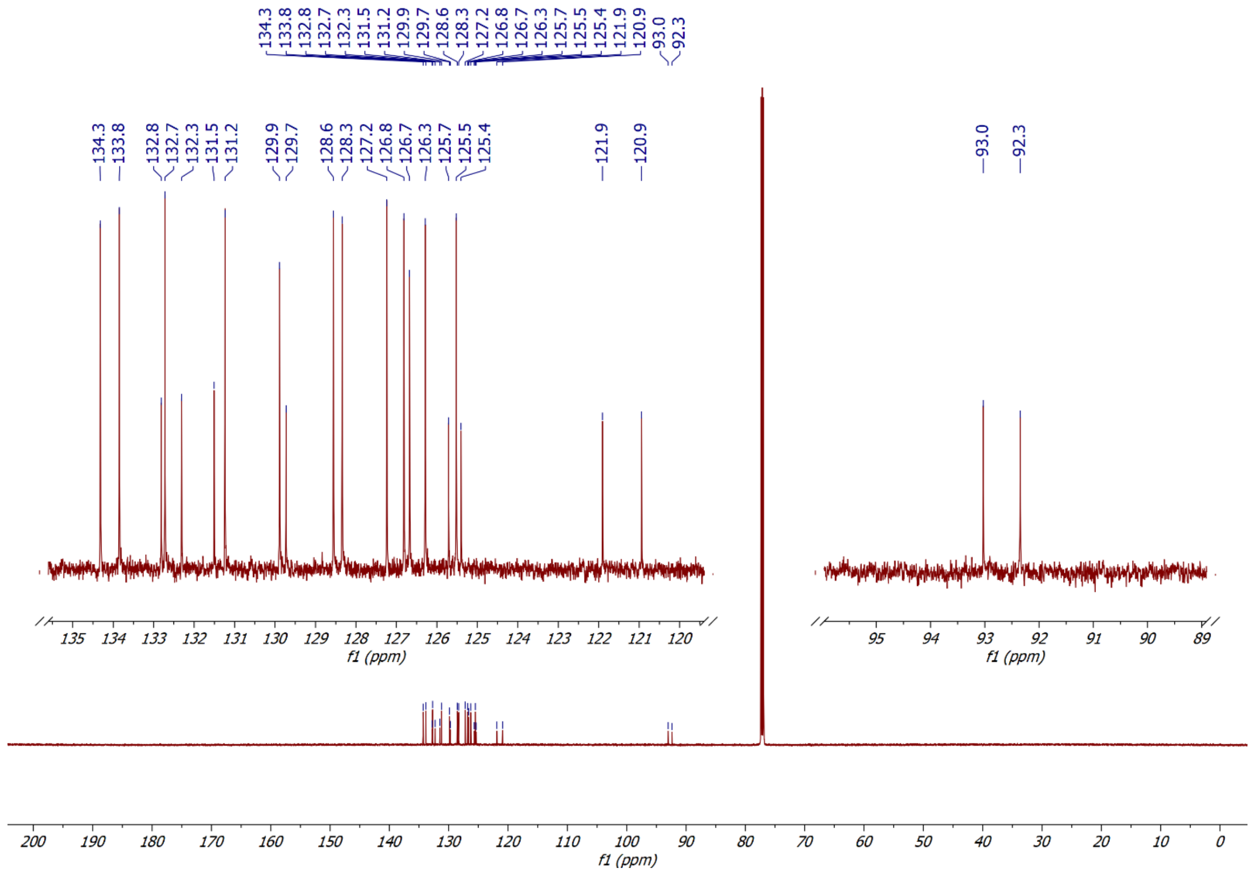

Figure S54. **^{13}C {^{1}H} NMR** (151 MHz, $CDCl_3$, 298K) spectrum of **2c-Ph**.

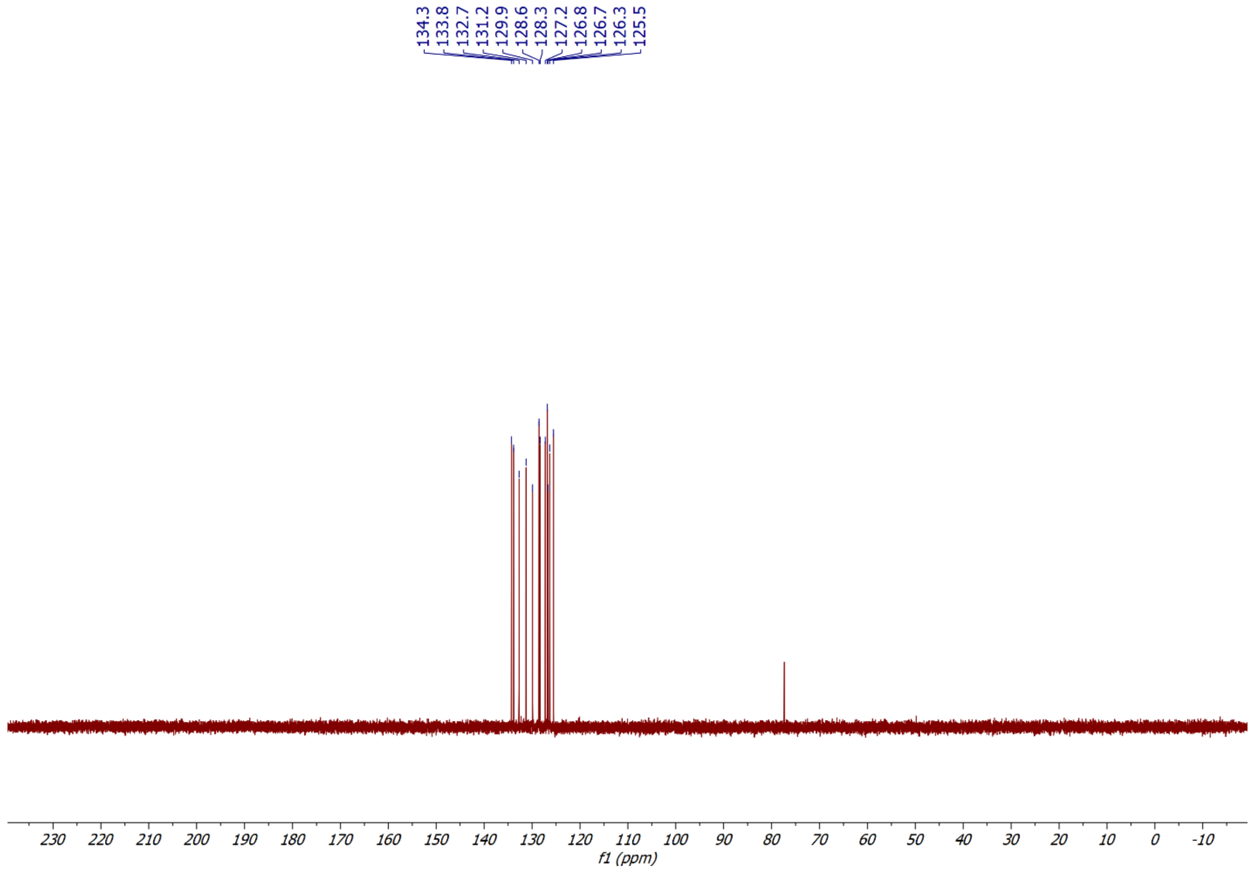

Figure S55. **^{13}C {^{1}H} DEPT-135 NMR** (151 MHz, $CDCl_3$, 298K) spectrum of **2c-Ph**.

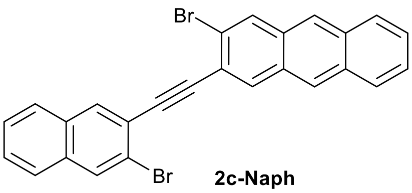

2c-Naph

1f (3.64 g, 10.1 mmol, 1.0 eq) was dissolved in dry THF (300 mL) under argon atmosphere. The solution was cooled to –78 °C, followed by the addition of LiHMDS (1.75 g, 10.5 mmol, 1.04 eq) over 30 min. The mixture was stirred for 15 min and a solution of **1e** (3.60 g, 12.6 mmol, 1.2 eq) in dry THF (200 mL) was added over 15 min and the mixture was stirred at –70 °C for another 30 min. Then the $POCl(EtO)_2$ (1.81 g, 10.5 mmol, 1.52 mL, 1.04 eq) was added dropwise. The reaction solution was warmed to 0 °C and stirred for an additional 1.5 h. A solution of KO^tBu (11.3 g, 101 mmol, 10.0 eq) in dry THF (100 mL) was added dropwise and the reaction mixture was stirred at room temperature for 17 h. The solution was cooled to 0 °C and quenched with a mixture of brine and water (1:1, 400 mL), then extracted with DCM (3 × 300 ml). The combined organic phase was dried (Na_2SO_4), filtered and the solvent was removed under reduced pressure. Purification by two-fold column chromatography (SiO_2, PE/CH_2Cl_2 20:1, 10:1, 5:1) afforded compound **2c-Naph** as a yellow solid (1.09 g, 2.24 mmol, 18%). Mp: 268 °C R_f: 0.14 (silica gel, PE:DCM = 40:1) **IR** $\tilde{\nu}$ [cm^{-1}] = 1580, 1489, 1461, 1427, 1319, 1283, 1009, 960, 908, 890, 860, 793, 748, 700, 670, 665, 586, 532, 475, 426. **^{1}H NMR** (600 MHz, $CDCl_3$): δ (in ppm) = 8.42 (s, 1H), 8.38 (s, 1H), 8.33 (s, 1H), 8.32 (s, 1H), 8.21 (s, 1H), 8.16 (s, 1H), 8.02–8.00 (m, 2H), 7.85–7.83 (m, 1H), 7.78–7.76 (m, 1H), 7.54–7.51 (m, 4H) **^{13}C{^{1}H} NMR** (151 MHz, $CDCl_3$): δ (in ppm) = 134.4 (CH, 1C), 134.0 (C_q, 1C), 133.8 (CH, 1C), 132.8 (C_q, 1C), 132.3 (C_q, 1C), 131.9 (C_q, 1C), 131.5 (C_q, 1C), 131.3 (CH, 1C), 131.3 (CH, 1C), 129.8 (C_q, 1C), 128.6 (CH, 1C), 128.4 (CH, 1C), 127.9 (CH, 1C), 127.9 (CH, 1C), 127.1 (CH, 1C), 127.1 (CH, 1C), 126.8 (CH, 1C), 126.7 (CH, 1C), 126.3 (CH, 1C), 125.5 (CH, 1C), 122.7 (C_q, 1C), 122.0 (C_q, 1C), 121.9 (C_q, 1C), 121.0 (C_q, 1C), 92.7 (C_q, 1C), 92.7 (C_q, 1C). **MALDI HR-MS** (pos): *m/z* calcd for $C_{26}H_{14}Br_2^+$ $[M]^+$ 483.9457, found 483.9459.

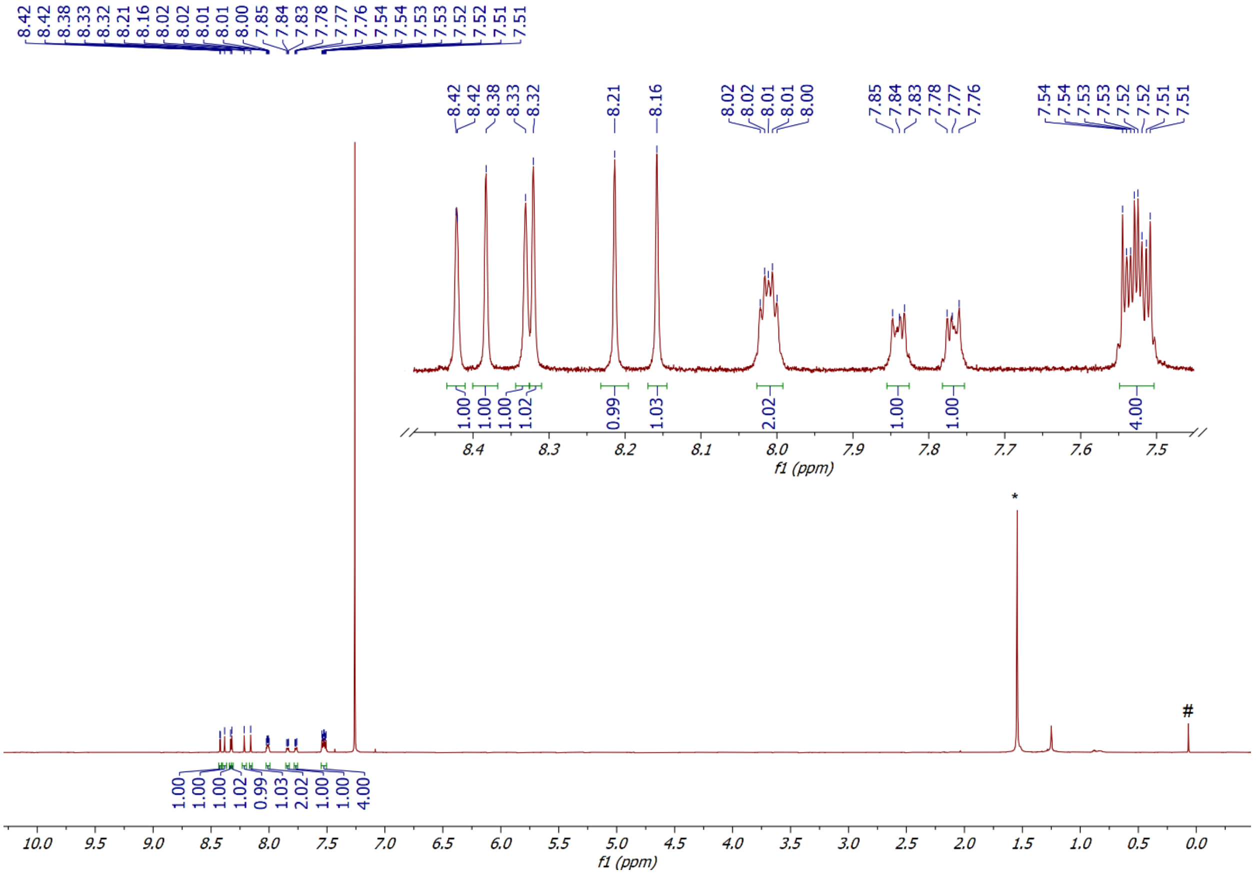

Figure S56. **^{1}H NMR** (600 MHz, $CDCl_3$, 298 K) spectrum of **2c-Naph** (traces of impurities of water (*) and silicon grease (#) are marked).

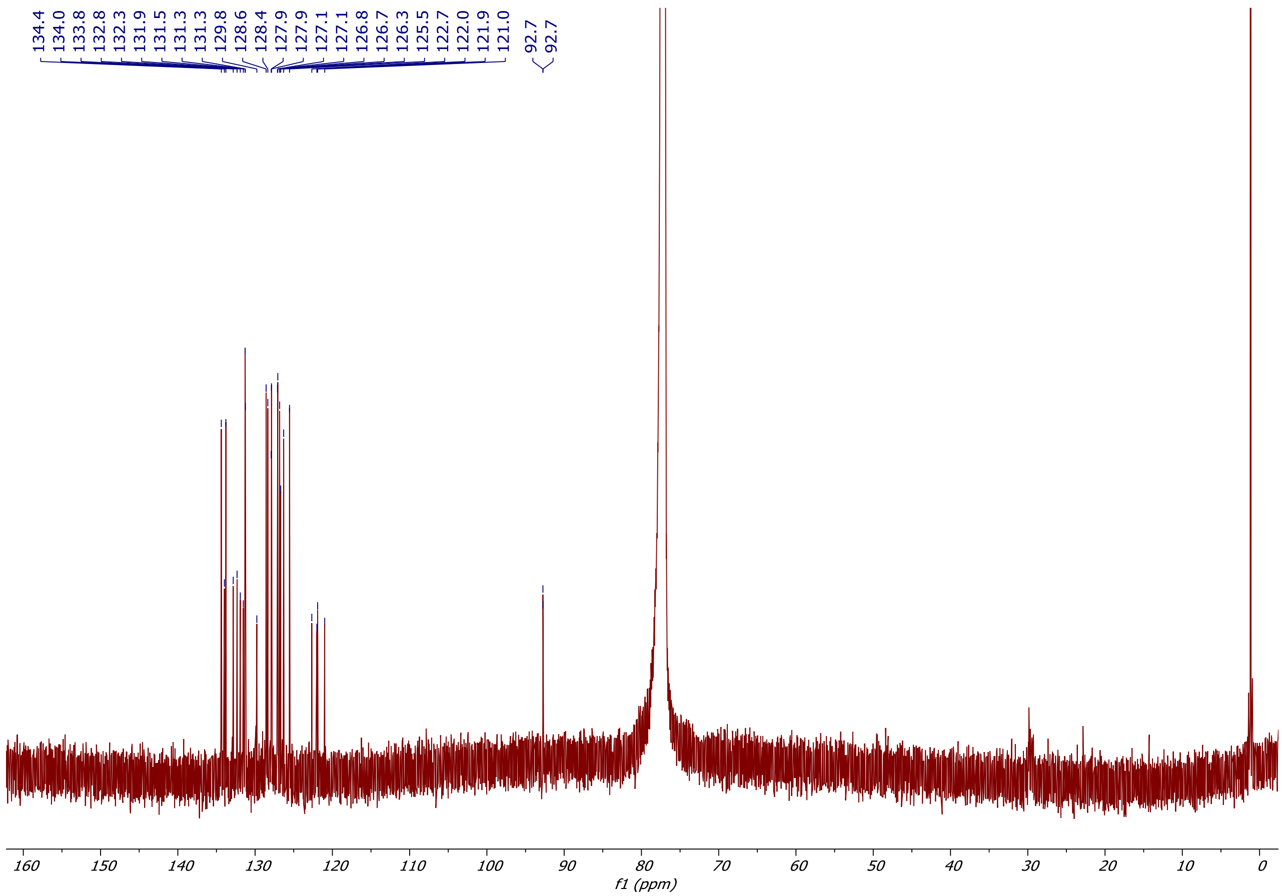

Figure S57. **^{13}C{^{1}H} NMR** (151 MHz, $CDCl_3$, 298K) spectrum of **2c-Naph**.

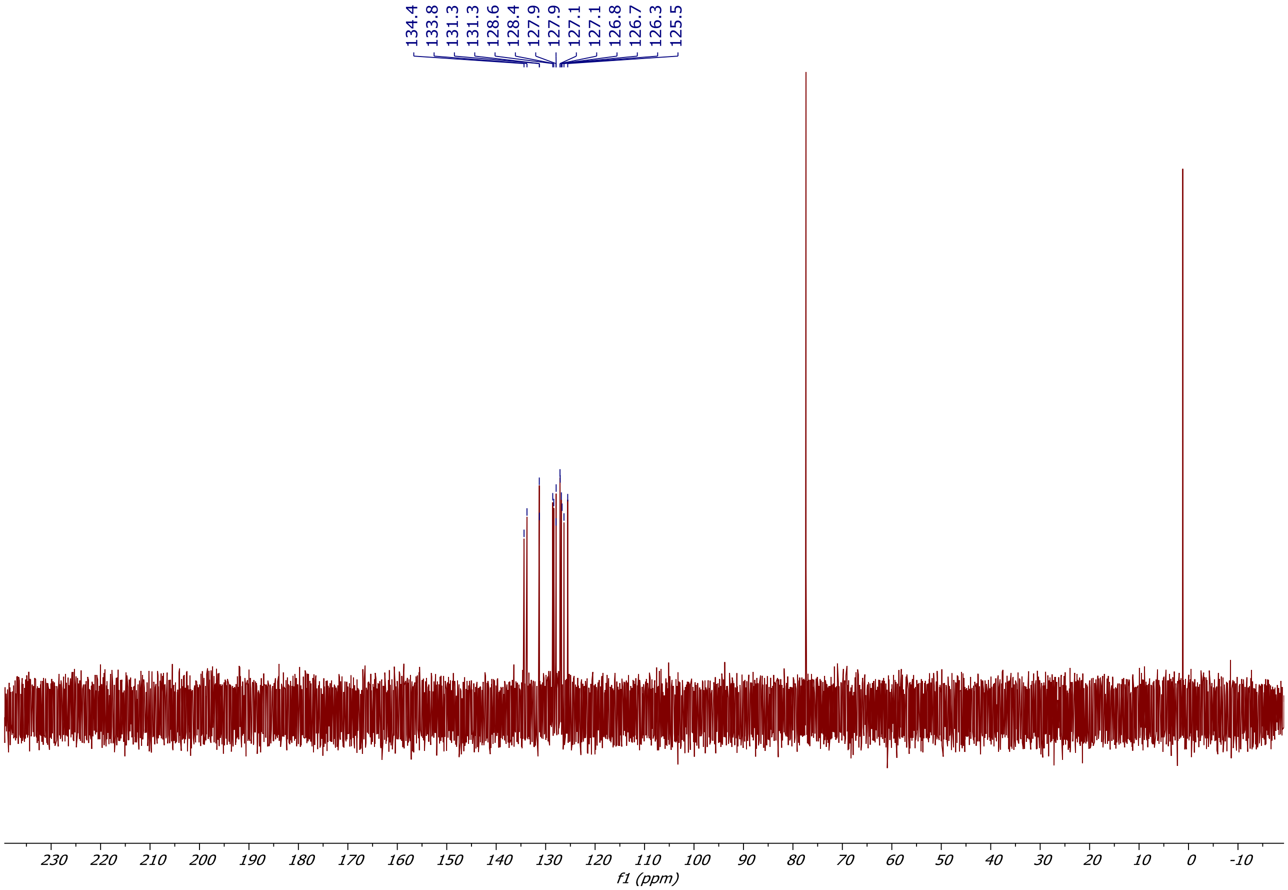

Figure S58. **^{13}C{^{1}H} DEPT-135 NMR** (151 MHz, $CDCl_3$, 298K) spectrum of **2c-Naph**.

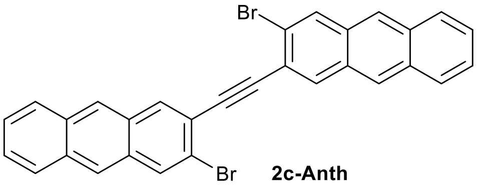

2c-Anth

1d (4.50 g, 11.8 mmol, 1.0 eq), $Pd(PPh_3)_4$ (339 mg, 294 μmol, 2.5 mol%), and 1,2-bis(tributylstannyl)ethyne (3.55 g, 5.87 mmol, 3.09 mL) were stirred under argon atmosphere in dry 1,4-dioxane (70 mL) at 100 °C for 20 h. After cooling the mixture to rt, the precipitate was filtered off and washed with DCM (50 mL). Compound **2c-Anth** was obtained as a red-brown solid (2.85 g, 5.31 mmol, 90%). **Mp**: 371 °C (decomp.) **IR** $\tilde{\nu}$ [cm^{-1}] = 1612, 1456, 1428, 1284, 1167, 1010, 961, 908, 855, 749, 682, 550, 474, 464, 417 **^{1}H NMR** (600 MHz, toluene-d_8): δ (in ppm) = 8.24 (s, 2H), 8.07 (s, 2H), 7.95 (s, 2H), 7.84 (s, 2H), 7.71–7.70 (m, 4H), 7.23–7.21 (m, 4H). **MALDI HR-MS** (pos): *m/z* calcd for $C_{30}H_{16}Br_2^+$ [M]$^+$ 533.9613, found 533.9622. *Due to very low solubility, it was not possible to measure a reasonably resolved $^{13}C\{^1H\}$ NMR spectrum.*

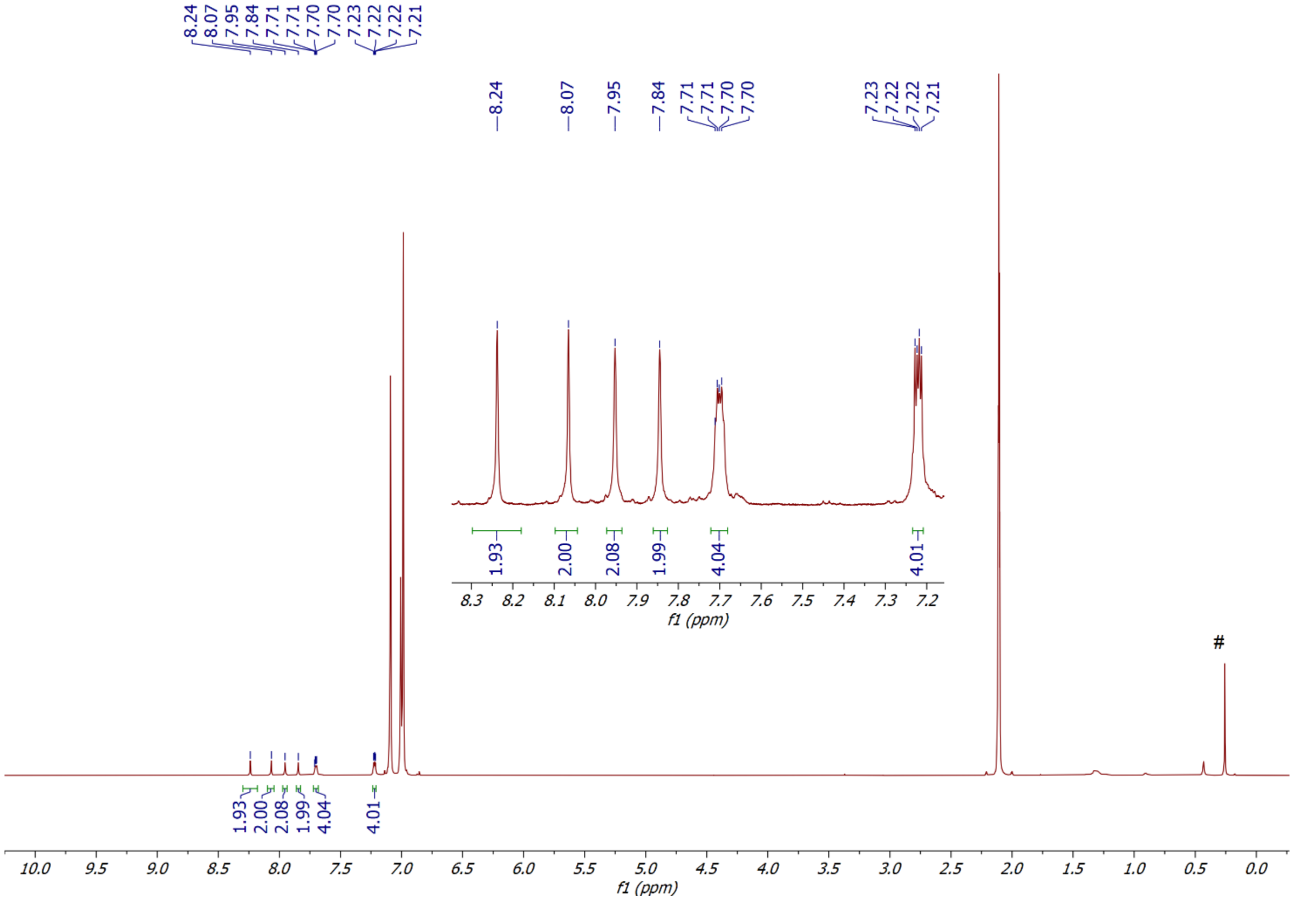

Figure S59. **^{1}H NMR** (600 MHz, Tol-d_8, 353 K) spectrum of **2c-Anth** (traces of silicon grease (#) are marked).

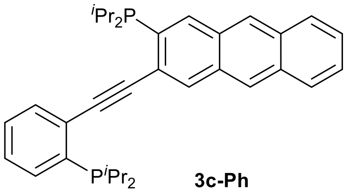

3c-Ph

2c-Ph (600 mg, 1.38 mmol, 1.0 eq.) was dissolved in a mixture of dry Et_2O and dry THF (10:1) under argon atmosphere. The solution was cooled to –40 °C and a 2.5 M solution of *n*-BuLi in hexanes (1.16 mL, 2.89 mmol, 2.1 eq.) was added via cannula over 5 min. After stirring the reaction mixture at –20 °C for 2 h, the clear brown solution was cooled to –40 °C and ^{i}Pr$_2$PCl (441 mg, 460 μL, 2.89 mmol, 2.1 eq.) was added. The reaction mixture was stirred in a cryostat at –5 °C for 20 h. The solvent was removed under reduced pressure and the flask was imported into the glovebox. The dark green solid was dissolved in toluene and filtrated over silica. Removing the solvent under reduced pressure afforded **3c-Ph** as a dark green-brown oil (586 mg, 1.15 mmol, 83%). Due to high air sensitivity and temperature instability no further purification for **3c-Ph** was performed. The formation of **3c-Ph** was oberved via **^{31}P{^{1}H} NMR spectroscopy** from the sample of the reaction solution. **^{31}P{^{1}H} NMR** (243 MHz, Et_2O/ THF): δ(in ppm) = 9.8 (s, 1P), 3.4 (s, 1P).

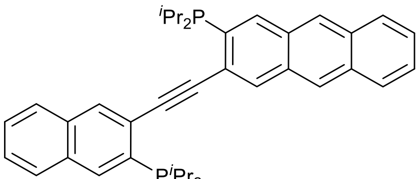

3c-Naph

2c-Naph (964 mg, 1.98 mmol, 1.0 eq.) was dissolved in a mixture of dry Et_2O and dry THF (10:1, 110 mL) under argon atmosphere. A 2.5 M solution of *n*-BuLi in hexanes (1.67 ml, 4.16 mmol, 2.1 eq.) was added dropwise at –40 °C. After stirring the reaction mixture at –10 °C for 2 h, the solution was cooled to –40 °C and ^{i}Pr$_2$PCl (635 mg, 663 μl, 4.16 mmol, 2.1 eq.) was added dropwise. The mixture was stirred in a cryostat at –5 °C for 87 h. The reaction solution was filtered over a silica plug. The filtrate was evaporated under vacuum and the precipitate was washed with HDMSO, and compound **3c-Naph** obtained as a dark brown oil (800 mg, 72%). Due to high air sensitivity and temperature instability no further purification for **3c-Naph** was performed. The formation of **3c-Naph** was oberved via **^{31}P{^{1}H} NMR spectroscopy** from a sample of the reaction solution. **^{31}P{^{1}H} NMR** (162 MHz, THF-*d$_8$*): δ(in ppm) = 9.2 (s), 7.8 (s). LIFDI (pos): *m/z* calcd for $C_{38}H_{42}P_2^+$ [M]$^+$ 560.2756, found 560.3157.

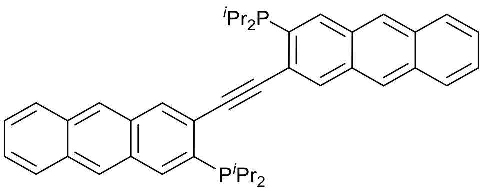

3c-Anth

2c-Anth (1.00 g, 1.86 mmol, 1.0 eq.) was dissolved in a mixture of dry Et_2O and dry THF (10:1, 110 mL). A 2.5 M solution of *n*-BuLi in hexanes (1.57 ml, 3.92 mmol, 2.1 eq.) was added dropwise at –40 °C. After stirring the reaction mixture at 0 °C for 2 h, the solution was cooled to –40 °C and ^{i}Pr$_2$PCl (598 mg, 623 μl, 3.92 mmol, 2.1 eq) was added dropwise. The mixture was stirred in a cryostat at –5 °C for 92 h. The solvent was removed under reduced pressure and the flask was imported into the glovebox. The residue was dissolved in toluene and filtrated over silica. The filtrate was evaporated under vacuum and the precipitate was washed with HDMSO and **3c-Anth** was obtained as a dark green-brown solid (0.66 mmol, 406 mg, 35%). %). Due to high air sensitivity and temperature instability no further purification for **3c-Anth** was performed. The formation of **3c-Anth** was oberved via **^{31}P{^{1}H} NMR spectroscopy** from a sample of the reaction solution. **^{31}P{^{1}H} NMR** (162 MHz, toluene-*d$_8$*): δ(in ppm) = 7.6 (s). **LIFDI** (pos): *m/z* calcd for $C_{42}H_{44}P_2^+$ [M]$^+$ 610.2913, found 610.3403.

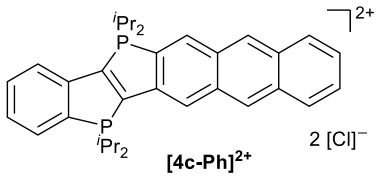

[4c-Ph]$^{2+}$

3c-Ph (702 mg, 1.37 mmol, 1.0 eq.) was dissolved in deoxygenated and dry DCM (10 mL) inside an argon filled glovebox and hexachloroethane (340 mg, 1.44 mmol, 1.1 eq.) was added. The dark clear red solution was stirred at rt for 1 h. The solvent was removed under reduced pressure and the red solid was washed with Et_2O and pentane. Recrystallization from MeOH/Et_2O afforded **[4c-Ph]$^{2+}$** as a red solid (564 mg, 972 μmol, 71%). **Mp:** 253 °C **IR** $\tilde{\nu}$ [cm^{-1}] = 3390, 2967, 2834, 1624, 1540, 1436, 1370, 1341, 1262, 1227, 1125, 1038, 934, 877, 830, 790, 774, 749, 695, 670, 665, 645, 545, 457, 437, 413 **^{1}H {^{31}P} NMR** (600 MHz, CD_2Cl_2): δ (in ppm) = 10.23 (s, 1H), 9.22 (s, 1H), 9.16 (s, 1H), 9.00 (d, $J_{H\text{-}H}$ = 7.6 Hz, 1H), 8.80 (s, 1H), 8.23–8.11 (m, 4H), 7.83 (t, $J_{H\text{-}H}$ = 7.5 Hz, 1H), 7.70–7.68 (m, 2H), 5.12–5.10 (m, 2H), 4.89–4.87 (m, 2H), 1.69–165 (m, 12H), 1.30–1.23 (m, 12H). **^{31}P {^{1}H} NMR** (243 MHz, CD_2Cl_2): δ (in ppm) = 56.5 (d, $J_{P\text{-}P}$ = 24.0 Hz), 54.6 (d, $J_{P\text{-}P}$ = 23.9 Hz). **^{13}C {^{1}H} NMR** (151 MHz, CD_2Cl_2): δ (in ppm) = 148.5 (dd, $J_{C\text{-}P}$ = 64.7, 9.3 Hz, C_q, 1C), 143.6 (dd, $J_{C\text{-}P}$ = 65.9, 11.5 Hz, C_q, 1C), 140.7 (d, $J_{C\text{-}P}$ = 9.5 Hz, CH, 1C), 140.2 (dd, $J_{C\text{-}P}$ = 13.3, 11.0 Hz, C_q, 1C), 137.9 (d, $J_{C\text{-}P}$ = 2.0 Hz, CH, 1C), 134.7 (C_q, 1C), 134.5 (d, $J_{C\text{-}P}$ = 9.7 Hz, CH, 1C), 134.1 (d, $J_{C\text{-}P}$ = 1.2 Hz, C_q, 1C), 132.7 (d, $J_{C\text{-}P}$ = 5.4 Hz, CH, 1C), 132.4 (C_q, 1C), 132.0 (d, $J_{C\text{-}P}$ = 10.5 Hz, CH, 1C), 131.8 (CH, 1C), 131.5 (dd, $J_{C\text{-}P}$ = 14.9, 11.5 Hz, C_q, 1C), 130.7 (CH, 1C), 129.9 (d, $J_{C\text{-}P}$ = 12.7 Hz, C_q, 1C), 129.5 (CH, 1C), 129.3 (d, $J_{C\text{-}P}$ = 6.6 Hz, CH, 1C), 129.2 (CH, 1C), 128.8 (CH, 1C), 128.7 (CH, 1C), 119.9 (dd, $J_{C\text{-}P}$ = 78.2, 8.7 Hz, C_q, 1C), 114.8 (dd, $J_{C\text{-}P}$ = 83.3, 9.0 Hz, C_q, 1C), 24.4 (dd, $J_{C\text{-}P}$ = 51.8, 49.6 Hz, CH, 4C), 17.5 (dd, $J_{C\text{-}P}$ = 35.0, 3.3 Hz, CH_3, 4C), 17.0 (dd, $J_{C\text{-}P}$ = 34.0, 2.7 Hz, CH_3, 4C). **ESI HR-MS** (pos): *m/z* calcd for $C_{34}H_{39}P_2$ [M-H]$^+$ 509.2522, found 509.2521; calcd for $C_{34}H_{41}P_2O$ [M+OH]$^+$ 527.2627, found 527.2627.

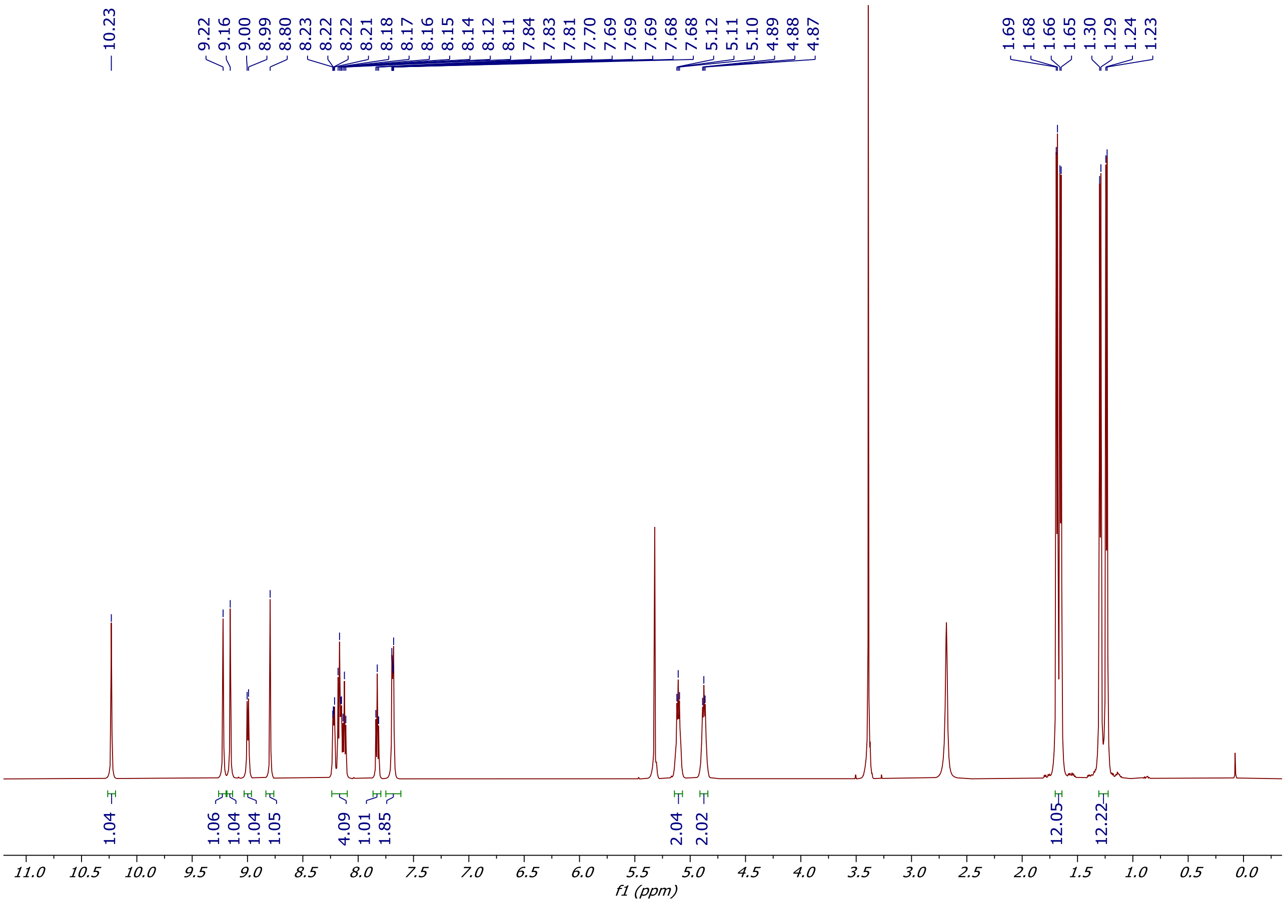

Figure S60. **^{1}H {^{31}P} NMR** (600 MHz, CD_2Cl_2, 298k) spectrum of **[4c-Ph]$^{2+}$**.

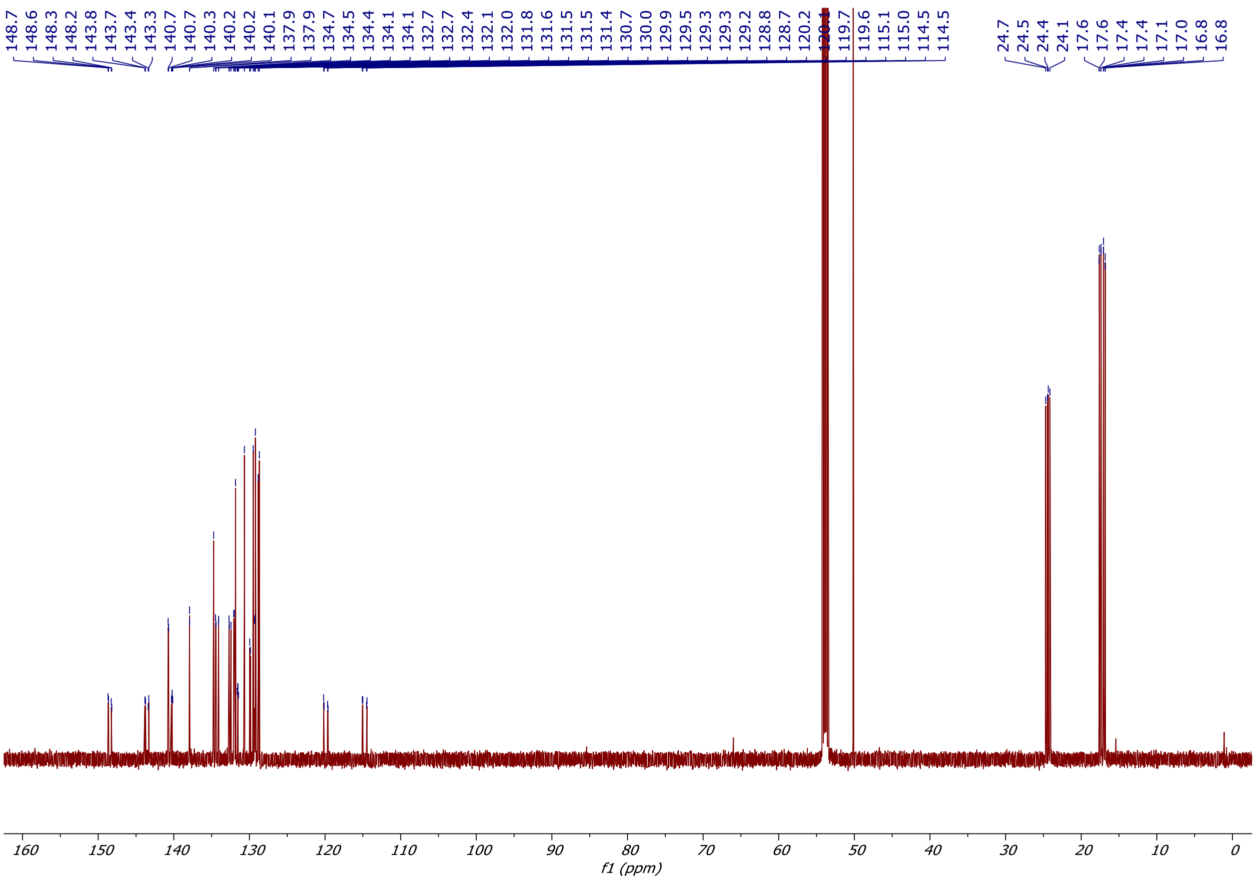

Figure S61. **^{13}C {^{1}H} NMR** (151 MHz, CD_2Cl_2, 298K) spectrum of **[4c-Ph]$^{2+}$**.

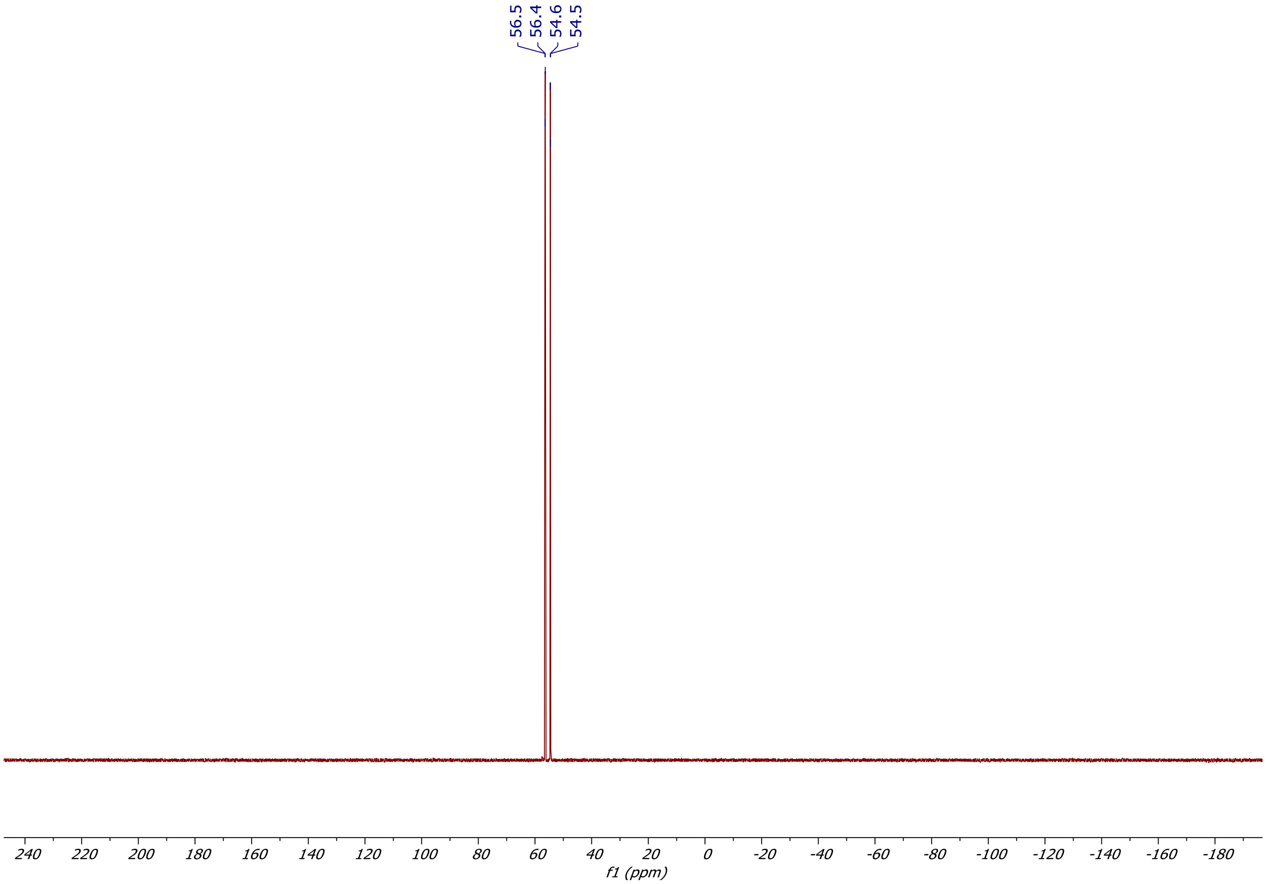

Figure S62. **^{31}P {^{1}H} NMR** (243 MHz, CD_2Cl_2, 298K) spectrum of **[4c-Ph]$^{2+}$**.

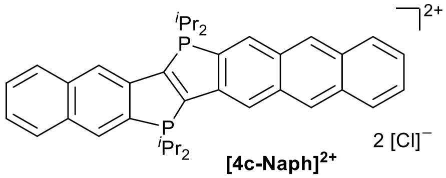

3c-Naph (710 mg, 1.27 mmol) and C_2Cl_6 (312 mg, 1.32 mmol) were stirred inside an argon filled glovebox in dry DCM (5 mL) at rt for 2 h. The solvent was removed under reduced pressure and the red solid was washed with Et_2O and pentane. Recrystallization from MeOH/Et_2O afforded **[4c-Naph]$^{2+}$** as a red solid (121 mg, 187 μmol, 15%). **Mp:** 285 °C (decomp.) **IR** $\tilde{\nu}$ [cm^{-1}] = 3373, 2966, 2929, 2867, 1617, 1540, 1457, 1277, 1220, 1169, 1037, 932, 914, 877, 829, 789, 751, 706, 663, 538, 474 **^{1}H NMR {^{31}P}** (400 MHz, CD_3OD): δ (in ppm) = 9.22 (s, 1H), 9.04 (s, 1H), 8.96 (s, 1H), 8.94 (s, 1H), 8.84 (s, 1H), 8.69 (s, 1H), 8.35–8.26 (m, 4H), 7.99–7.91 (m, 2H), 7.82–7.77 (m, 2H), 4.06–3.97 (m, 4H), 1.63–1.60 (m, 12H), 1.51–1.48 (m, 12H). **^{31}P NMR {^{1}H}** (162 MHz, CD_3OD): δ (in ppm) = 56.2 (d, J_{P-P} = 23.5), 55.2 (d, J_{P-P} = 23.5). **^{13}C NMR {^{31}P}** (151 MHz, CD_3OD): δ (in ppm) = 146.8 (C_q, 1C), 145.6 (C_q, 1C), 142.0 (CH, 1C), 139.7 (CH, 1C), 137.5 (C_q, 1C), 136.0 (C_q, 1C), 135.3 (C_q, 1C), 135.1 (C_q, 1C), 134.7 (C_q, 1C), 133.4 (C_q, 1C), 133.0 (CH, 1C), 133.0 (C_q, 1C), 132.0 (CH, 1C), 131.8 (CH, 1C), 131.6 (CH, 1C), 131.4 (CH, 1C), 131.2 (CH, 1C), 131.0 (C_q, 1C), 130.8 (CH, 1C), 130.2 (CH, 1C), 130.1 (CH, 1C), 129.9 (CH, 1C), 129.8 (CH, 1C), 129.4 (CH, 1C), 117.0 (C_q, 1C), 115.7 (C_q, 1C), 24.8 (CH, 2C), 24.6 (CH, 2C), 16.9 (CH_3, 2C), 16.8 (CH_3, 2C), 16.0 (CH_3, 4C). **ESI HR-MS** (pos): *m/z* calcd for $C_{38}H_{43}P_2^+$ [M+H]$^+$ 561.2835, found 561.2839; $C_{38}H_{43}OP_2^+$ [M+OH]$^+$ 577.2784, found 577.2786.

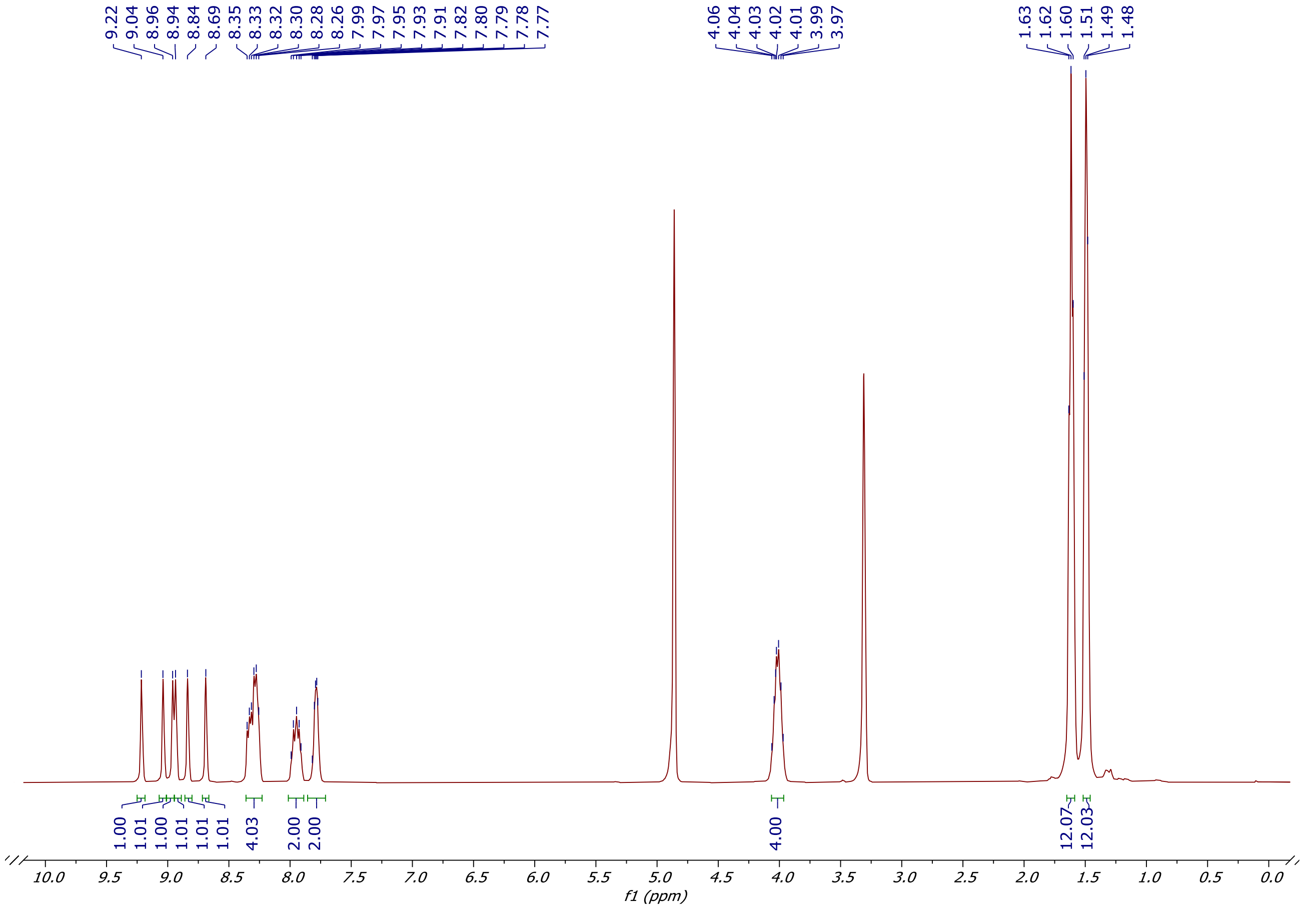

Figure S63. **^{1}H {^{31}P} NMR** (400 MHz, CD_3OD, 298K) spectrum of **[4c-Naph]$^{2+}$**.

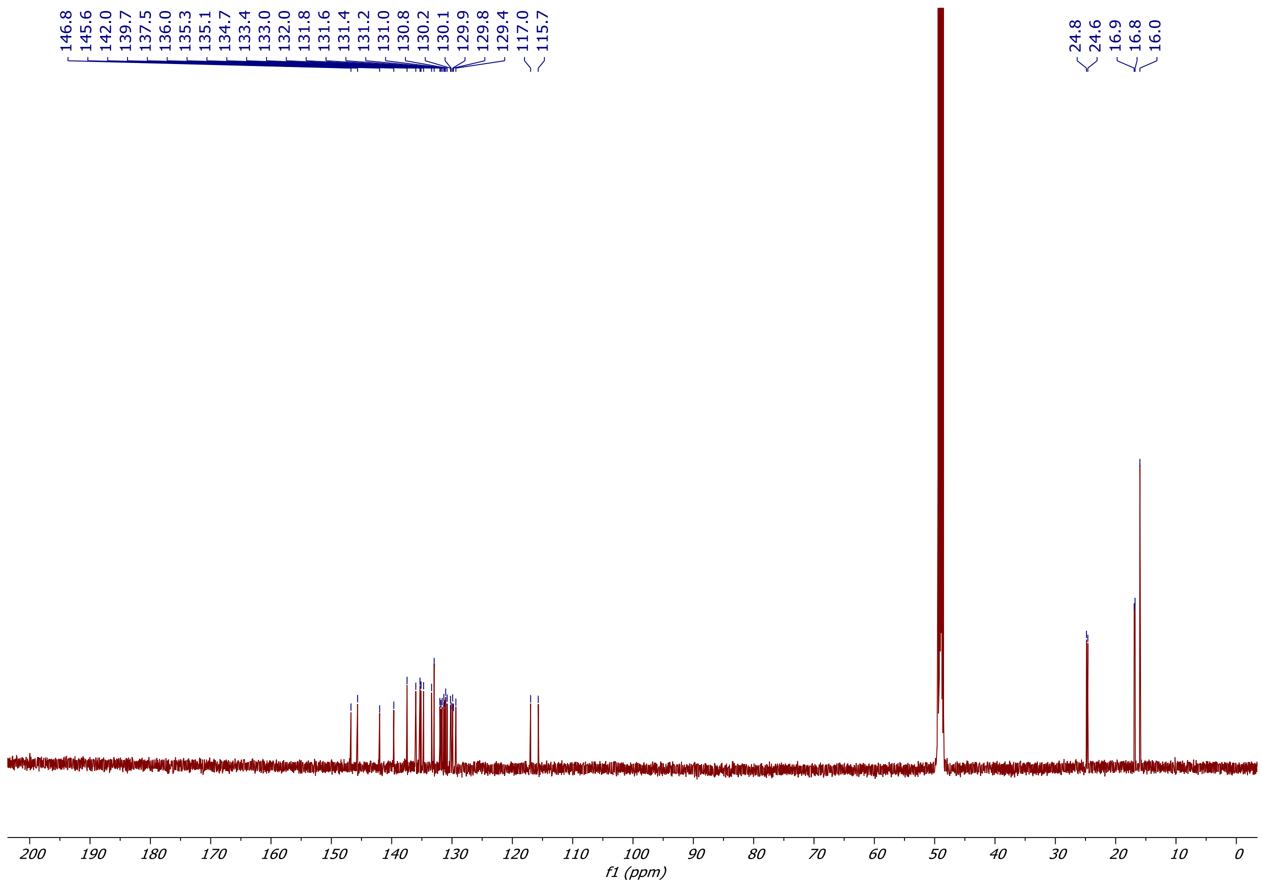

Figure S64. **^{13}C {^{31}P} NMR** (151 MHz, CD_3OD, 298K) spectrum of **[4c-Naph]$^{2+}$**.

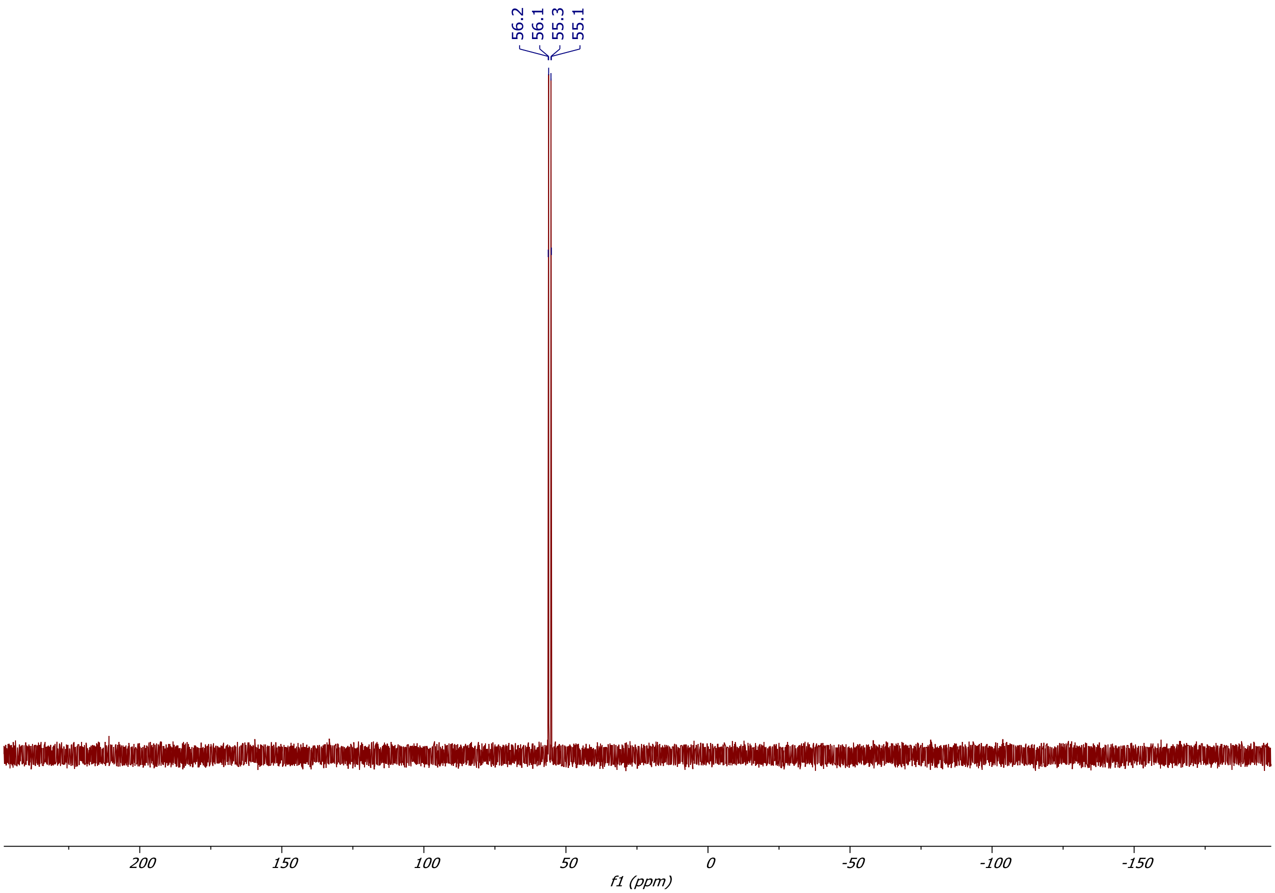

Figure S65. **^{31}P {^{1}H} NMR** (162 MHz, CD_3OD, 298K) spectrum of **[4c-Naph]$^{2+}$**.

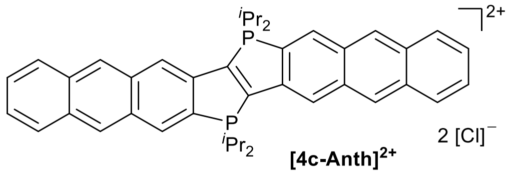

[4c-Anth]$^{2+}$

3c-Anth (406 mg, 665 μmol, 1.0 eq.) and C_2Cl_6 (164 mg, 691 μmol, 1.0 eq.) were stirred in dry DCM (5 mL) at rt for 90 min. The solvent was removed under reduced pressure and the dark red solid was washed with Et_2O and pentane. Recrystallization from MeOH/Et_2O afforded **[4c-Anth]$^{2+}$** as a dark red solid (94.0 mg, 138 μmol, 21%). **Mp:** 306 °C **IR** $\tilde{\upsilon}$ [cm^{-1}] = 3375, 2977, 2846, 2331, 2165, 2130, 2047, 2025, 1980, 1933, 1627, 1565, 1542, 1440, 1340, 1280, 1225, 1043, 897, 830, 753, 699, 659, 579, 542, 479, 462, 448, 406 **^{1}H NMR {^{31}P}** (400 MHz, CD_3OD): δ (in ppm) = 9.21 (s, 2H), 8.94 (d, $J_{H–H}$ = 3.9 Hz, 4H), 8.81 (s, 2H), 8.27 (t, $J_{H–H}$ = 8.5 Hz, 4H), 7.79 (td, $J_{H–H}$ = 6.4, 3.4 Hz, 4H), 4.05–3.97 (m, 4H), 1.64 (d, $J_{H–H}$ = 6.9 Hz, 12H), 1.53 (d, $J_{H–H}$ = 6.9 Hz, 12H). **^{31}P NMR {^{1}H}** (162 MHz, CD_3OD): δ (in ppm) = 53.2 (s). **^{13}C NMR {^{1}H, ^{31}P}** (151 MHz, CD_3OD): δ (in ppm) = 145.1 (C_q, 2C), 141.5 (CH, 2C), 135.6 (C_q, 2C), 134.9 (C_q, 2C), 133.2 (C_q, 2C), 132.6 (C_q, 2C), 131.6 (CH, 2C), 131.1 (CH, 2C), 130.6 (C_q, 2C), 130.1 (CH, 2C), 129.8 (CH, 2C), 129.7 (CH, 2C), 129.5 (CH, 2C), 129.3 (CH, 2C), 115.4 (C_q, 2C), 24.5 (CH, 4C), 16.5 (CH_3, 4C), 15.6 (CH_3, 4C). **ESI HR-MS** (pos): *m/z* calcd for $C_{42}H_{45}OP_2^+$ [M+OH]$^+$ 627.2940, found 627.2941.

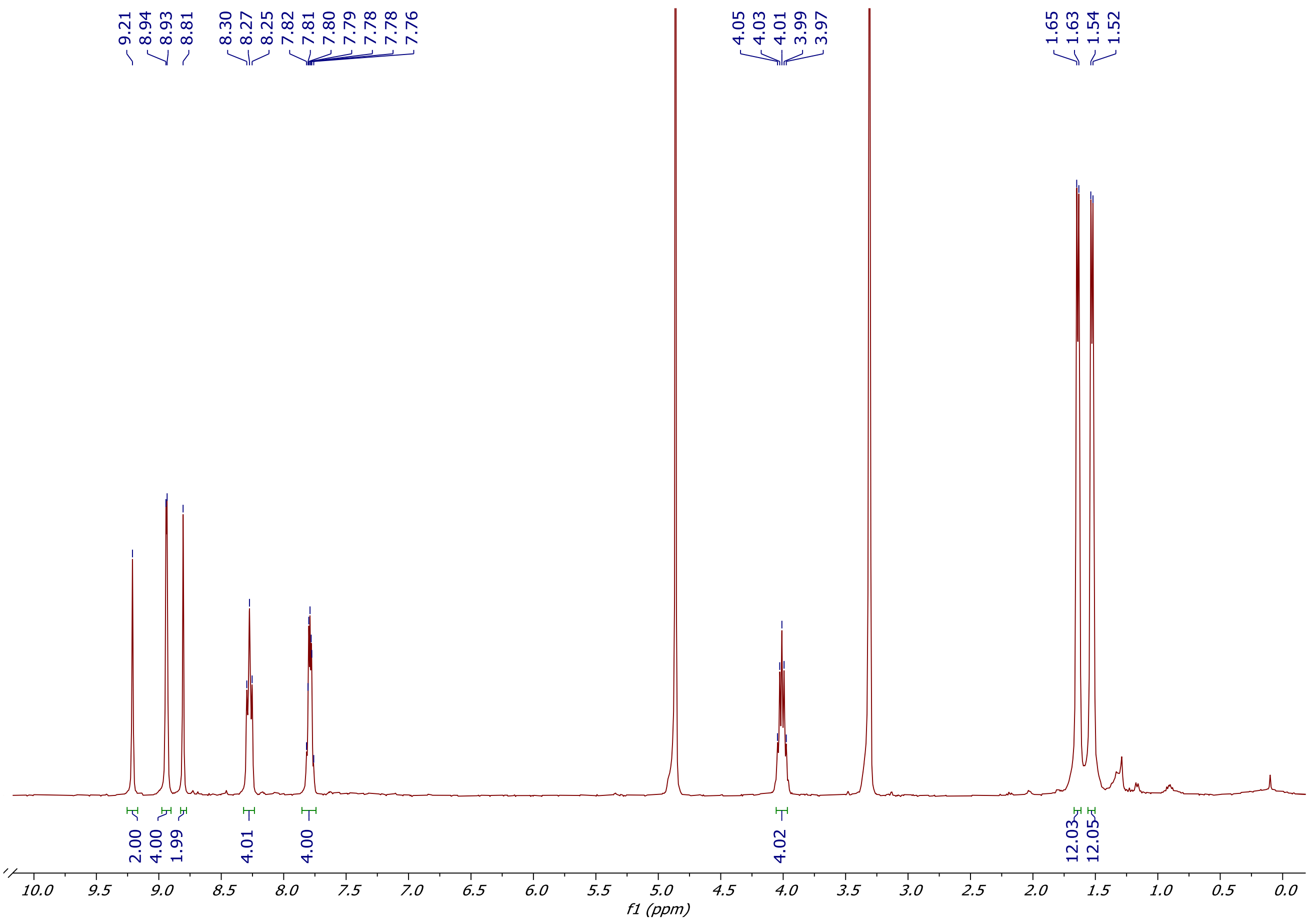

Figure S66. **^{1}H {^{31}P} NMR** (400 MHz, CD_3OD, 298K) spectrum of **[4c-Anth]$^{2+}$**.

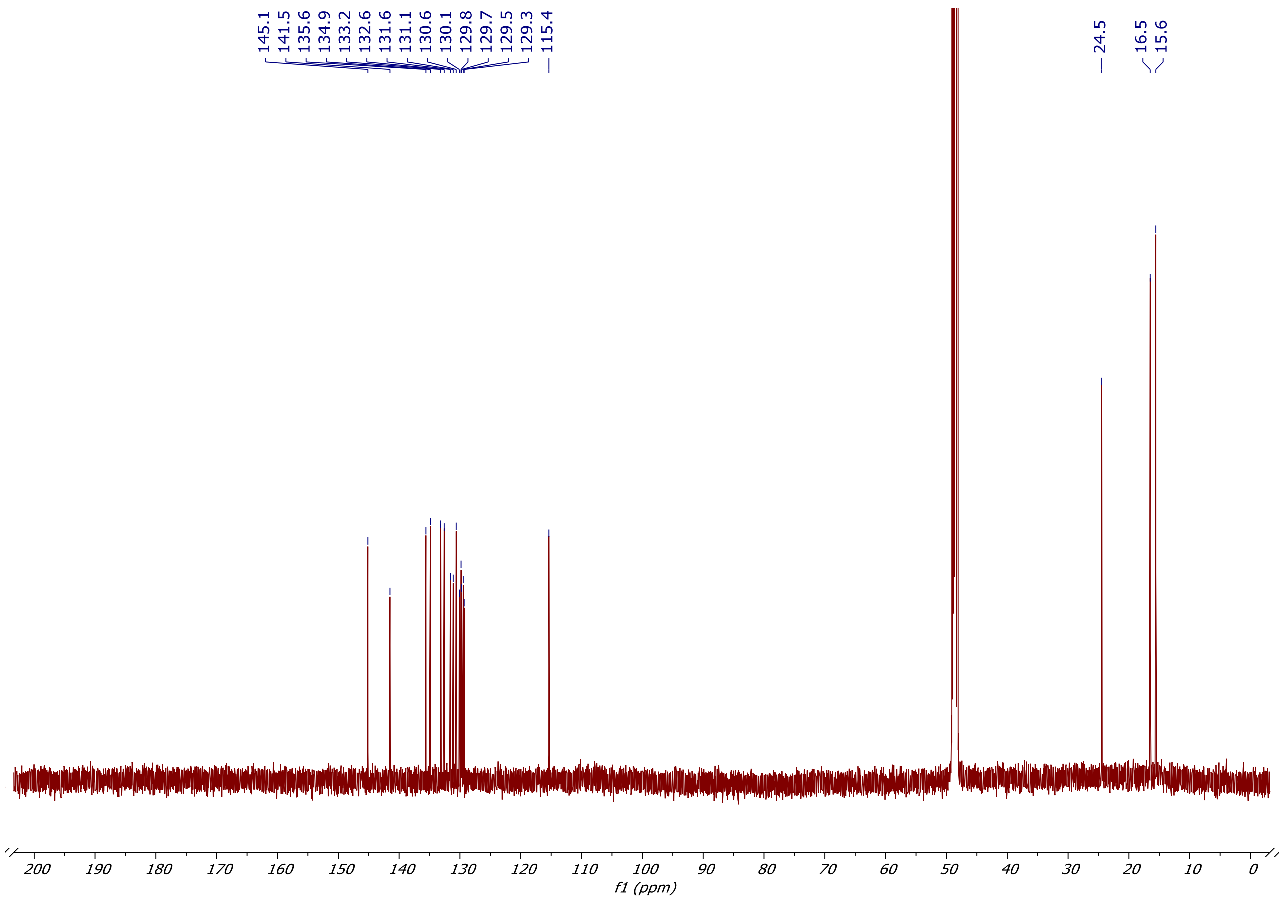

Figure S67. **^{13}C {^{1}H, ^{31}P} NMR** (151 MHz, CD_3OD, 298K) spectrum of **[4c-Anth]$^{2+}$**.

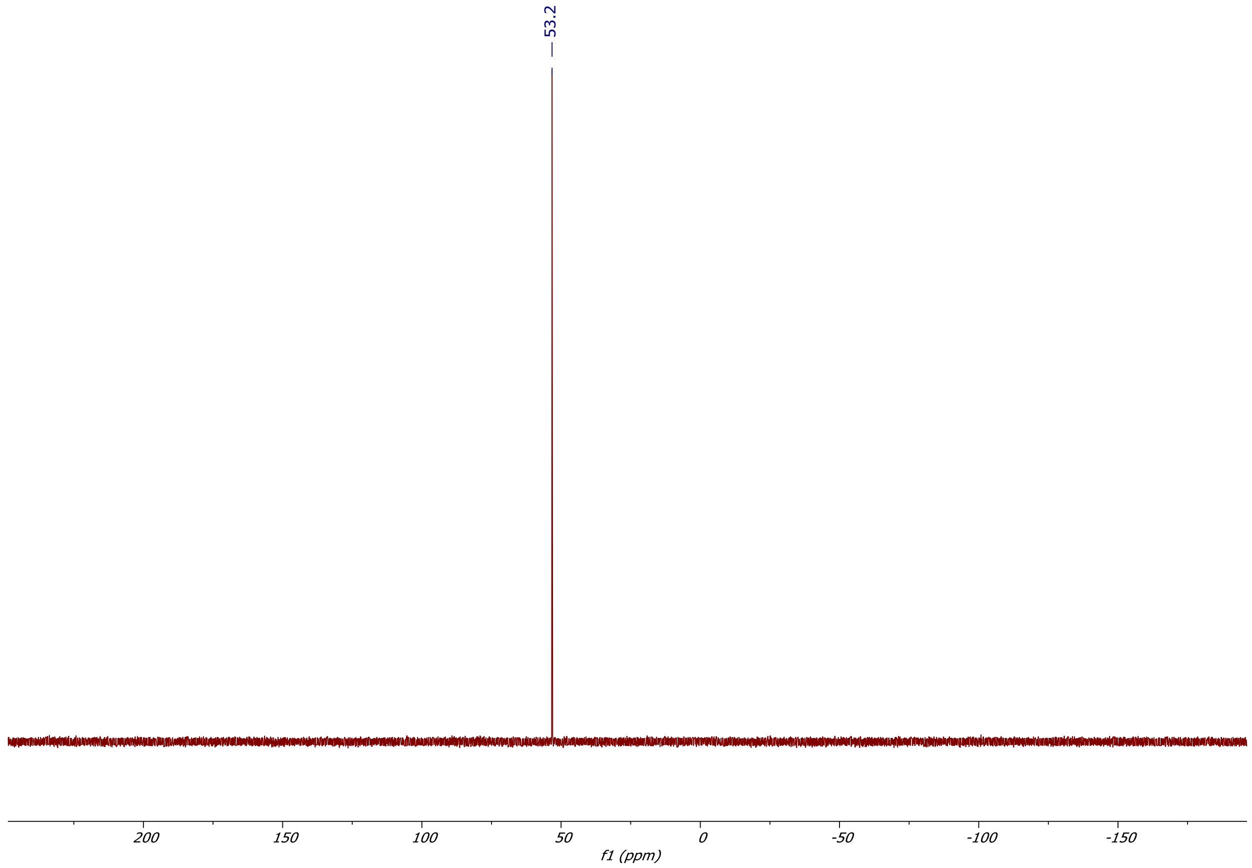

Figure S68. **^{31}P {^{1}H} NMR** (162 MHz, CD_3OD, 298K) spectrum of **[4c-Anth]$^{2+}$**.

2) Optical spectroscopy

2.1) Chemicals

The emissive diphosphonium compounds were obtained and purified as chloride salts (see section 1). The chemicals that were used as reference compounds were used as obtained from the manufacturer without further purification (fluorescein, Riedel-De Haen, p. a. quality), sodium hydroxide (NaOH, Sigma-Aldrich, ≥98%), tris(2,2'-bipyridyl) dichlororuthenium(II) hexahydrate ($[Ru(bpy)_3]^{2+}$, Sigma-Aldrich, 99.95%).

2.2) Sample preparation

The solutions for all spectroscopic measurements were prepared using ultrapure Millipore MilliQ water (specific resistance 18.2 MΩcm), MeCN (HPLC grade, VWR), MeOH (>99.8%, HPLC grade, Fisher Chemical), CH_2Cl_2 (DCM, MQ 200 grade, Sigma-Aldrich). All solutions were sonicated for 5 min and transfered to quartz glass cuvettes with 1 cm path length. For steady-state and time-resolved emission measurements, the solutions were purged with argon (Nippon Gases 5.0) for 10 min before the measurement and sealed under inert gas.

2.3) Steady-state measurements

Absorption spectra were recorded with a LAMBDA 356 from Perkin Elmer. Emission spectra were measured using a FL 6500 from Perkin Elmer using the built in xenon lamp for excitation and the PMT R928 for detection. The emission spectra for **$[4c\text{-}Ph]^{2+}$**, **$[4c\text{-}Naph]^{2+}$** and **$[4c\text{-}Anth]^{2+}$** (Figures S74-S76) and therefore Figure 3 in the main paper were measured with a FS5 from Edingburgh Instruments using the built in xenon lamp for excitation and the PMT R13456 for detection (which has a higher sensitivity in the red spectral region). For the lifetime measurements (Table S5, Table S6, Table S7) an EPL-510 (pulse-width 141.9 ps, average power 5 mW, λ_{exc} = 505 nm) was used as excitation source. All steady-state absorption and emission measurements were performed at room temperature (295 ± 2 K) and the emission spectra were corrected for the wavelength-dependent sensitivity of the emission spectrometer. The E_{00} energies were determined from the intersections of the absorption and emission spectra. The fluorescence quantum yields were referenced against known standard compounds (Section S 3.2.). To avoid filter effects, the absorbance was adjusted below 0.1 at the excitation wavelengths and in the overlap area of emission and absorption spectra. The quantum yield was then determined according to Equation S 1 with Φ … quantum yield, I … integrated luminescence intensity, A … optical density at the excitation wavelength, and η … refractive indices of the used solvents. All variables with the index *ref* correspond to the reference compounds.[17]

$$\Phi = \Phi_{\mathrm{ref}} \times \frac{I}{I_{\mathrm{ref}}} \times \frac{A_{\mathrm{ref}}}{A} \times \frac{\eta^2}{\eta_{\mathrm{ref}}^2}$$

Equation S 1

2.4) Time-resolved measurements

The fluorescence lifetimes in H_2O were recorded with a mini-τ spectrometer from Edinburgh Instruments (time-correlated single photon counting TCSPC technique), with a pulsed LED (EPLED-300, pulse-width 1020.0 ps, average power 40 µW, λ_{exc} = 293 nm) or different lasers (EPL-375, pulse-width 58.3 ps, average power 5 mW, λ_{exc} = 371 nm; EPL-450, pulse-width 74.5 ps, average

power 5 mW, λ_{exc} = 446 nm; EPL-510, pulse-width 141.9 ps, average power 5 mW, λ_{exc} = 505 nm) for excitation and the built-in band-pass filters in the detection pathway. The concentrations of all solutions were adjusted such that the optical density at the excitation wavelength was between 0.1 and 0.2.

3) Steady-state absorption and emission measurements

3.1) Molar absorption coefficients and E_{00} energies

The molar absorption coefficients of all chromophores in water were determined in Lambert-Beer plots using 4-6 different concentrations for the absorption spectra and analyzed at three different wavelengths (highlighted in blue, red or green in the corresponding spectra Figure S69 and Figure S70). For the fitting, the linear fit tool of the program Origin was used with the intercept being zero. The values for the molar absorption coefficients obtained from the slopes are given in Table 1 of the main part and have been rounded to the nearest hundred and the respective errors from the linear fits are displayed in Table S1. The absorption spectra did not show any noticeable differences after normalization at the chosen concentrations, indicating the absence of aggregation or decomposition phenomena under the experimental conditions.

Table S1. List for molar absorption coefficients and the errors from the fitting at three different wavelengths for each compound in water. For corresponding absorption spectra see Figure S69 and Figure S70.

**Compound**	**1. ε /10^3 M^{-1}s^{-1} (λ / nm)**	**2. ε /10^3 M^{-1}s^{-1} (λ / nm)**	**3. ε /10^3 M^{-1}s^{-1} (λ / nm)**
**[4a-Ph]$^{2+}$**	41.6 ± 0.3 (265)	8.6 ± 0.0 (305)	11.8 ± 0.1 (426)
**[4a]$^{2+}$**	42.1 ± 1.1 (285)	19.3 ± 0.5 (310)	19.4 ± 0.6 (435)
**[4b-Ph]$^{2+}$**	20.9 ± 0.7 (270)	7.3 ± 0.2 (408)	4.9 ± 0.1 (450)
**[4b]$^{2+}$**	28.6 ± 0.0 (298)	8.2 ± 0.2 (408)	6.9 ± 0.1 (484)
**[4c-Ph]$^{2+}$**	37.9 ± 0.2 (269)	10.1 ± 0.1 (389)	9.6 ± 0.0 (489)
**[4c-Napth]$^{2+}$**	24.4 ± 0.3 (322)	12.0 ± 0.1 (410)	12.5 ± 0.2 (479)
**[4c-Anth]$^{2+}$**	53.2 ± 0.4 (336)	17.2 ± 0.3 (418)	22.3 ± 0.4 (498)

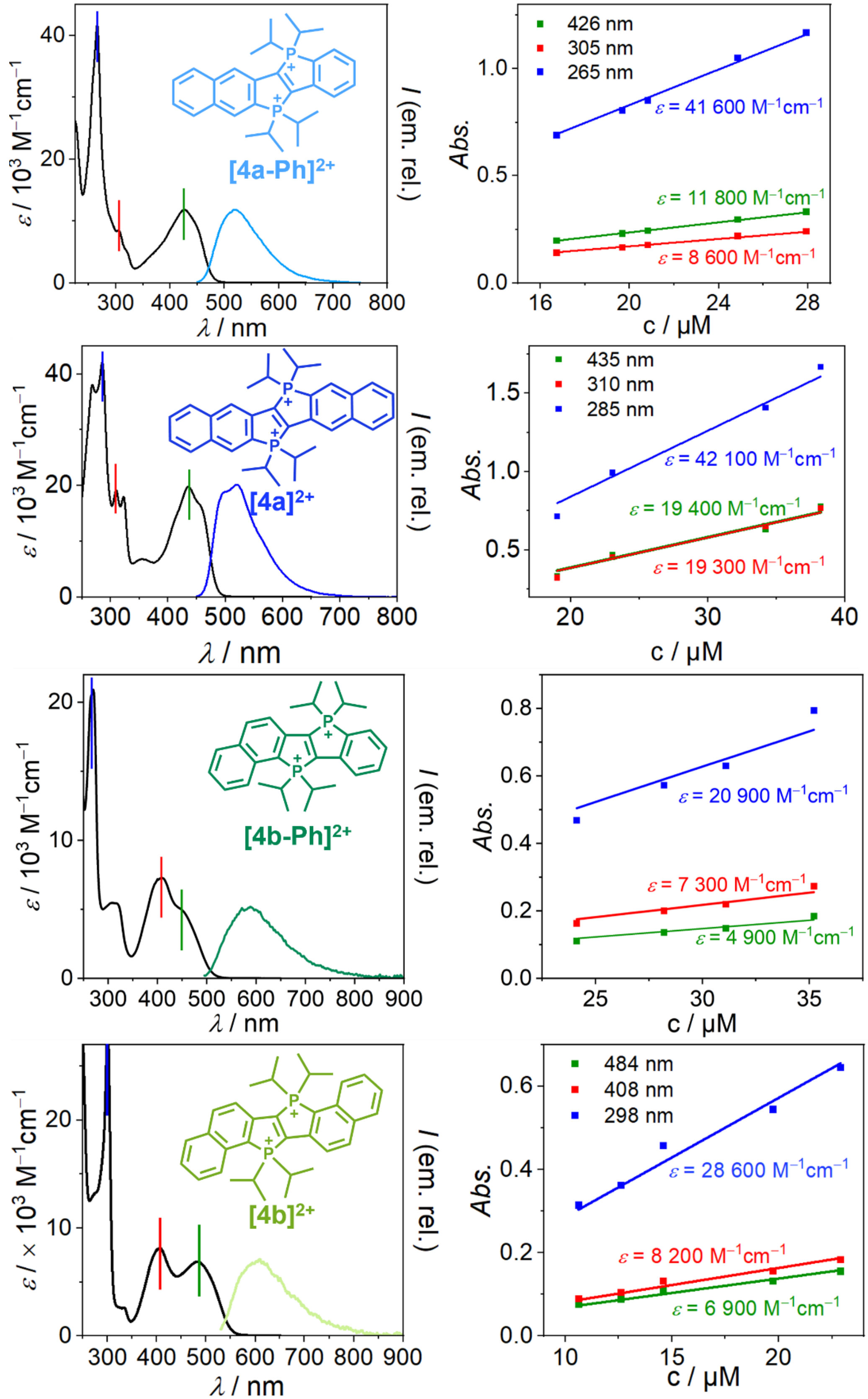

Figure S69. Molar absorption coefficients and E_{00} of compounds [**4a-Ph**]$^{2+}$, [**4a**]$^{2+}$, [**4b-Ph**]$^{2+}$ and [**4b**]$^{2+}$. Left side: Chemical structure of the chromophores, the corresponding molar absorption coefficients and emission spectrum (intersection was used for E_{00} value determination). Right side: Lambert-Beer plots of the chromophores at three different wavelengths (color coded), and their respective molar absorption coefficients.

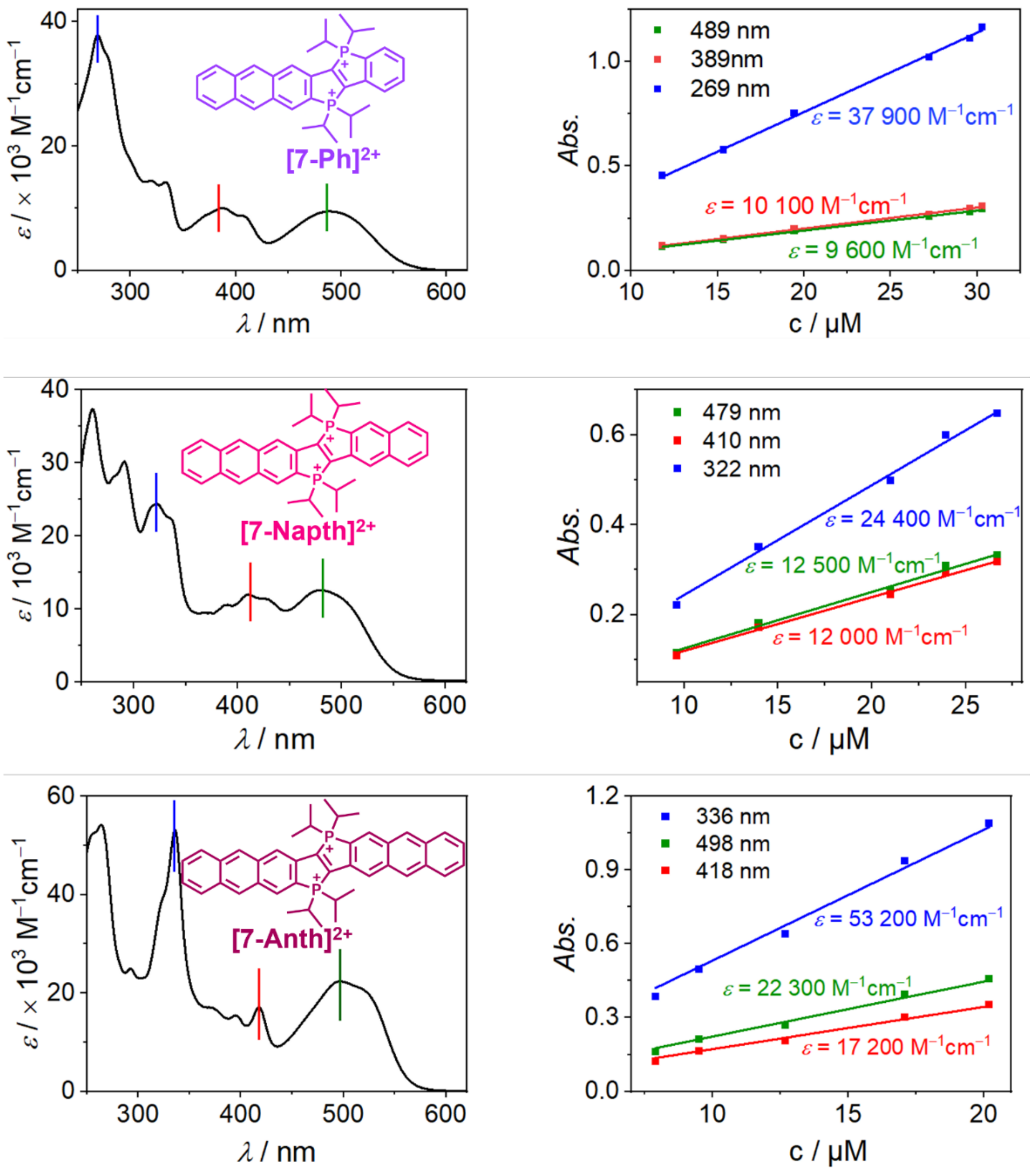

Figure S70. Molar absorption coefficients and E_{00} of compounds [**4c-Ph**]$^{2+}$, [**4c-Naph**]$^{2+}$ and [**4c-Anth**]$^{2+}$. Left side: Chemical structure of the chromophores, the corresponding molar absorption coefficients (emission spectra and E_{00} determination in Figures S74 – S76 and Tables S5 – S7). Right side: Lambert-Beer plots of the chromophores at three different wavelengths (color coded), and their respective molar absorption coefficients.

3.2) Emission quantum yields

The quantum yields of the organic chromophores were measured against well-established reference compounds. The average of 2 or 3 measurements at different excitation wavelengths was used as quantum yield. For each measurement the concentration was adjusted such that the optical density is ≤ 0.1 at the chosen excitation wavelength and the overlap area of the absorption and emission spectrum. For clarity, spectra of the purged solutions were normalized to 1 for the highest emission intensity (while maintaining the relation of the intensities) and the resulting emission spectra are given in Figure S71, Figure S72 and Figure S73.

For **[4a-Ph]$^{2+}$** and **[4a]$^{2+}$**, Fluorescein (F^{2-}, in 0.1 M NaOH, Φ_{FL} = 0.88)[18] was chosen as a reference compound. Figure S71 shows the absorption spectra and the excitation wavelength for the emission spectra indicated at the abscissa together with the corresponding emission spectra. The individual quantum yields, and the resulting average values and standard deviations are given in Table S2.

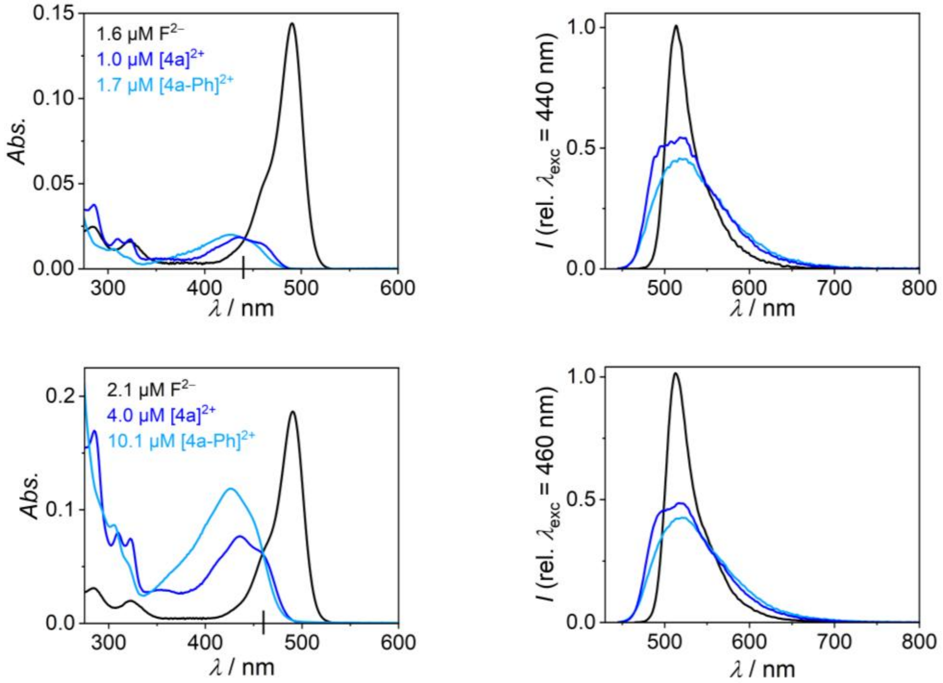

Figure S71. Absorption (upper and lower, left) and emission spectra (upper and lower, right) of Fluorescein (F^{2-}, black traces, in 0.1 M NaOH), [**4a-Ph**]$^{2+}$ (light blue, in H_2O) and [**4a**]$^{2+}$ (blue, in H_2O). The excitation wavelength of the emission spectra is indicated at the abscissa of the absorption spectra.

Table S2. Quantum yields (Φ_{FL}) of individual measurements using different excitation wavelengths (λ_{exc} = 440 or 460 nm), the averaged quantum yield (Φ_{AV}) and the standard deviation.

**Compound**	**Φ_{FL} (λ_{exc} = 440 nm)**	**Φ_{FL} (λ_{exc} = 460 nm)**	**Φ_{AV}** (stdev.)
**[4a-Ph]$^{2+}$**	0.79	0.83	0.81 (±0.020)
**[4a]$^{2+}$**	0.88	0.88	0.88 (±0.000)

For **[4b-Ph]$^{2+}$**, **[4c-Ph]$^{2+}$** and **[4c-Naph]$^{2+}$** $[Ru(bpy)_3]^{2+}$ (in H_2O, Φ_{FL} = 0.063)[18] was chosen as a reference compound. Figure S72 shows the absorption spectra and the excitation wavelength for the emission spectra indicated at the abscissa together with the corresponding emission spectra. The individual quantum yields, and the resulting average values and standard deviations are given in Table S3.

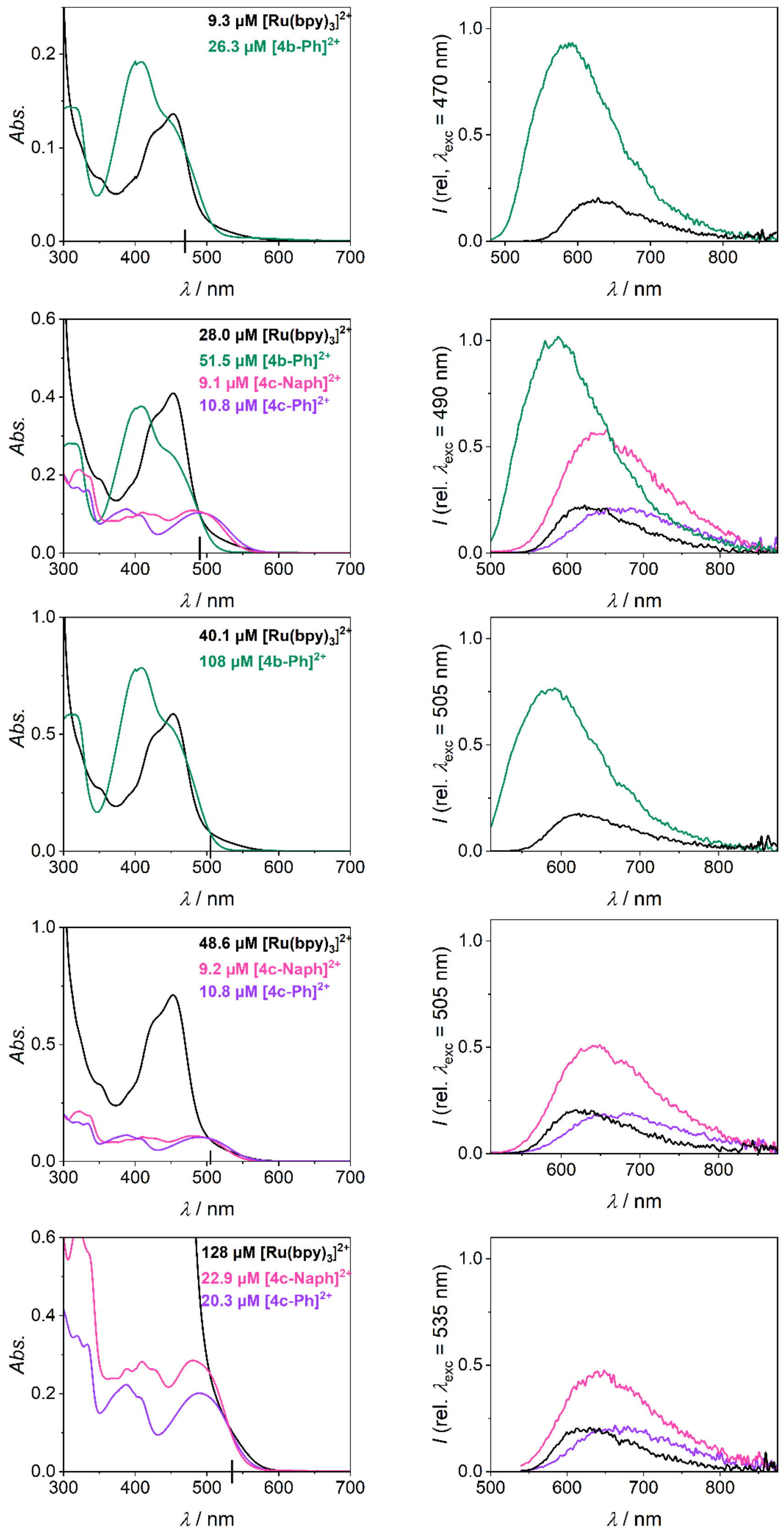

Figure S72. Absorption (left) and emission spectra (right) of $[Ru(bpy)_3]^{2+}$ (black in H_2O), **[4b-Ph]$^{2+}$** (dark green, in H_2O), **[4c-Ph]$^{2+}$** (violet, in H_2O) and **[4c-Naph]$^{2+}$** (pink, in H_2O). The excitation wavelength of the emission spectra is indicated at the abscissa of the absorption spectra.

Table S3. Quantum yields (Φ_{FL}) of individual measurements using different excitation wavelengths (λ_{exc} = 470, 490, 505 or 535 nm), the averaged quantum yield (Φ_{AV}) and the standard deviation.

**Quantum yield**	**[4b-Ph]$^{2+}$**	**[4c-Naph]$^{2+}$**	**[4c-Ph]$^{2+}$**
$\Phi_{\Phi L}$ (λ_{exc} = 470 nm)	0.33	-	-
Φ_{FL} (λ_{exc} = 490 nm)	0.34	0.19	0.08
Φ_{FL} (λ_{exc} = 505 nm)	0.34	0.18	0.08
Φ_{FL} (λ_{exc} = 535 nm)	-	0.20	0.10
Φ_{AV} (stdev.)	0.34 (± 0.005)	0.19 (± 0.008)	0.09 (± 0.010)

For **[4b]$^{2+}$** and **[4c-Anth]$^{2+}$**, $[Ru(bpy)_3]^{2+}$ (in H_2O, Φ_{FL} = 0.063)[18] was again chosen as a reference compound. Figure S73 shows the absorption spectra and the excitation wavelength for the emission spectra indicated at the abscissa together with the corresponding emission spectra. The individual quantum yields, and the resulting average values and standard deviations are given in Table S4.

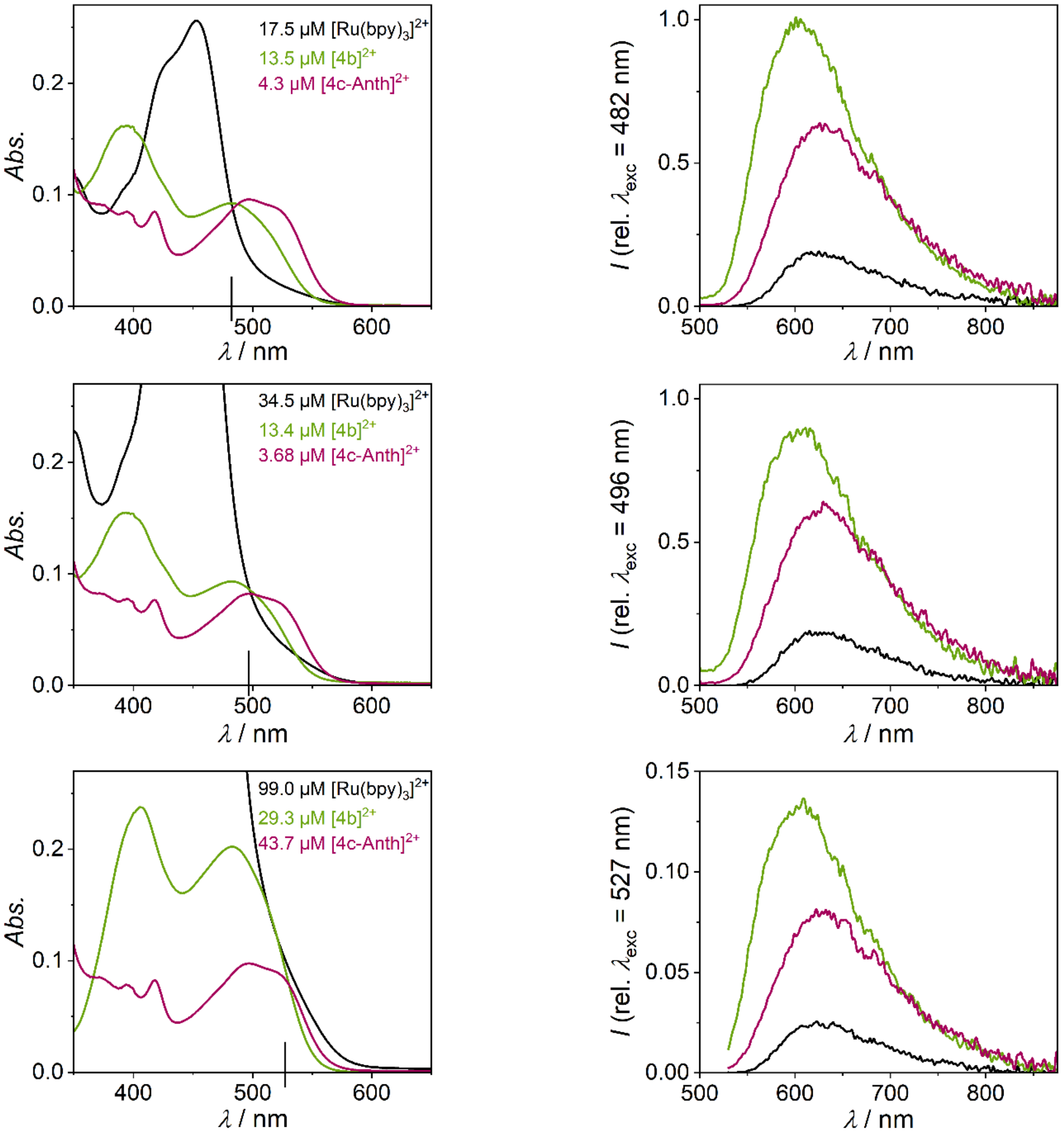

Figure S73. Absorption (left) and emission spectra (right) of $[Ru(bpy)_3]^{2+}$ (black, in H_2O), **[4b]$^{2+}$** (light green, in H_2O) and **[4c-Anth]$^{2+}$** (red, in H_2O). The excitation wavelength of the emission spectra is indicated at the abscissa of the absorption spectra.

Table S4. Quantum yields (Φ_{FL}) of individual measurements using different excitation wavelengths (λ_{exc} = 482, 496 or 527 nm), the averaged quantum yield (Φ_{AV}) and the standard deviation.

**Quantum yield**	**[4b]$^{2+}$**	**[4c-Anth]$^{2+}$**
Φ_{FL} (λ_{exc} = 482 nm)	0.35	0.26
Φ_{FL} (λ_{exc} = 496 nm)	0.34	0.26
Φ_{FL} (λ_{exc} = 527 nm)	0.40	0.29
Φ_{AV} (stdev.)	0.36 (± 0.026)	0.27 (± 0.014)

3.3) Solvent effects in compounds [4c-Ph]$^{2+}$, [4c-Naph]$^{2+}$ and [4c-Anth]$^{2+}$

Three compounds [**4c-Ph**]$^{2+}$ (Figure S74 and Table S5), [**4c-Naph**]$^{2+}$ (Figure S75 and Table S6) and [**4c-Anth**]$^{2+}$ (Figure S76 and Table S7) were tested for solvent effects using H_2O (E_T(30) = 63.1 kcal/mol; dielectric constant at 20 °C, ε = 80.16), MeOH (E_T(30) = 55.4 kcal/mol; dielectric constant at 20 °C, ε = 32.66), MeCN (E_T(30) = 45.6 kcal/mol; dielectric constant at 20 °C, ε = 35.94) and DCM (E_T(30) = 40.7 kcal/mol; dielectric constant at 25 °C, ε = 8.93) [17, 19]. For better comparability the absolute difference (Δ) between the maximum and minimum value of a given parameter was calculated. The ratio of this difference relative to the lowest value of the parameter was then calculated. The effect of the solvents on the absorption (λ_{max} (abs.)) and emission (λ_{max} (em.)) maxima is only minor and the relative difference amounts to ≤ 2% for all three compounds. Hence, the derived parameters (Stokes shift, $\Delta\nu$ and energy for the 0-0 transition, E_{00}) are also not highly sensitive to different solvent environments (relative difference ≤ 10%). These results indicate the absence of strong charge transfer effects after photoexcitation[19]. From the visualizations of the HOMO and LUMO orbitals (Figure S 142, Figure S 144 and Figure S 146), two factors become apparent: i) The orbital coefficients are distributed over the entire molecule in the HOMOs and the LUMOs and only the extent of the orbital contributions differ. From these calculations a slight redistribution of charges becomes apparent that might indicate a weakly pronounced charge transfer. However, no predominant charge transfer is predicted as for example in metal complexes[20], TADF molecules[21] or structurally related compounds containing a donor moiety (amino, oxo or thio groups)[25]. ii) The phosphorus atoms are not directly involved in either the HOMO or the LUMO electron distribution and only the bridging pentalene moiety and for the LUMO the ^{i}Pr substituents show orbital contributions. Those two observations also hold true for the other π-extended diphosphonium-bridged ladder stilbenes under investigation.

The lifetimes (τ_0) and relative quantum yields (Φ_{rel}) differ greatly. For **[4c-Ph]$^{2+}$** the lifetime in MeCN exceeds the lifetime in water by a factor of ~4 which is also reflected in an increased relative quantum yield (Table S7). This is consistent with the frequent observation that water acts as a quencher for red emitters. The differences in the lifetimes become smaller for the more symmetrical molecules **[4c-Naph]$^{2+}$** and **[4c-Anth]$^{2+}$.**

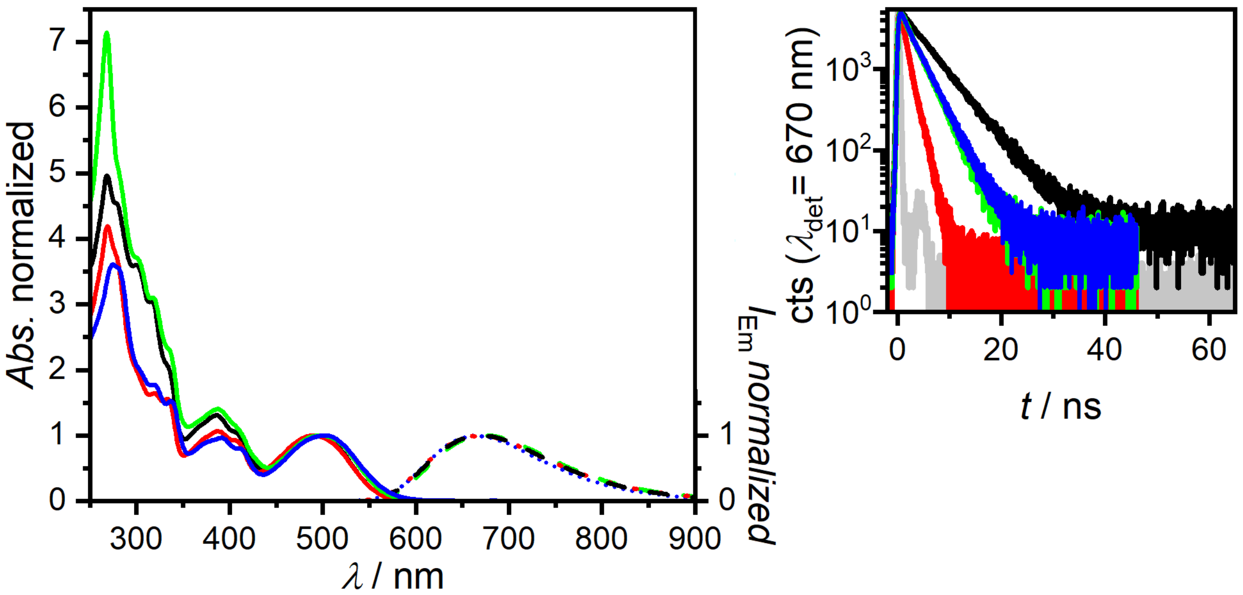

Figure S 74. Normalized UV-vis (solid lines) and emission spectra (dotted or dashed lines) and emission lifetimes (right part) of **[4c-Ph]$^{2+}$** in different solvents: 14 µM in MeCN (black data), 12 µM in H_2O (red data), 12 µM in MeOH (green data) and 8.3 µM in DCM (blue data). The UV-vis spectra have been normalized to 1 at the long-wavelength absorption band and accordingly the emission spectra were normalized. For the emission spectra, the samples dissolved in MeCN and H_2O were excited at 510 nm and the samples dissolved in MeOH and DCM were excited at 505 nm. For all lifetime measurements the EPL-510 was employed.

Table S5. Spectroscopic data for **[4c-Ph]$^{2+}$** in different solvents: absorption maxima at the lowest energy transition (λ_{max} (abs.)), emission maxima (λ_{max} (em.)), Stokes shift ($\Delta\nu$), the energy for the lowest energy transition (E_{00}), the emission lifetime (τ_0) and the emission quantum yield relative to the value obtained in aqueous solution (Φ). The scaled spectra and the lifetimes are provided in Figure S74.

	**H_2O**	**MeOH**	**MeCN**	**DCM**	**Δ[a] (relative)[b]**
**λ_{max} (abs.) / nm**	491	499	492	501	10 (0.02)
**λ_{max} (em.) / nm**	670	675	671	667	8 (0.01)
**$\Delta\nu$ / eV[c]**<br>**(cm^{-1})**	0.67<br>(5441)	0.65<br>(5225)	0.67<br>(5422)	0.62<br>(4968)	0.06 (0.09)<br>(474) (0.09)
**E_{00} / eV[d]**	2.19	2.17	2.19	2.16	0.03 (0.01)
**τ_0 / ns**	1.31	3.13	5.32	3.21	4.01 (>> 1)
**Φ_{rel}[e]**	1.00	n.a.[f]	1.22	n.a.	(~ 0.2)

[a] Absolute difference between highest and lowest value of the parameter.
[b] Ratio between Δ and the lowest value of the parameter.
[c] Calculated from the difference between λ_{max} (abs.) and λ_{max} (em.).
[d] Calculated from the intersection of the normalized absorption and emission spectrum.
[e] Calculated relative to the aqueous solution.
[f] Decomposition (>5%) during the measurement.

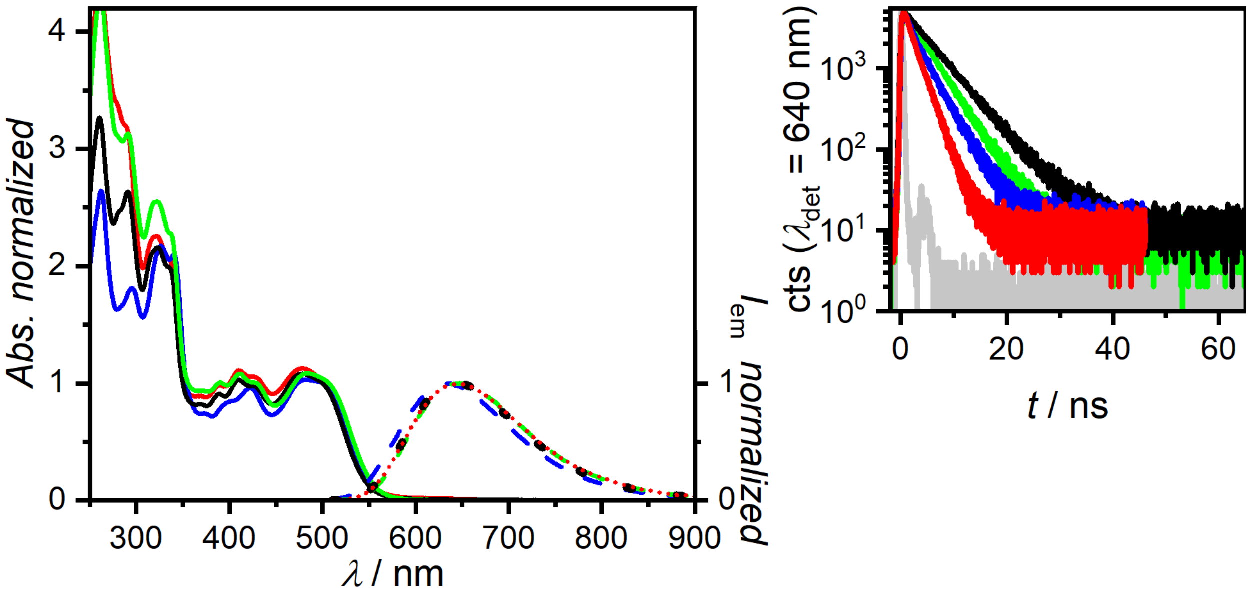

Figure S 75. Normalized UV-vis (solid lines) and emission spectra (dotted or dashed lines) and emission lifetimes (right part) of **[4c-Naph]$^{2+}$** in different solvents: 16 µM in MeCN (black data), 7.9 µM in H_2O (red data), 14 µM in MeOH (green data) and 12 µM in DCM (blue data). The UV-vis spectra have been normalized to 1 at the long-wavelength absorption band and accordingly the emission spectra were normalized. For the emission spectra, the samples were excited at 505 nm. For all lifetime measurements the EPL510 was employed.

Table S6. Spectroscopic data for **[4c-Naph]$^{2+}$** in different solvents: absorption maxima at the lowest energy transition (λ_{max} (abs.)), emission maxima (λ_{max} (em.)), Stokes shift ($\Delta\nu$), the energy for the lowest energy transition (E_{00}), the emission lifetime (τ_0) and the emission quantum yield relative to the value obtained in aqueous solution (Φ). The scaled spectra and the lifetimes are provided in Figure S 75.

	**H_2O**	**MeOH**	**MeCN**	**DCM**	**Δ[a] (relative)[b]**
**λ_{max} (abs.) / nm**	502	507	500	501	8 (0.01)
**λ_{max} (em.) / nm**	646	647	644	635	12 (0.02)
**$\Delta\nu$ / eV[c] (cm^{-1})**	0.55 (4440)	0.53 (4268)	0.55 (4472)	0.52 (4212)	0.03 (0.06) (260) (0.06)
**E_{00} / eV[d]**	2.23	2.23	2.24	2.25	0.03 (0.01)
**τ_0 / ns**	2.21	4.23	5.59	3.12	3.38 (0.40)
**Φ_{rel}[e]**	1.00	1.07[f]	1.66	0.88	(~ 0.9)

[a] Absolute difference between highest and lowest value.
[b] Ratio of the absolute difference Δ and the highest value of the.
[c] Calculated from the difference between λ_{max} (abs.) and λ_{max} (em.).
[d] Calculated from the intersection of the normalized absorption and emission spectrum.
[e] Calculated relative to the aqueous solution.
[f] Decomposition (>5%) during the measurement.

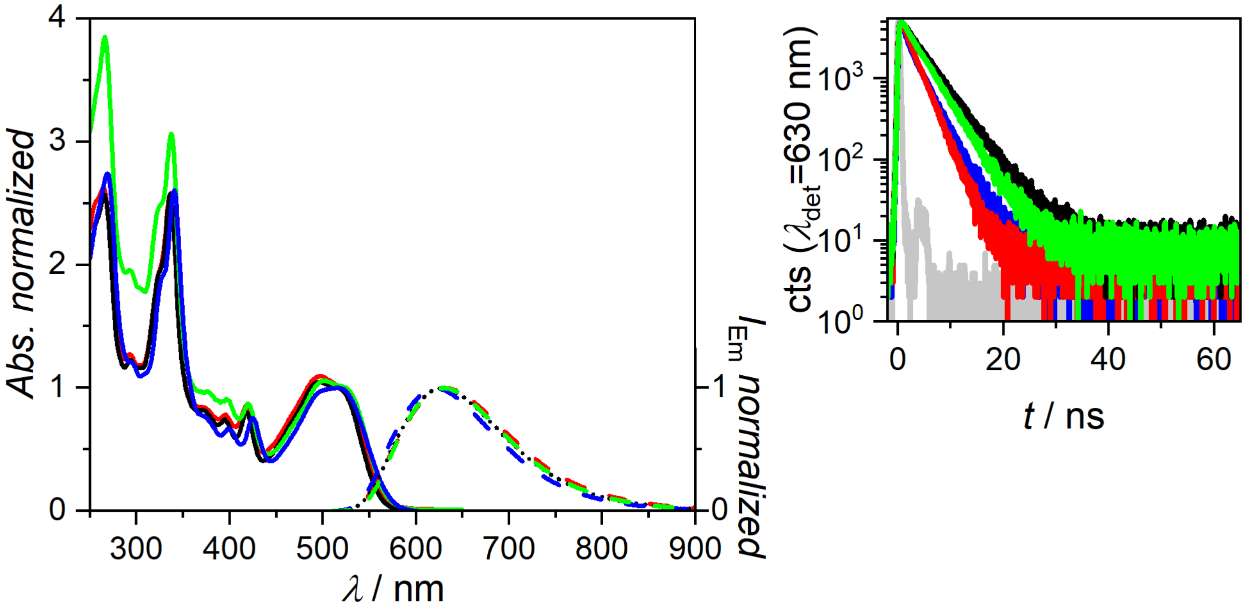

Figure S 76. Normalized UV-vis (solid lines) and emission spectra (dotted or dashed lines) and emission lifetimes (right part) of **[4c-Anth]$^{2+}$** in different solvents: 3.8 µM in MeCN (black data), 4.4 µM in H_2O (red data), 5.1 µM in MeOH (green data) and 4.6 µM in DCM (blue data). The UV-vis spectra have been normalized to 1 at the long-wavelength absorption band and accordingly the emission spectra were normalized. For the emission spectra, the samples were excited at 505 nm. For all lifetime measurements the EPL-510 was employed.

Table S7. Spectroscopic data for **[4c-Anth]$^{2+}$** in different solvents: absorption maxima at the lowest energy transition (λ_{max} (abs.)), emission maxima (λ_{max} (em.)), Stokes shift ($\Delta\nu$), the energy for the lowest energy transition (E_{00}), the emission lifetime (τ_0) and the emission quantum yield relative to the value obtained in aqueous solution (Φ). The scaled spectra and the lifetimes are provided in Figure S76.

	**H_2O**	**MeOH**	**MeCN**	**DCM**	**Δ[a] (relative) [b]**
**λ_{max} (abs.) / nm**	517	520	517	515	10 (0.02)
**λ_{max} (em.) / nm**	631	629	628	621	10 (0.02)
**$\Delta\nu$ / eV[c] (cm^{-1})**	0.42 (3383)	0.39 (3149)	0.42 (3419)	0.41 (3314)	0.02 (0.05) (165) (0.05)
**E_{00} / eV[d]**	2.23	2.21	2.23	2.22	0.02 (0.01)
**τ_0 / ns**	2.68	4.07	4.73	3.10	2.05 (0.77)
**Φ_{rel}[e]**	1.00	1.56[f]	0.66[f]	0.99	(~ 1.4)

[a] Absolute difference between highest and lowest value.
[b] Ratio of the absolute difference Δ and the lowest value of the.[c] Calculated from the difference between λ_{max} (abs.) and λ_{max} (em.).
[d] Calculated from the intersection of the normalized absorption and emission spectrum.
[e] Calculated relative to the aqueous solution.
[f] Decomposition (>5%) during the measurement.

The emission spectra were measured in water for **[4c-Ph]$^{2+}$** (Figure S74), **[4c-Naph]$^{2+}$** (Figure S75), and **[4c-Anth]$^{2+}$** (Figure S76) and they are also displayed in the main manuscript (Table 1).

3.4) Comparison of photophysical properties between *P*-containing ladder stilbenes

To evaluate the influence of the R_2P^+ group on the photophysical properties of phosphorus-bridged ladder stilbenes, we compared these compounds to the well-known 5,10-dihydro indeno[2,1-a]indene motif **[A]**. A direct comparison reveals a pronounced bathochromic shift in both the absorption and emission maxima of the phosphorus-bridged derivatives relative to

[A], while maintaining similarly high photoluminescence quantum yields. Moreover, compound **[A]** exhibits a larger HOMO–LUMO energy gap of 4.1 eV. The most significant difference, however, lies in the fluorescence lifetimes: **[A]** displays an exceptionally short fluorescence lifetime of 1.6 ns, whereas the phosphorus-bridged analogues **[H]$^{2+}$**, **[H$_{Ph}$]$^{2+}$**, and **[H$_{PhO}$]** exhibit lifetimes approximately an order of magnitude longer.

Table S8. Photophysical characteristics of different bridged ladder-stilbene chromophores.

	[A]	$[H]^{2+}$	$[H_{ph}]^{2+}$	$[H_{pho}]$
***ε* / 10^3**	28.2	8.4	5.17	6.92
***M^{-1}cm^{-1}* (nm)**	(322)[e]	(400)[f]	(417)[g]	(395)[h]
**τ_0 / ns**	1.6	14.7	15.2	15.7
**λ_{max} / nm[d]**	367	494	518	480
**ϕ_{FL}**	0.92	0.87	0.84[a]	0.98
**k_{FL} / 10^7 s^{-1}**	57.5	5.9	5.5[a]	6.2
**k_{nr} / 10^7 s^{-1}**	5.0	0.9	1.1[a]	0.1
**Δν (cm^{-1})**	3807	4757	4675	4483
**E$_{HOMO/LUMO}$ [eV]**	4.1[b]	3.5[c]	3.4[c]	3.6[b]
**Ref.**	**[24]**	**[22]**	**[23]**	**[24]**

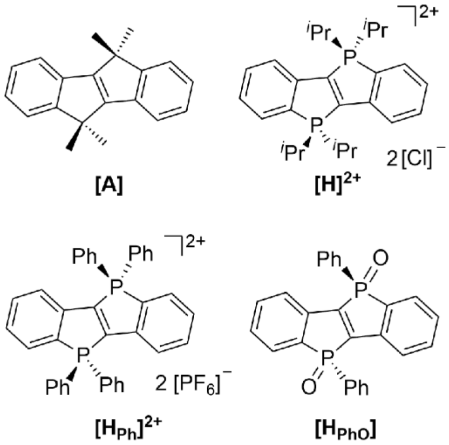

[a] The quantum yield of $[H_{Ph}]^{2+}$ was redetermined for this work. [b] Kohn-Sham HOMO/LUMO energy levels were calculated at the B3LYP/6-31G(d) level[24]. [c] Kohn-Sham HOMO/LUMO energy levels were calculated at the B3LYP-D3/def2TZVPP level and PCM solvent-corrected for CH_2Cl_2[23].[d] λ_{max} of the emission spectrum. [e] Measured in THF[24]. [f] Measured in aqueous 50 mM H_2SO_4[22]. [g] Measured in MeCN[23]. [e] Measured in CH_2Cl_2[24].

The quantum yield of $[H_{Ph}]^{2+}$ (see Table S8 for its structure) was redetermined in MeCN and for that two reference compounds were used; the structurally related **[H]$^{2+}$** (in 50 mM H_2SO_4, Φ_{FL} = 0.87)[22] and fluorescein (in 0.1 M NaOH, Φ_{FL} = 0.88)[18]. An emission quantum yield of 0.84 (± 0.02) was determined, which is higher compared to the value that was published earlier (Φ_{FL} = 0.24)[23]; likely due to decomposition in the previous measurement as a result of intense excitation. For the measurements displayed in Figure S77 all solutions were purged 10 min prior to the measurements ensuring that no acetonitrile evaporates thoughout the process for the $[H_{Ph}]^{2+}$ solutions. Decomposition of $[H_{Ph}]^{2+}$ in acetonitrile was observed and the measurements in Figure S77 were performed very carefully, ensuring <5% of decomposition, which was controlled via accompanied UV-vis absorption spectroscopy.

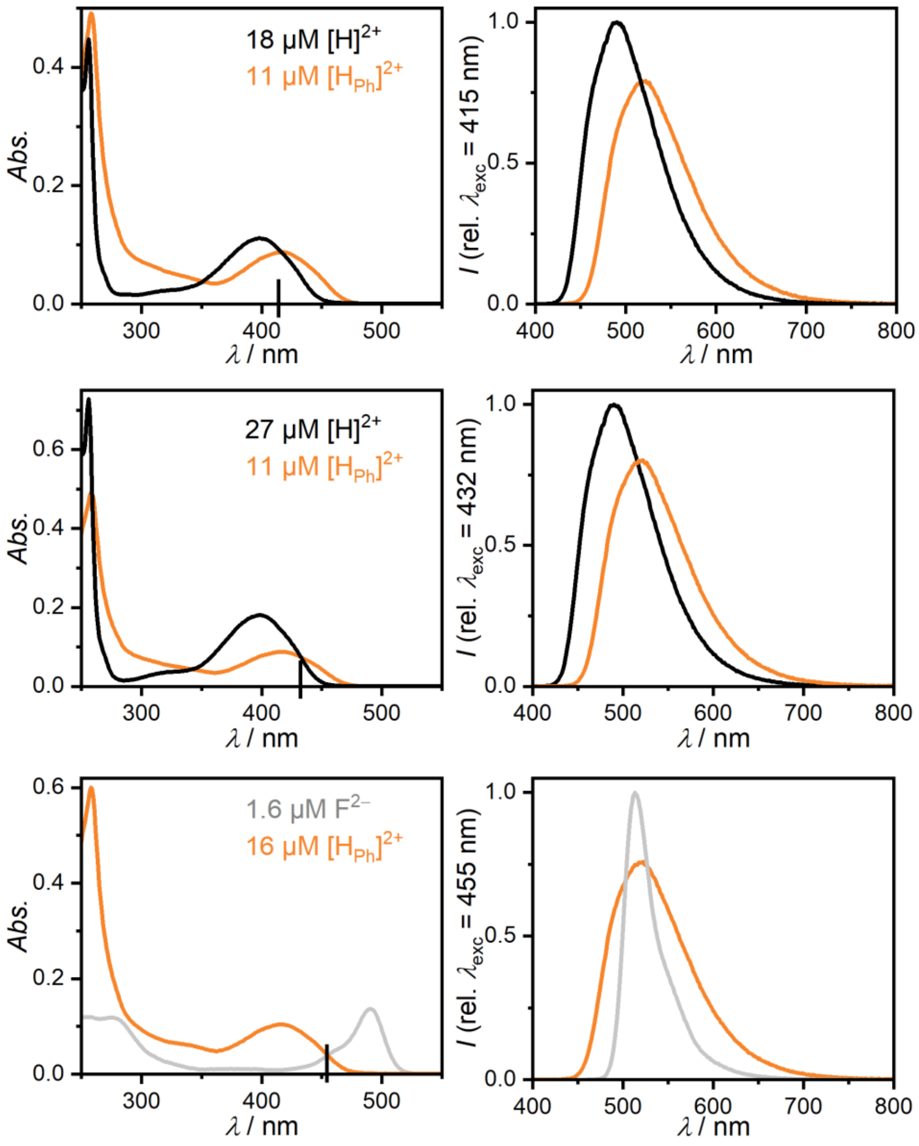

Figure S 77 Absorption (left) and emission spectra (right) of $[H_{Ph}]^{2+}$ (orange, in MeCN), $[H]^{2+}$ (black, in 50 mM H_2SO_4) and fluorescein (F^{2-}, gray, in 0.1 M NaOH). The excitation wavelength of the emission spectra is indicated at the abscissa of the absorption spectra.

Table S9. Fluorescence quantum yields of $[H_{Ph}]^{2+}$ in aceteonitrile (Φ_{FL}) for individual measurements using different excitation wavelengths (λ_{exc} = 415 nm, λ_{exc} = 432 nm or λ_{exc} = 455 nm) and two different reference compounds.

**Compound**	Φ_{FL} **(λ_{exc} = 415 nm)**	Φ_{FL} **(λ_{exc} = 432 nm)**	Φ_{FL} **(λ_{exc} = 455 nm)**	Φ_{AV} ***(stdev.)***
**$[H_{Ph}]^{2+}$**	0.82[a]	0.82[a]	0.87[b]	0.84 (0.02)

[a] Relative quantum yield using $[H]^{2+}$ (Φ_{FL} = 0.87)[22] as a reference.
[b] Relative quantum yield using Fluorescein (F^{2-}, Φ_{FL} = 0.88)[18] as a reference.

In comparison to structurally related monocationic P/O-, P/N-, and P/S-bridged ladder stilbenes reported by Sobolewski, Szewczyk, and Gryko, the **$[H]^{2+}$** system described herein exhibits a bathochromically shifted absorption maximum (by ~30 to ~60 nm), while the emission spectra are largely comparable. The HOMO–LUMO gap at approximately 3.1 eV is significantly smaller than in **$[H]^{2+}$**. Notably, the P/N- as well as the P/O-bridged derivative feature a substantially larger Stokes shift of about 6000 cm^{-1},

indicative of a more pronounced structural relaxation upon excitation, which suggests a greater geometric disparity between the ground and excited states in these systems compared to **[H]²⁺**.

Table S10. Photophysical characteristics of different P-containing bridged ladder-stilbene chromophores.

	$[H]^{2+}$	$[H_N]^{+}$	$[H_O]^{+}$	$[H_S]^{+}$
**ε / 10^3 $M^{-1}cm^{-1}$ (nm)**	8.4 (400)[d]	3.8 (371)[e]	5.4 (360)[e]	3.9 (340)[e]
**τ_0 / ns**	14.7	-	-	-
**λ_{max} / nm[c]**	494	490	460	460
**ϕ_{FL}**	0.87	0.89	0.69	0.61
**k_{FL} / 10^7 s^{-1}**	5.9	-	-	-
**k_{nr} / 10^7 s^{-1}**	0.9	-	-	-
**$\Delta\nu$ (cm^{-1})**	4757	6500	6000	5400
**$E_{HOMO/LUMO}$ [eV]**	3.5[a]	3.0[b]	3.1[b]	3.1[b]
**Ref.**	**[22]**	**[25]**	**[25]**	**[25]**

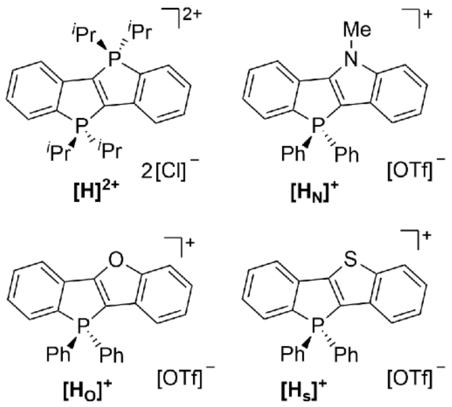

[a] Kohn-Sham HOMO/LUMO energy levels were calculated at the B3LYP-D3/def2TZVPP level and PCM solvent-corrected for CH_2Cl_2[23]. [b] The HOMO/LUMO energy level were determined via cyclic voltammetry[25].[c] λ_{max} of the emission spectrum. [d] Measured in aqueous 50 mM H_2SO_4[22]. [e] Measured in CH_2Cl_2[25].

4) Time-resolved measurements

The lifetimes of all emitters dissolved in argon-saturated water were determined using the TSCPC technique. The diphosphonium compounds were excited at 3-4 different wavelengths. Filters were chosen such that a good overlap with the emission spectrum was ensured, while avoiding scattering artifacts. The mono-exponential fitting function of Origin was employed to obtain the lifetimes.

Table S11. Lifetimes (τ) of all emitters in argon-saturated water. The averaged lifetimes and the standard deviations are given in the right column.

**compound (Filter)**	**τ / ns (λ_{exc} = 293 nm)**	**τ / ns (λ_{exc} = 371 nm)**	**τ / ns (λ_{exc} = 446 nm)**	**τ / ns (λ_{exc} = 505 nm)**	**τ / ns (stdev.)**
**[4a-Ph]$^{2+}$**	9.1	9.1	9.0	-	9.1 (±0.05)
**[4a]$^{2+}$**	6.4	6.4	6.3	-	6.4 (±0.05)
**[4b-Ph]$^{2+}$**	14.2	14.0	14.1	-	14.1 (±0.08)
**[4b]$^{2+}$**	10.5	10.5	10.5	10.5	10.5 (±0.00)
**[4c-Ph]$^{2+}$**	1.3	1.3	1.3	1.3	1.3 (±0.00)
**[4c-Naph]$^{2+}$**	2.2	2.1	2.2	2.2	2.2 (±0.04)
**[4c-Anth]$^{2+}$**	2.6	2.7	2.6	2.6	2.6 (±0.04)

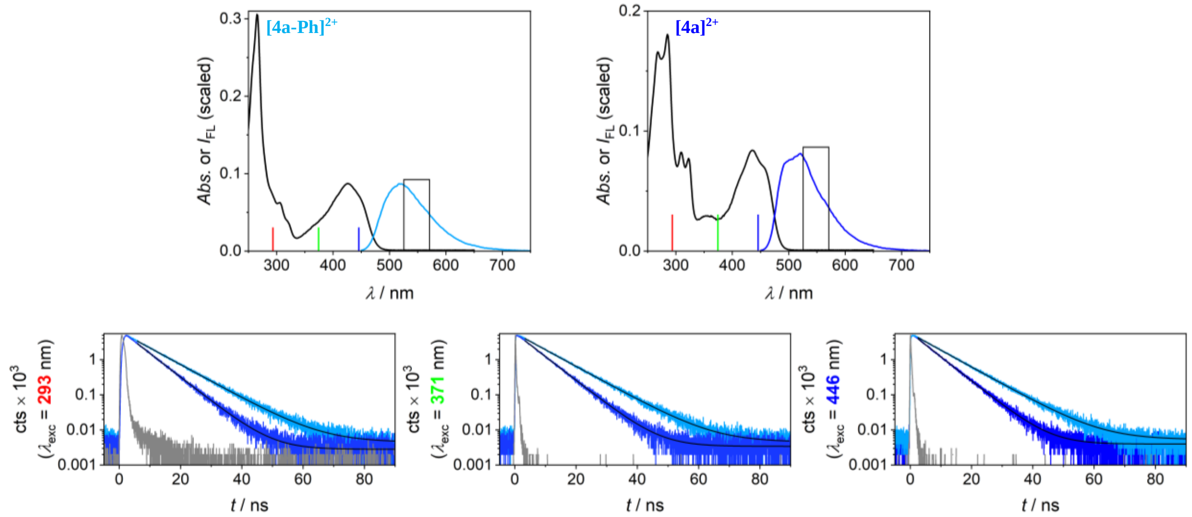

Figure S78. Emission decay measurements of **[4a-Ph]$^{2+}$** (light blue data, 19 µM for excitation at 371 nm, 7.5 µM for excitation at 446 nm and 293 nm) and **[4a]$^{2+}$** (blue data, 18 µM for excitation at 371 nm, 5.1 µM for excitation at 446 nm and 293 nm). Upper part: Absorption and emission spectra with the wavelengths of excitation highlighted in red (293 nm), green (371 nm) and blue (446 nm, this data set was used in Figure 3 of the main part). The box indicates the bandpass filter that was used for the detection. Lower part: Emission decay traces at different excitation wavelengths (same color code as before), the IRF of the excitation lasers/diodes (gray) and the fitting functions (black). The resulting lifetimes can be found in Table S11.

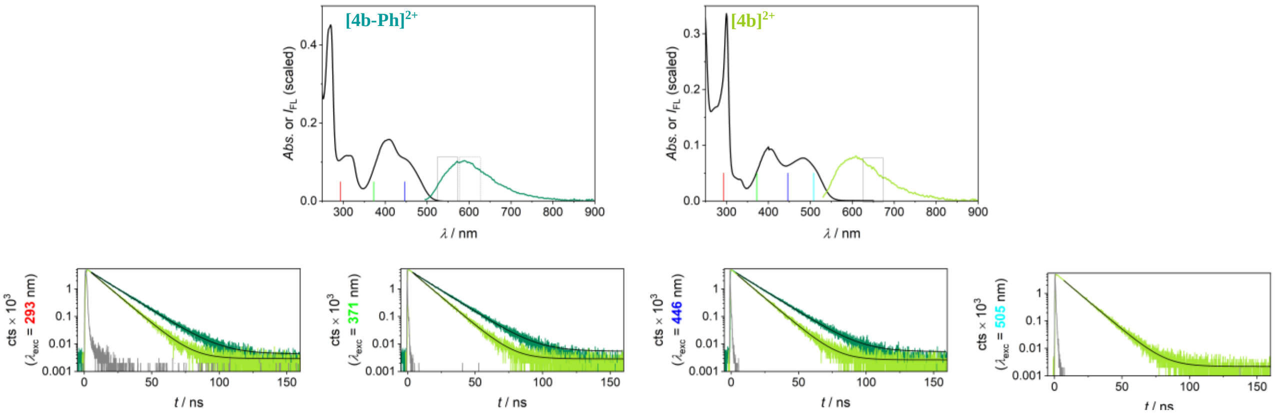

Figure S79. Emission decay measurements of [**4b-Ph**]$^{2+}$ (dark green data, 24 µM for all excitation wavelengths) and [**4b**]$^{2+}$ (light green data, 16 µM for excitation at 371 nm, 446 nm and 505 nm, 6.9 µM for excitation at 293 nm). Upper part: Absorption and emission spectra with the wavelengths of excitation highlighted in red (293 nm), green (371 nm), blue (446 nm this data set was used in Figure 3 of the main part) and turquoise (505 nm). The box indicates the bandpass filters that were used for the detection. Lower part: Emission decay traces at different excitation wavelengths (same color code as before), the IRF of the excitation lasers/diodes (gray) and the fitting functions (black). The resulting lifetimes can be found in Table S11.

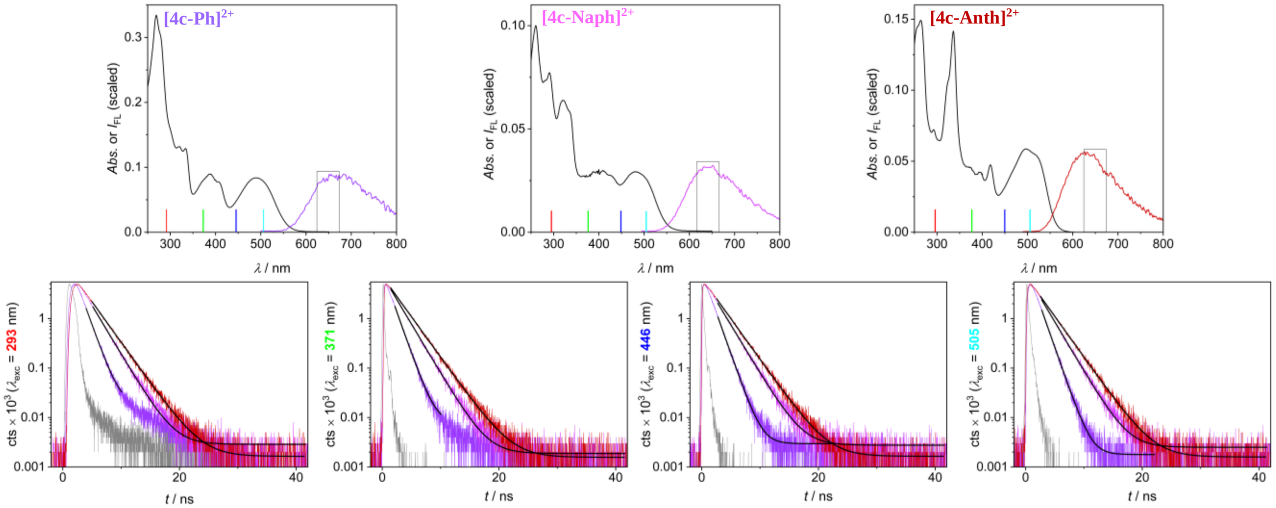

Figure S80. Emission decay measurements of [**4c-Ph**]$^{2+}$ (purple data, 8.6 µM for all excitation wavelengths), [**4c-Naph**]$^{2+}$ (pink data, 8.3 µM for excitation at 505nm, 446 nm and 371 nm, 2.6 µM for excitation at 293 nm) and [**4c-Anth**]$^{2+}$ (red data, 2.7 µM for all excitation wavelengths). Upper part: Absorption and emission spectra with the wavelengths of excitation highlighted in red (293 nm), green (371 nm), blue (446 nm) and turquoise (505 nm this data set was used in Figure 3 of the main part). The box indicates the bandpass filter that was used for the detection. Lower part: Emission decay traces at different excitation wavelengths (same color code as before), the IRF of the excitation lasers/diodes (gray) and the fitting functions (black). The resulting lifetimes can be found in Table S11.

5) Details on DFT Calculations

5.1) General Remarks

DFT calculations were carried out using Orca 5.0.4.[26] All structures were optimized without symmetry restrictions and identified as minima by frequency analyses. The r^{2}SCAN-3c functional was employed in combination with the def2-mTZVPP basis set.[27] To speed-up the computations, the resolution of the identity (RI) approximation was used in conjunction with the def2-mTZVPP/J auxiliary basis set.[27] Geometrical counterpoise corrections (gCP)[28] and dispersion corrections (D4)[29] were taken into account as implemented in Orca.

TD-DFT calculations were carried out using Gaussian 16 (G16RevC.01)[30] using the B3LYP hybrid functional.[31] For the respective ground states, geometry optimizations were carried out without symmetry restrictions and all stationary point were identified as minima by analytical frequency analysis. Ahlrichs' def2-TZVPP basis set[32] was used in combination with Grimme's dispersion correction (GD3)[33]. The energies were corrected for solvent effects using the polarizable continuum model (PCM) for water (SCRF=(Solvent=Water))[34] as implemented in Gaussian.

AICD computation were carried out using AICD-3.0.4[35] in combination with Gaussian 16 (keyword iop(10/93=1)) and plotted using POV-Ray 3.7.0[36]. NICS calculations were carried out with Gaussian 16 using py.Aroma4 for input preparation and analysis.[37] The calculated UV-Vis spectra were visualized using GaussView 6.1.[38] All other structures were visualized plotted using ChemCraft (v1.8).[39]

Optimized coordinates for all structures are provided as separate files (xyz).

5.2) Computational Results for Mechanism A (Cyclization prior to SET Steps)

Thermal Cyclization of 3a-Ph to [4a-Ph] ^{0}

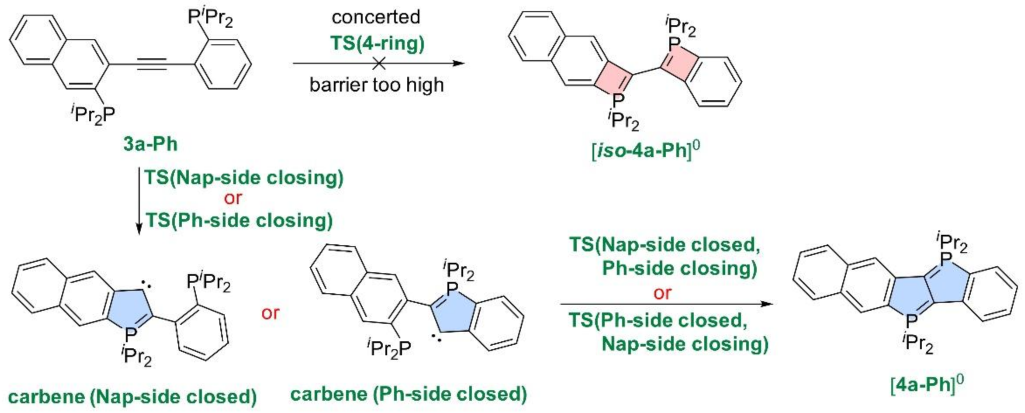

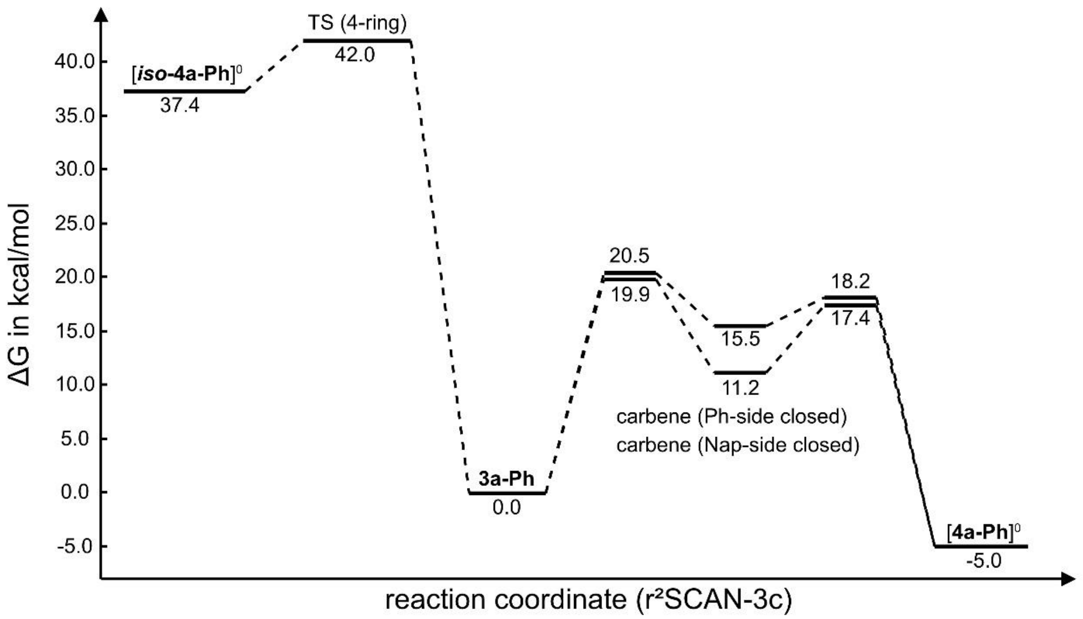

Figure S81. Gibbs free energy profile for the conversion of **3a-Ph** to [***iso*-4a-Ph**]^{0} and to [**4a-Ph**]^{0} (r^2SCAN-3c, def2-mTZVPP, D4, CPCM for CH_2Cl_2). The formation of [***iso*-4a-Ph**]^{0} is excluded at rt.

Table S12. Gibbs free energies for the stationary points shown in the energy profile above (r^2SCAN-3c, def2-mTZVPP, D4, CPCM for CH_2Cl_2).

compound	Gibbs free energy (a.u.)	ΔG (rel. to **3a-Ph**) (kcal/mol)
**[*iso*-4a-Ph]^{0}**	-1847.8322086	**37.36**
TS(4-ring)	-1847.8247730	**42.03**
**3a-Ph**	-1847.8917460	**0.00**
TS(Nap-side closing)	-1847.8600376	**19.90**
TS(Ph-side closing)	-1847.8591256	**20.47**
**carbene (Ph-side closed)**	-1847.8669882	**15.54**
**carbene (Nap-side closed)**	-1847.8739685	**11.16**
TS(Nap-side closed, Ph-side closing)	-1847.8639818	**17.42**
TS(Ph-side closed, Nap-side closing)	-1847.8628096	**18.16**
[**4a-Ph**]^{0}	-1847.8996931	**-4.99**

Thermal Cyclization of 3b-Ph to [4b-Ph]⁰

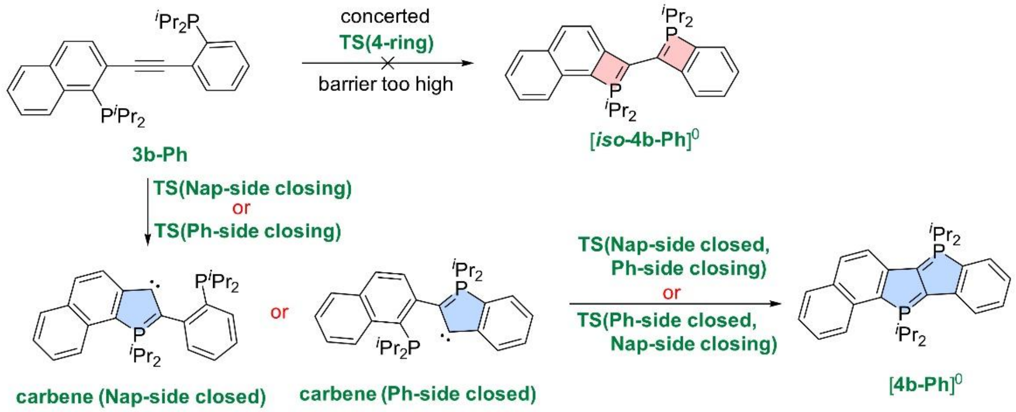

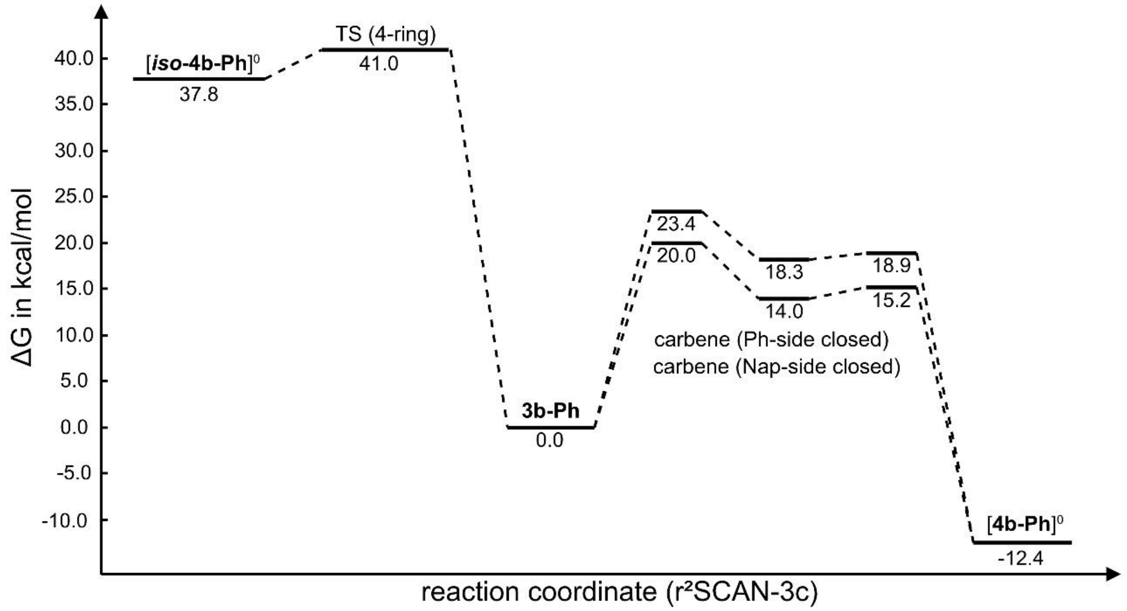

Figure S82. Gibbs free energy profile for the conversion of **3b-Ph** to [***iso*-4b-Ph**]⁰ and to [**4b-Ph**]⁰ (r^2SCAN-3c, def2-mTZVPP, D4, CPCM for CH_2Cl_2). The formation of [***iso*-4b-Ph**]⁰ is excluded at rt.

Table S13. Gibbs free energies for the stationary points shown in the energy profile above (r^2SCAN-3c, def2-mTZVPP, D4, CPCM for CH_2Cl_2).

compound	Gibbs free energy (a.u.)	ΔG (rel. to **3a-Ph**) (kcal/mol)
**[*iso*-4b-Ph]⁰**	-1847.8240517	**37.82**
TS(4-ring)	-1847.8190361	**40.97**
**3b-Ph**	-1847.8843258	**0.00**
TS(Nap-side closing)	-1847.8524453	**20.01**
TS(Ph-side closing)	-1847.8470253	**23.41**
**carbene (Ph-side closed)**	-1847.8551467	**18.31**
**carbene (Nap-side closed)**	-1847.8619751	**14.03**
TS(Nap-side closed, Ph-side closing)	-1847.8600368	**15.24**
TS(Ph-side closed, Nap-side closing)	-1847.8542352	**18.88**
[**4b-Ph**]⁰	-1847.9040639	**-12.39**

Thermal Cyclization of 3a to [4a]^0

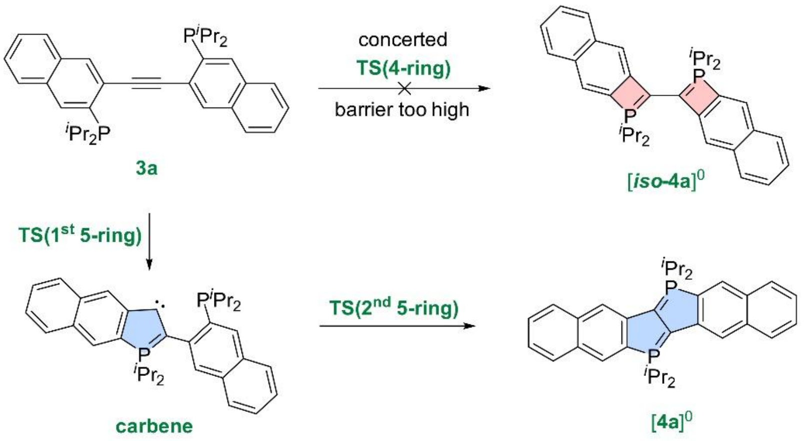

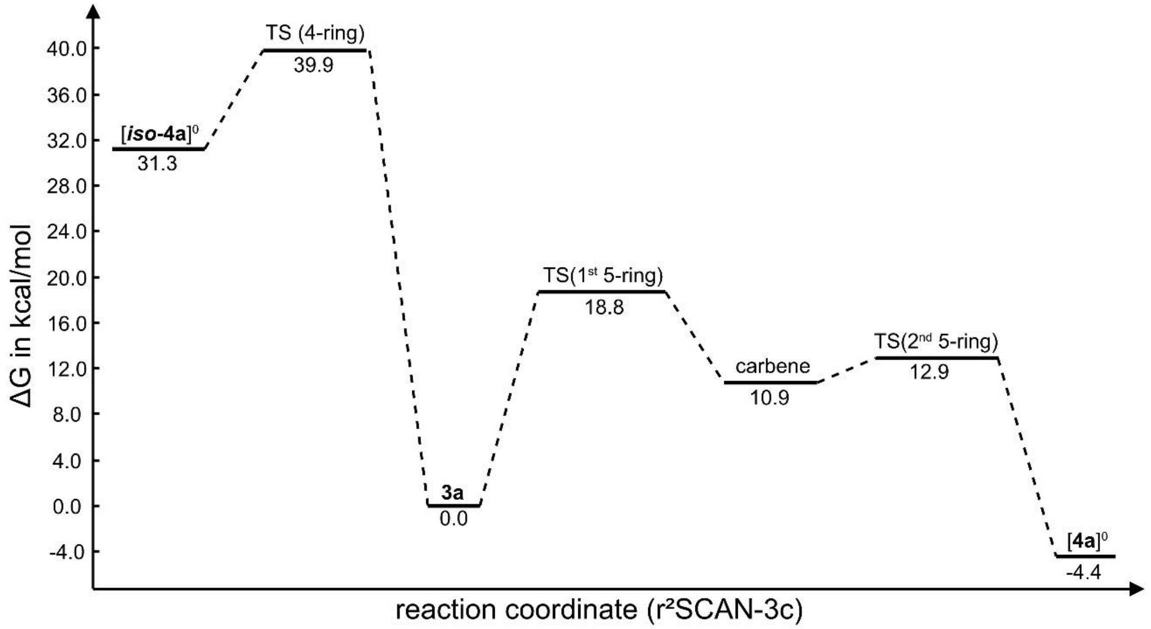

Figure S83. Gibbs free energy profile for the conversion of **3a** to [***iso*-4a**]^0 and to [**4a**]^0 (r^{2}SCAN-3c, def2-mTZVPP, D4, CPCM for CH_2Cl_2). The formation of [***iso*-4a**]^0 is excluded at rt.

Table S14. Gibbs free energies for the stationary points shown in the energy profile above (r^{2}SCAN-3c, def2-mTZVPP, D4, CPCM for CH_2Cl_2).

compound	Gibbs free energy (a.u.)	ΔG (rel. to **3a-Ph**) (kcal/mol)
**[*iso*-4a]^0**	-2001.4110728	**31.31**
TS(4-ring)	-2001.3974240	**39.88**
**3a**	-2001.4609747	**0.00**
TS(1st 5-ring)	-2001.4310601	**18.77**
**carbene**	-2001.4436604	**10.86**
TS(2nd 5-ring)	-2001.4403438	**12.94**
[**4a**]^0	-2001.4679452	**-4.37**

Thermal Cyclization of 3b to [4b]^0

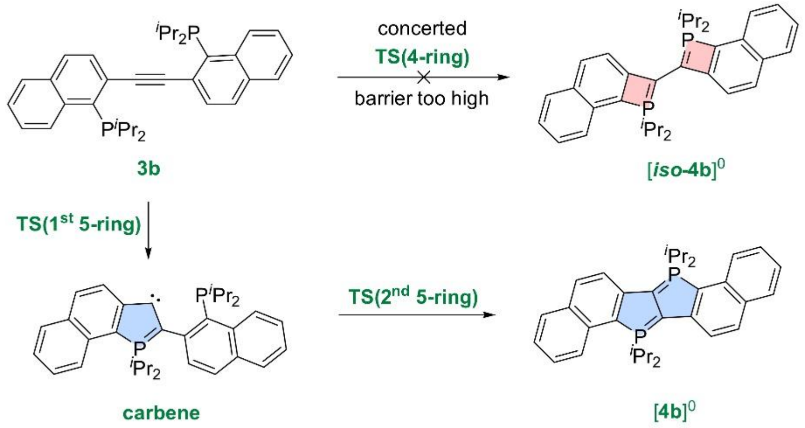

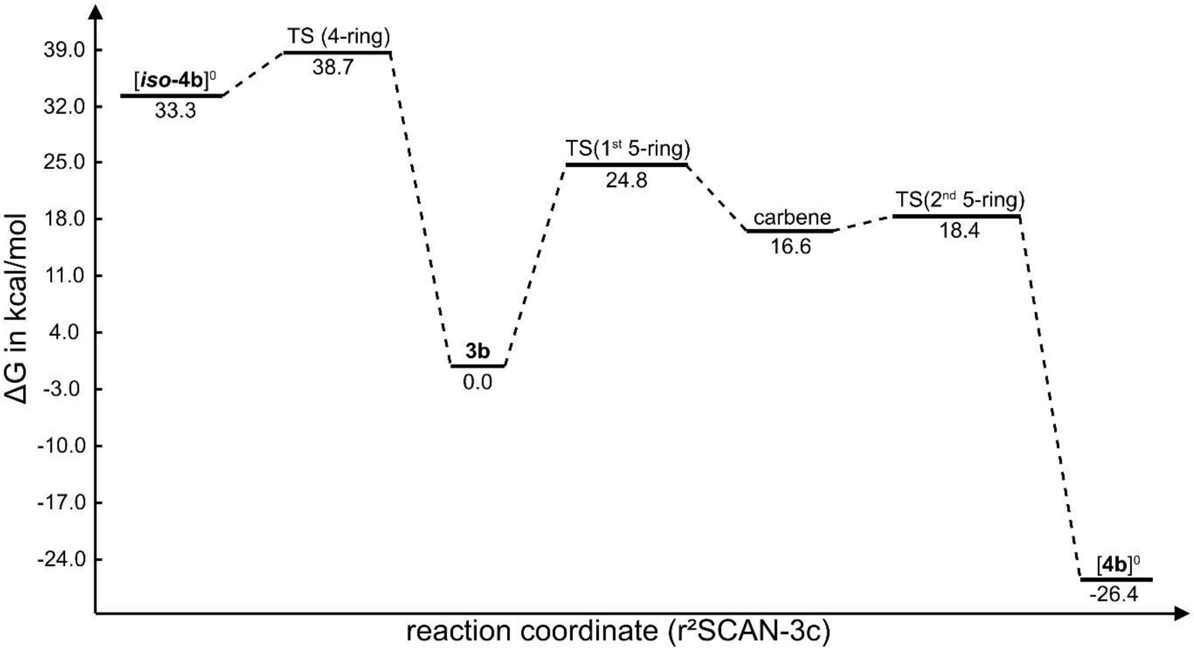

Figure S84. Gibbs free energy profile for the conversion of **3b** to [***iso*-4b**]^0 and to [**4b**]^0 (r^2SCAN-3c, def2-mTZVPP, D4, CPCM for CH_2Cl_2). The formation of [***iso*-4b**]^0 is excluded at rt.

Table S15. Gibbs free energies for the stationary points shown in the energy profile above (r^2SCAN-3c, def2-mTZVPP, D4, CPCM for CH_2Cl_2).

compound	Gibbs free energy (a.u.)	ΔG (rel. to **3a-Ph**) (kcal/mol)
**[*iso*-4b]^0**	-2001.3959868	**33.31**
TS(4-ring)	-2001.3874294	**38.68**
**3b**	-2001.4490757	**0.00**
TS(1st 5-ring)	-2001.4096076	**24.77**
**carbene**	-2001.4226620	**16.57**
TS(2nd 5-ring)	-2001.4197145	**18.42**
[**4b**]^0	-2001.4911497	**-26.40**

Relaxed PES scans for 3a-Ph/[iso-4a-Ph]^0, 3b-Ph/[iso-4b-Ph]^0, 3a/[iso-4a]^0 and 3b/[iso-4b]^0

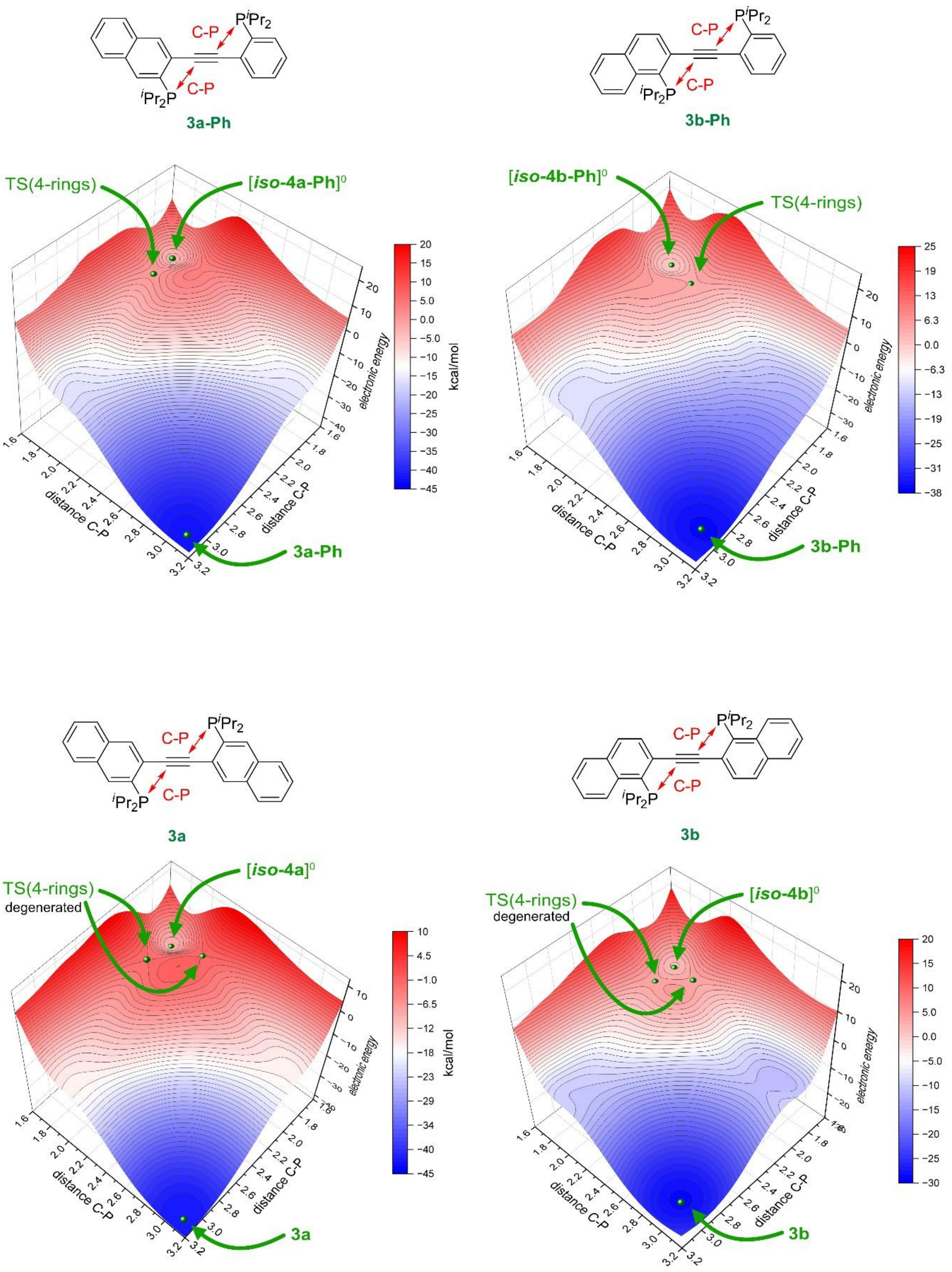

Figure S85. Relaxed two-dimensional potential energy surface scans for the thermal cyclization of compounds **3** to compounds [***iso*-4**]^0 (r^{2}SCAN-3c, def2-mTZVPP, D4, CPCM for CH_2Cl_2). The formation of compounds [***iso*-4**]^0 is thermodynamically uphill in all cases, i.e. the formation of compounds [***iso*-4**]^0 is excluded.

Optimized Geometries for the Thermal Cyclization of 3a-Ph to *P*-diylidic [4a-Ph]^0

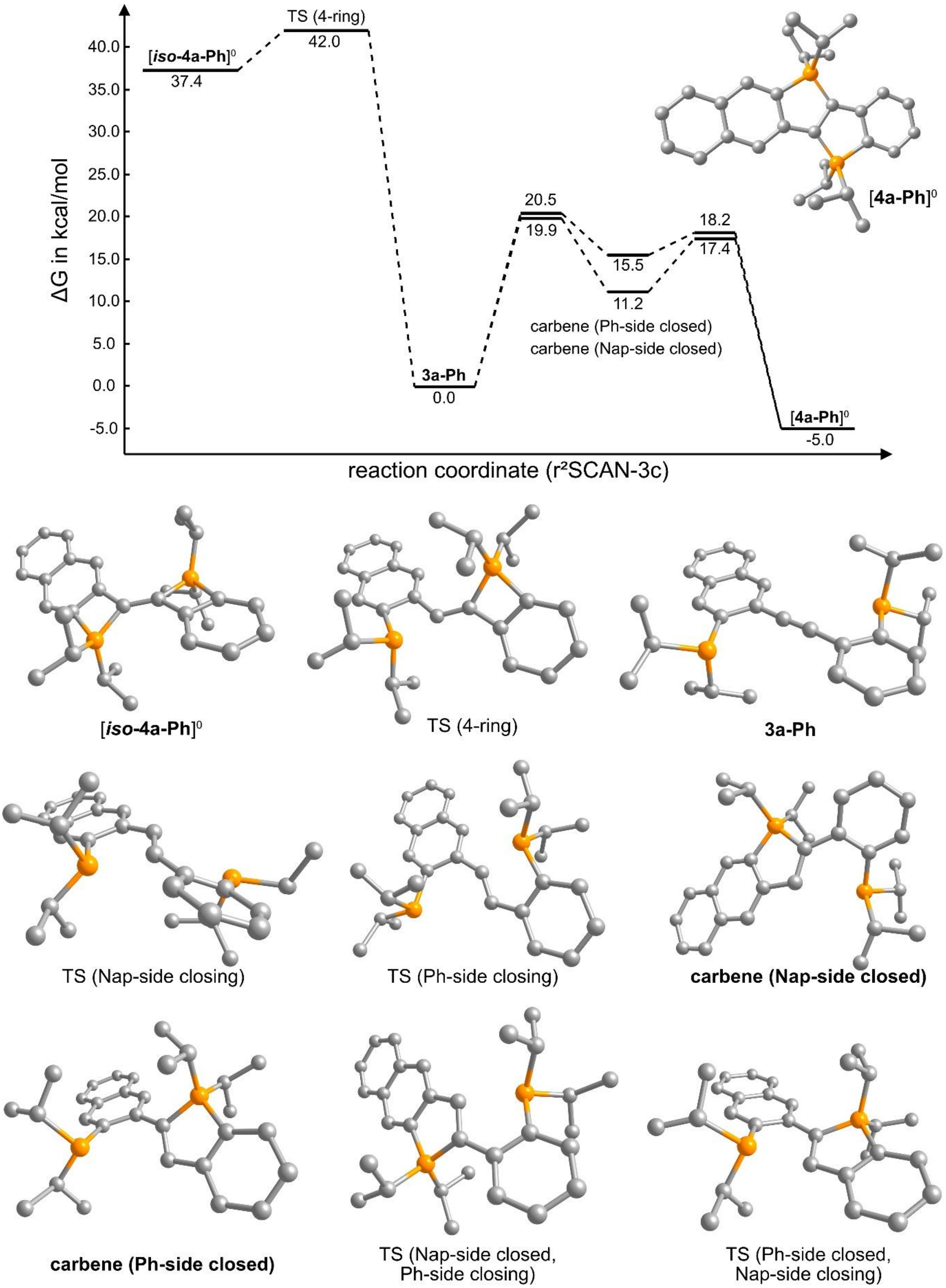

Figure S86. Optimized geometries (r^{2}SCAN-3c, def2-mTZVPP, D4, CPCM for CH_2Cl_2) for all compounds that may play a role in the thermal cyclization of **3a-Ph** to [**4a-Ph**]^0. Hydrogen atoms are omitted for clarity.

Optimized Geometries for the Thermal Cyclization of 3b-Ph to *P*-diylidic [4b-Ph]^0

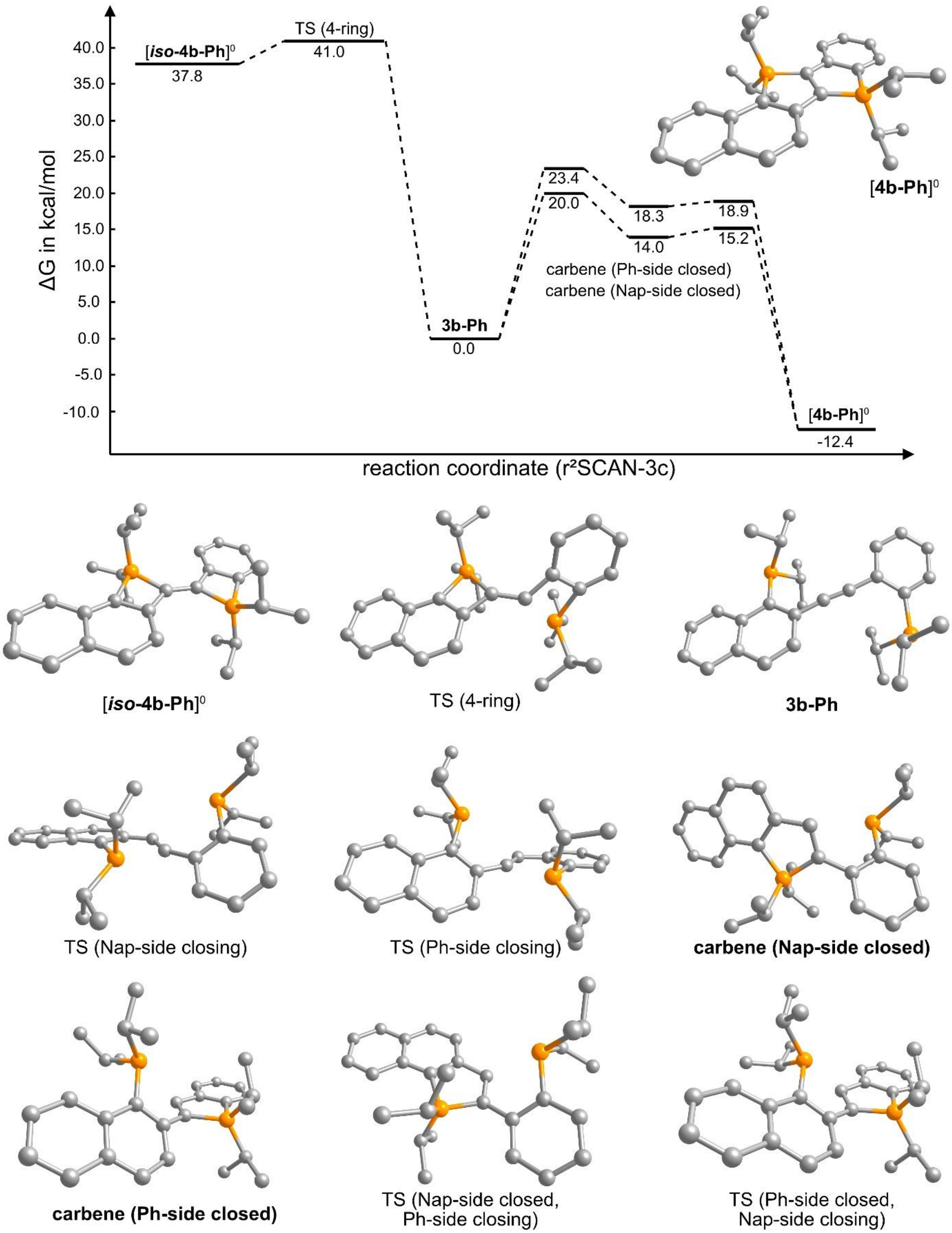

Figure S87. Optimized geometries (r^{2}SCAN-3c, def2-mTZVPP, D4, CPCM for CH_2Cl_2) for all compounds that may play a role in the thermal cyclization of **3b-Ph** to [**4b-Ph**]^0. Hydrogen atoms are omitted for clarity.

Optimized Geometries for the Thermal Cyclization of 3a to *P*-diylidic [4a]^0

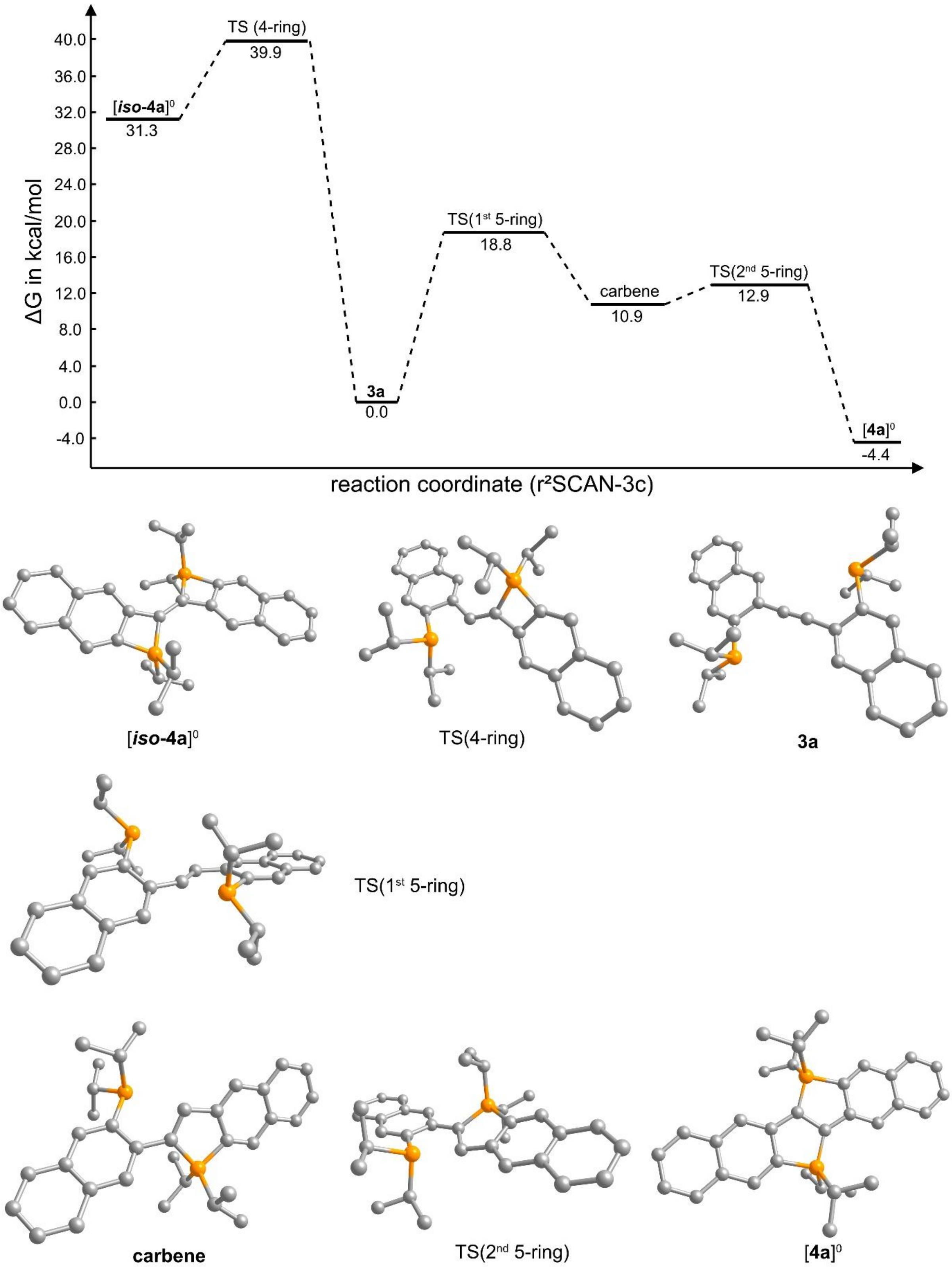

Figure S88. Optimized geometries (r^2SCAN-3c, def2-mTZVPP, D4, CPCM for CH_2Cl_2) for all compounds that may play a role in the thermal cyclization of **3a** to [**4a**]^0. Hydrogen atoms are omitted for clarity.

Optimized Geometries for the Thermal Cyclization of 3b to *P*-diylidic [4b]^0

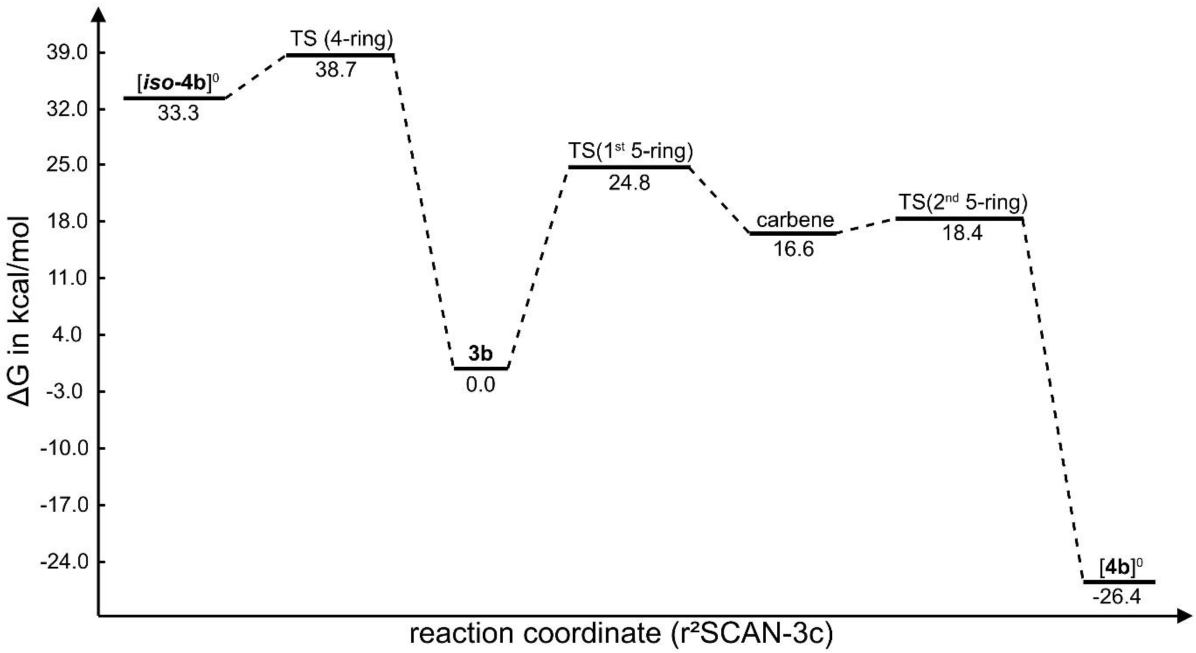

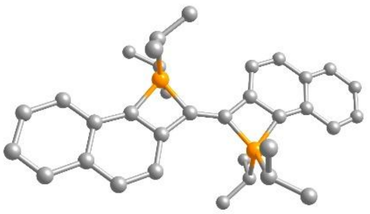

[***iso*-4b**]^0

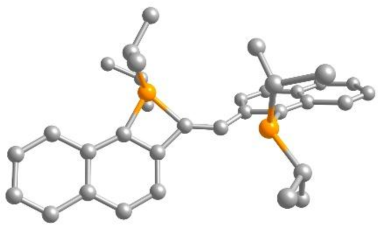

TS(4-ring)

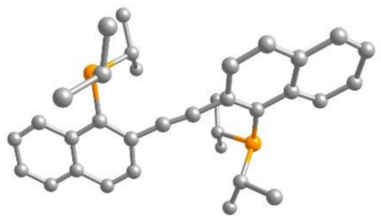

3b

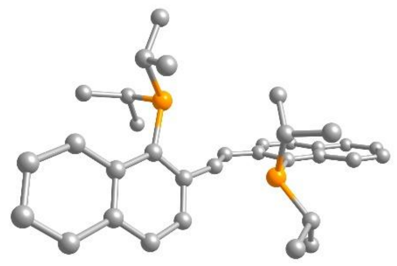

TS(1st 5-ring)

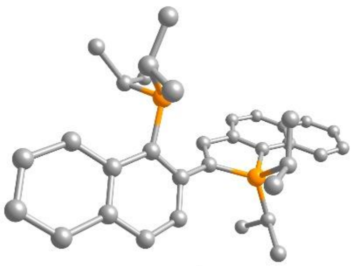

carbene

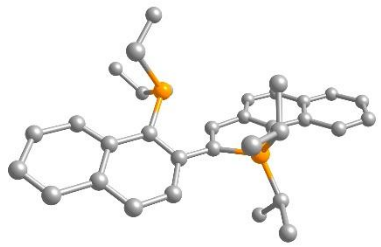

TS(2nd 5-ring)

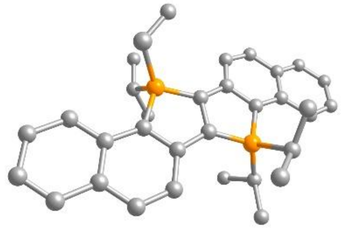

[**4b**]^0

Figure S89. Optimized geometries (r^{2}SCAN-3c, def2-mTZVPP, D4, CPCM for CH_2Cl_2) for all compounds that may play a role in the thermal cyclization of **3b** to [**4b**]^0. Hydrogen atoms are omitted for clarity.

5.3) Computational Results for Mechanism B (Cyclization after Ar^{i}Pr$_2$P–Cl$^+$ Formation)

Cyclization of [INT-Cl]$^+$ (for 3a-Ph) to [*iso*-4a-Ph]$^{2+}$ *vs.* [4a-Ph]$^{2+}$

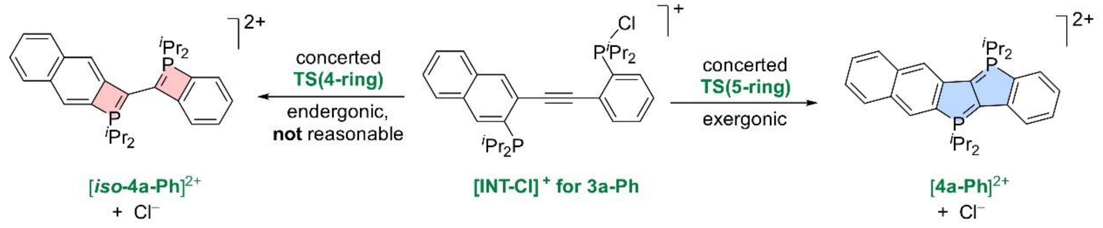

Note: energy diagram shown for P-chlorination at the Ph-side; similar energies were obtained for P-chlorination at the Nap-side

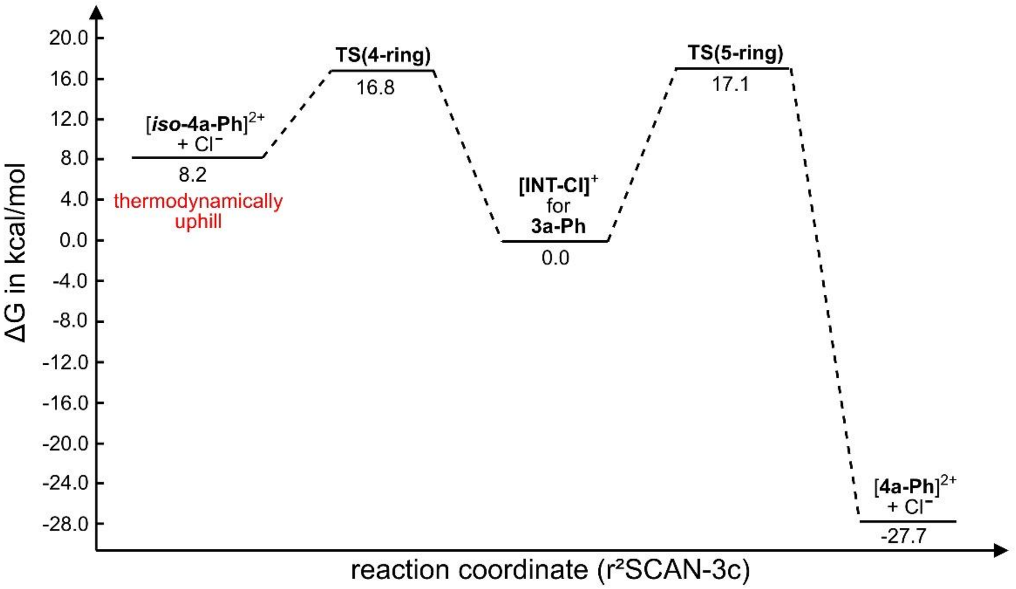

Figure S90. Gibbs free energy profile for the conversion of **[INT-Cl]**$^+$ (for **3a-Ph**) to [***iso*-4a-Ph**]$^{2+}$ and to [**4a-Ph**]$^{2+}$ (r^{2}SCAN-3c, def2-mTZVPP, D4, CPCM for CH_2Cl_2). The formation of [***iso*-4a-Ph**]$^{2+}$ is endergonic and therefore excluded.

Table S16. Gibbs free energies for the stationary points shown in the energy profile above (r^{2}SCAN-3c, def2-mTZVPP, D4, CPCM for CH_2Cl_2).

compound	Gibbs free energy (a.u.)	ΔG (rel. to most stable [INT-Cl]$^+$ for **3a-Ph**) (kcal/mol)
**[*iso*-4a-Ph]$^{2+}$** + Cl$^-$	-2307.92947814	**8.19**
TS(4-ring, P-Cl bond at Ph-side)	-2307.91579607	**16.80**
TS(4-ring, P-Cl bond atNap-side)	-2307.91582382	**16.76**
**[INT-Cl]**$^+$ (for **3a-Ph**), (P-Cl bond at Ph-side)	-2307.94253592	**0.00**
**[INT-Cl]**$^+$ (for **3a-Ph**) (P-Cl bond at Nap-side)	-2307.94249422	**0.03**
TS(5-ring, P-Cl bond at Ph-side)	-2307.91536423	**17.05**
TS(5-ring, P-Cl bond at Nap-side)	-2307.91482371	**17.39**
[**4a-Ph**]$^{2+}$ + Cl$^-$	-2307.98664271	**-27.68**

Cyclization of [INT-Cl]⁺ (for 3b-Ph) to [*iso*-4b-Ph]$^{2+}$ *vs.* [4b-Ph]$^{2+}$

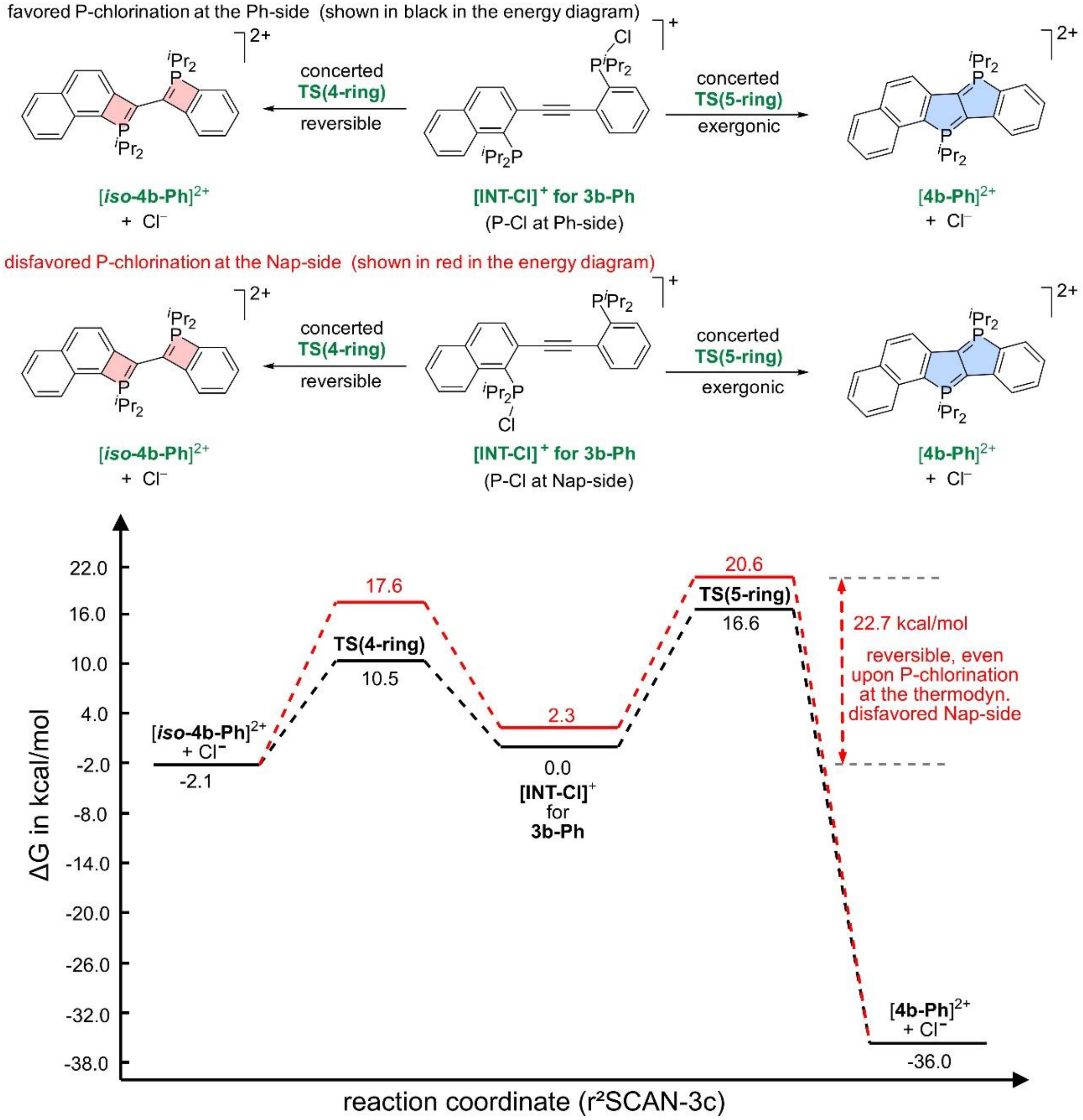

Figure S91. Gibbs free energy profile for the conversion of **[INT-Cl]⁺** (for **3b-Ph**) to [***iso*-4b-Ph**]$^{2+}$ and to [**4b-Ph**]$^{2+}$ (r^{2}SCAN-3c, def2-mTZVPP, D4, CPCM for CH_2Cl_2).

Table S17. Gibbs free energies for the stationary points shown in the energy profile above (r^{2}SCAN-3c, def2-mTZVPP, D4, CPCM for CH_2Cl_2).

compound	Gibbs free energy (a.u.)	ΔG (rel. to most stable [INT-Cl]⁺ for **3b-Ph**) (kcal/mol)
**[*iso*-4b-Ph]$^{2+}$** + Cl⁻	-2307.93533375	**-2.12**
TS(4-ring, P-Cl bond at Ph-side)	-2307.91523208	**10.49**
TS(4-ring, P-Cl bond at Nap-side)	-2307.90393109	**17.58**
**[INT-Cl]⁺** (for **3b-Ph**) (P-Cl bond at Ph-side)	-2307.93194656	**0.00**
**[INT-Cl]⁺** (for **3b-Ph**) (P-Cl bond at Nap-side)	-2307.92822620	**2.33**
TS(5-ring, P-Cl bond at Ph-side)	-2307.90545134	**16.63**
TS(5-ring, P-Cl bond at Nap-side)	-2307.89909942	**20.61**
[**4b-Ph**]$^{2+}$ + Cl⁻	-2307.98933230	**-36.01**

Cyclization of [INT-Cl]⁺ (for 3a) to [*iso*-4a]$^{2+}$ *vs.* [4a]$^{2+}$

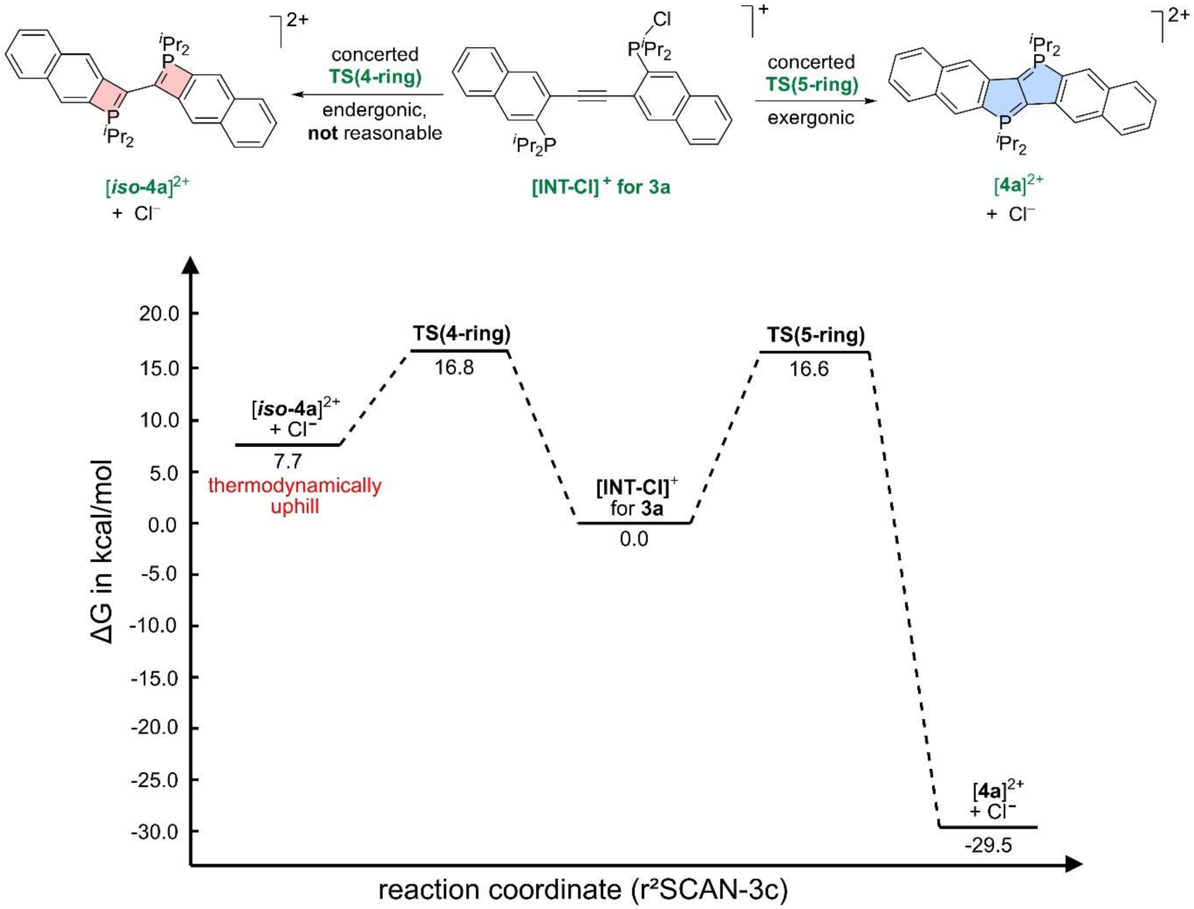

Figure S92. Gibbs free energy profile for the conversion of **[INT-Cl]⁺** (for **3a**) to [***iso*-4a**]$^{2+}$ and to [**4a**]$^{2+}$ (r^{2}SCAN-3c, def2-mTZVPP, D4, CPCM for CH_2Cl_2). The formation of [***iso*-4a**]$^{2+}$ is endergonic and therefore excluded.

Table S18. Gibbs free energies for the stationary points shown in the energy profile above (r^{2}SCAN-3c, def2-mTZVPP, D4, CPCM for CH_2Cl_2).

compound	Gibbs free energy (a.u.)	ΔG (rel. to [INT-Cl]⁺ for **3a**) (kcal/mol)
**[*iso*-4a]$^{2+}$** + Cl⁻	-2461.49956810	**7.66**
TS(4-ring)	-2461.48504242	**16.77**
**[INT-Cl]⁺** (for **3a**)	-2461.51177053	**0.00**
TS(5-ring)	-2461.48527368	**16.63**
[**4a**]$^{2+}$ + Cl⁻	-2461.55883514	**-29.53**

Cyclization of [INT-Cl]⁺ (for 3b) to [*iso*-4b]²⁺ *vs.* [4b]²⁺

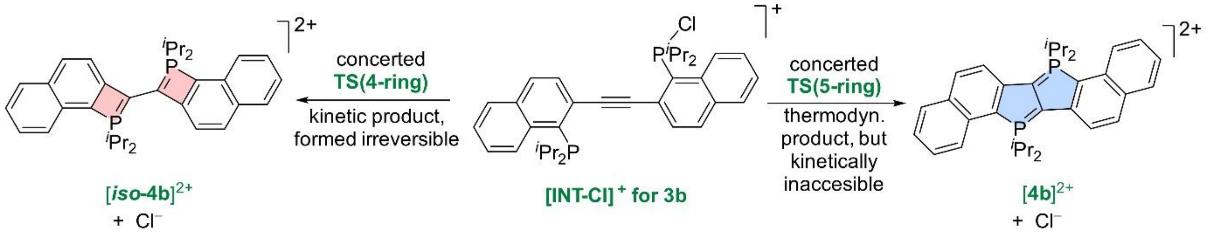

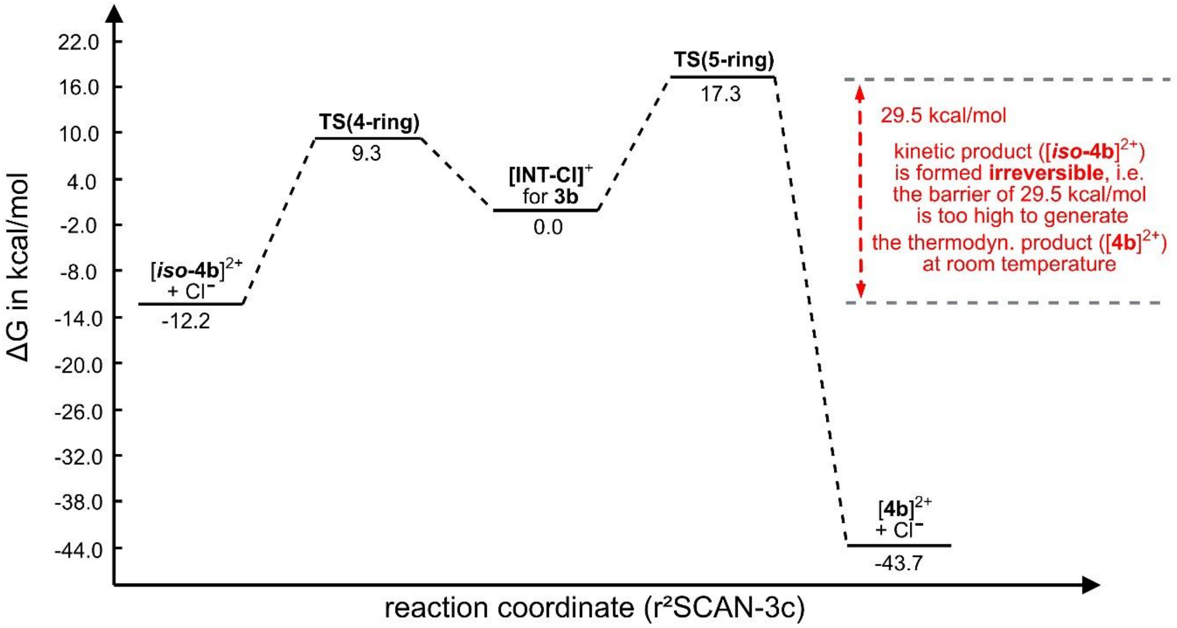

Figure S93. Gibbs free energy profile for the conversion of **[INT-Cl]⁺** (for **3b**) to [***iso*-4b**]²⁺ and to [**4b**]²⁺ (r²SCAN-3c, def2-mTZVPP, D4, CPCM for CH_2Cl_2). The experimentally observed product [***iso*-4a**]²⁺ is formed as the kinetic product. Once [***iso*-4b**]²⁺ is generated, it is impossible to overcome the barrier to the thermodynamic product ([**4b**]²⁺), suggesting that only [***iso*-4b**]²⁺ is formed at room temperature.

Table S19. Gibbs free energies for the stationary points shown in the energy profile above (r²SCAN-3c, def2-mTZVPP, D4, CPCM for CH_2Cl_2).

compound	Gibbs free energy (a.u.)	ΔG (rel. to [INT-Cl]⁺ for **3b**) (kcal/mol)
**[*iso*-4b]²⁺** + Cl⁻	-2461.50708266	**-12.19**
TS(4-ring)	-2461.47283417	**9.30**
**[INT-Cl]⁺** (for **3b**)	-2461.48765729	**0.00**
TS(5-ring)	-2461.46002371	**17.34**
[**4b**]²⁺ + Cl⁻	-2461.55725250	**-43.67**

Optimized Geometries for the Cyclization of [INT-Cl]$^{+}$ (for 3a-Ph) to [*iso*-4a-Ph]$^{2+}$ *vs.* [4a-Ph]$^{2+}$

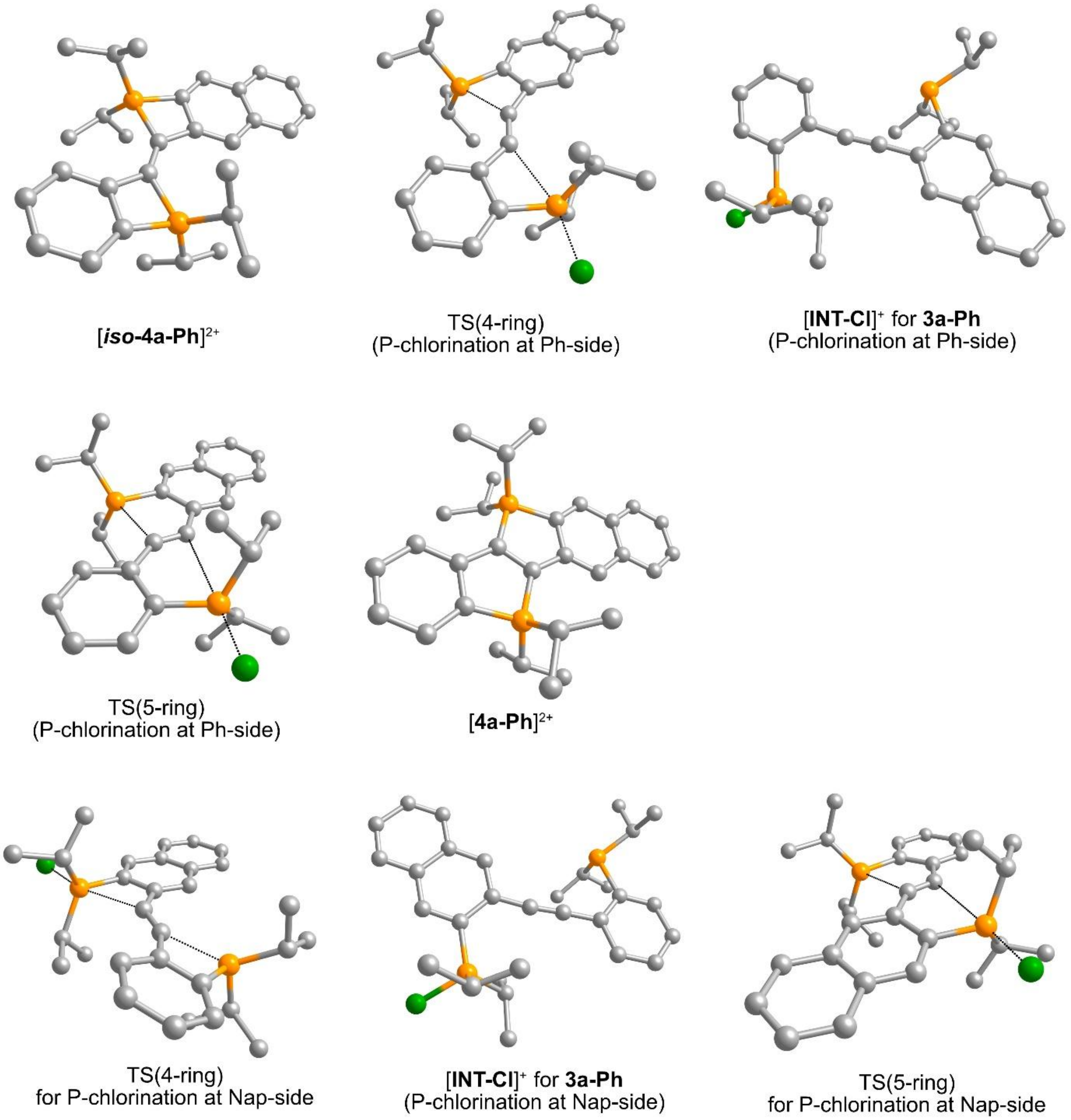

Figure S94. Optimized geometries (r^{2}SCAN-3c, def2-mTZVPP, D4, CPCM for CH_2Cl_2) for all compounds that may play a role in the cyclization of [**INT-Cl**]$^{+}$ (for **3a-Ph**) to [***iso*-4a-Ph**]$^{2+}$ or to [**4a-Ph**]$^{2+}$. Hydrogen atoms are omitted for clarity.

Optimized Geometries for the Cyclization of [INT-Cl]$^{+}$ (for 3b-Ph) to [*iso*-4b-Ph]$^{2+}$ *vs.* [4b-Ph]$^{2+}$

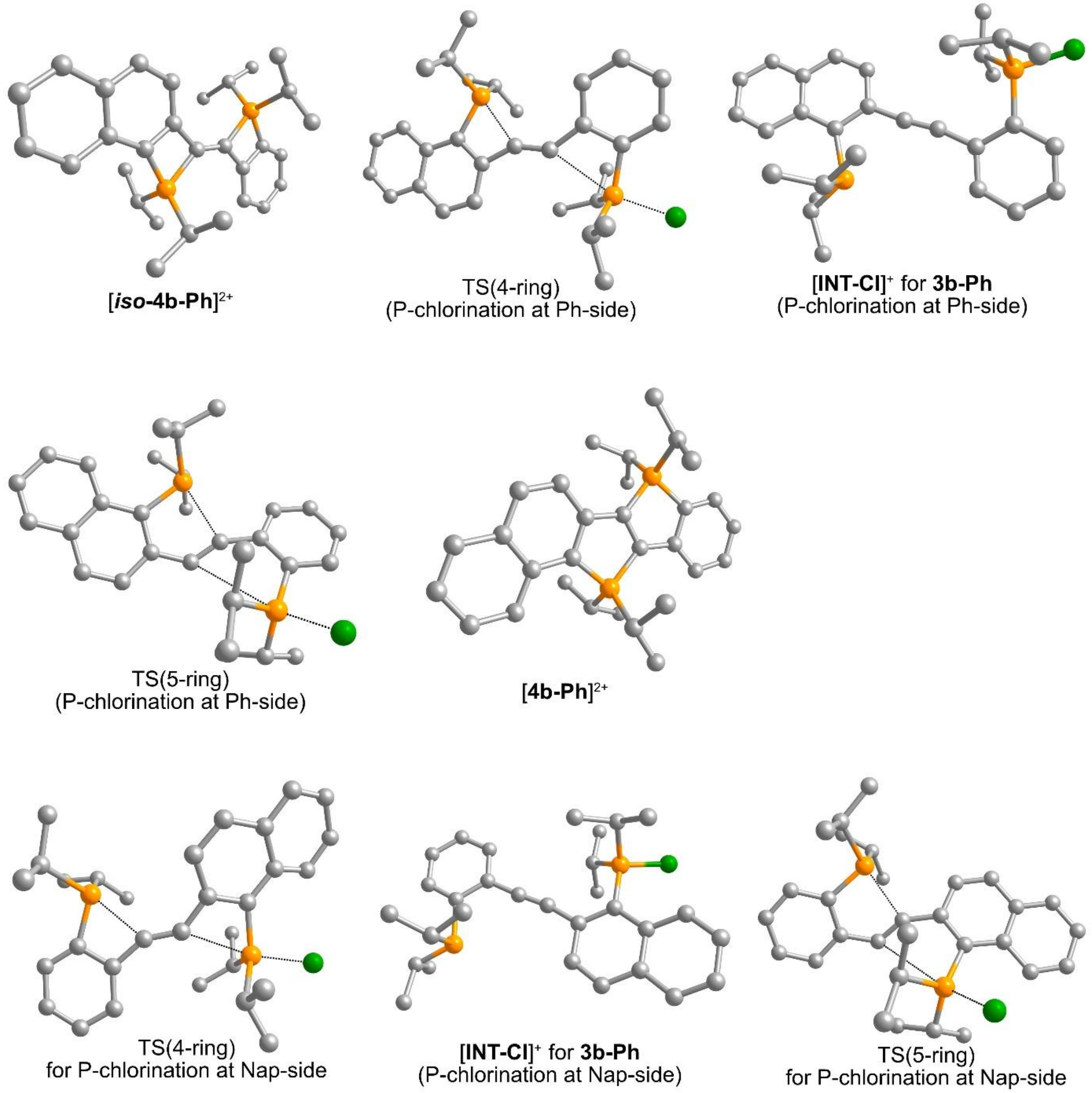

Figure S95. Optimized geometries (r^{2}SCAN-3c, def2-mTZVPP, D4, CPCM for CH_2Cl_2) for all compounds that may play a role in the cyclization of [**INT-Cl**]$^{+}$ (for **3b-Ph**) to [***iso*-4b-Ph**]$^{2+}$ or to [**4b-Ph**]$^{2+}$. Hydrogen atoms are omitted for clarity.

Optimized Geometries for the Cyclization of [INT-Cl]$^{+}$ (for 3a) to [*iso*-4a]$^{2+}$ *vs.* [4a]$^{2+}$

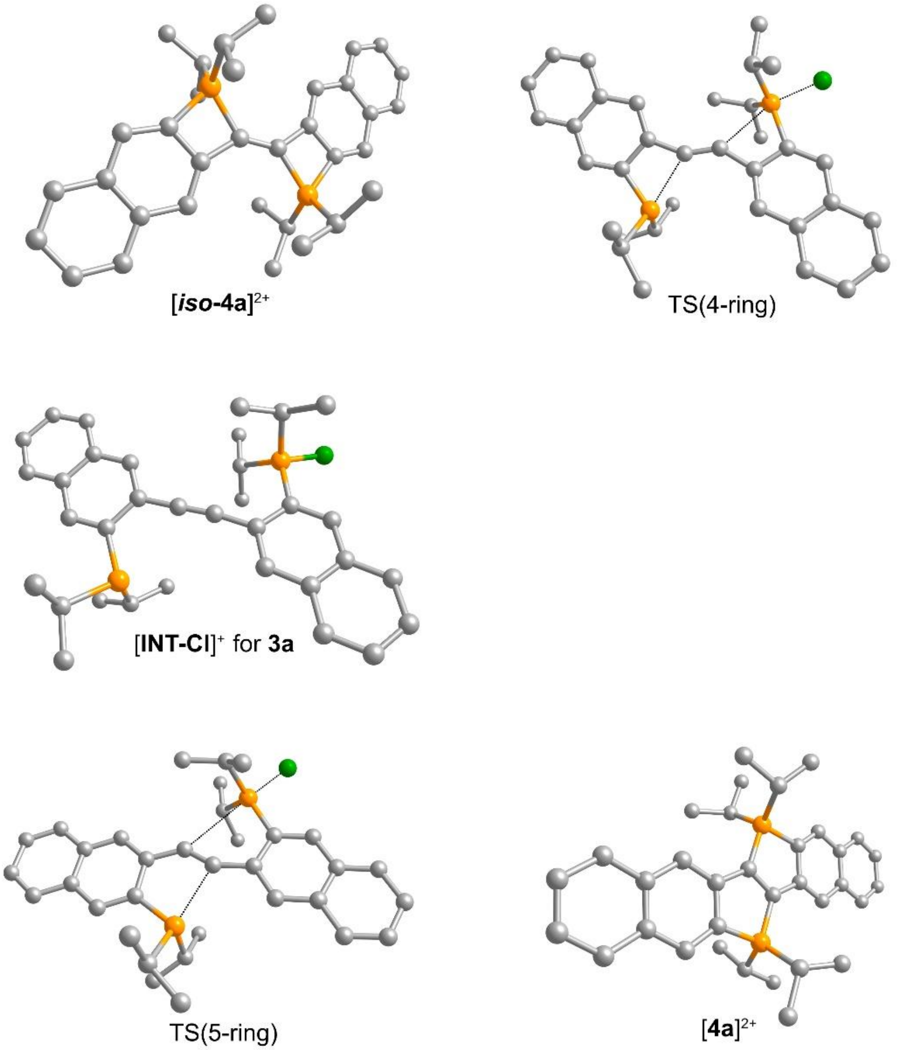

Figure S96. Optimized geometries (r^2SCAN-3c, def2-mTZVPP, D4, CPCM for CH_2Cl_2) for all compounds that may play a role in the cyclization of [**INT-Cl**]$^{+}$ (for **3a**) to [***iso*-4a**]$^{2+}$ or to [**4a**]$^{2+}$. Hydrogen atoms are omitted for clarity.

Optimized Geometries for the Cyclization of [INT-Cl]$^+$ (for 3b) to [*iso*-4b]$^{2+}$ *vs.* [4b]$^{2+}$

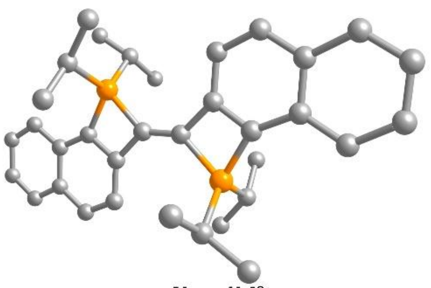

[***iso*-4b**]$^{2+}$

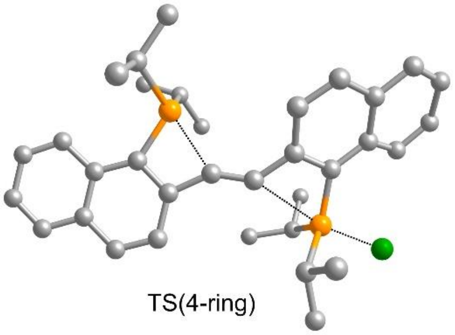

TS(4-ring)

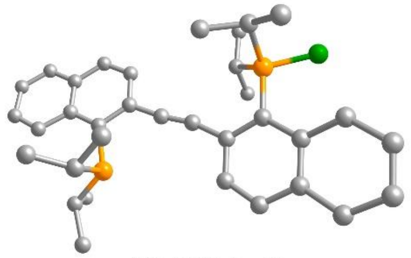

[**INT-Cl**]$^+$ for **3b**

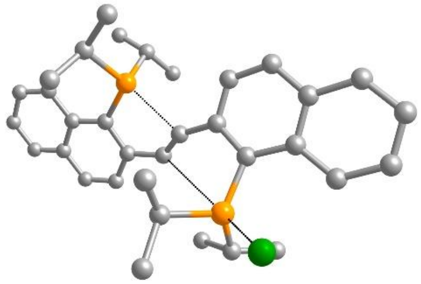

TS(5-ring)

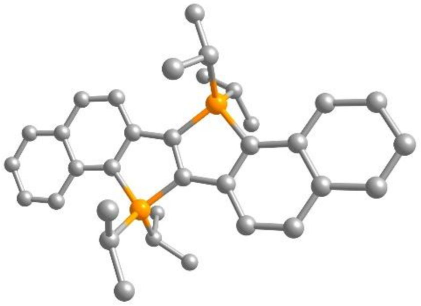

[**4b**]$^{2+}$

Figure S97. Optimized geometries (r^{2}SCAN-3c, def2-mTZVPP, D4, CPCM for CH_2Cl_2) for all compounds that may play a role in the cyclization of [**INT-Cl**]$^+$ (for **3b**) to [***iso*-4b**]$^{2+}$ or to [**4b**]$^{2+}$. Hydrogen atoms are omitted for clarity.

5.4) Computational Results for Mechanism C (Cyclization via a Radical Intermediate)

Radical Cyclization of 3a-Ph$^{+\bullet}$ to [4a-Ph]$^{+\bullet}$ (exp.: [4a-Ph]$^{2+}$) or to [*iso*-4a-Ph]$^{+\bullet}$ (hypothetical)

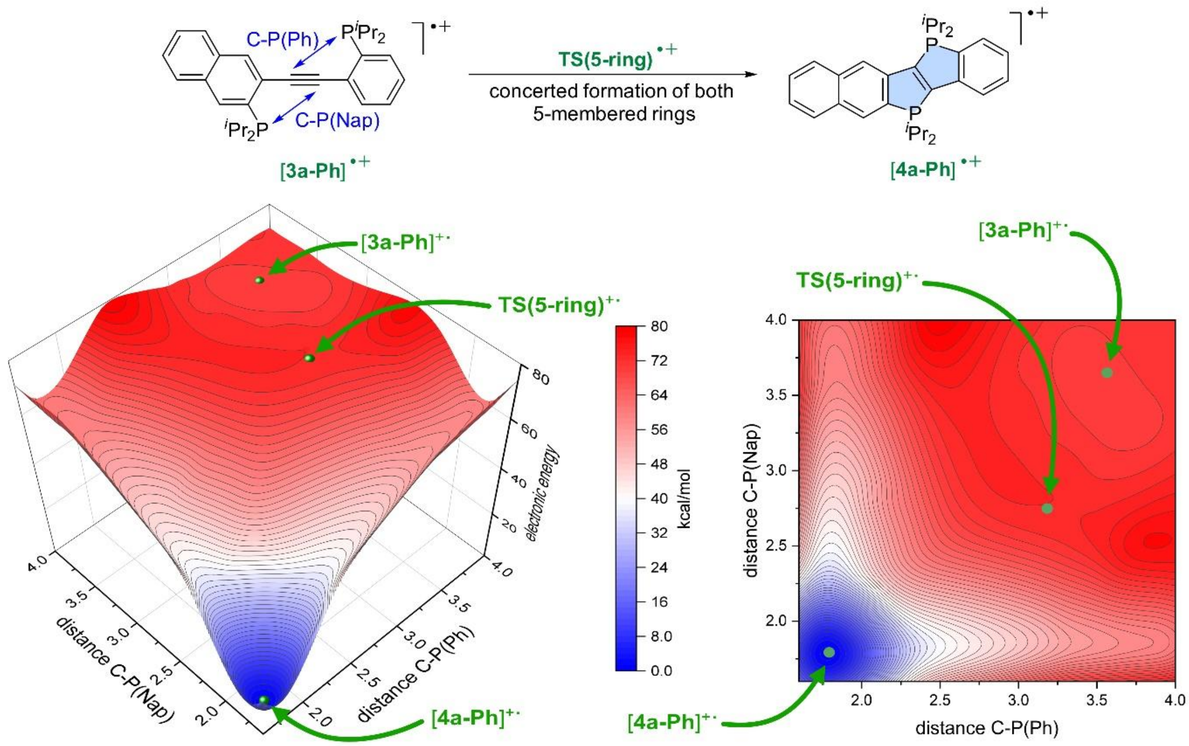

Figure S98. Relaxed potential energy surface scan (PES scan) along both C-P vectors (blue lines), which are contracted upon formation of [**4a-Ph**]$^{+\bullet}$ starting from [**3a-Ph**]$^{+\bullet}$ (r^{2}SCAN-3c, def2-mTZVPP, D4, CPCM for CH_2Cl_2). Note that electronic energies are provided in z-direction, while Gibbs free energies were obtained for the stationary points by means of frequency calculations.

Table S20. Gibbs free energies for the stationary points (green dots) in the relaxed PES shown above (r^{2}SCAN-3c, def2-mTZVPP, D4, CPCM for CH_2Cl_2).

compound	Gibbs free energy (a.u.)	ΔG (rel. to [**3a-Ph**]$^{+\bullet}$) (kcal/mol)	ΔG (rel. to [**4a-Ph**]$^{+\bullet}$) (kcal/mol)
[**3a-Ph**]$^{+\bullet}$	-1847.6938569	**0.00**	55.72
TS(5-ring)$^{+\bullet}$	-1847.6880875	**3.62**	59.34
[**4a-Ph**]$^{+\bullet}$	-1847.7826508	**-55.72**	0.00

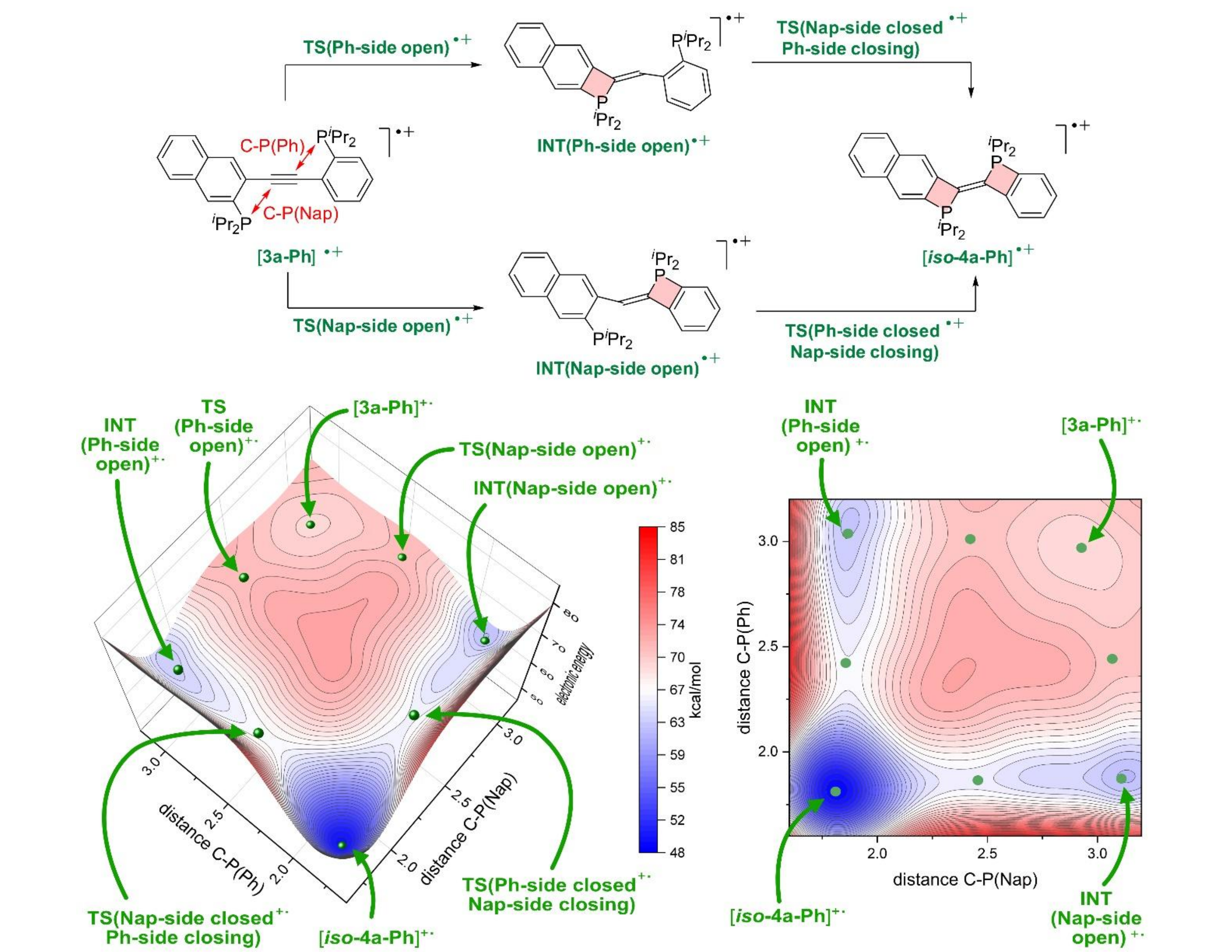

Figure S99. Relaxed potential energy surface scan (PES scan) along both C-P vectors (red lines), which are contracted upon formation of [***iso*-4a-Ph**]$^{+\bullet}$ starting from [**3a-Ph**]$^{+\bullet}$ (r^{2}SCAN-3c, def2-mTZVPP, D4, CPCM for CH_2Cl_2). Note that electronic energies are provided in z-direction, while Gibbs free energies were obtained for the stationary points by means of frequency calculations.

Table S21. Gibbs free energies for the stationary points (green dots) in the relaxed PES shown above (r^{2}SCAN-3c, def2-mTZVPP, D4, CPCM for CH_2Cl_2).

compound	Gibbs free energy (a.u.)	ΔG (rel. to [**3a-Ph**]$^{+\bullet}$) (kcal/mol)	ΔG (rel. to [**4a-Ph**]$^{+\bullet}$) (kcal/mol)
[**3a-Ph**]$^{+\bullet}$	-1847.6938569	**0.00**	55.72
TS(Nap-side open)$^{+\bullet}$	-1847.6930724	**0.49**	56.21
INT(Nap-side open)$^{+\bullet}$	-1847.7046179	**-6.75**	48.97
TS(Ph-side open)$^{+\bullet}$	-1847.6918234	**1.28**	57.00
INT(Ph-side open)$^{+\bullet}$	-1847.7054622	**-7.28**	48.44
TS(Ph-side closed Nap-side closing) $^{+\bullet}$	-1847.6985494	**-2.94**	52.77
TS(Nap-side closed Ph-side closing) $^{+\bullet}$	-1847.6988080	**-3.11**	52.61
[***iso*-4a-Ph**]$^{+\bullet}$	-1847.7197135	**-16.23**	39.49

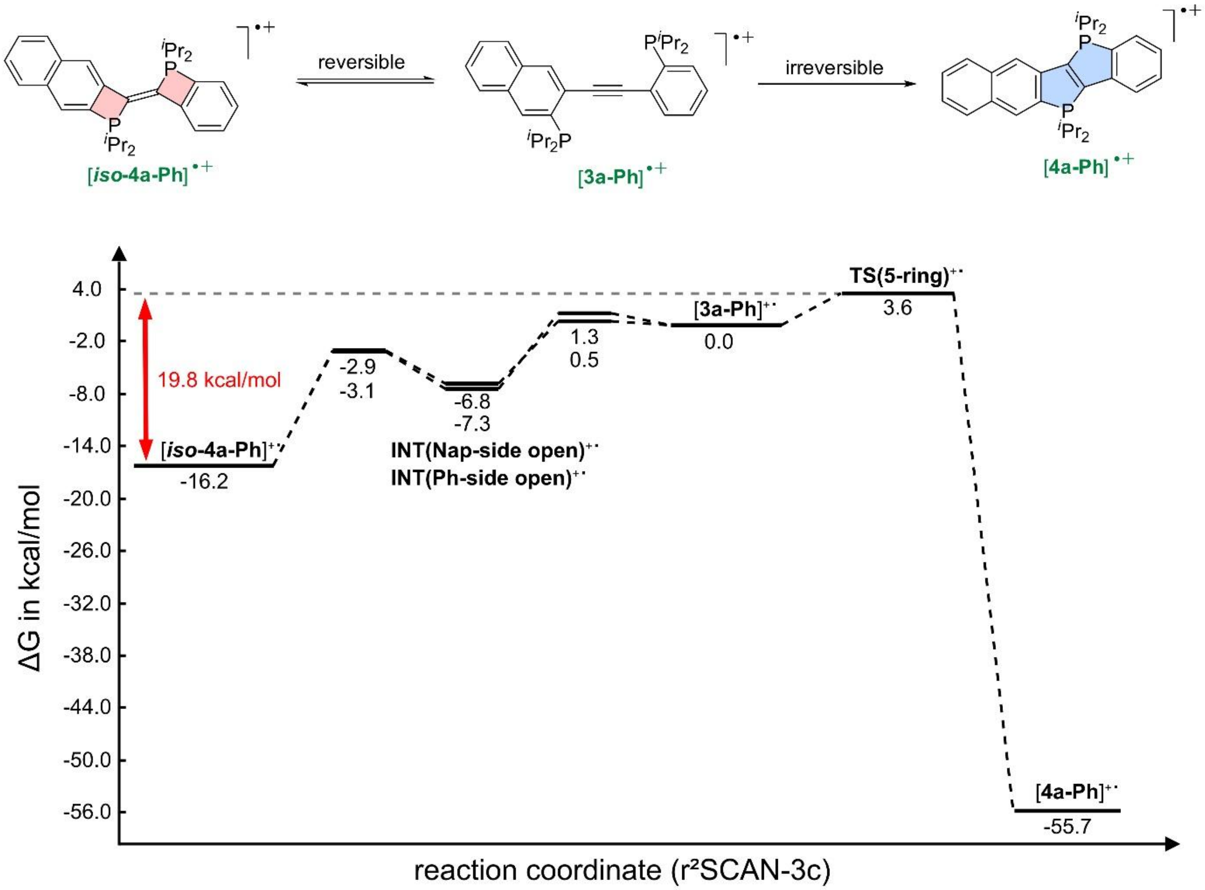

Figure S100. Gibbs free energy profile for the conversion of [**3a-Ph**]$^{+•}$ to [***iso*-4a-Ph**]$^{+•}$ and to [**4a-Ph**]$^{+•}$ (r^{2}SCAN-3c, def2-mTZVPP, D4, CPCM for CH_2Cl_2). [***iso*-4a-Ph**]$^{+•}$ is formed as the kinetic product (lower-lying transition states), but the overall barrier between [***iso*-4a-Ph**]$^{+•}$ and **TS(5-ring)** $^{+•}$ (19.8 kcal/mol) is readily overcome at room temperature. Hence, [**4a-Ph**]$^{+•}$ is eventually formed irreversibly (thermodynamic product).

Note: The possibility that [***iso*-4a-Ph**]$^{+•}$ may be converted *directly* to [**4a-Ph**]$^{+•}$ via a concerted shift of both C-P bonds was also considered. Nudged elastic band (NEB) calculations, however, suggested a *minimum* energy path (MEP) via [**3a-Ph**]$^{+•}$, i.e. the mechanism shown in reaction profile (above) seems to be the most reasonable possibility on the S = ½ surface.

Radical Cyclization of 3b-Ph$^{+\bullet}$ to [4b-Ph]$^{+\bullet}$ (exp.: [4b-Ph]$^{2+}$) or to [*iso*-4b-Ph]$^{+\bullet}$ (hypothetical)

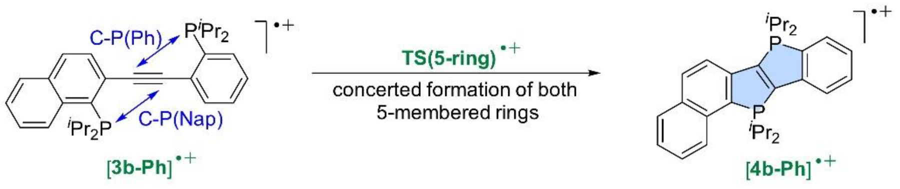

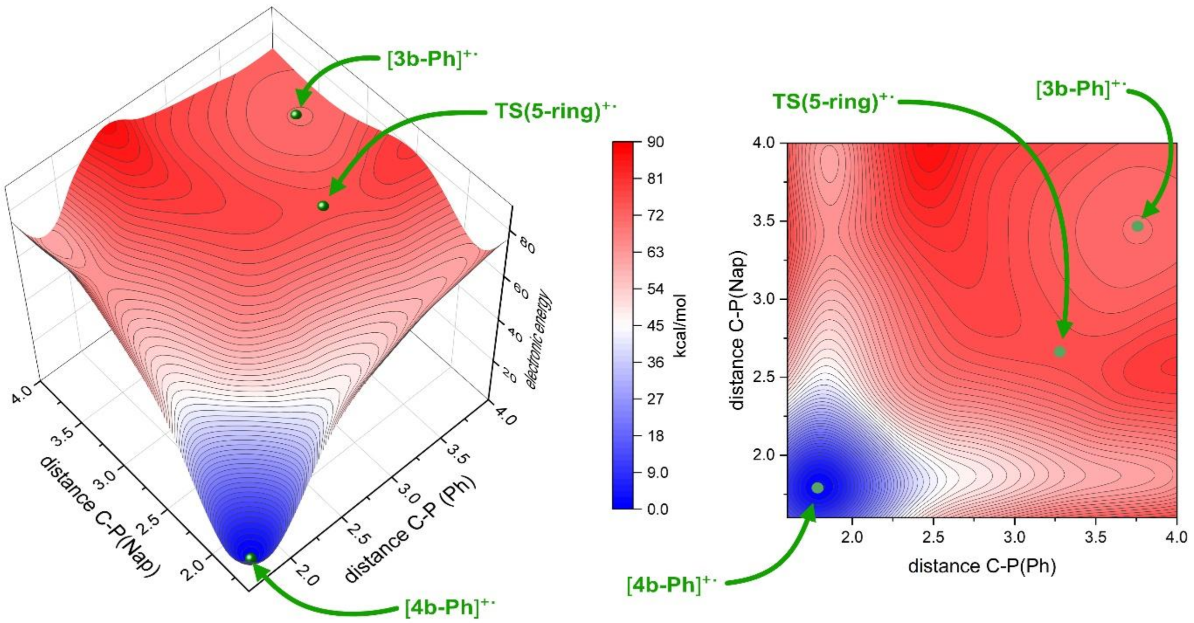

Figure S101. Relaxed potential energy surface scan (PES scan) along both C-P vectors (blue lines), which are contracted upon formation of [**4a-Ph**]$^{+\bullet}$ starting from [**3a-Ph**]$^{+\bullet}$ (r^{2}SCAN-3c, def2-mTZVPP, D4, CPCM for CH_2Cl_2). Note that electronic energies are provided in z-direction, while Gibbs free energies were obtained for the stationary points by means of frequency calculations.

Table S22. Gibbs free energies for the stationary points (green dots) in the relaxed PES shown above (r^{2}SCAN-3c, def2-mTZVPP, D4, CPCM for CH_2Cl_2).

compound	Gibbs free energy (a.u.)	ΔG (rel. to [**3b-Ph**]$^{+\bullet}$) (kcal/mol)	ΔG (rel. to [**4b-Ph**]$^{+\bullet}$) (kcal/mol)
[**3b-Ph**]$^{+\bullet}$	-1847.6922223	**0.00**	62.80
TS(5-ring)$^{+\bullet}$	-1847.6822310	**6.27**	69.07
[**4b-Ph**]$^{+\bullet}$	-1847.7923013	**-62.80**	0.00

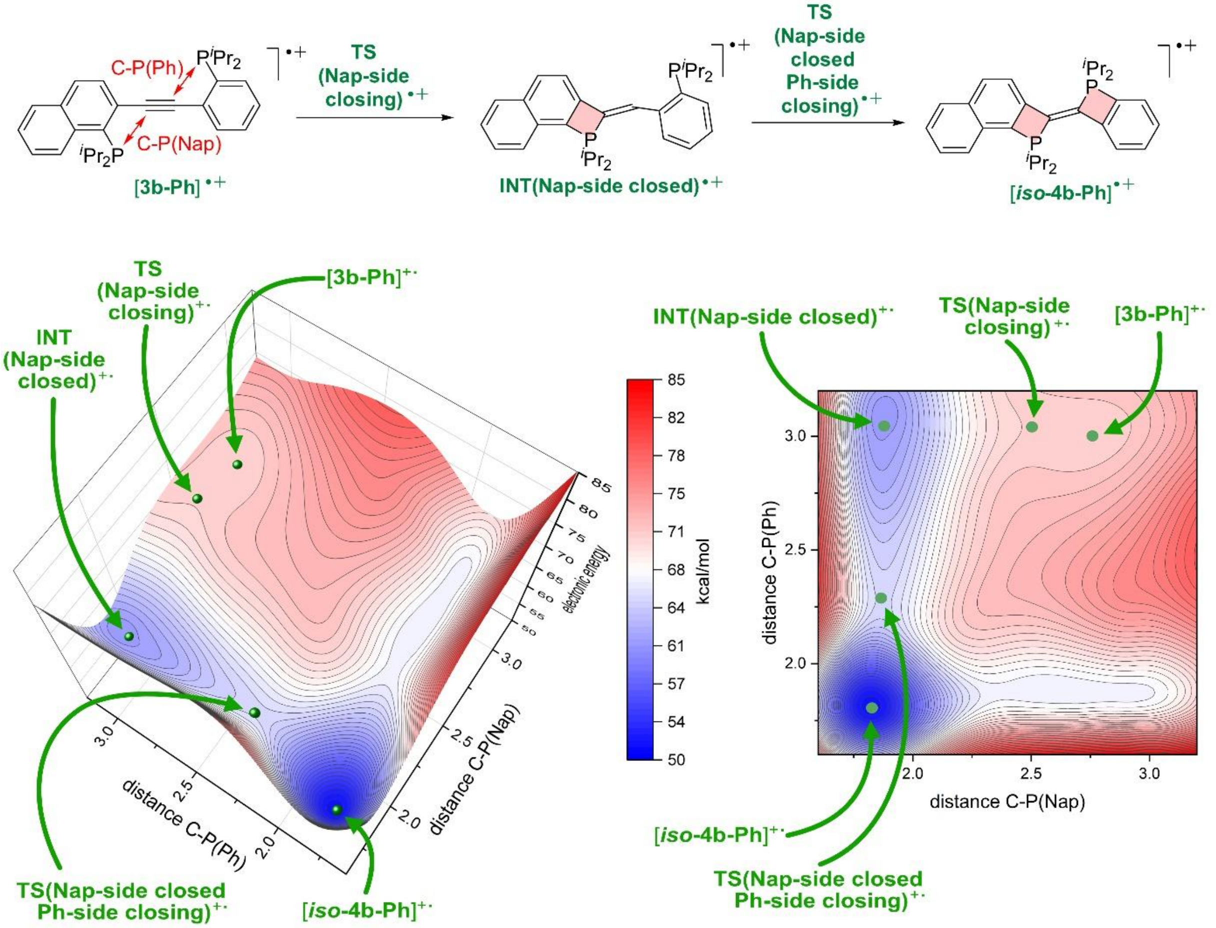

Figure S102. Relaxed potential energy surface scan (PES scan) along both C-P vectors (red lines), which are contracted upon formation of [***iso*-4b-Ph**]$^{+\bullet}$ starting from [**3b-Ph**]$^{+\bullet}$ (r^2SCAN-3c, def2-mTZVPP, D4, CPCM for CH_2Cl_2). Note that electronic energies are provided in z-direction, while Gibbs free energies were obtained for the stationary points by means of frequency calculations.

Table S23. Gibbs free energies for the stationary points (green dots) in the relaxed PES shown above (r^2SCAN-3c, def2-mTZVPP, D4, CPCM for CH_2Cl_2).

compound	Gibbs free energy (a.u.)	ΔG (rel. to [**3b-Ph**]$^{+\bullet}$) (kcal/mol)	ΔG (rel. to [**4b-Ph**]$^{+\bullet}$) (kcal/mol)
[**3b-Ph**]$^{+\bullet}$	-1847.6922223	**0.00**	62.80
TS(Nap-side closing)$^{+\bullet}$	-1847.6921745	**0.03**	62.83
INT(Nap-side closed$^{+\bullet}$	-1847.7034992	**-7.08**	55.72
TS(Nap-side closed Ph-side closing) $^{+\bullet}$	-1847.6986577	**-4.04**	58.76
[***iso*-4b-Ph**]$^{+\bullet}$	-1847.7197135	**-17.25**	45.55

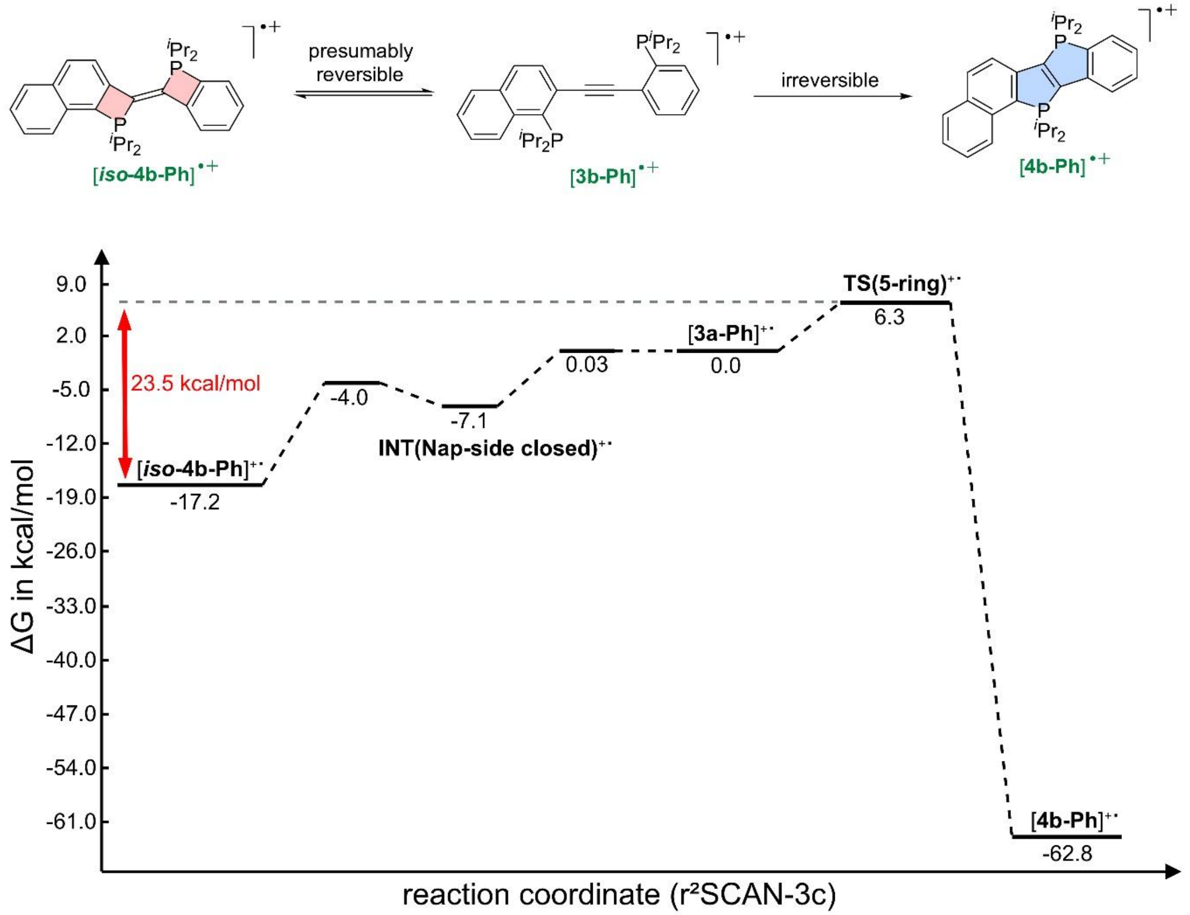

Figure S103. Gibbs free energy profile for the conversion of [**3b-Ph**]$^{+\bullet}$ to [***iso***-**4b-Ph**]$^{+\bullet}$ and to [**4b-Ph**]$^{+\bullet}$ (r^{2}SCAN-3c, def2-mTZVPP, D4, CPCM for CH_2Cl_2). [***iso***-**4b-Ph**]$^{+\bullet}$ is formed as the kinetic product (lower-lying transition states), but the overall barrier between [***iso***-**4b-Ph**]$^{+\bullet}$ and **TS(5-ring)** $^{+\bullet}$ (23.5 kcal/mol) *may* still be overcome at room temperature. Therefore, it is assumed that [***iso***-**4b-Ph**]$^{+\bullet}$ is generated reversible, while [**4b-Ph**]$^{+\bullet}$ is certainly formed irreversibly (thermodynamic product).

Note: The possibility that [***iso***-**4b-Ph**]$^{+\bullet}$ may be converted *directly* to [**4b-Ph**]$^{+\bullet}$ via a concerted shift of both C-P bonds was also considered. Nudged elastic band (NEB) calculations, however, suggested a *minimum* energy path (MEP) via [**3b-Ph**]$^{+\bullet}$, i.e. the mechanism shown in reaction profile (above) seems to be the most reasonable possibility on the S = ½ surface.

Radical Cyclization of 3a$^{+\bullet}$ to [4a]$^{+\bullet}$ (exp.:[4a]$^{2+}$) or to [*iso*-4a]$^{+\bullet}$ (hypothetical)

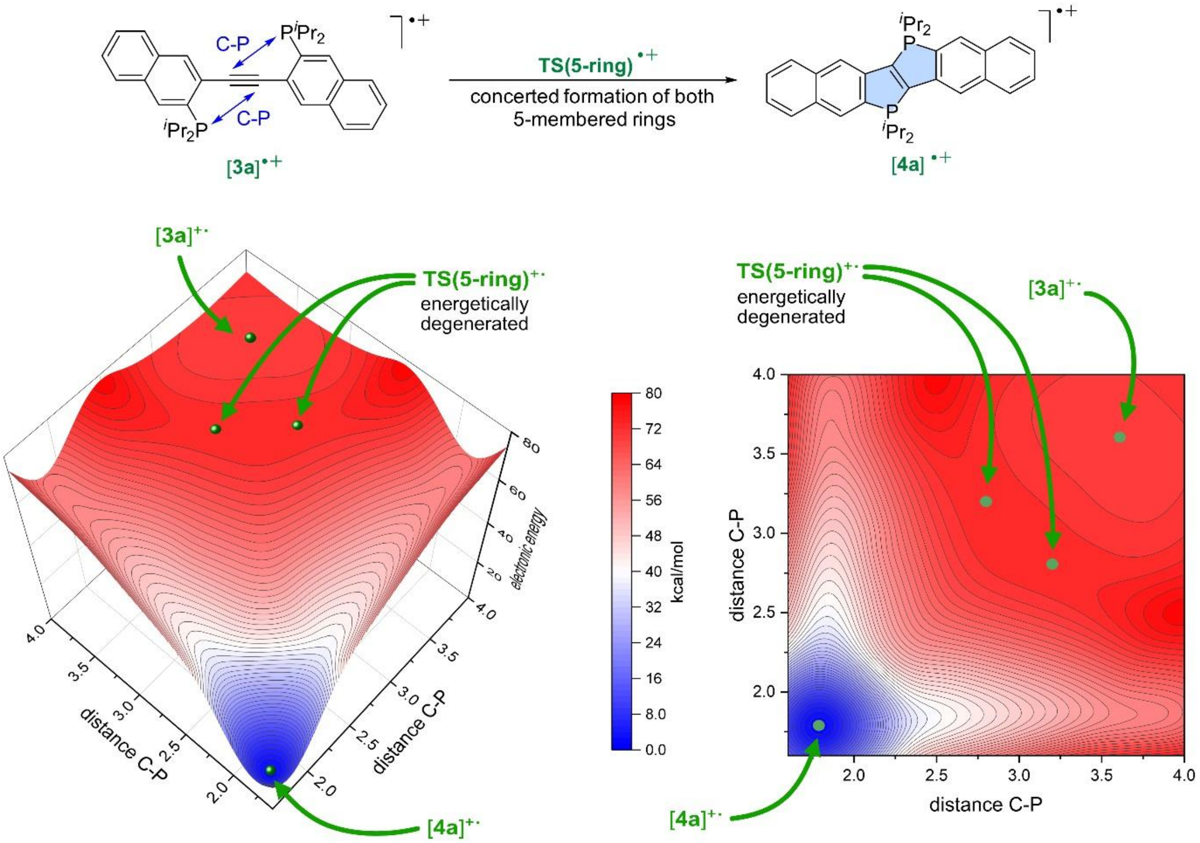

Figure S104. Relaxed potential energy surface scan (PES scan) along both C-P vectors (blue lines), which are contracted upon formation of [**4a**]$^{+\bullet}$ starting from [**3a**]$^{+\bullet}$ (r^{2}SCAN-3c, def2-mTZVPP, D4, CPCM for CH_2Cl_2). Note that electronic energies are provided in z-direction, while Gibbs free energies were obtained for the stationary points by means of frequency calculations.

Table S24. Gibbs free energies for the stationary points (green dots) in the relaxed PES shown above (r^{2}SCAN-3c, def2-mTZVPP, D4, CPCM for CH_2Cl_2).

compound	Gibbs free energy (a.u.)	ΔG (rel. to [**3a**]$^{+\bullet}$) (kcal/mol)	ΔG (rel. to [**4a**]$^{+\bullet}$) (kcal/mol)
[**3a**]$^{+\bullet}$	-2001.2631150	**0.00**	55.70
TS(5-ring)$^{+\bullet}$	-2001.2581202	**3.13**	58.83
[**4a**]$^{+\bullet}$	-2001.3518730	**-55.70**	0.00

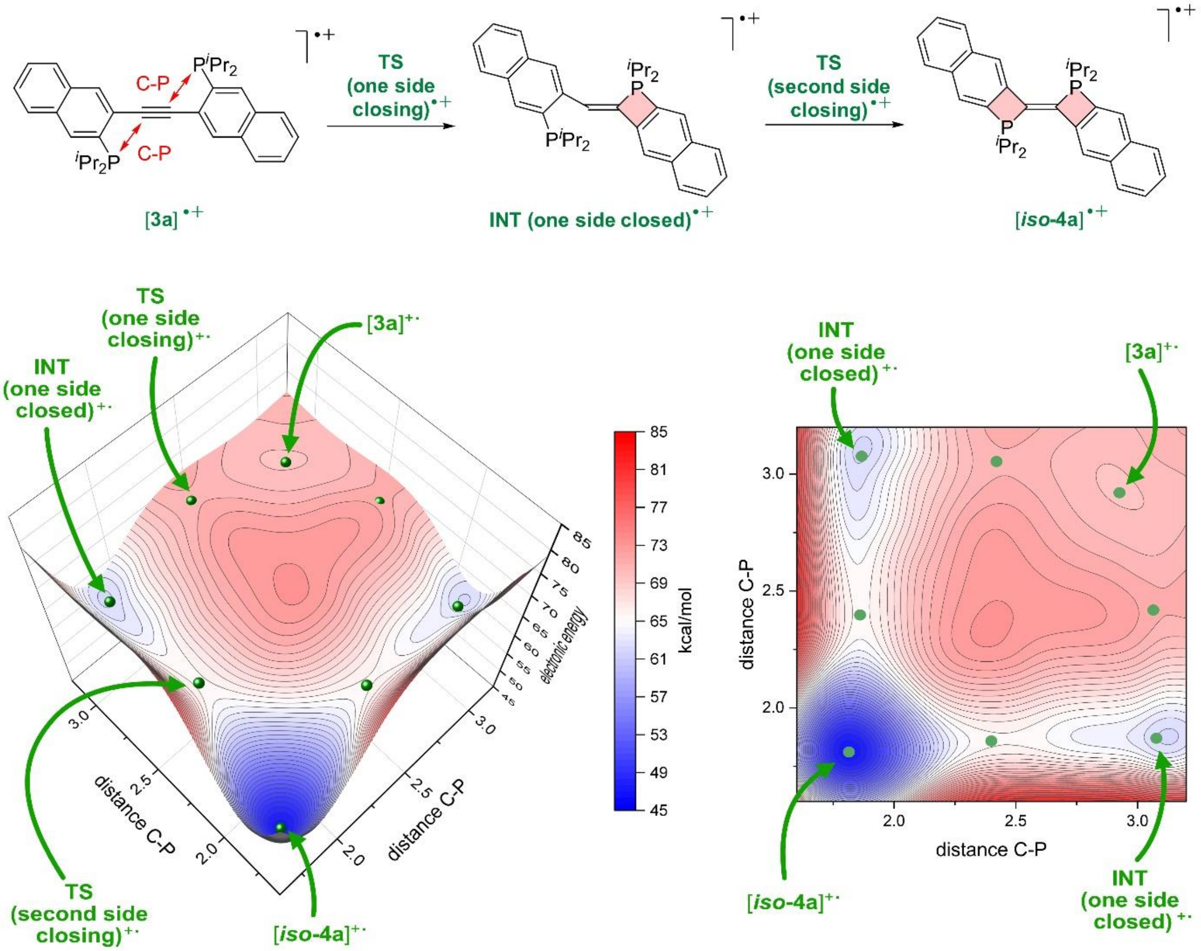

Figure S105. Relaxed potential energy surface scan (PES scan) along both C-P vectors (red lines), which are contracted upon formation of [***iso*-4a**]$^{+\bullet}$ starting from [**3a**]$^{+\bullet}$ (r^2SCAN-3c, def2-mTZVPP, D4, CPCM for CH_2Cl_2). Note that electronic energies are provided in z-direction, while Gibbs free energies were obtained for the stationary points by means of frequency calculations.

Table S25. Gibbs free energies for the stationary points (green dots) in the relaxed PES shown above (r^2SCAN-3c, def2-mTZVPP, D4, CPCM for CH_2Cl_2).

compound	Gibbs free energy (a.u.)	ΔG (rel. to [**3a**]$^{+\bullet}$) (kcal/mol)	ΔG (rel. to [**4a**]$^{+\bullet}$) (kcal/mol)
[**3a**]$^{+\bullet}$	-2001.2631150	**0.00**	55.70
TS(one side closing)$^{+\bullet}$	-2001.2617706	**0.84**	56.54
INT(one side closed)$^{+\bullet}$	-2001.2738760	**-6.75**	48.94
TS(second side closing $^{+\bullet}$	-2001.2681673	**-3.17**	52.53
[***iso*-4a**]$^{+\bullet}$	-2001.2962849	**-20.81**	34.88

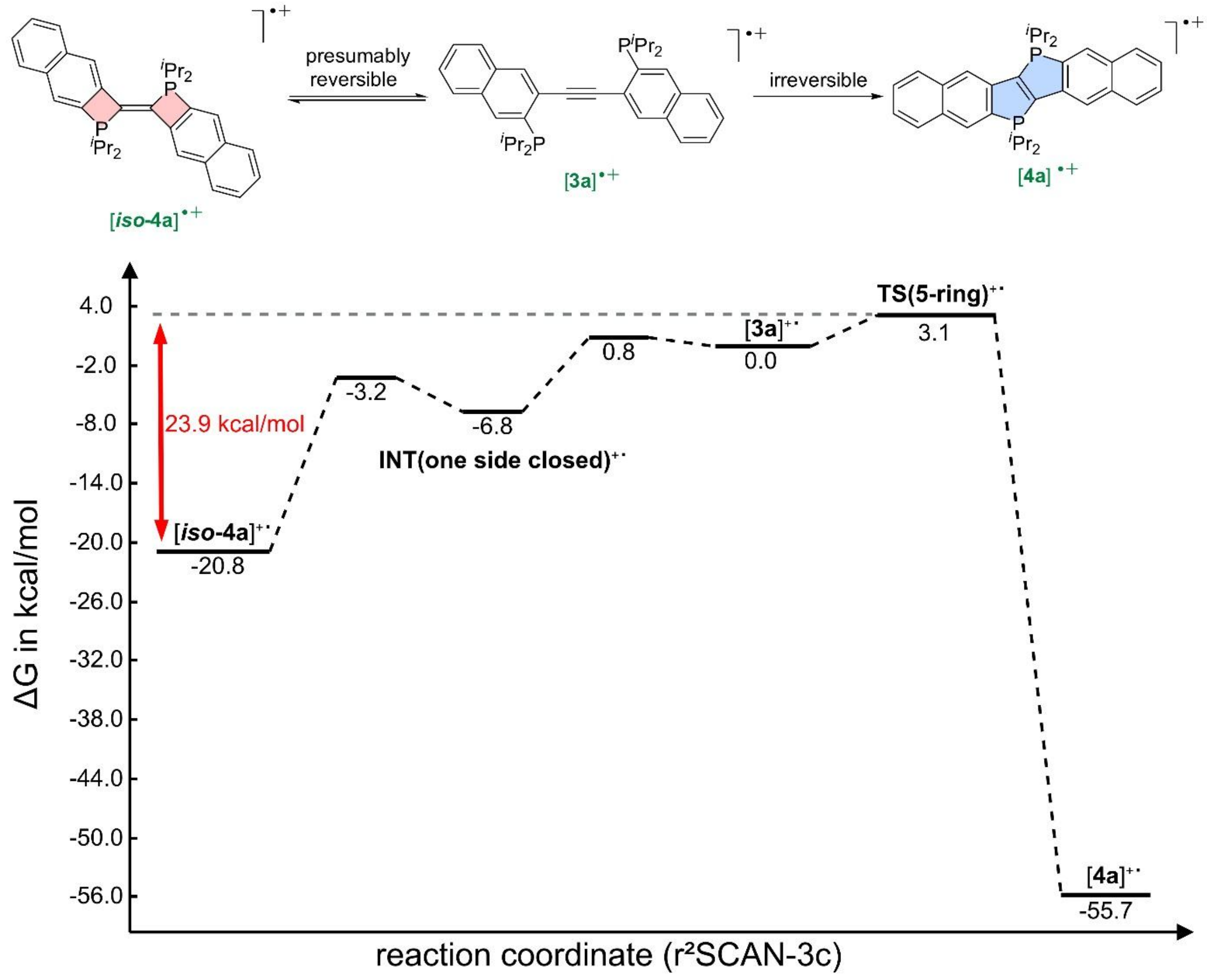

Figure S106. Gibbs free energy profile for the conversion of [**3a**]$^{+\bullet}$ to [***iso*-4a**]$^{+\bullet}$ and to [**4a**]$^{+\bullet}$ (r^2SCAN-3c, def2-mTZVPP, D4, CPCM for CH_2Cl_2). [***iso*-4a**]$^{+\bullet}$ is formed as the kinetic product (lower-lying transition states), but the overall barrier between [***iso*-4a**]$^{+\bullet}$ and **TS(5-ring)** $^{+\bullet}$ (23.5 kcal/mol) *may* still be overcome at room temperature. Therefore, it is assumed that [***iso*-4a**]$^{+\bullet}$ is generated reversibly, while [**4a**]$^{+\bullet}$ is certainly formed irreversibly (thermodynamic product).

Note: The possibility that [***iso*-4a**]$^{+\bullet}$ may be converted *directly* to [**4a**]$^{+\bullet}$ via a concerted shift of both C-P bonds was also considered. Nudged elastic band (NEB) calculations, however, suggested a *minimum* energy path (MEP) via [**3a**]$^{+\bullet}$, i.e. the mechanism shown in reaction profile (above) seems to be the most reasonable possibility on the S = ½ surface.

Formation of [*iso*-4b]$^{+\bullet}$ (exp.: [iso-4b]$^{2+}$) versus Formation of [4b]$^{+\bullet}$

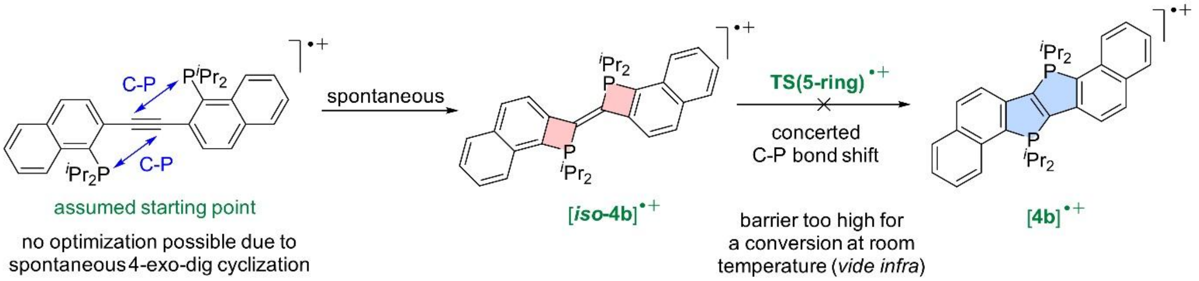

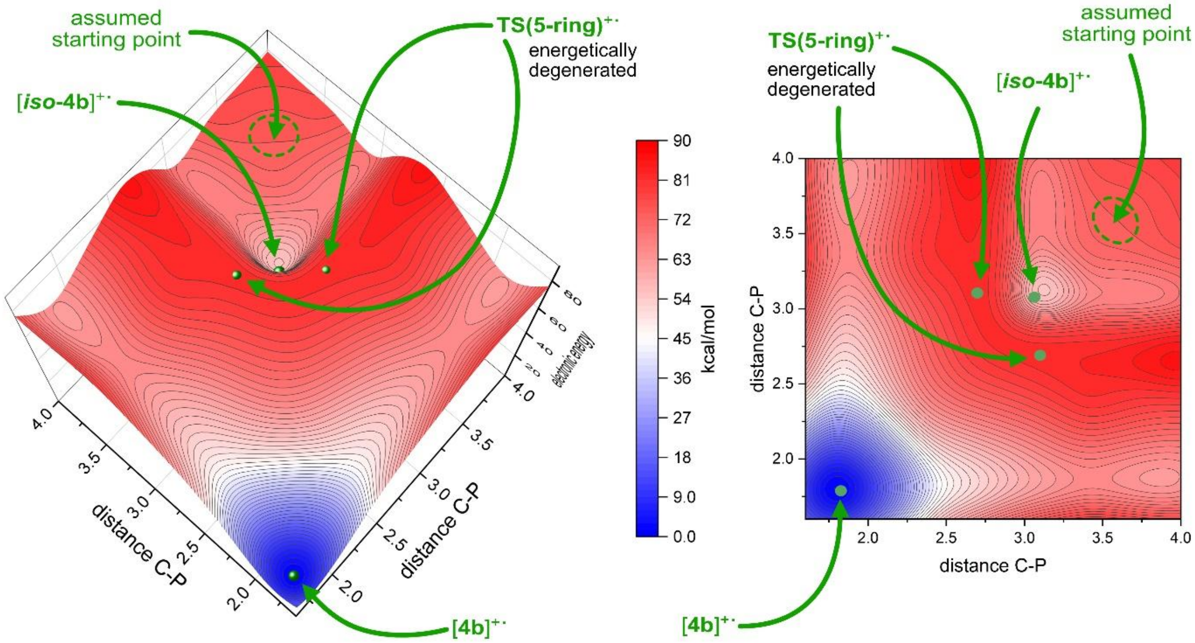

Figure S107. Relaxed potential energy surface scan (PES scan) along both C-P vectors (blue lines), which are contracted upon formation of [**4b**]$^{+\bullet}$ (r^2SCAN-3c, def2-mTZVPP, D4, CPCM for CH_2Cl_2). Note that electronic energies are provided in z-direction, while Gibbs free energies were obtained for the stationary points by means of frequency calculations.

Table S26. Gibbs free energies for the stationary points (green dots) in the relaxed PES shown above (r^2SCAN-3c, def2-mTZVPP, D4, CPCM for CH_2Cl_2).

compound	Gibbs free energy (a.u.)	ΔG (rel. to [***iso*-4b**]$^{+\bullet}$) (kcal/mol)	ΔG (rel. to [**4b**]$^{+\bullet}$) (kcal/mol)
[***iso*-4b**]$^{+\bullet}$	-2001.2903472	**0.00**	44.87
TS(5-ring)$^{+\bullet}$	-2001.2426157	**29.95**	74.82
[**4b**]$^{+\bullet}$	-2001.3618503	**-44.87**	0.00

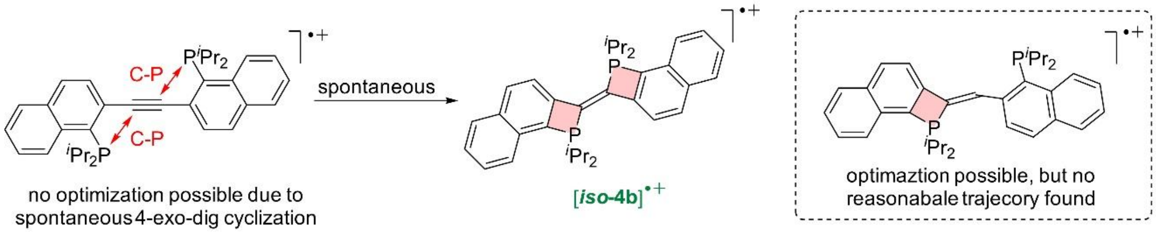

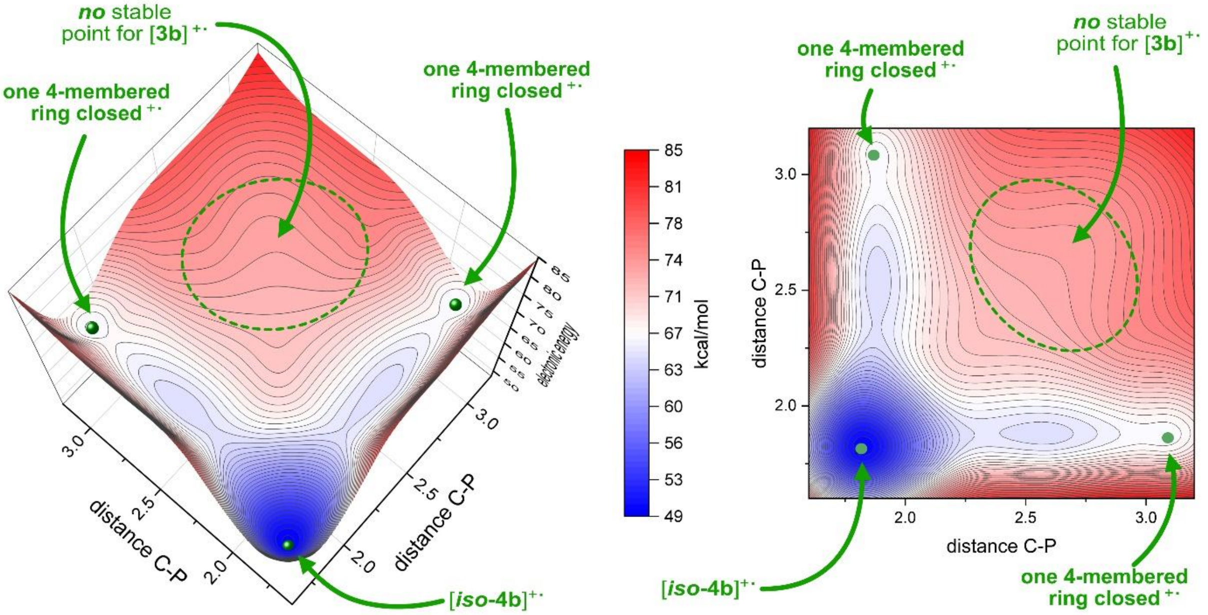

Figure S108. Relaxed potential energy surface scan (PES scan) along both C-P vectors (red lines), which are contracted upon formation of [***iso*-4b**]+• (r^{2}SCAN-3c, def2-mTZVPP, D4, CPCM for CH_2Cl_2). Note that electronic energies are provided in z-direction, while Gibbs free energies were obtained for the stationary points by means of frequency calculations.

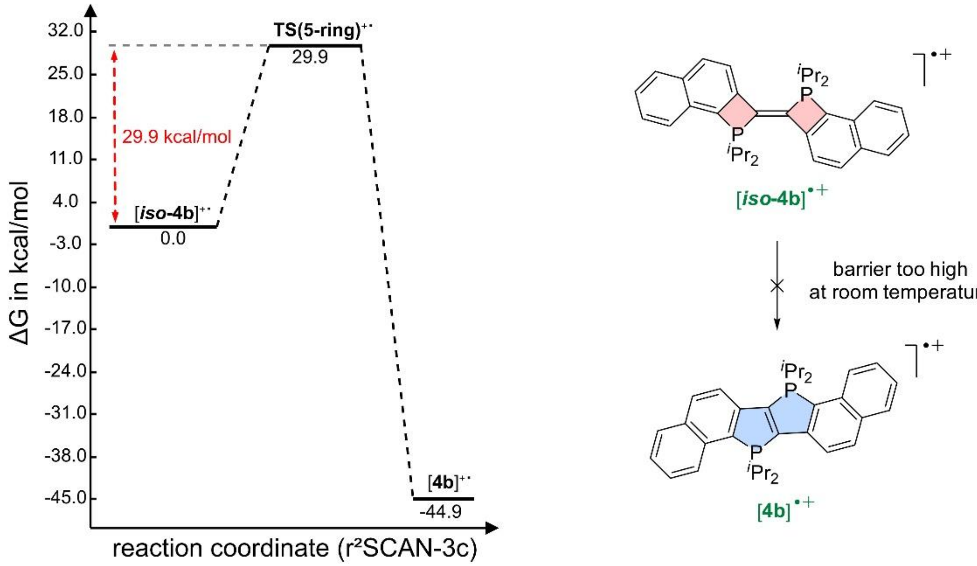

Figure S109. Gibbs free energy profile for the conversion of [***iso*-4b**]+• and to [**4b**]+• (r^{2}SCAN-3c, def2-mTZVPP, D4, CPCM for CH_2Cl_2). The barrier of 29.9 kcal/mol is too high for a conversion at room temperature. The shown reaction pathway is consistent with NEB calculations.

Optimized Geometries for the Radical Cyclization of 3a-Ph$^{+\bullet}$

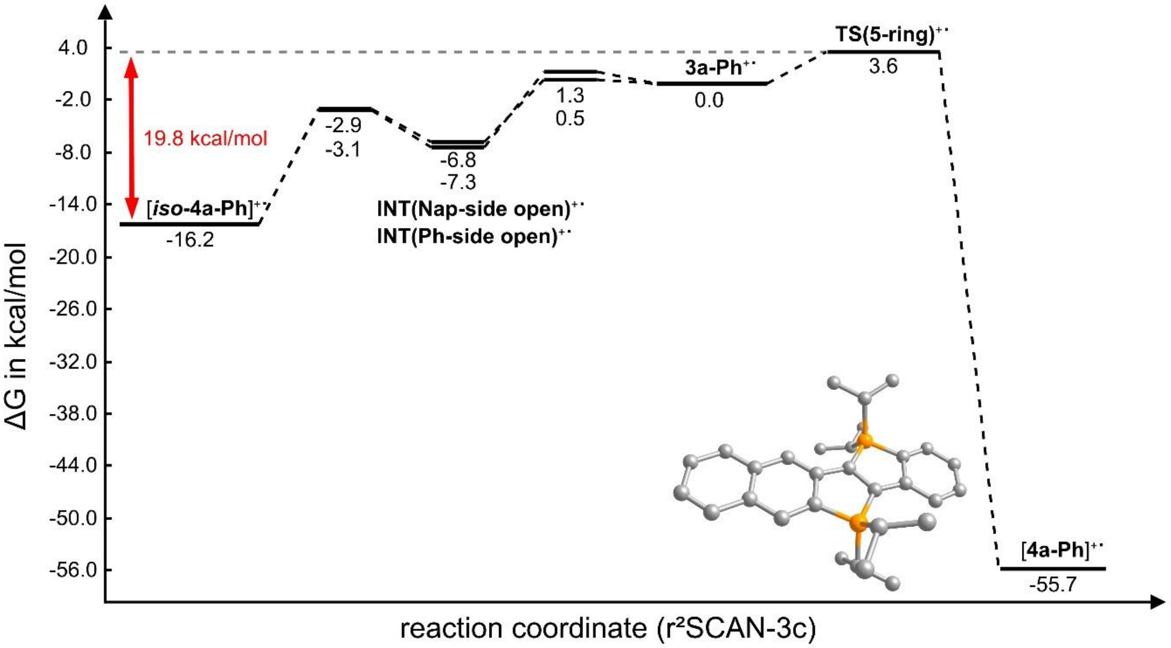

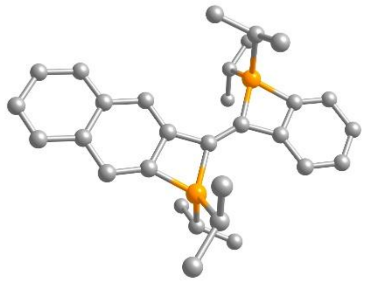

[***iso*-4a-Ph**]$^{+\bullet}$

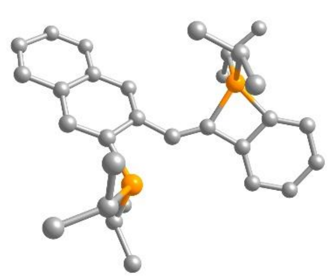

TS from [***iso*-4a**]$^{+\bullet}$ to **INT(Nap-side open)**$^{+\bullet}$

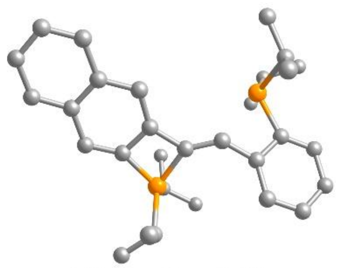

TS from [***iso*-4a**]$^{+\bullet}$ to **INT(Ph-side open)**$^{+\bullet}$

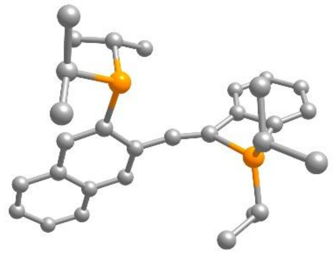

INT(Nap-side open)$^{+\bullet}$

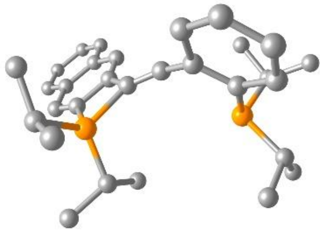

INT(Ph-side open)$^{+\bullet}$

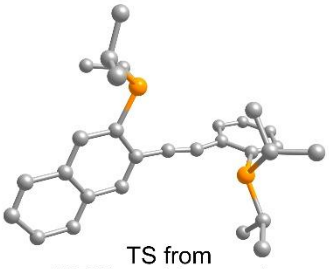

TS from **INT(Nap-side open)**$^{+\bullet}$ to **3a-Ph**$^{+\bullet}$

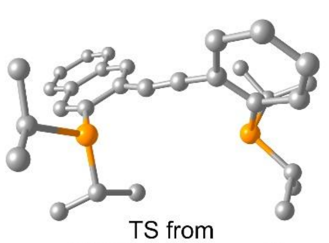

TS from **INT(Ph-side open)**$^{+}$ to **3a-Ph**$^{+\bullet}$

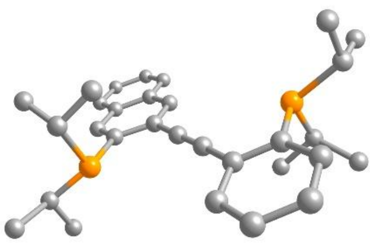

3a-Ph$^{+\bullet}$

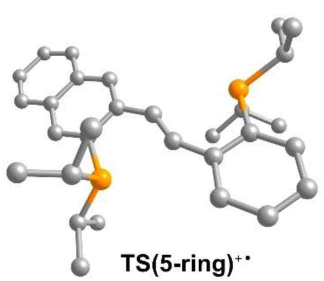

TS(5-ring)$^{+\bullet}$

Figure S110. Optimized geometries (r^{2}SCAN-3c, def2-mTZVPP, D4, CPCM for CH_2Cl_2) for all compounds that may play a role in the radical cyclization of [**3a-Ph**]$^{+\bullet}$ to [***iso*-4a-Ph**]$^{+\bullet}$ or to [**4a-Ph**]$^{+\bullet}$. Hydrogen atoms are omitted for clarity.

Optimized Geometries for the Radical Cyclization of 3b-Ph$^{+\bullet}$

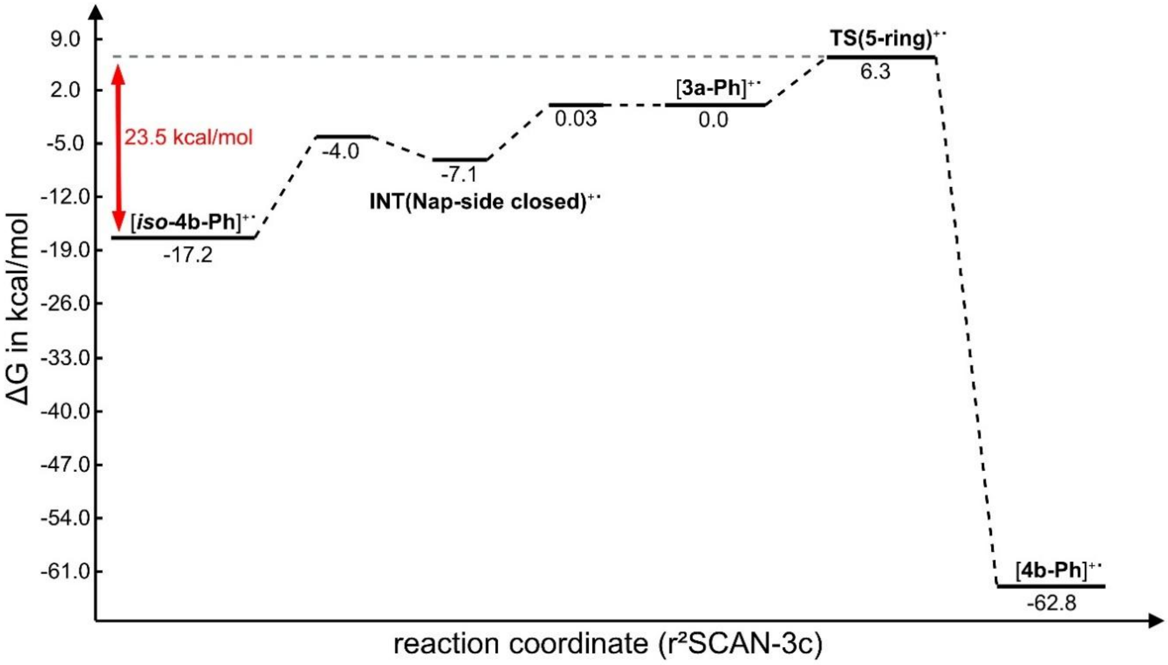

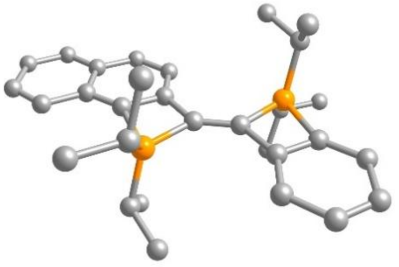

[***iso*-4b-Ph**]$^{+\bullet}$

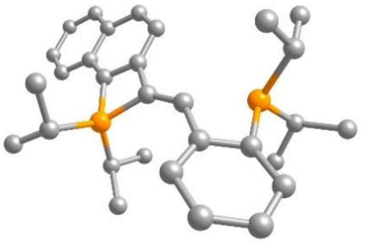

TS from [***iso*-4b-Ph**]$^{+\bullet}$ to **INT(Nap-side closed)**$^{+\bullet}$

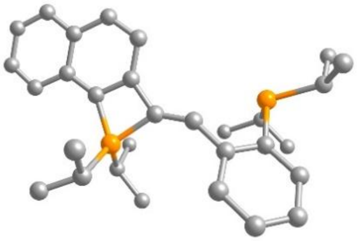

INT(Nap-side closed)$^{+\bullet}$

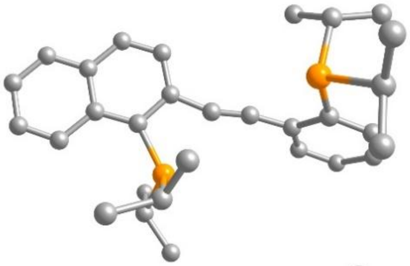

TS from **INT(Nap-side closed)**$^{+\bullet}$ to **3b-Ph**$^{+\bullet}$

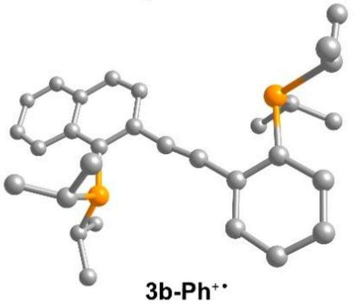

3b-Ph$^{+\bullet}$

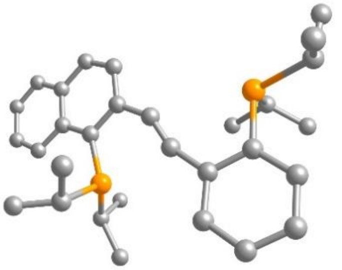

TS(5-ring)$^{+\bullet}$

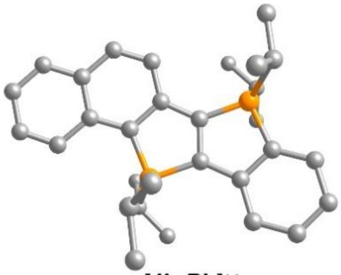

[**4b-Ph**]$^{+\bullet}$

Figure S111. Optimized geometries (r^{2}SCAN-3c, def2-mTZVPP, D4, CPCM for CH_2Cl_2) for all compounds that may play a role in the radical cyclization of [**3b-Ph**]$^{+\bullet}$ to [***iso*-4b-Ph**]$^{+\bullet}$ or to [**4b-Ph**]$^{+\bullet}$. Hydrogen atoms are omitted for clarity.

Optimized Geometries for the Radical Cyclization of 3a$^{+\bullet}$

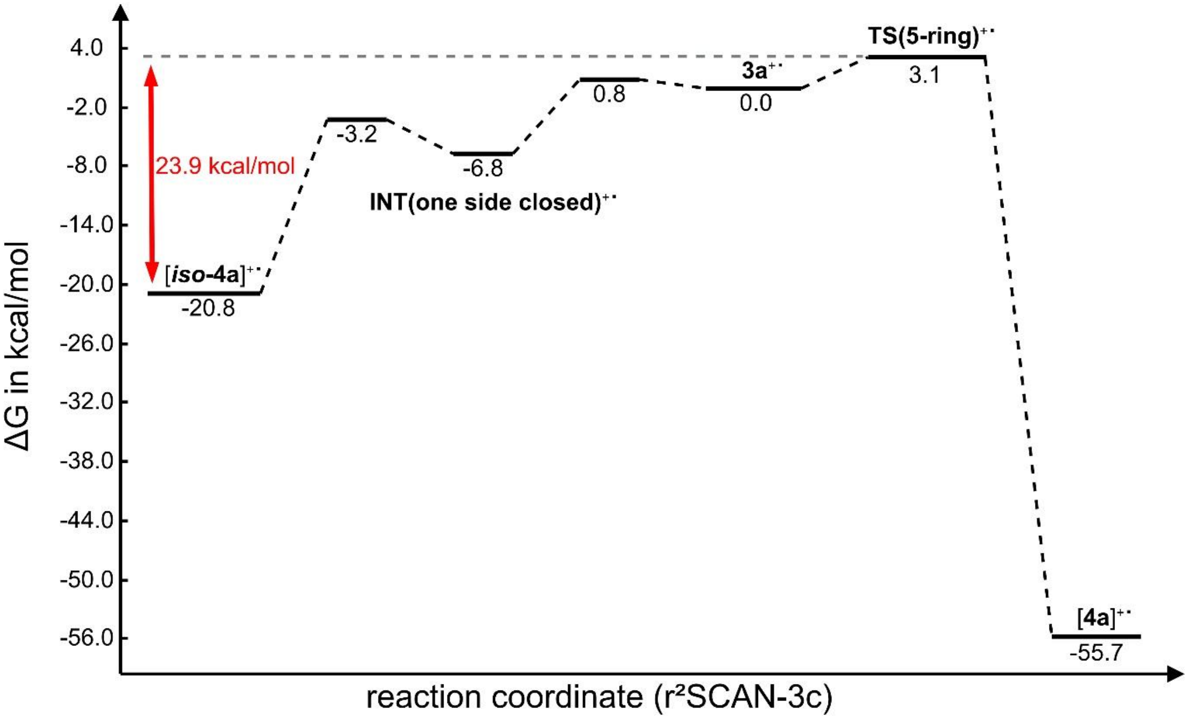

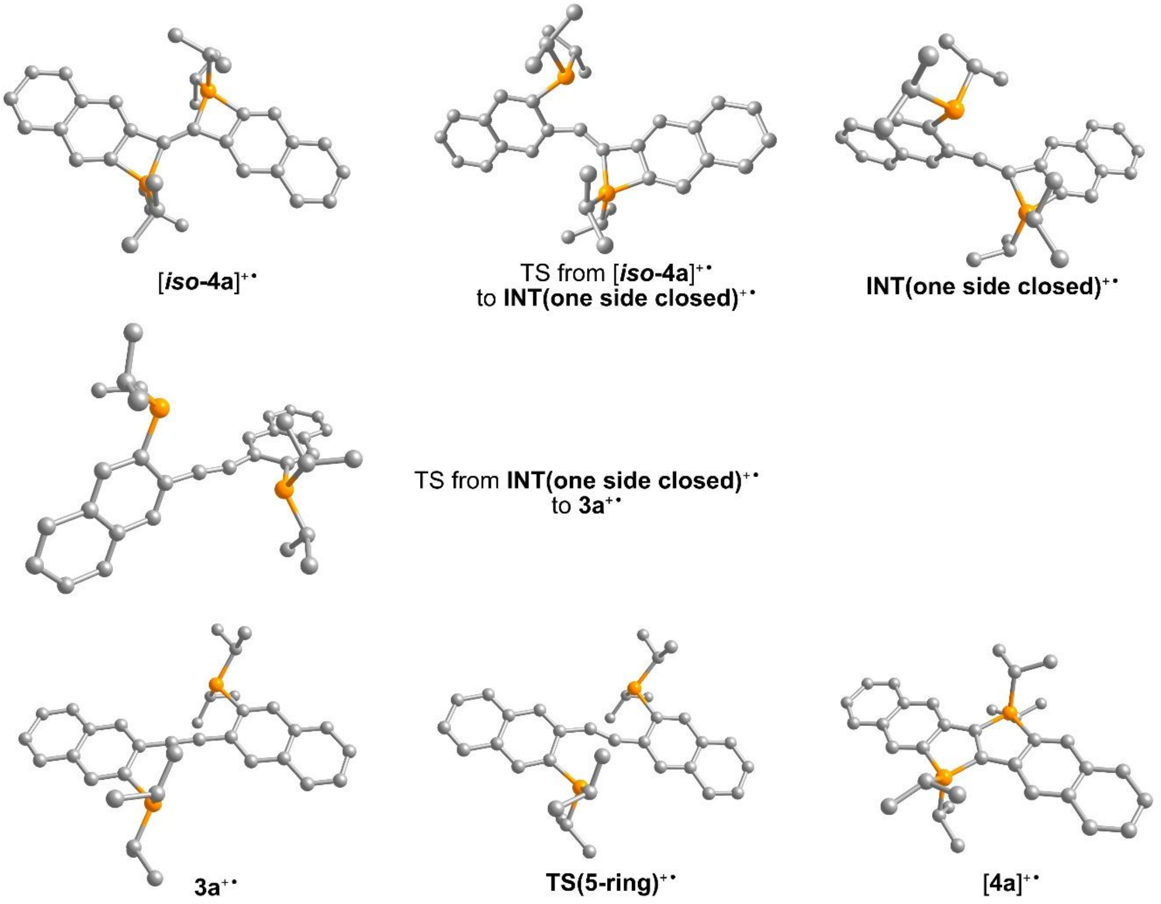

Figure S112. Optimized geometries (r^{2}SCAN-3c, def2-mTZVPP, D4, CPCM for CH_2Cl_2) for all compounds that may play a role in the radical cyclization of [**3a**]$^{+\bullet}$ to [***iso*-4a**]$^{+\bullet}$ or to [**4a**]$^{+\bullet}$. Hydrogen atoms are omitted for clarity.

Optimized Geometries for the Radical Cyclization of "3b$^{+\bullet}$" (no optimization possible for 3b$^{+\bullet}$)

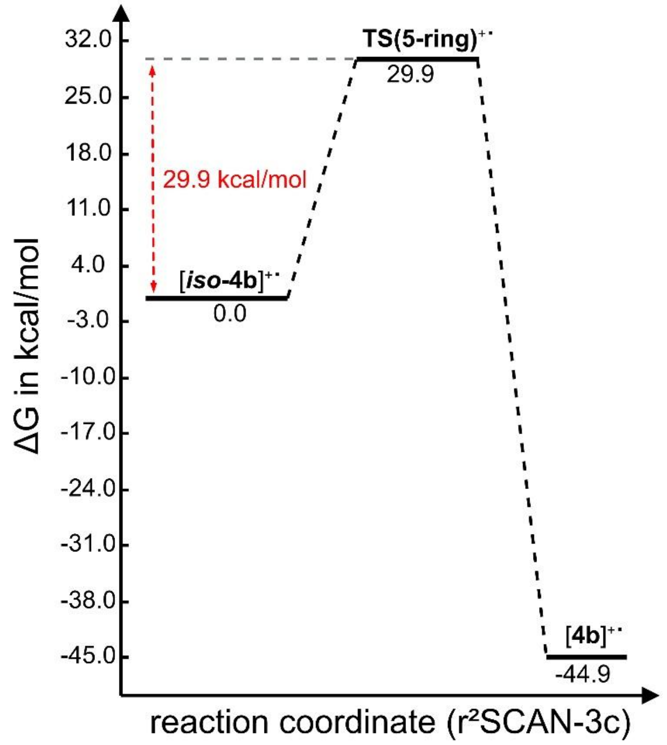

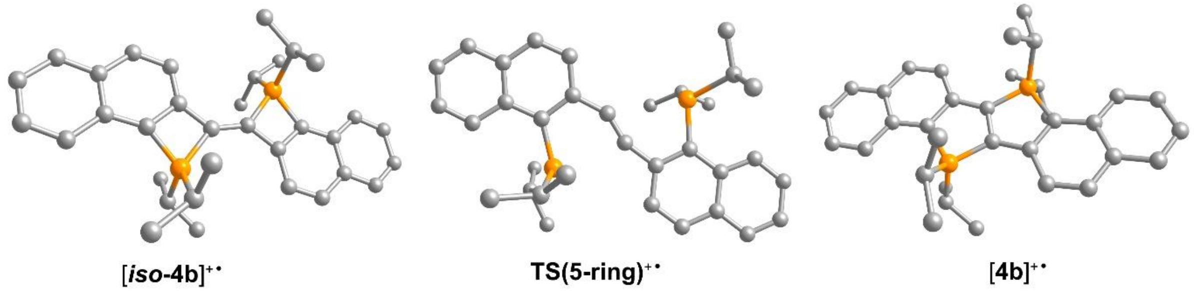

Figure S113. Optimized geometries (r^{2}SCAN-3c, def2-mTZVPP, D4, CPCM for CH_2Cl_2) for [**3b**]$^{+\bullet}$, [***iso*-4b**]$^{+\bullet}$ and for the transition state interconnecting these two compounds. Hydrogen atoms are omitted for clarity.

5.5) Choice of the DFT Method

In this article, a vast number of DFT calculations had to be carried out (approximately 50 opt/freq calculations for the ground states and intermediates, approximately 45 optts/freq calculations for the transitions states and approximately 3500 optimizations for the relaxed 2-dimensional surface scans). For that reason, only a very limited number of functionals was considered a priori and r^2SCAN-3c was eventually selected in order to keep the computational costs within reasonable limits.

Given that three quite different mechanisms were studied (mechanism A: thermal cyclization via uncharged compounds, mechanism B: twofold SET and cyclization via doubly charged compounds and mechanism C: radical cyclization via mono-cationic species, UKS), we briefly evaluated whether r^2-SCAN-3c, B3LYP D3, M06 D3zero, ωB97X-D3 and/or TPSSh D3 lead to similar or significantly different results. For each mechanism, one example was chosen (3b for mechanism A and 3a for mechanism B and C) and all relevant structure were re-optimized using the respective functional (opt/freq or opts/freq). Overall, fairly large differences were found (in some cases 10 kcal/mol), but the overall picture did *not* change, i.e. the conclusions drawn for each mechanism are *not* affected by the choice of the functional.

In the case of mechanism A (thermal cyclization of **3b**), the largest functional dependence is found for the carbene intermediate. The overall conclusion that **[*iso*-4b]^0** is not accessible, however, is not affected. For all functionals **[*iso*-4b]^0** is formed in an endergonic reaction, while **[4b]^0** is produced as the thermodynamic and the kinetic product (see Figure S114).

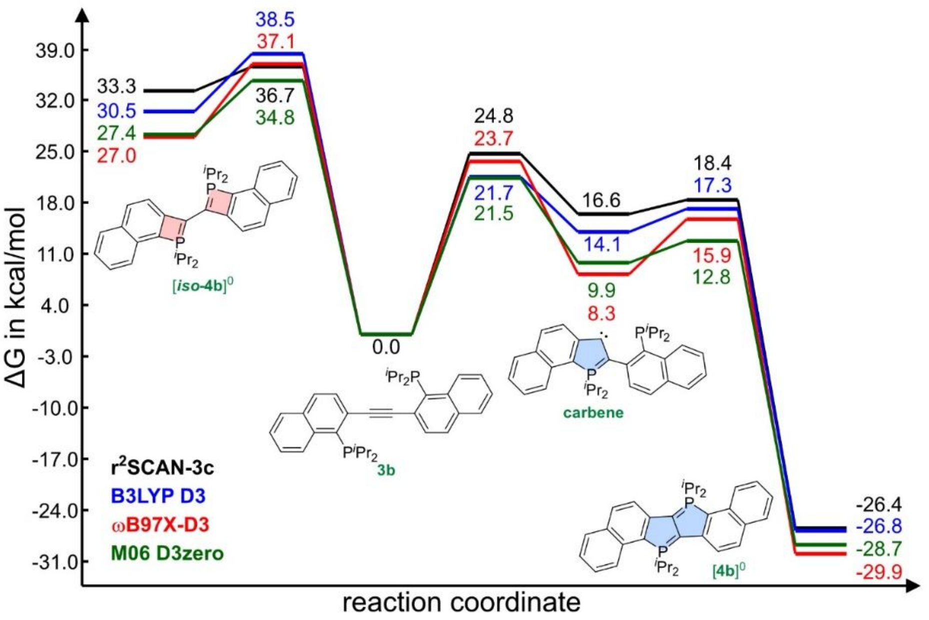

Figure S114. Thermal cyclization (mechanism A) of **3b** to either produce **[4b]^0** (experimentally observed) or **[*iso*-4b]^0** (hypothetical, not observed and excluded due to the high barriers associated with its formation) calculated using different functionals (basis set: def2-TZVPP in each case).

For mechanism B (initiated by two SET steps to afford a chlorinated phosphonium cation prior to cyclization), fairly large differences (approximately 10 kcal/mol) between the different functionals were noticed, in particular for the dications **[4a]$^{2+}$** and **[*iso*-4a]$^{2+}$**. Nevertheless, the mechanistic picture is *not* affected: The formation of **[4a]$^{2+}$** is thermodynamically (and kinetically) favored for all functionals, while **[*iso*-4a]$^{2+}$** is generated in an endergonic reaction (see Figure S115), which suggests that **[*iso*-4a]$^{2+}$** is not produced via mechanism B.

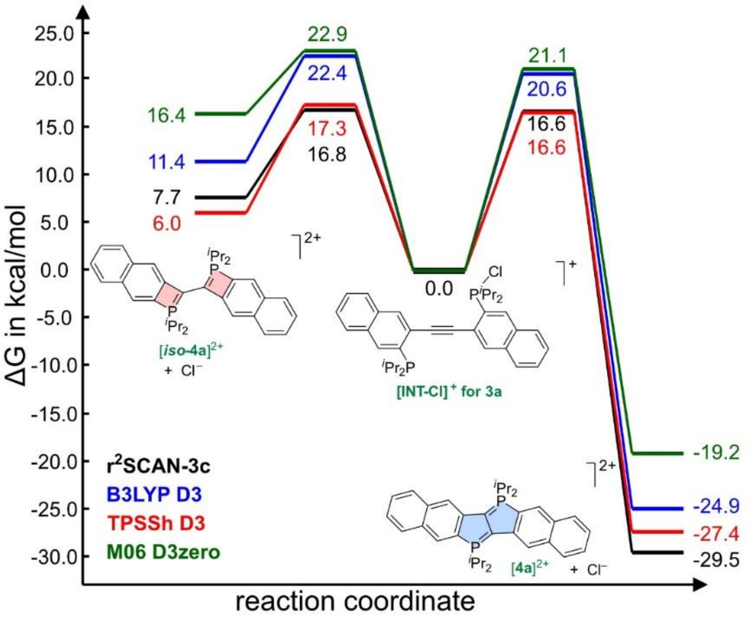

Figure S115. Cyclization (mechanism B) of **[INT-Cl]$^{+}$** for **3a** to either produce **[4a]$^{2+}$** (experimentally observed) or **[*iso*-4a]$^{2+}$** (hypothetical, not observed and excluded due to its endergonic formation) calculated using different functionals (basis set: def2-TZVPP in each case).

In the case of mechanism C (radical cyclization), the radical cation **[3a]$^{\bullet+}$** and its cyclization either afford to **[4a]$^{\bullet+}$** or **[*iso*-4a]$^{\bullet+}$** was calculated using different functionals (see Figure S116). The largest differences were observed for the products (**[4a]$^{\bullet+}$** or **[*iso*-4a]$^{\bullet+}$**), while the overall picture was *not* affected, i.e. **[4a]$^{\bullet+}$** is produced as the thermodynamic product, while **[*iso*-4a]$^{\bullet+}$** is produced as the kinetic product. All in all, it is clear that r^{2}SCAN is comparable to the other functionals, i.e. the ΔG values calculated with r^{2}SCAN-3c are neither systemically low nor systematically high.

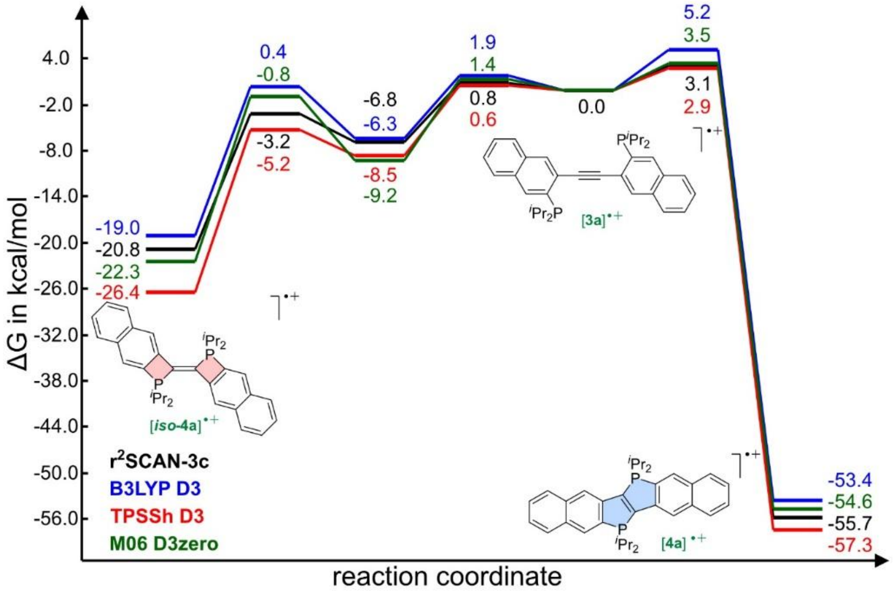

Figure S116. Radical cyclization (mechanism C) of **[3a]$^{\bullet+}$** to either produce **[4a]$^{\bullet+}$** or **[*iso*-4a]$^{\bullet+}$** calculated using different functionals (basis set: def2-TZVPP in each case).

5.6) AICD and NICS Plots

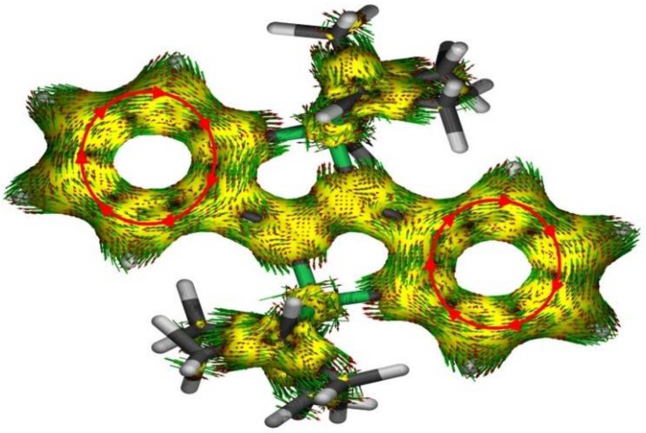

Figure S117. AICD plot of [**H**]$^{2+}$ (B3LYP, def2-TZVPP, GD3, SCRF for Water, isovalue = 0.03 = default). Diatropic ring currents (current density vectors arranged in a clockwise fashion) indicative of aromaticity are shown in red. The current density vectors at the central C=C bond are pointing to the front. The P^+ bridges are not involved in electron delocalization. Hence, the compound is interpreted as a diphosphonium bridged ladder stilbene.

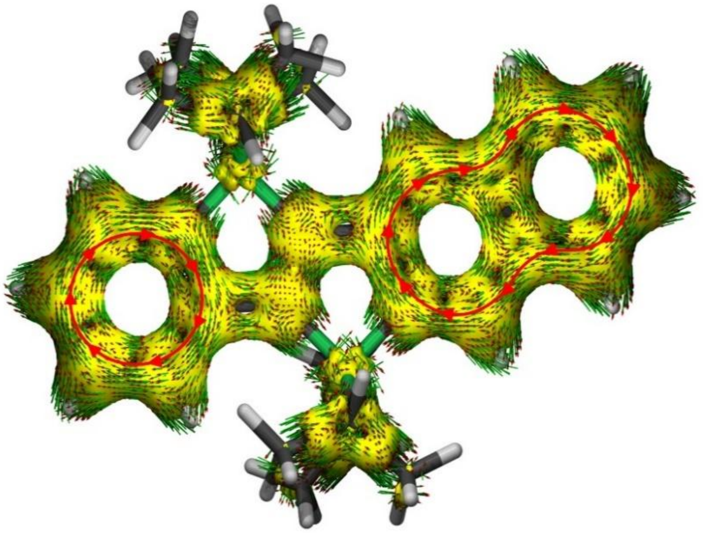

Figure S118. AICD plot of [**4a-Ph**]$^{2+}$ (B3LYP, def2-TZVPP, GD3, SCRF for Water, isovalue = 0.03 = default). Diatropic ring currents (current density vectors arranged in a clockwise fashion) indicative of aromaticity are shown in red. The current density vectors at the central C=C bond are pointing to the front. The P^+ bridges are not involved in electron delocalization. Hence, the compound is interpreted as a diphosphonium bridged ladder stilbene.

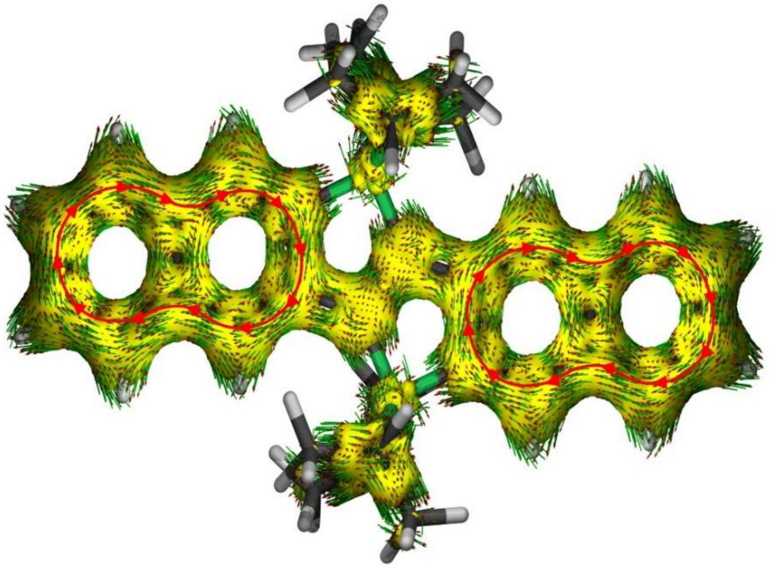

Figure S119. AICD plot of [**4a**]$^{2+}$ (B3LYP, def2-TZVPP, GD3, SCRF for Water, isovalue = 0.03 = default). Diatropic ring currents (current density vectors arranged in a clockwise fashion) indicative of aromaticity are shown in red. The current density vectors at the central C=C bond are pointing to the front. The P^+ bridges are not involved in electron delocalization. Hence, the compound is interpreted as a diphosphonium bridged ladder stilbene.

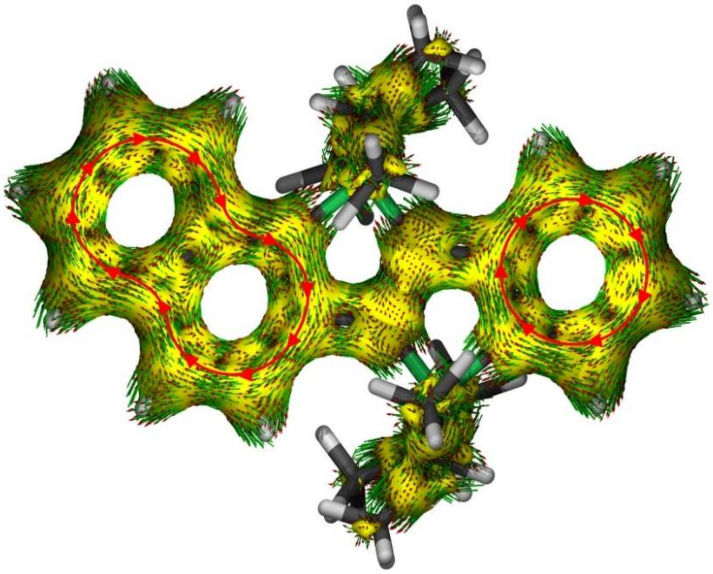

Figure S120. AICD plot of [**4b-Ph**]$^{2+}$ (B3LYP, def2-TZVPP, GD3, SCRF for Water, isovalue = 0.03 = default). Diatropic ring currents (current density vectors arranged in a clockwise fashion) indicative of aromaticity are shown in red. The current density vectors at the central C=C bond are pointing to the front. The P^+ bridges are not involved in electron delocalization. Hence, the compound is interpreted as a diphosphonium bridged ladder stilbene.

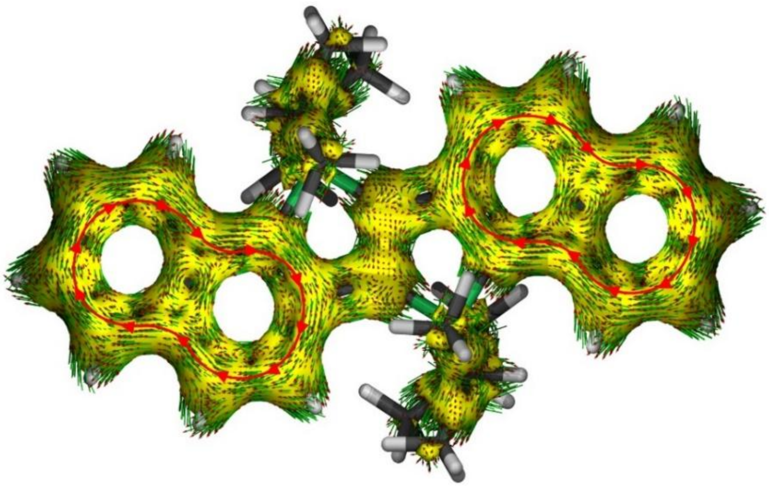

Figure S121. AICD plot of [**4b**]$^{2+}$ (B3LYP, def2-TZVPP, GD3, SCRF for Water, isovalue = 0.03 = default). Diatropic ring currents (current density vectors arranged in a clockwise fashion) indicative of aromaticity are shown in red. The current density vectors at the central C=C bond are pointing to the front. The P^{+} bridges are not involved in electron delocalization. Hence, the compound is interpreted as a diphosphonium bridged ladder stilbene.

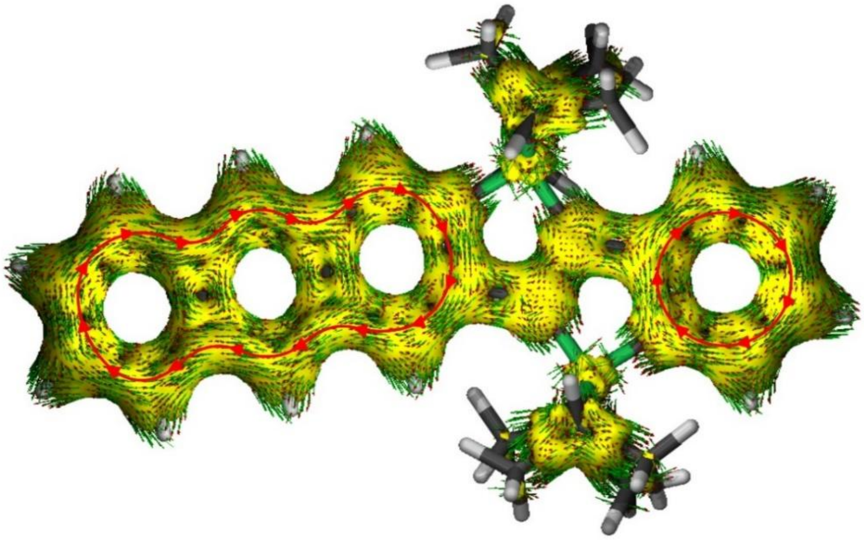

Figure S122. AICD plot of [**4c-Ph**]$^{2+}$ (B3LYP, def2-TZVPP, GD3, SCRF for Water, isovalue = 0.03 = default). Diatropic ring currents (current density vectors arranged in a clockwise fashion) indicative of aromaticity are shown in red. The current density vectors at the central C=C bond are pointing to the front. The P^{+} bridges are not involved in electron delocalization. Hence, the compound is interpreted as a diphosphonium bridged ladder stilbene.

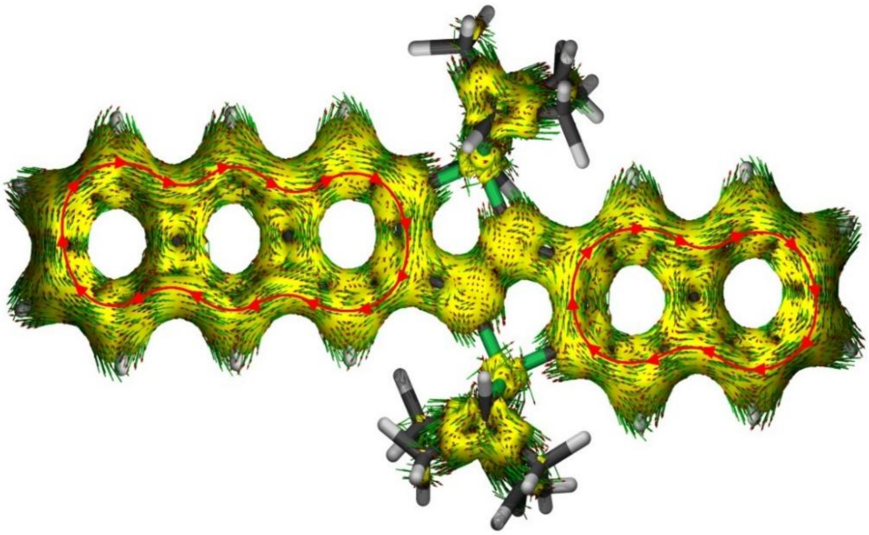

Figure S123. AICD plot of [**4c-Naph**]$^{2+}$ (B3LYP, def2-TZVPP, GD3, SCRF for Water, isovalue = 0.03 = default). Diatropic ring currents (current density vectors arranged in a clockwise fashion) indicative of aromaticity are shown in red. The current density vectors at the central C=C bond are pointing to the front. The P$^+$ bridges are not involved in electron delocalization. Hence, the compound is interpreted as a diphosphonium bridged ladder stilbene.

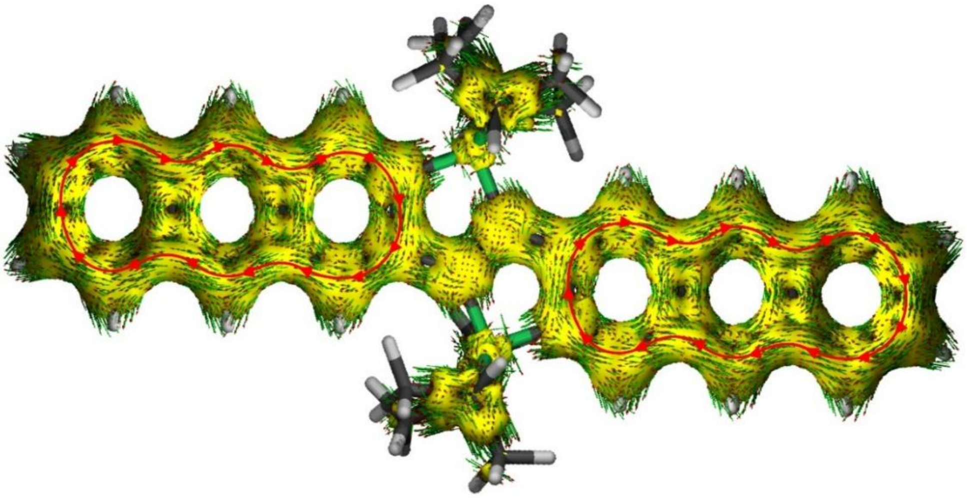

Figure S124. AICD plot of [**4c-Anth**]$^{2+}$ (B3LYP, def2-TZVPP, GD3, SCRF for Water, isovalue = 0.03 = default). Diatropic ring currents (current density vectors arranged in a clockwise fashion) indicative of aromaticity are shown in red. The current density vectors at the central C=C bond are pointing to the front. The P$^+$ bridges are not involved in electron delocalization. Hence, the compound is interpreted as a diphosphonium bridged ladder stilbene.

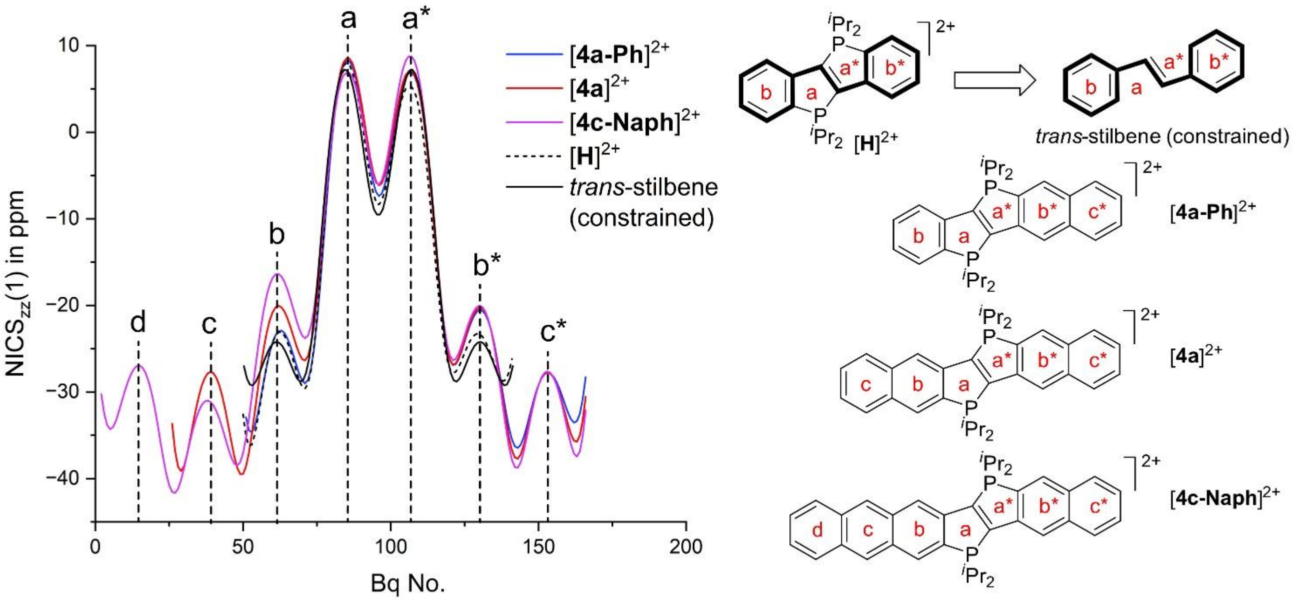

Figure S125. NICS$_{zz}$(1)-*XY* scans for [**H**]$^{2+}$, [**4a-Ph**]$^{2+}$, [**4a**]$^{2+}$ and [**4c-Naph**]$^{2+}$ (B3LYP, def2-TZVPP, GD3, SCRF for Water) together with the corresponding NICS$_{zz}$(1) scan for a geometrically constrained *trans*-stilbene ($C_{14}H_{12}$, uncharged, constrained to resemble the skeleton in [**H**]$^{2+}$ printed in bold). In compounds [**4a-Ph**]$^{2+}$, [**4a**]$^{2+}$ and [**4c-Naph**]$^{2+}$, a comparable naphtho-annulation pattern (b* and c* rings) is present at one end of the chromophores. At the other end, the π-system increases linearly in the series [**4a-Ph**]$^{2+}$ → [**4a**]$^{2+}$ → [**4c-Naph**]$^{2+}$. This NICS-*XY* scan suggests that the dicationic phospholo[3,2-b]phosphole core (a and a* rings) is electronically decoupled from the annulated arenes on each side. The positive NICS$_{zz}$(1) values for the 5-membered rings are **not** indicative of antiaromaticity as nearly identical values were obtained for the constrained *trans*-stilbene.

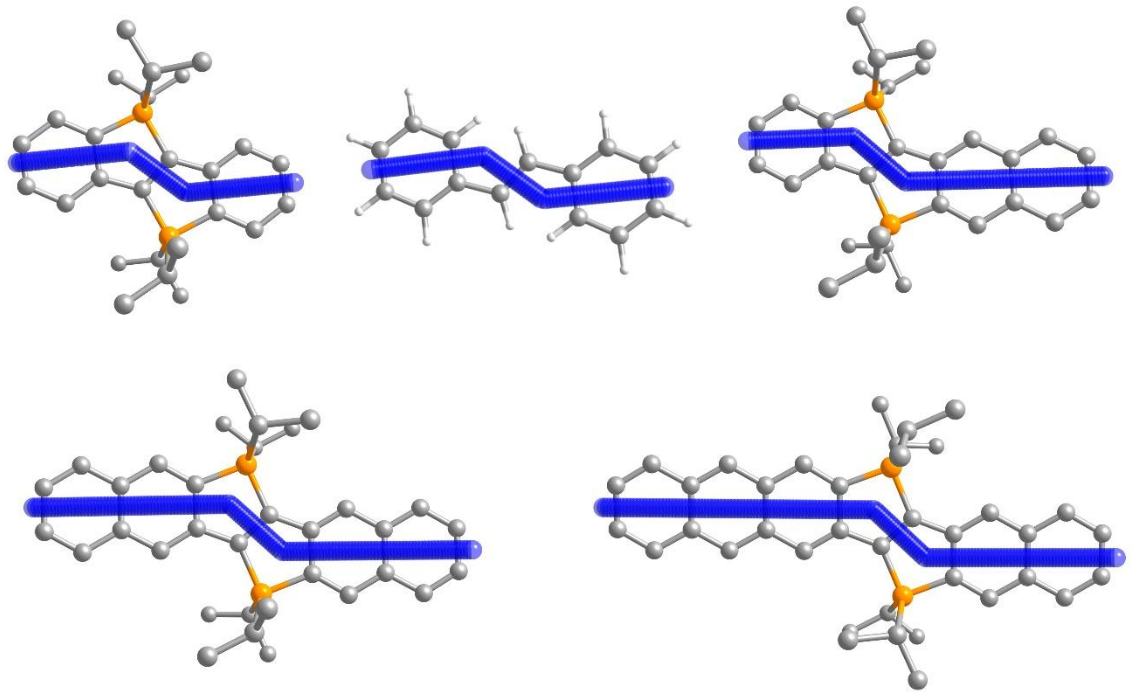

Figure S126. Trajectories for the NICS$_{zz}$(1)-*XY* scans for [**H**]$^{2+}$, [**4a-Ph**]$^{2+}$, [**4a**]$^{2+}$ and [**4c-Naph**]$^{2+}$ (B3LYP, def2-TZVPP, GD3, SCRF for water) together with the corresponding trajectory for the constrained *trans*-stilbene. Hydrogen atoms are omitted for clarity (except for the constrained *trans*-stilbene). Ghost atoms (blue) were placed 1 Å above the mean plane of each compound.

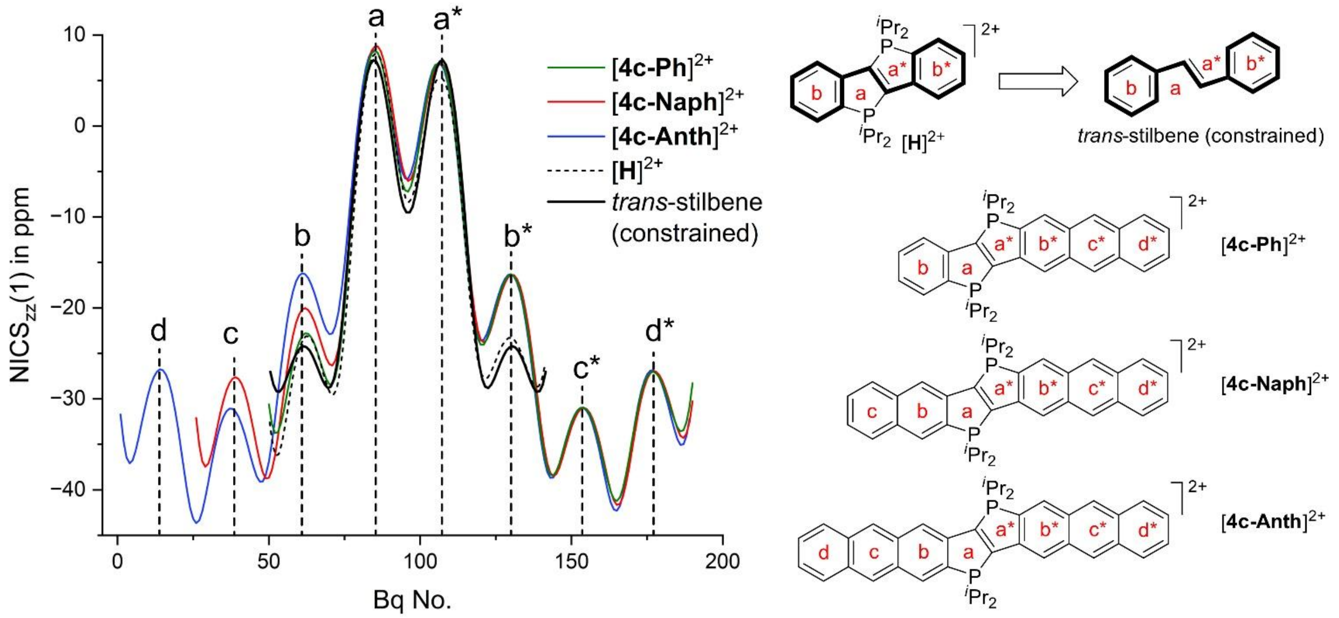

Figure S127. $NICS_{zz}$(1)-*XY* scans for [**H**]$^{2+}$, [**4c-Ph**]$^{2+}$, [**4c-Naph**]$^{2+}$ and [**4c-Anth**]$^{2+}$ (B3LYP, def2-TZVPP, GD3, SCRF for water) together with the corresponding $NICS_{zz}$(1) scan for a constrained *trans*-stilbene ($C_{14}H_{12}$, uncharged, bold bonds were constrained to resemble the skeleton in [**H**]$^{2+}$). In compounds [**4c-Ph**]$^{2+}$, [**4c-Naph**]$^{2+}$ and [**4c-Anth**]$^{2+}$, a comparable anthraceno-annulation pattern (b*, c* and d* rings) is present at one end of the chromophores. At the other end, the π-system increases linearly in the series [**4c-Ph**]$^{2+}$ → [**4c-Naph**]$^{2+}$ → [**4c-Anth**]^{2}. This NICS-*XY* scan suggests that the dicationic phospholo[3,2-b]phosphole core (a and a* rings) is electronically decoupled from the annulated arenes on each side. The positive $NICS_{zz}$(1) values for the 5-membered rings are **not** indicative of antiaromaticity as nearly identical values were obtained for the constrained *trans*-stilbene.

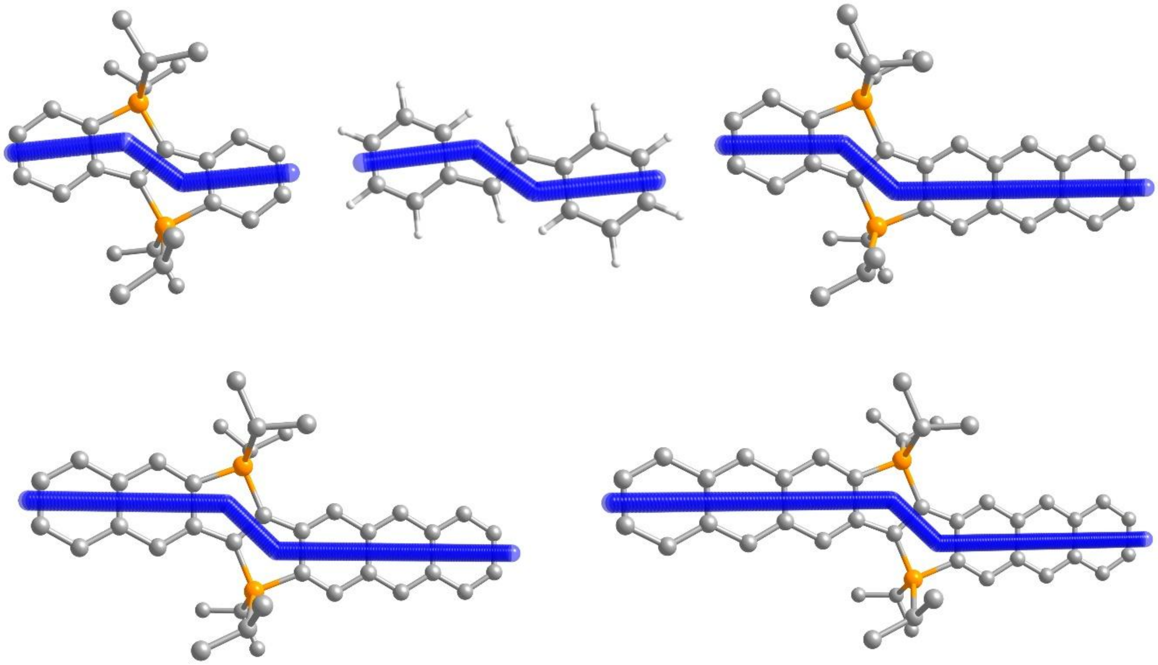

Figure S128. Trajectories for the $NICS_{zz}$(1)-*XY* scans for [**H**]$^{2+}$, [**4c-Ph**]$^{2+}$, [**4c-Naph**]$^{2+}$ and [**4c-Anth**]$^{2+}$ (B3LYP, def2-TZVPP, GD3, SCRF for water) together with the corresponding trajectory for the constrained *trans*-stilbene. Hydrogen atoms are omitted for clarity (except for the constrained *trans*-stilbene). Ghost atoms (blue) were placed 1 Å above the mean plane of each compound.

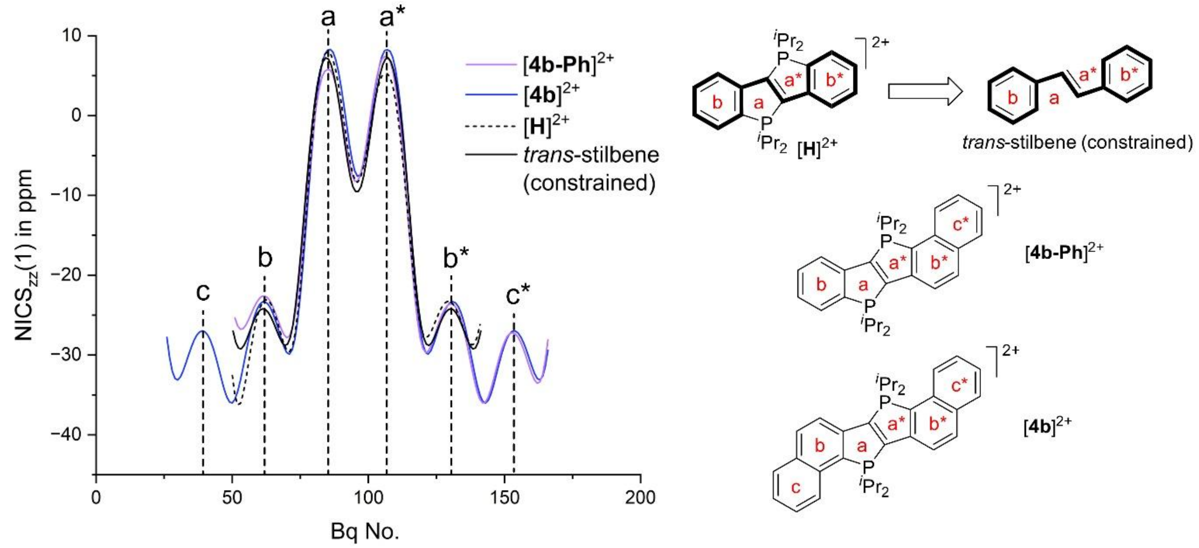

Figure S129. NICS$_{zz}$(1)-*XY* scans for [**H**]$^{2+}$, [**4b-Ph**]$^{2+}$ and [**4b**]$^{2+}$ (B3LYP, def2-TZVPP, GD3, SCRF for water) together with the corresponding NICS$_{zz}$(1) scan for a constrained *trans*-stilbene ($C_{14}H_{12}$, uncharged, bold bonds were constrained to resemble the skeleton in [**H**]$^{2+}$). This NICS-*XY* scan suggests that the dicationic phospholo[3,2-b]phosphole core (a and a* rings) is electronically decoupled from the annulated arenes on each side. The aromaticity in the b ring remains nearly identical within the series. The positive NICS$_{zz}$(1) values for the 5-membered rings are **not** indicative of antiaromaticity as nearly identical values were obtained for the constrained *trans*-stilbene.

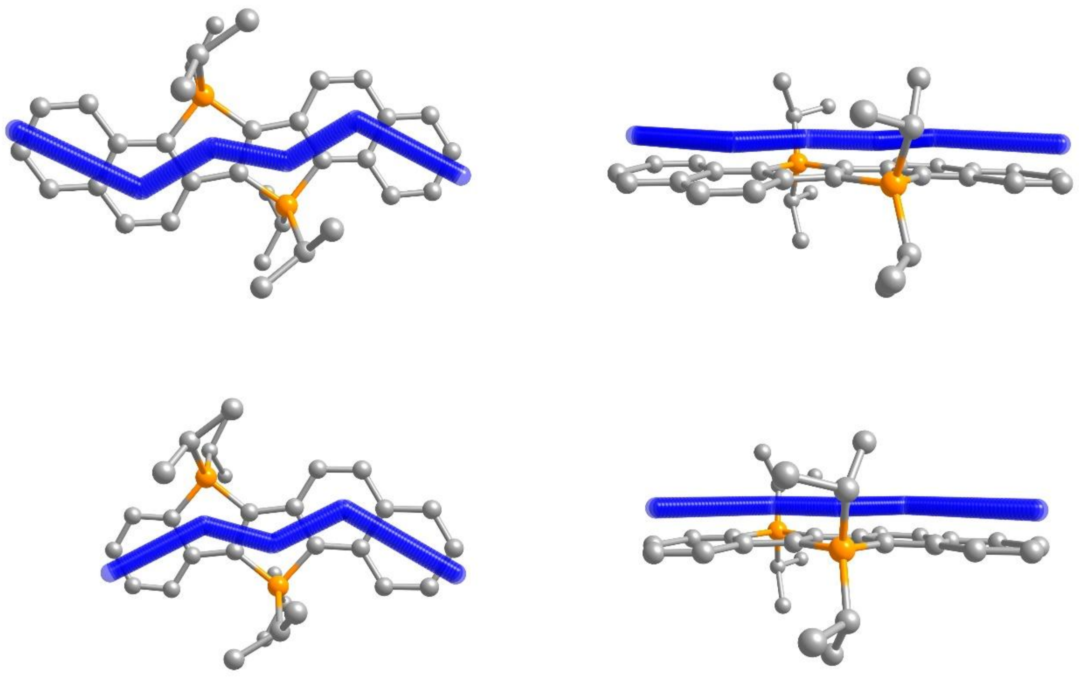

Figure S130. Trajectories for the NICS$_{zz}$(1)-*XY* scans for [**4b**]$^{2+}$ and [**4b-Ph**]$^{2+}$ (B3LYP, def2-TZVPP, GD3, SCRF for water). Hydrogen atoms are omitted for clarity. Ghost atoms (blue) were placed 1 Å above the mean plane of each compound.

5.6) TD-DFT Results and Calculated UV-Vis Spectra

Table S27. TD-DFT results for [**H**]$^{2+}$ (B3LYP, def2-TZVPP, GD3, SCRF for water, TD(NStates=40)).

excitation	λ (nm)	E (eV)	f	orbital contribution for transitions with f > 0.1
**1**	419.98	2.9521	0.2167	**110 = HOMO** -> **111 = LUMO** (0.70197)
**2**	319.3	3.883	0.0679	f < 0.1
**3**	317.77	3.9017	0.0012	f < 0.1
**4**	287.26	4.3161	0.0	f < 0.1
**5**	275.41	4.5018	0.0001	f < 0.1
**6**	257.74	4.8104	0.3675	110->113 (0.66095); 109->111 (-0.15677); 108->112 (-0.12479)
**7**	243.25	5.0969	0.1088	106->111 (0.69814)
**8**	235.34	5.2684	0.0054	f < 0.1
**9**	232.14	5.341	0.0004	f < 0.1
**10**	229.47	5.4031	0.0023	f < 0.1
**11**	228.02	5.4374	0.0006	f < 0.1
**12**	227.43	5.4516	0.0208	f < 0.1
**13**	225.38	5.5011	0.0002	f < 0.1
**14**	217.34	5.7047	0.0891	f < 0.1
**15**	216.07	5.7381	0.0028	f < 0.1
**16**	214.32	5.785	0.0001	f < 0.1
**17**	211.8	5.8539	0.3797	108->112 (0.46213); 109->113 (-0.36122); 110->116 (-0.26207); 101->111 (-0.21524)
**18**	208.25	5.9537	0.1689	107->112 (0.43213); 110->116 (0.3151); 110->117 (0.30545); 108->112 (0.22359); 101->111 (0.19515)
**19**	206.93	5.9916	0.0262	f < 0.1
**20**	205.93	6.0208	0.0018	f < 0.1
**21**	205.12	6.0445	0.0086	f < 0.1
**22**	204.01	6.0774	0.0021	f < 0.1
**23**	202.29	6.1291	0.0170	f < 0.1
**24**	201.91	6.1405	0.0308	f < 0.1
**25**	201.39	6.1564	0.0213	f < 0.1
**26**	200.83	6.1736	0.1545	96->111 (0.3958); 95->111 (0.26944); 109->113 (-0.25723); 98->111 (0.23106); 108->114 (-0.16398); 110->116 (0.16202); 101->111 (0.13312); 100->111 (0.12915); 94->111 (-0.1207); 99->111 (-0.10965); 107->112 (-0.10093)
**27**	200.31	6.1897	0.0600	f < 0.1
**28**	199.37	6.2188	0.0166	f < 0.1
**29**	198.8	6.2366	0.0712	f < 0.1
**30**	195.74	6.3341	0.0185	f < 0.1
**31**	195.11	6.3547	0.0101	f < 0.1
**32**	194.45	6.376	0.0059	f < 0.1
**33**	192.84	6.4293	0.0062	f < 0.1
**34**	192.5	6.4406	0.0233	f < 0.1
**35**	192.04	6.4562	0.0001	f < 0.1
**36**	191.06	6.4892	0.0011	f < 0.1
**37**	186.54	6.6466	0.0004	f < 0.1
**38**	184.59	6.7169	0.0010	f < 0.1
**39**	182.36	6.799	0.0006	f < 0.1
**40**	181.87	6.8173	0.0101	f < 0.1

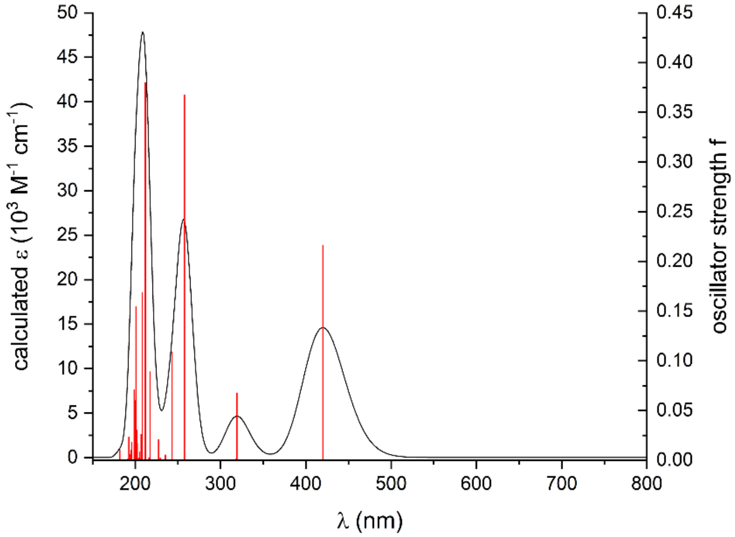

Figure S131. Calculated UV/Vis spectrum (B3LYP, def2-TZVPP, GD3, SCRF for water, TD(NStates=40)) for [**H**]$^{2+}$. The line broadening was simulated with a half width of 0.2 eV (1613 cm^{-1}) at the half height of each peak.

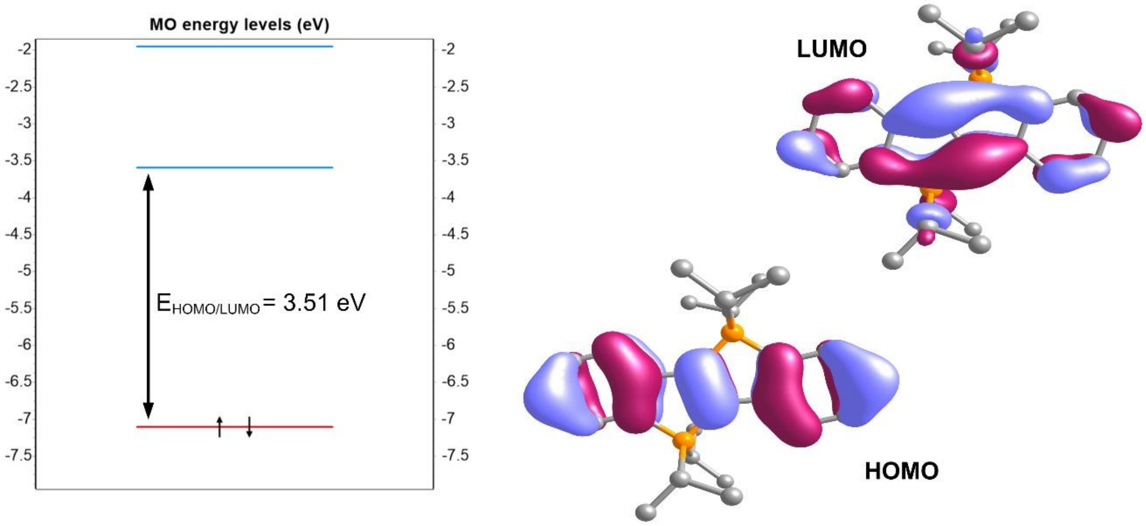

Figure S132. Calculated (B3LYP, def2-TZVPP, GD3, SCRF for water) HOMO LUMO gap for [**H**]$^{2+}$ in the **ground state** and Kohn-Sham frontier orbitals for [**H**]$^{2+}$. Molecular orbitals were plotted with an isovalue of 0.04. Note that the HOMO LUMO gap in the ground state is **not** a proper estimate for the energy separation between the ground state and the first excited state ($\Delta E_{TD\text{-}DFT}$ $S_0 \rightarrow S_1$ = 2.9521 eV).

Table S28. TD-DFT results for [**4a-Ph**]$^{2+}$ (B3LYP, def2-TZVPP, GD3, SCRF for water, TD(NStates=40)).

excitation	λ (nm)	E (eV)	f	orbital contribution for transitions with f > 0.1
**1**	449.42	2.7588	0.3839	**123 = HOMO** -> **124 = LUMO** (0.69697)
**2**	389.51	3.1831	0.0223	f < 0.1
**3**	322.95	3.8391	0.0282	f < 0.1
**4**	313.95	3.9492	0.0228	f < 0.1
**5**	305.14	4.0631	0.0423	f < 0.1
**6**	289.12	4.2884	0.0244	f < 0.1
**7**	271.95	4.5591	0.8578	122->125 (0.51449); 123->126 (-0.38468); 120->124 (0.14485); 123->127 (0.14344)
**8**	263.55	4.7043	0.0426	f < 0.1
**9**	253.32	4.8943	0.0136	f < 0.1
**10**	245.75	5.0451	0.0130	f < 0.1
**11**	241.29	5.1384	0.0031	f < 0.1
**12**	240.85	5.1478	0.0618	f < 0.1
**13**	234.18	5.2944	0.0931	f < 0.1
**14**	231.28	5.3607	0.0007	f < 0.1
**15**	230.68	5.3747	0.0029	f < 0.1
**16**	229.43	5.4041	0.0061	f < 0.1
**17**	227.29	5.4549	0.1991	116->124 (0.42785); 117->124 (-0.32377); 113->124 (0.20345); 121->125 (-0.20299); 123->127 (0.1993); 120->126 (-0.13361); 123->129 (0.13131); 118->124 (0.10275)
**18**	225.75	5.492	0.0300	f < 0.1
**19**	225.13	5.5071	0.0521	f < 0.1
**20**	224.04	5.5341	0.3994	122->127 (0.36702); 123->129 (0.27298); 116->124 (-0.2505); 121->125 (-0.24876); 123->130 (0.24839); 121->126 (0.13475); 123->127 (0.13215); 113->124 (0.11003); 120->125 (0.10637); 119->125 (-0.10519)
**21**	219.25	5.6548	0.0581	f < 0.1
**22**	218.26	5.6806	0.0108	f < 0.1
**23**	216.41	5.7291	0.0170	f < 0.1
**24**	216.04	5.7391	0.1299	121->126 (0.36408); 122->128 (-0.3441); 123->129 (-0.22277); 122->127 (0.20309); 120->127 (0.18865); 116->124 (0.16504); 123->130 (-0.12817); 120->126 (0.1139)
**25**	214.44	5.7818	0.0419	f < 0.1
**26**	212.19	5.8431	0.0057	f < 0.1
**27**	209.09	5.9298	0.0306	f < 0.1
**28**	207.8	5.9665	0.0273	f < 0.1
**29**	207.1	5.9866	0.2798	120->126 (0.43958); 123->130 (0.26387); 119->125 (0.23992); 122->129 (-0.21501); 123->132 (-0.187); 122->127 (-0.15161); 121->127 (-0.12343); 122->130 (-0.12174)
**30**	204.28	6.0694	0.0158	f < 0.1
**31**	202.78	6.1143	0.0014	f < 0.1
**32**	201.11	6.1649	0.0265	f < 0.1
**33**	200.81	6.1741	0.0146	f < 0.1
**34**	200.22	6.1924	0.0131	f < 0.1
**35**	199.57	6.2125	0.0056	f < 0.1
**36**	199	6.2304	0.0091	f < 0.1
**37**	198.4	6.2491	0.0069	f < 0.1
**38**	198.25	6.2538	0.0121	f < 0.1
**39**	196.62	6.3058	0.0189	f < 0.1
**40**	196.12	6.3217	0.0010	f < 0.1

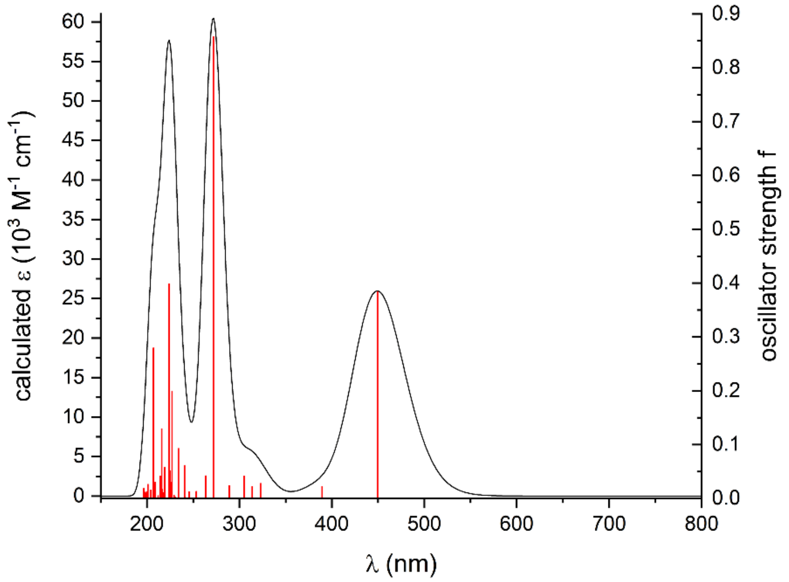

Figure S133. Calculated UV/Vis spectrum (B3LYP, def2-TZVPP, GD3, SCRF for water, TD(NStates=40)) for [**4a-Ph**]$^{2+}$. The line broadening was simulated with a half width of 0.2 eV (1613 cm^{-1}) at the half height of each peak.

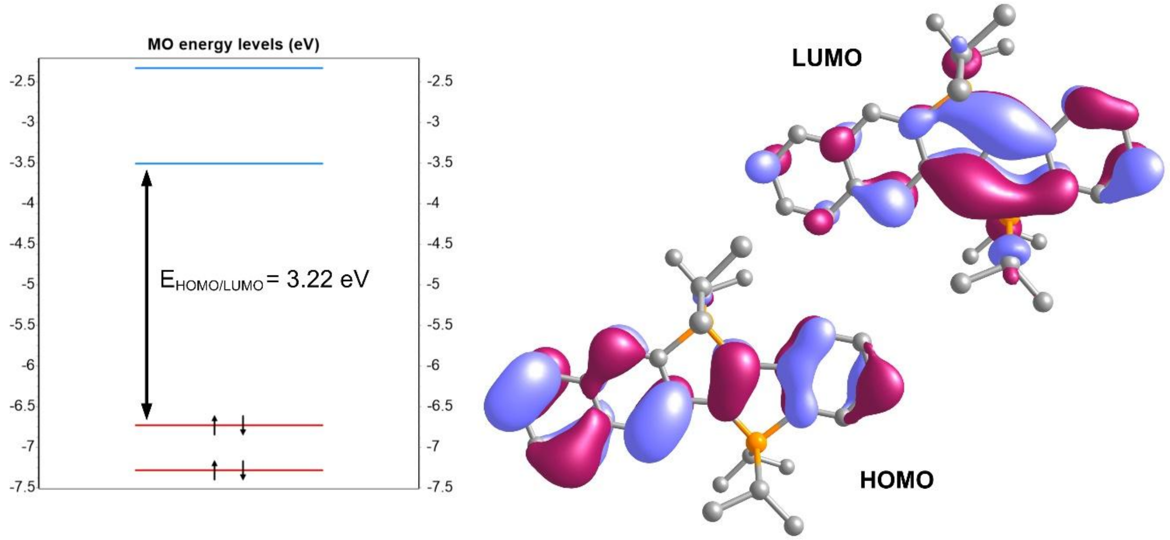

Figure S134. Calculated (B3LYP, def2-TZVPP, GD3, SCRF for water) HOMO LUMO gap for [**4a-Ph**]$^{2+}$ in the **ground state** and Kohn-Sham frontier orbitals for [**4a-Ph**]$^{2+}$. Molecular orbitals were plotted with an isovalue of 0.04. Note that the HOMO LUMO gap in the ground state is **not** a proper estimate for the energy separation between the ground state and the first excited state ($\Delta E_{TD\text{-}DFT}$ $S_0 \rightarrow S_1$ = 2.7588 eV).

Table S29. TD-DFT results for [**4a**]$^{2+}$ (B3LYP, def2-TZVPP, GD3, SCRF for Water, TD(NStates=40).

excitation	λ (nm)	E (eV)	f	orbital contribution for transitions with f > 0.1
**1**	458.54	2.7039	0.6105	**136 = HOMO** -> **137 = LUMO** (0.69513)
**2**	399.12	3.1065	0.0001	f < 0.1
**3**	377.3	3.2861	0.0206	f < 0.1
**4**	335.44	3.6962	0.2207	136->138 (0.66772); 134->137 (0.14767); 136->137 (-0.10157)
**5**	327.06	3.7909	0.0007	f < 0.1
**6**	317.22	3.9085	0.0	f < 0.1
**7**	295.92	4.1897	0.0002	f < 0.1
**8**	283.87	4.3676	0.0071	f < 0.1
**9**	281.91	4.398	1.0395	134->138 (0.50044); 135->139 (0.44625); 132->137 (-0.10962); 135->140 (0.10403)
**10**	272.22	4.5545	0.0002	f < 0.1
**11**	267.22	4.6398	0.0660	f < 0.1
**12**	258.87	4.7894	0.0	f < 0.1
**13**	255.65	4.8498	0.0001	f < 0.1
**14**	249.56	4.9681	0.0706	f < 0.1
**15**	247.34	5.0127	0.0	f < 0.1
**16**	240.86	5.1475	0.0012	f < 0.1
**17**	238.57	5.197	0.0179	f < 0.1
**18**	233.43	5.3114	0.1028	129->137 (0.65333); 136->142 (0.21387)
**19**	230.42	5.3808	1.2372	133->139 (0.46514); 135->140 (-0.40681); 136->142 (0.18836); 136->143 (-0.18622)
**20**	229.44	5.4038	0.0334	f < 0.1
**21**	228.68	5.4217	0.1302	136->142 (0.40597); 130->137 (0.31018); 135->140 (0.24372); 132->138 (0.20339); 134->140 (0.18521); 129->137 (-0.17978); 133->140 (-0.10107)
**22**	227.58	5.4479	0.0017	f < 0.1
**23**	226.48	5.4744	0.0033	f < 0.1
**24**	225.23	5.5048	0.0090	f < 0.1
**25**	225.03	5.5098	0.0469	f < 0.1
**26**	223.16	5.5559	0.0018	f < 0.1
**27**	222.37	5.5756	0.0015	f < 0.1
**28**	221.03	5.6094	0.0003	f < 0.1
**29**	219.31	5.6533	0.0049	f < 0.1
**30**	216.21	5.7345	0.2455	132->138 (0.48564); 136->143 (-0.42055); 136->142 (-0.1276); 133->140 (0.10875)
**31**	215.15	5.7626	0.0023	f < 0.1
**32**	213.39	5.8101	0.0012	f < 0.1
**33**	211.74	5.8555	0.0010	f < 0.1
**34**	210.07	5.9021	0.0032	f < 0.1
**35**	209.57	5.9161	0.0009	f < 0.1
**36**	208.79	5.9383	0.0001	f < 0.1
**37**	207.2	5.9838	0.0205	f < 0.1
**38**	207.12	5.9861	0.0045	f < 0.1
**39**	206.72	5.9977	0.0075	f < 0.1
**40**	205.91	6.0214	0.0	f < 0.1

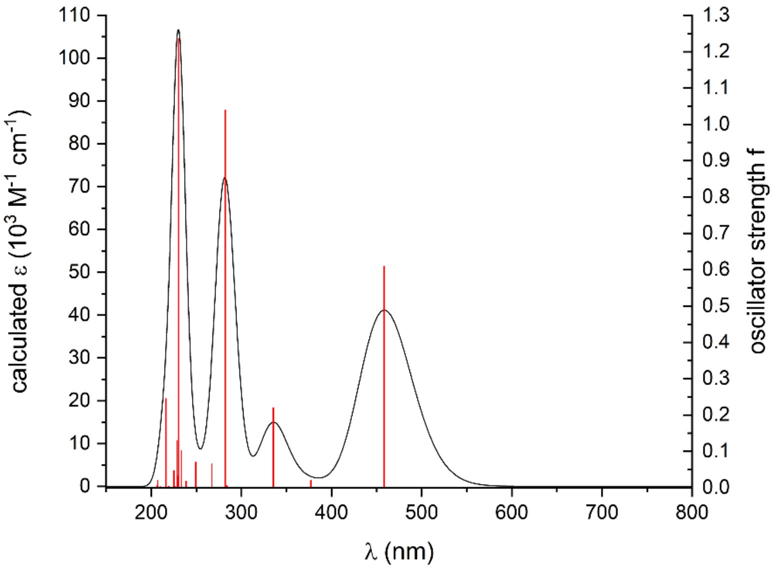

Figure S135. Calculated UV/Vis spectrum (B3LYP, def2-TZVPP, GD3, SCRF for water, TD(NStates=40)) for [**4a**]$^{2+}$. The line broadening was simulated with a half width of 0.2 eV (1613 cm^{-1}) at the half height of each peak.

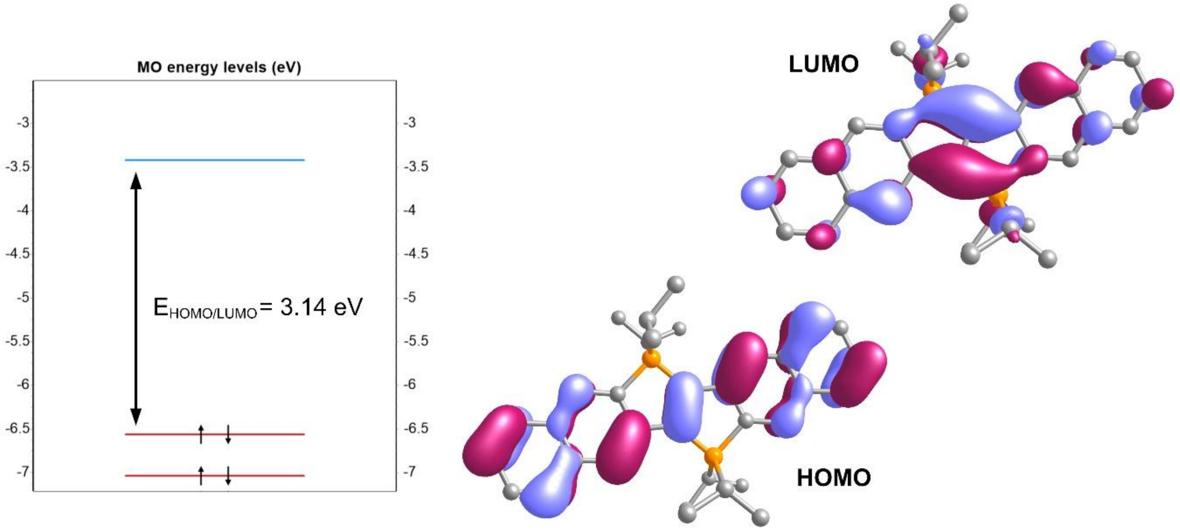

Figure S136. Calculated (B3LYP, def2-TZVPP, GD3, SCRF for water) HOMO LUMO gap for [**4a**]$^{2+}$ in the **ground state** and Kohn-Sham frontier orbitals for [**4a**]$^{2+}$. Molecular orbitals were plotted with an isovalue of 0.04. Note that the HOMO LUMO gap in the ground state is **not** a proper estimate for the energy separation between the ground state and the first excited state ($\Delta E_{TD\text{-}DFT}$ $S_0 \rightarrow S_1$ = 2.7039 eV).

Table S30. TD-DFT results for [**4b-Ph**]$^{2+}$ (B3LYP, def2-TZVPP, GD3, SCRF for water, TD(NStates=40)).

excitation	λ (nm)	E (eV)	f	orbital contribution for transitions with f > 0.1
**1**	494.98	2.5048	0.1194	**123 = HOMO** -> **124 = LUMO** (0.70443)
**2**	412.15	3.0082	0.2005	122->124 (0.69814)
**3**	328.56	3.7736	0.031	f < 0.1
**4**	322.55	3.8439	0.0212	f < 0.1
**5**	310.47	3.9935	0.0525	f < 0.1
**6**	282.74	4.3851	0.0406	f < 0.1
**7**	276.33	4.4868	0.0164	f < 0.1
**8**	269.99	4.5921	0.4805	122->125 (0.43364); 123->126 (-0.42244); 119->124 (0.26347); 123->127 (-0.11906)
**9**	248.5	4.9894	0.0381	f < 0.1
**10**	245.32	5.054	0.0732	f < 0.1
**11**	243.24	5.0971	0.0359	f < 0.1
**12**	242.54	5.1118	0.0001	f < 0.1
**13**	241.19	5.1406	0.0029	f < 0.1
**14**	239.26	5.182	0.1210	123->128 (0.56306); 122->126 (0.22676); 123->129 (-0.1867); 118->124 (0.16262); 121->125 (-0.15676); 115->124 (-0.1115)
**15**	236.15	5.2503	0.0073	f < 0.1
**16**	235.05	5.2748	0.0103	f < 0.1
**17**	231.9	5.3464	0.051	f < 0.1
**18**	230.98	5.3678	0.2325	120->125 (0.43708); 113->124 (0.31155); 121->125 (0.2822); 123->127 (0.16339); 114->124 (0.1574); 116->124 (-0.11233); 122->127 (0.10772); 117->124 (-0.10688)
**19**	229.5	5.4024	0.0265	f < 0.1
**20**	221.58	5.5954	0.2374	120->125 (0.35055); 123->127 (-0.32364); 121->125 (-0.26255); 122->126 (-0.20938); 122->128 (0.1928); 122->127 (-0.18536); 121->126 (-0.15251)
**21**	219.69	5.6436	0.1163	122->127 (0.39964); 122->128 (0.3747); 121->126 (0.25456); 122->129 (0.21151); 121->125 (-0.17021)
**22**	218.85	5.6652	0.0064	f < 0.1
**23**	218.03	5.6864	0.0150	f < 0.1
**24**	215.55	5.752	0.1134	123->130 (0.54173); 119->125 (-0.29894); 122->129 (0.1479); 122->128 (-0.14716); 120->126 (-0.10341)
**25**	212.83	5.8255	0.2112	122->128 (0.42673); 121->126 (-0.23321); 123->130 (0.22557); 122->127 (-0.22055); 120->126 (0.15639); 123->127 (0.15233); 123->131 (0.14183); 121->125 (0.11225); 120->125 (-0.10537); 120->128 (-0.10158)
**26**	212.59	5.832	0.0067	f < 0.1
**27**	211.14	5.8721	0.0076	f < 0.1
**28**	210.95	5.8775	0.0109	f < 0.1
**29**	210.13	5.9002	0.0199	f < 0.1
**30**	209.37	5.9217	0.0737	f < 0.1
**31**	209.25	5.925	0.0173	f < 0.1
**32**	208.81	5.9377	0.0039	f < 0.1
**33**	207.11	5.9865	0.1246	120->126 (0.50094); 121->126 (0.29887); 119->125 (-0.27503); 122->130 (-0.15751); 121->128 (-0.11103)
**34**	205.16	6.0433	0.0070	f < 0.1
**35**	204.9	6.0511	0.0065	f < 0.1
**36**	204.72	6.0561	0.0017	f < 0.1
**37**	200.99	6.1688	0.1141	119->125 (0.32502); 120->126 (0.28209); 103->124 (0.25124); 104->124 (-0.20641); 123->132 (0.16483); 122->130 (0.16391); 122->128 (-0.15943); 123->130 (0.14968); 122->127 (0.13225); 123->133 (0.11917)
**38**	200.46	6.1848	0.0193	f < 0.1
**39**	197.67	6.2724	0.0099	f < 0.1
**40**	197.22	6.2866	0.0279	f < 0.1

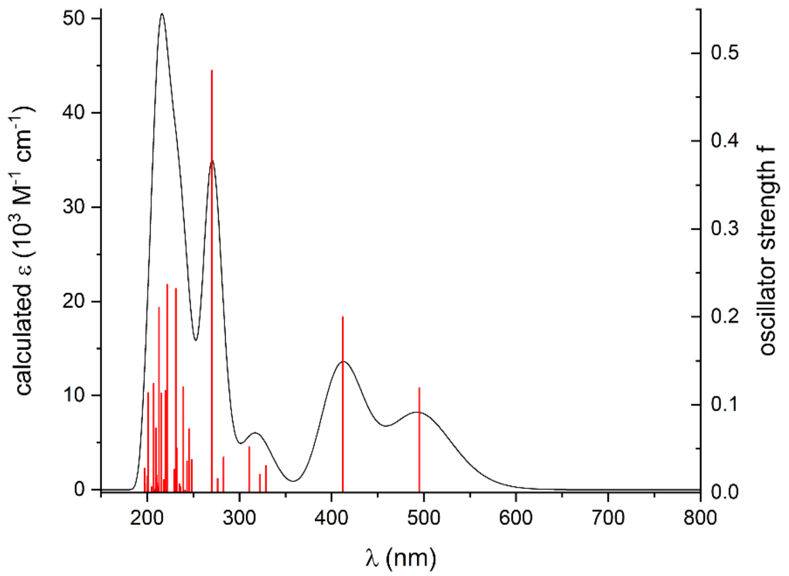

Figure S137. Calculated UV/Vis spectrum (B3LYP, def2-TZVPP, GD3, SCRF for water, TD(NStates=40)) for [**4b-Ph**]$^{2+}$. The line broadening was simulated with a half width of 0.2 eV (1613 cm^{-1}) at the half height of each peak.

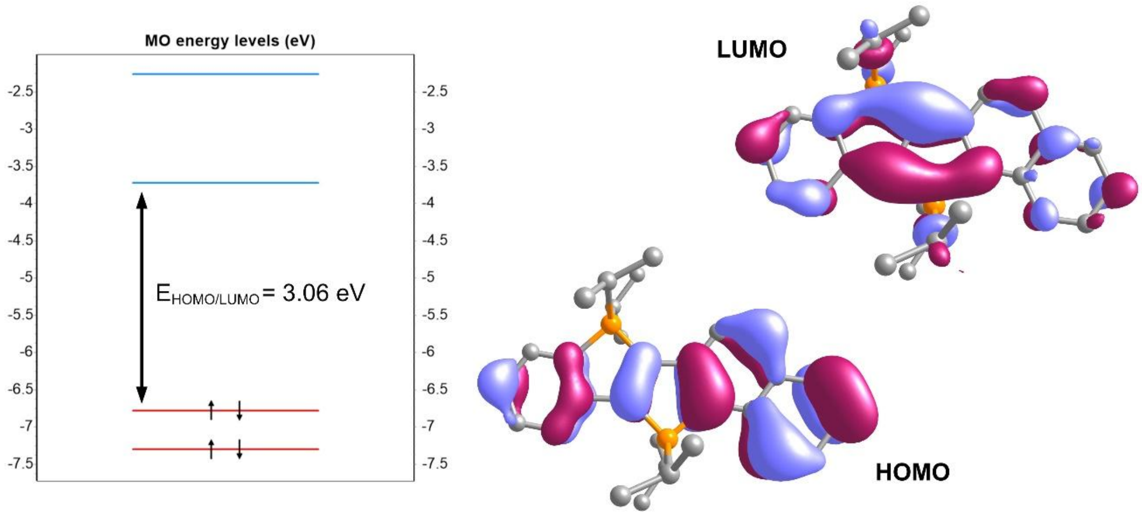

Figure S138. Calculated (B3LYP, def2-TZVPP, GD3, SCRF for water) HOMO LUMO gap for [**4b-Ph**]$^{2+}$ in the **ground state** and Kohn-Sham frontier orbitals for [**4b-Ph**]$^{2+}$. Molecular orbitals were plotted with an isovalue of 0.04. Note that the HOMO LUMO gap in the ground state is **not** a proper estimate for the energy separation between the ground state and the first excited state ($\Delta E_{TD\text{-}DFT}$ $S_0 \rightarrow S_1$ = 2.5048 eV).

Table S31. TD-DFT results for [**4b**]$^{2+}$ (B3LYP, def2-TZVPP, GD3, SCRF for water, TD(NStates=40)).

**excitation**	**λ (nm)**	**E (eV)**	**f**	**orbital contribution for transitions with f > 0.1**
**1**	536.73	2.3100	0.1858	**136 = HOMO** -> **137 = LUMO** (0.70512)
**2**	461.30	2.6877	0.0001	f < 0.1
**3**	414.83	2.9888	0.2567	134->137 (0.6985)
**4**	345.57	3.5878	0.0	f < 0.1
**5**	334.04	3.7116	0.0001	f < 0.1
**6**	302.60	4.0973	0.1172	135->138 (0.64708); 132->137 (0.24695)
**7**	297.41	4.1687	0.1724	136->139 (0.58392); 132->137 (0.33319); 135->141 (0.11159); 133->138 (0.10554)
**8**	286.72	4.3242	0.0	f < 0.1
**9**	283.12	4.3791	0.4090	132->137 (0.56457); 136->139 (-0.31381); 135->138 (-0.2472)
**10**	277.75	4.4639	0.0001	f < 0.1
**11**	262.06	4.7311	0.0831	f < 0.1
**12**	259.94	4.7698	0.0001	f < 0.1
**13**	258.90	4.7889	0.0597	f < 0.1
**14**	249.99	4.9597	0.2654	133->138 (0.52583); 134->139 (-0.26071); 136->140 (0.2597); 135->141 (0.17851); 136->142 (-0.1417); 136->139 (-0.12293)
**15**	247.42	5.0111	0.0053	f < 0.1
**16**	246.03	5.0395	0.0001	f < 0.1
**17**	244.40	5.0730	0.0102	f < 0.1
**18**	242.32	5.1165	0.0063	f < 0.1
**19**	241.53	5.1334	0.3952	134->139 (0.48566); 135->141 (0.33693); 136->140 (0.26351); 134->140 (0.13697); 136->142 (0.11872); 127->137 (0.11034); 130->137 (-0.1032)
**20**	239.78	5.1707	0.0136	f < 0.1
**21**	237.52	5.2201	0.0347	f < 0.1
**22**	237.25	5.2258	0.0	f < 0.1
**23**	236.47	5.2430	0.0001	f < 0.1
**24**	229.29	5.4074	0.0001	f < 0.1
**25**	225.52	5.4977	0.0	f < 0.1
**26**	223.6	5.5450	0.1763	134->140 (0.5915); 133->138 (-0.19851); 134->139 (-0.17317); 133->143 (-0.10328)
**27**	222.92	5.5618	0.0051	f < 0.1
**28**	220.79	5.6156	0.0001	f < 0.1
**29**	218.77	5.6673	0.0001	f < 0.1
**30**	218.56	5.6728	0.4174	131->138 (0.36852); 135->143 (0.34888); 136->144 (-0.33596); 135->141 (-0.18096); 132->139 (-0.13215); 134->140 (0.11666); 134->139 (0.10738)
**31**	217.53	5.6997	0.9280	135->141 (0.49002); 136->144 (-0.33286); 133->138 (-0.18553); 134->140 (-0.18079); 134->139 (-0.17982); 135->143 (0.12964)
**32**	216.53	5.7259	0.0001	f < 0.1
**33**	216.53	5.7260	0.0003	f < 0.1
**34**	215.84	5.7442	0.0074	f < 0.1
**35**	214.67	5.7755	0.0001	f < 0.1
**36**	214.36	5.7840	0.0157	f < 0.1
**37**	212.94	5.8225	0.0	f < 0.1
**38**	212.34	5.8389	0.0113	f < 0.1
**39**	211.82	5.8533	0.0616	f < 0.1
**40**	211.11	5.8731	0.0005	f < 0.1

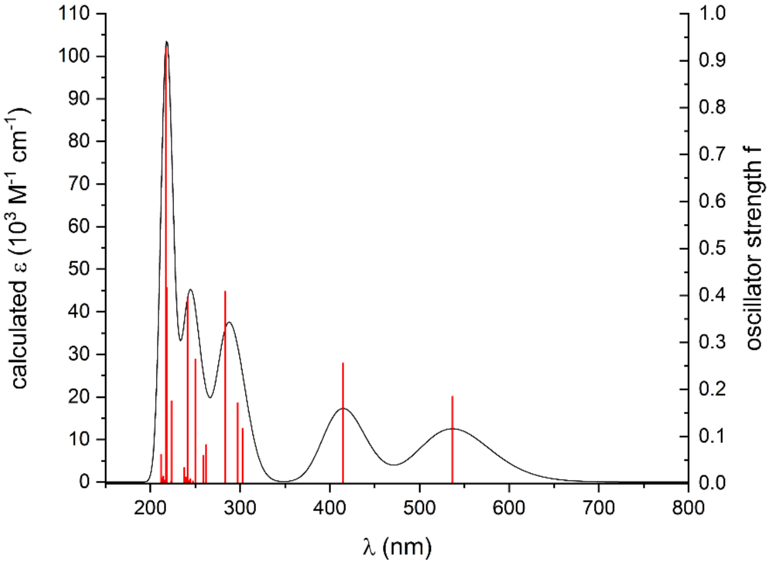

Figure S139. Calculated UV/Vis spectrum (B3LYP, def2-TZVPP, GD3, SCRF for water, TD(NStates=40)) for [**4b**]$^{2+}$. The line broadening was simulated with a half width of 0.2 eV (1613 cm^{-1}) at the half height of each peak.

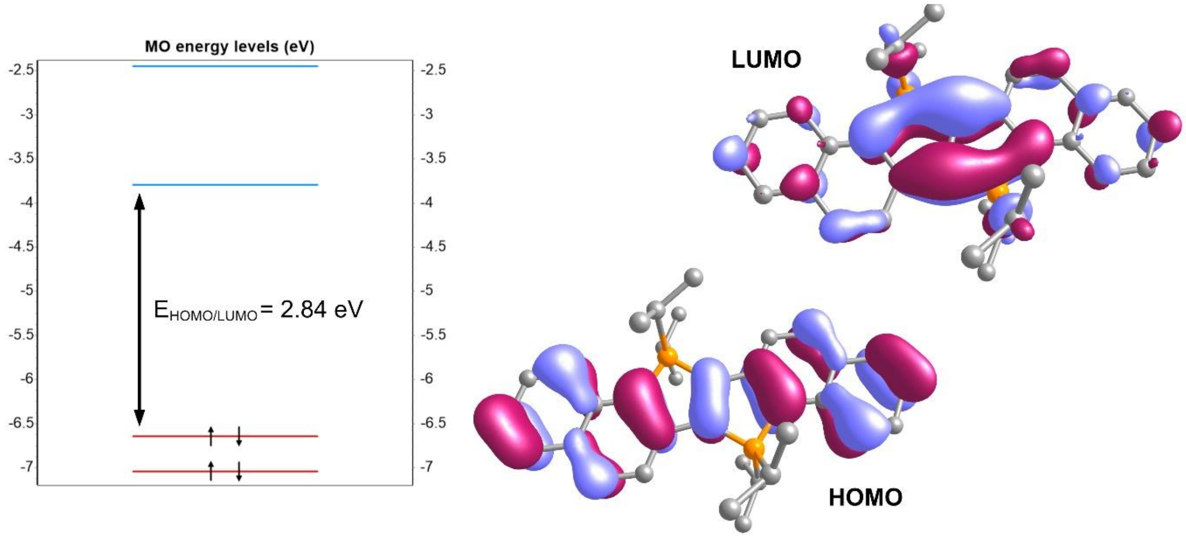

Figure S140. Calculated (B3LYP, def2-TZVPP, GD3, SCRF for water) HOMO LUMO gap for [**4b**]$^{2+}$ in the **ground state** and Kohn-Sham frontier orbitals for [**4b**]$^{2+}$. Molecular orbitals were plotted with an isovalue of 0.04. Note that the HOMO LUMO gap in the ground state is **not** a proper estimate for the energy separation between the ground state and the first excited state ($\Delta E_{TD\text{-}DFT}$ $S_0 \rightarrow S_1$ = 2.3100 eV).

Table S32. TD-DFT results for [**4c-Ph**]$^{2+}$ (B3LYP, def2-TZVPP, GD3, SCRF for water, TD(NStates=40)).

**excitation**	**λ (nm)**	**E (eV)**	**f**	**orbital contribution for transitions with f > 0.1**
**1**	538.77	2.3012	0.3700	**136 = HOMO** -> **137 = LUMO** (0.69918)
**2**	417.97	2.9663	0.0254	f < 0.1
**3**	408.95	3.0318	0.2327	135->137 (0.616); 136->138 (0.3314)
**4**	334.13	3.7107	0.0989	f < 0.1
**5**	327.05	3.791	0.0417	f < 0.1
**6**	315.39	3.9312	0.2073	132->137 (0.51314); 136->139 (0.29995); 135->138 (0.29395); 135->139 (0.11212); 136->140 (-0.10577)
**7**	305.64	4.0566	0.3579	133->137 (0.45833); 132->137 (0.36367); 136->139 (-0.2835); 135->138 (-0.21572); 134->138 (-0.10778)
**8**	301.63	4.1105	0.3994	133->137 (0.50505); 136->139 (0.30304); 132->137 (-0.2587); 135->138 (0.23657)
**9**	287.96	4.3056	0.001	f < 0.1
**10**	274.74	4.5128	0.1538	136->140 (-0.37893); 134->138 (0.37881); 136->141 (-0.34721); 135->139 (-0.19319); 136->142 (0.10554)
**11**	264.23	4.6922	0.0311	f < 0.1
**12**	261.56	4.7402	0.0473	f < 0.1
**13**	260.54	4.7588	0.5762	136->141 (0.3831); 133->138 (-0.35101); 136->142 (-0.30802); 135->139 (-0.1877); 134->138 (0.16693); 136->140 (-0.13728); 135->138 (-0.12059); 135->140 (0.10882)
**14**	254.11	4.8791	0.0036	f < 0.1
**15**	249.26	4.974	0.3238	133->138 (0.43641); 131->137 (0.39045); 136->141 (0.20438); 134->138 (0.15449); 130->137 (-0.12073); 136->140 (-0.11382)
**16**	246.92	5.0213	0.1922	131->137 (0.37719); 136->143 (0.3496); 130->137 (0.34531); 135->140 (-0.18599); 132->138 (-0.17133); 134->138 (-0.1566)
**17**	246.71	5.0255	0.0543	f < 0.1
**18**	244.03	5.0807	0.0846	f < 0.1
**19**	237.45	5.2214	0.1018	129->137 (0.62382); 135->140 (-0.24309); 127->137 (-0.12968); 136->143 (-0.11577)
**20**	235.61	5.2622	0.1134	135->140 (0.49143); 129->137 (0.28931); 136->143 (0.21112); 134->139 (0.20901)
**21**	231.64	5.3524	0.0037	f < 0.1
**22**	231.12	5.3644	0.0088	f < 0.1
**23**	230.3	5.3837	0.0227	f < 0.1
**24**	228.66	5.4221	0.0064	f < 0.1
**25**	226.81	5.4665	0.0488	f < 0.1
**26**	226.03	5.4852	0.0002	f < 0.1
**27**	225.03	5.5096	0.0036	f < 0.1
**28**	224.23	5.5294	0.0084	f < 0.1
**29**	223.15	5.5561	0.0021	f < 0.1
**30**	219.46	5.6495	0.0092	f < 0.1
**31**	219.11	5.6585	0.0028	f < 0.1
**32**	218.03	5.6867	0.0117	f < 0.1
**33**	216.67	5.7222	0.0571	f < 0.1
**34**	214.47	5.7811	0.0079	f < 0.1
**35**	213.24	5.8143	0.0360	f < 0.1
**36**	211.84	5.8527	0.0633	f < 0.1
**37**	211.23	5.8696	0.0014	f < 0.1
**38**	208.97	5.933	0.0078	f < 0.1
**39**	208.77	5.9387	0.0039	f < 0.1
**40**	206.33	6.0091	0.0342	f < 0.1

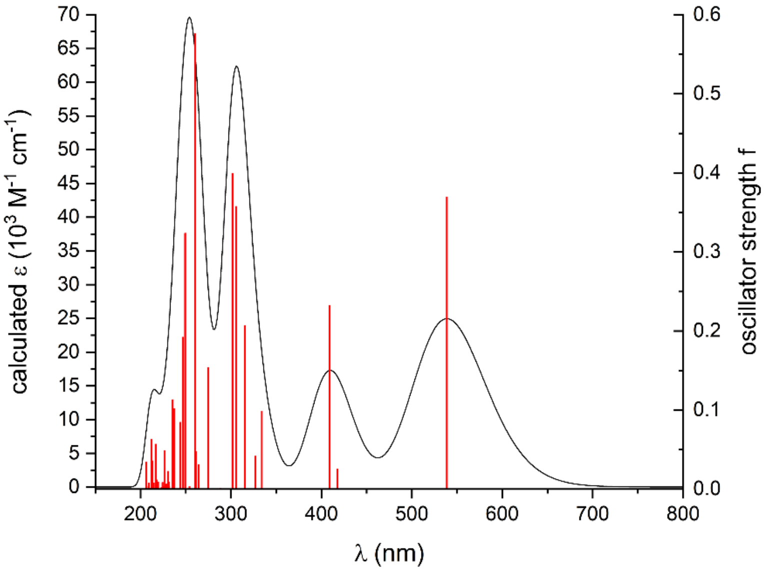

Figure S141. Calculated UV/Vis spectrum (B3LYP, def2-TZVPP, GD3, SCRF for water, TD(NStates=40)) for [**4c-Ph**]$^{2+}$. The line broadening was simulated with a half width of 0.2 eV (1613 cm^{-1}) at the half height of each peak.

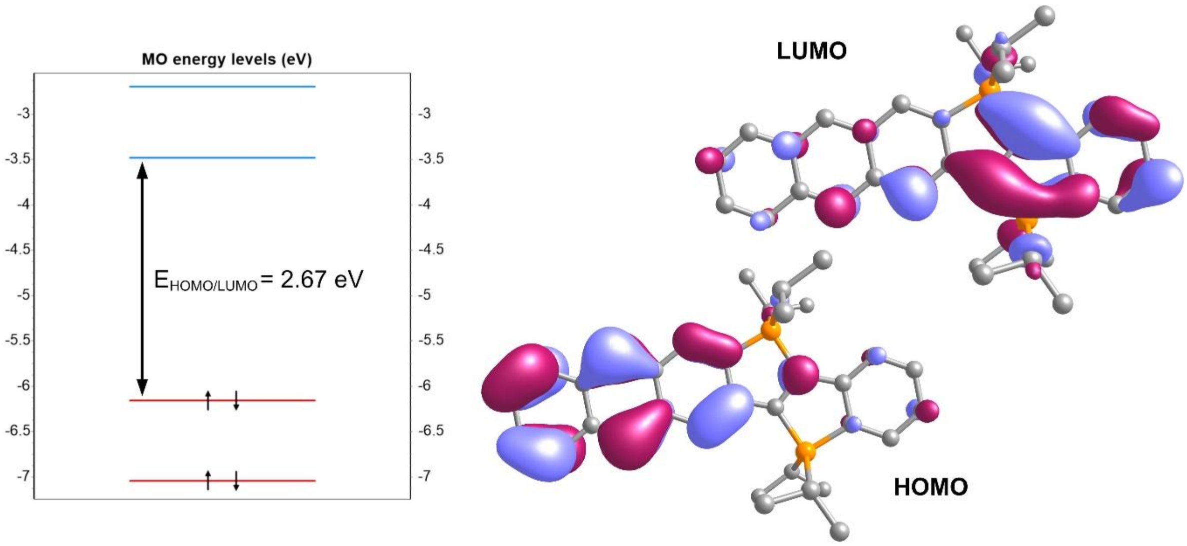

Figure S142. Calculated (B3LYP, def2-TZVPP, GD3, SCRF for water) HOMO LUMO gap for [**4c-Ph**]$^{2+}$ in the **ground state** and Kohn-Sham frontier orbitals for [**4c-Ph**]$^{2+}$. Molecular orbitals were plotted with an isovalue of 0.04. Note that the HOMO LUMO gap in the ground state is **not** a proper estimate for the energy separation between the ground state and the first excited state ($\Delta E_{TD\text{-}DFT}$ $S_0 \rightarrow S_1$ = 2.3012 eV).

Table S33. TD-DFT results for [**4c-Naph**]$^{2+}$ (B3LYP, def2-TZVPP, GD3, SCRF for water, TD(NStates=40)).

excitation	λ (nm)	E (eV)	f	orbital contribution for transitions with f > 0.1
**1**	527.46	2.3506	0.5390	**149 = HOMO** -> **150 = LUMO** (0.6969)
**2**	428.09	2.8962	0.2922	148->150 (0.68971)
**3**	421.75	2.9397	0.0192	f < 0.1
**4**	380.94	3.2547	0.0496	f < 0.1
**5**	358.40	3.4594	0.0694	f < 0.1
**6**	343.41	3.6104	0.4127	148->151 (0.64658); 146->150 (0.18657)
**7**	327.87	3.7816	0.0962	f < 0.1
**8**	311.3	3.9828	0.1318	147->151 (0.43461); 148->152 (-0.30199); 149->153 (-0.29971); 146->151 (0.18149); 145->150 (-0.16892); 146->150 (-0.15433); 148->151 (0.11751)
**9**	308.71	4.0163	0.0063	f < 0.1
**10**	304.39	4.0733	0.0326	f < 0.1
**11**	295.06	4.202	0.5481	149->153 (0.42475); 147->151 (0.38319); 146->151 (-0.29729); 147->152 (-0.22242)
**12**	279.14	4.4417	0.1430	147->152 (0.42127); 146->151 (-0.42027); 148->153 (-0.19373); 149->156 (0.14789); 149->153 (-0.12989); 146->152 (-0.1228); 145->151 (-0.12276); 147->151 (0.10943)
**13**	272.90	4.5432	0.0332	f < 0.1
**14**	266.74	4.6482	0.2213	144->150 (0.56219); 145->151 (0.29069); 148->153 (-0.14005); 149->156 (0.11197); 147->152 (0.11131); 146->151 (0.10455)
**15**	264.86	4.6812	0.2915	145->151 (0.38778); 149->154 (-0.3407); 144->150 (-0.31389); 148->153 (-0.18438); 149->153 (0.15536); 147->152 (0.11467); 147->151 (0.10458); 149->156 (-0.10034)
**16**	262.75	4.7188	0.2348	148->153 (0.36801); 146->152 (0.31421); 145->151 (0.24078); 146->151 (-0.22043); 147->152 (0.2104); 149->155 (0.18955); 145A>152 (-0.10498)
**17**	262.29	4.7271	0.0045	f < 0.1
**18**	258.20	4.8019	0.0141	f < 0.1
**19**	250.49	4.9496	0.4098	143->150 (0.4069); 149->156 (0.3395); 146->152 (0.25991); 146->151 (0.15776); 149->155 (-0.13499); 148->153 (0.13352); 147->152 (0.13334); 145->151 (-0.11732); 149->154 (-0.11441)
**20**	247.40	5.0116	0.3618	143->150 (0.42466); 146->152 (-0.26026); 149->156 (-0.24215); 147->153 (-0.23419); 148->153 (0.14744); 148->156 (0.13443); 144->150 (0.1255); 146->151 (-0.10339); 149->155 (0.1005)
**21**	245.08	5.059	0.0591	f < 0.1
**22**	243.20	5.098	0.0034	f < 0.1
**23**	240.33	5.159	0.1439	145->152 (0.4487); 146->152 (0.35558); 147->153 (-0.30111); 148->156 (0.12239)
**24**	234.30	5.2917	0.0500	f < 0.1
**25**	234.06	5.2971	0.0046	f < 0.1
**26**	233.14	5.3181	0.0909	f < 0.1
**27**	232.33	5.3364	0.0107	f < 0.1
**28**	231.37	5.3586	0.0006	f < 0.1
**29**	230.29	5.3839	0.1603	149->157 (0.42798); 147->153 (-0.40873); 145->152 (-0.24865); 148->154 (-0.16998); 149->159 (-0.10299)
**30**	228.65	5.4224	0.0516	f < 0.1
**31**	228.13	5.4348	0.0631	f < 0.1
**32**	226.84	5.4656	0.0035	f < 0.1
**33**	225.13	5.5071	0.0038	f < 0.1
**34**	224.48	5.5231	0.0122	f < 0.1
**35**	223.91	5.5371	0.1085	148->154 (0.34631); 149->159 (0.26557); 144->151 (-0.2303); 149->160 (-0.23006); 140->150 (0.19928); 148->156 (-0.18539); 146->153 (0.12399); 137->150 (-0.11184); 144->152 (0.10874); 143->151 (0.10668); 147->153 (-0.10644)
**36**	222.65	5.5685	0.0655	f < 0.1
**37**	221.68	5.5929	0.0059	f < 0.1
**38**	219.69	5.6437	0.0041	f < 0.1
**39**	218.75	5.668	0.0610	f < 0.1
**40**	216.95	5.7148	0.0095	f < 0.1

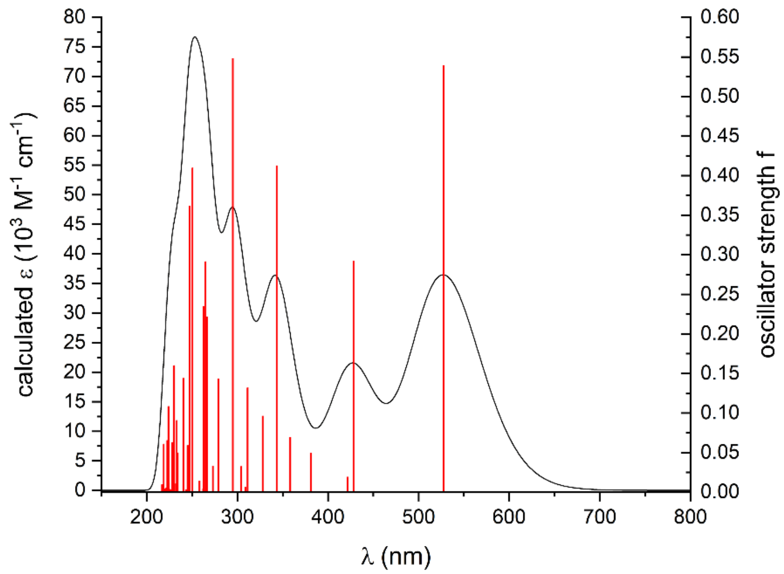

Figure S143. Calculated UV/Vis spectrum (B3LYP, def2-TZVPP, GD3, SCRF for water, TD(NStates=40)) for [**4c-Naph**]$^{2+}$. The line broadening was simulated with a half width of 0.2 eV (1613 cm^{-1}) at the half height of each peak.

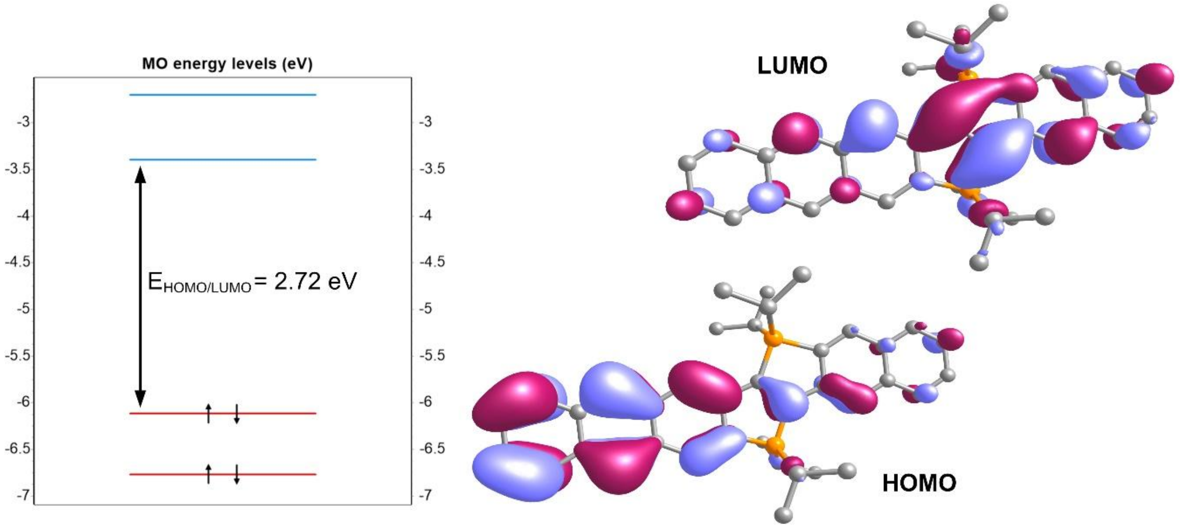

Figure S144. Calculated (B3LYP, def2-TZVPP, GD3, SCRF for water) HOMO LUMO gap for [**4c-Naph**]$^{2+}$ in the **ground state** and Kohn-Sham frontier orbitals for [**4c-Naph**]$^{2+}$. Molecular orbitals were plotted with an isovalue of 0.04. Note that the HOMO LUMO gap in the ground state is **not** a proper estimate for the energy separation between the ground state and the first excited state ($\Delta E_{TD\text{-}DFT}$ $S_0 \rightarrow S_1$ = 2.3506 eV).

Table S34. TD-DFT results for [**4c-Anth**]$^{2+}$ (B3LYP, def2-TZVPP, GD3, SCRF for water, TD(NStates=40)).

**excitation**	**λ (nm)**	**E (eV)**	**f**	**orbital contribution for transitions with f > 0.1**
**1**	542.48	2.2855	0.8911	**162 = HOMO** -> **163 = LUMO** (0.69412); 162 ->164 (-0.10234)
**2**	494.34	2.5081	0.0	f < 0.1
**3**	442.59	2.8013	0.0376	f < 0.1
**4**	414.57	2.9907	0.0001	f < 0.1
**5**	398.71	3.1096	0.2301	160->163 (0.69073)
**6**	392.56	3.1584	0.0004	f < 0.1
**7**	369.37	3.3566	0.3816	161->165 (0.68943)
**8**	334.94	3.7017	0.0	f < 0.1
**9**	331.64	3.7386	0.8823	160->164 (0.65813); 161->166 (0.16202); 162->168 (-0.113); 159->165 (-0.10272)
**10**	323.14	3.8369	0.0007	f < 0.1
**11**	311.03	3.9862	0.062	f < 0.1
**12**	302.16	4.1033	0.0	f < 0.1
**13**	295.08	4.2018	0.1928	16A->166 (0.43836); 159->165 (-0.32663); 158->164 (0.2298); 160->164 (-0.18972); 162->168 (-0.17795); 160->165 (0.1523); 159->164 (0.1435)
**14**	294.26	4.2134	0.0322	f < 0.1
**15**	279.55	4.4351	0.0004	f < 0.1
**16**	276.41	4.4855	0.1815	162->167 (0.62468); 161->170 (0.17745); 158->164 (-0.15172); 162->168 (-0.14751)
**17**	273.61	4.5315	0.6156	158->164 (0.47309); 161->166 (-0.40867); 162->168 (-0.20494); 157->165 (0.13756); 159->165 (-0.13689)
**18**	268.61	4.6157	0.0017	f < 0.1
**19**	267.67	4.632	0.0008	f < 0.1
**20**	265.91	4.6626	0.0186	f < 0.1
**21**	265.29	4.6736	0.4172	156->163 (0.47162); 159->165 (-0.41461); 158->164 (-0.21073); 161->166 (-0.12712); 162->168 (0.10263)
**22**	262.46	4.7239	0.0003	f < 0.1
**23**	258.53	4.7957	0.0002	f < 0.1
**24**	255.53	4.8521	1.3178	162->168 (0.49708); 156->163 (-0.31523); 159->165 (-0.26611); 157->165 (0.13183); 160->167 (-0.10403)
**25**	251.13	4.9371	0.0002	f < 0.1
**26**	250.07	4.958	0.0015	f < 0.1
**27**	249.04	4.9784	0.0991	f < 0.1
**28**	248.57	4.9879	0.0009	f < 0.1
**29**	243.81	5.0852	0.0881	f < 0.1
**30**	243.08	5.1006	0.0084	f < 0.1
**31**	241.85	5.1264	0.2097	161->170 (0.55499); 154->163 (0.24462); 155->163 (0.17879); 157->165 (0.16959); 162->167 (-0.12228); 161->169 (0.11295)
**32**	239.75	5.1715	0.0005	f < 0.1
**33**	236.82	5.2355	0.0013	f < 0.1
**34**	235.37	5.2676	0.0692	f < 0.1
**35**	232.53	5.3319	0.0384	f < 0.1
**36**	231.92	5.3459	0.0018	f < 0.1
**37**	229.87	5.3936	0.0051	f < 0.1
**38**	228.16	5.4342	0.0007	f < 0.1
**39**	227.98	5.4383	0.0011	f < 0.1
**40**	227.12	5.4591	0.0131	f < 0.1

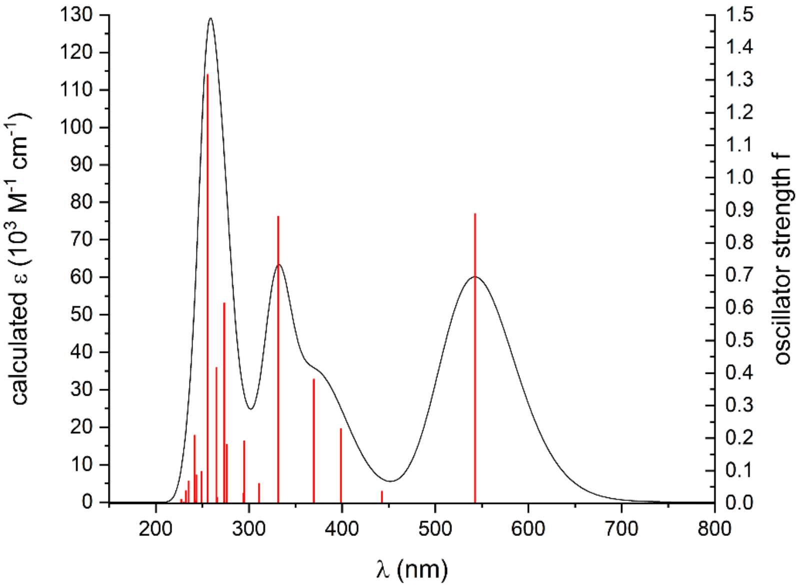

Figure S145. Calculated UV/Vis spectrum (B3LYP, def2-TZVPP, GD3, SCRF for water, TD(NStates=40)) for [**4c-Anth**]$^{2+}$. The line broadening was simulated with a half width of 0.2 eV (1613 cm^{-1}) at the half height of each peak.

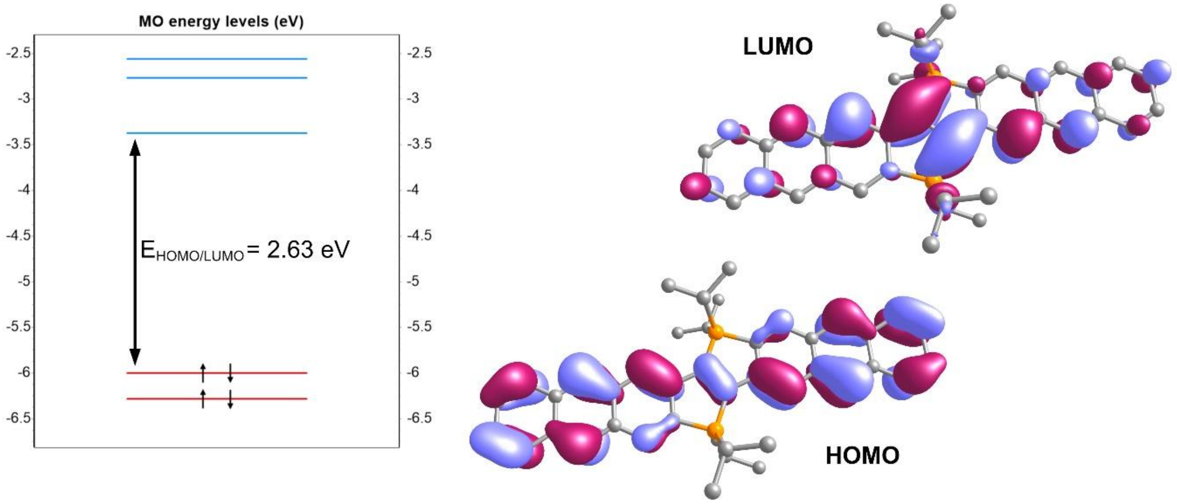

Figure S146. Calculated (B3LYP, def2-TZVPP, GD3, SCRF for water) HOMO LUMO gap for [**4c-Anth**]$^{2+}$ in the **ground state** and Kohn-Sham frontier orbitals for [**4c-Anth**]$^{2+}$. Molecular orbitals were plotted with an isovalue of 0.04. Note that the HOMO LUMO gap in the ground state is **not** a proper estimate for the energy separation between the ground state and the first excited state ($\Delta E_{TD\text{-}DFT}$ $S_0 \rightarrow S_1$ = 2.2855 eV).

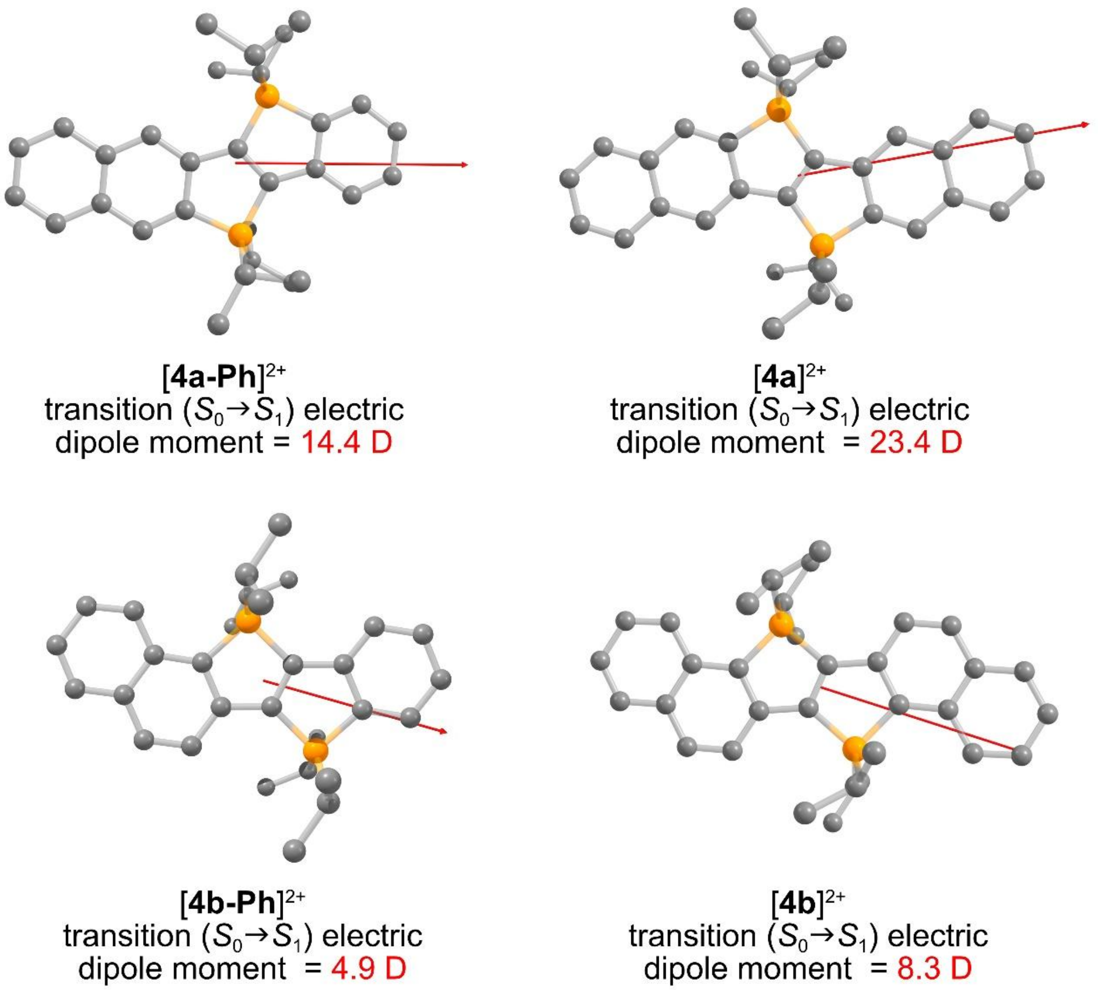

Figure S147. Calculated (B3LYP, def2-TZVPP, GD3, SCRF for water) electrical transition dipole vectors for the excitation from the singlet ground state (S_0) to the first excited singlet state (S_1) for [**4a-Ph**]$^{2+}$, [**4a**]$^{2+}$, [**4b-Ph**]$^{2+}$ and [**4b**]$^{2+}$. Note that the length of each vector was normalized for printing and only meant to illustrate the directionality. Numerical values (in Debye) are provided for each compound.

Table S35. Ground (S_0) to excited state (S_1) transition electric dipole moments (B3LYP, def2-TZVPP, GD3, SCRF for water, TD(NStates=40)) for compounds [**4a-Ph**]$^{2+}$, [**4a**]$^{2+}$, [**4b-Ph**]$^{2+}$ and [**4b**]$^{2+}$.

compound	X	Y	Z	dipole strength (a.u.)	dipole strength (D)	oscillator strength f
[**4a-Ph**]$^{2+}$	−0.4854	−2.3334	0.0002	5.6805	14.438	0.3839
[**4a**]$^{2+}$	0.4474	3.0026	0.0103	9.2158	23.424	0.6105
[**4b-Ph**]$^{2+}$	0.9777	−0.9951	0.0188	1.9464	4.947	0.1194
[**4b**]$^{2+}$	1.8066	0.1375	−0.0013	3.2827	8.344	0.1858

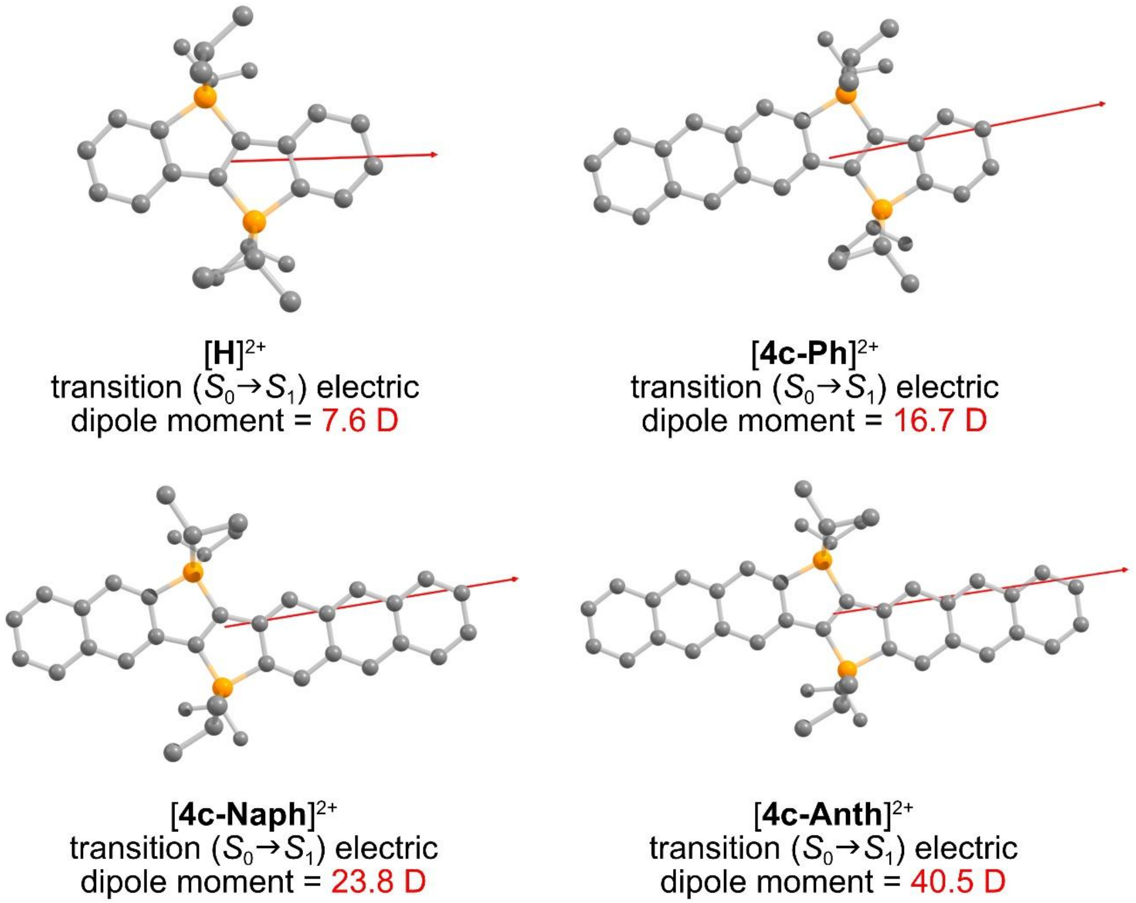

Figure S148. Calculated (B3LYP, def2-TZVPP, GD3, SCRF for water) electrical transition dipole vectors for the excitation from the singlet ground state (S_0) to the first excited singlet state (S_1) for [**H**]$^{2+}$, [**4c-Ph**]$^{2+}$, [**4c-Naph**]$^{2+}$ and [**4c-Anth**]$^{2+}$. Note that the length of each vector was normalized for printing and only meant to illustrate the directionality. Numerical values (in Debye) are provided for each compound.

Table S36. Ground (S_0) to excited state (S_1) transition electric dipole moments (B3LYP, def2-TZVPP, GD3, SCRF for water, TD(NStates=40)) for compounds [**H**]$^{2+}$, [**4c-Ph**]$^{2+}$, [**4c-Naph**]$^{2+}$ and [**4c-Anth**]$^{2+}$.

compound	X	Y	Z	dipole strength (a.u.)	dipole strength (D)	oscillator strength *f*
[**H**]$^{2+}$	−0.6027	−1.6224	−0.0028	2.9956	7.614	0.2167
[**4c-Ph**]$^{2+}$	2.0559	1.5286	0.0013	6.5631	16.682	0.3700
[**4c-Naph**]$^{2+}$	0.4095	3.0318	−0.0035	9.3592	23.789	0.5390
[**4c-Anth**]$^{2+}$	0.4750	3.9609	−0.0026	15.9143	40.450	0.8911

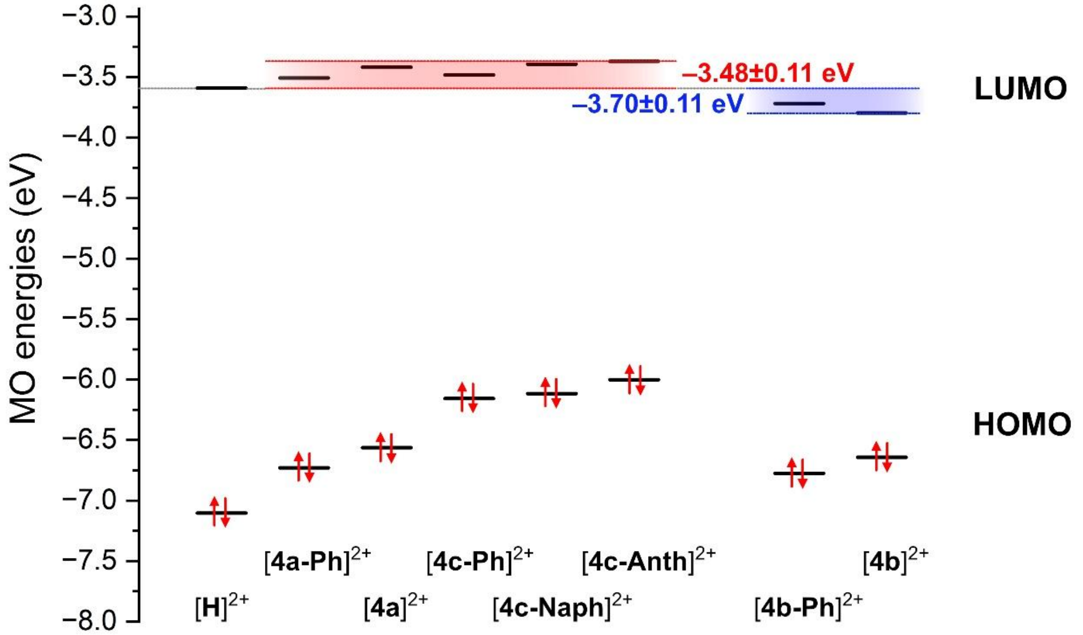

Figure S149. Calculated (B3LYP, def2-TZVPP, GD3, SCRF for water) HOMO-LUMO gaps for all the phospholo[3,2-*b*]phosphole dications reported herein. Upon extension of the π-system, the LUMO energies are nearly unaffected for the linear compounds (−3.48±0.11 eV). The angular compound exhibit similar LUMO energies as well (−3.70±0.11 eV), albeit at slightly lower energies in comparison to the linear derivatives. As expected the HOMO energies are raising upon extension of the π-system, not only within the linear series, but also when going from [**4b-Ph**]$^{2+}$ to [**4b-Ph**]$^{2+}$.

6) Electrochemical Measurements

Cyclovoltammetry measurements were carried out under an argon atmosphere using a PalmSens EmStat3 Blue potentiostat. Tetrabutylammonium triflate (0.1 M in anhydrous MeCN) was used as electrolyte. A glassy carbon electrode was used as working electrode (electrode tip with a graphite disc, 0.5 mm^2 surface area). A platinum wire and a silver wire was used as counter and reference electrode, respectively. The working electrode was polished with an aqueous suspension of Al_2O_3 (0.3 µm particle size) and the platinum electrode was heated with the flame of a Bunsen burner.

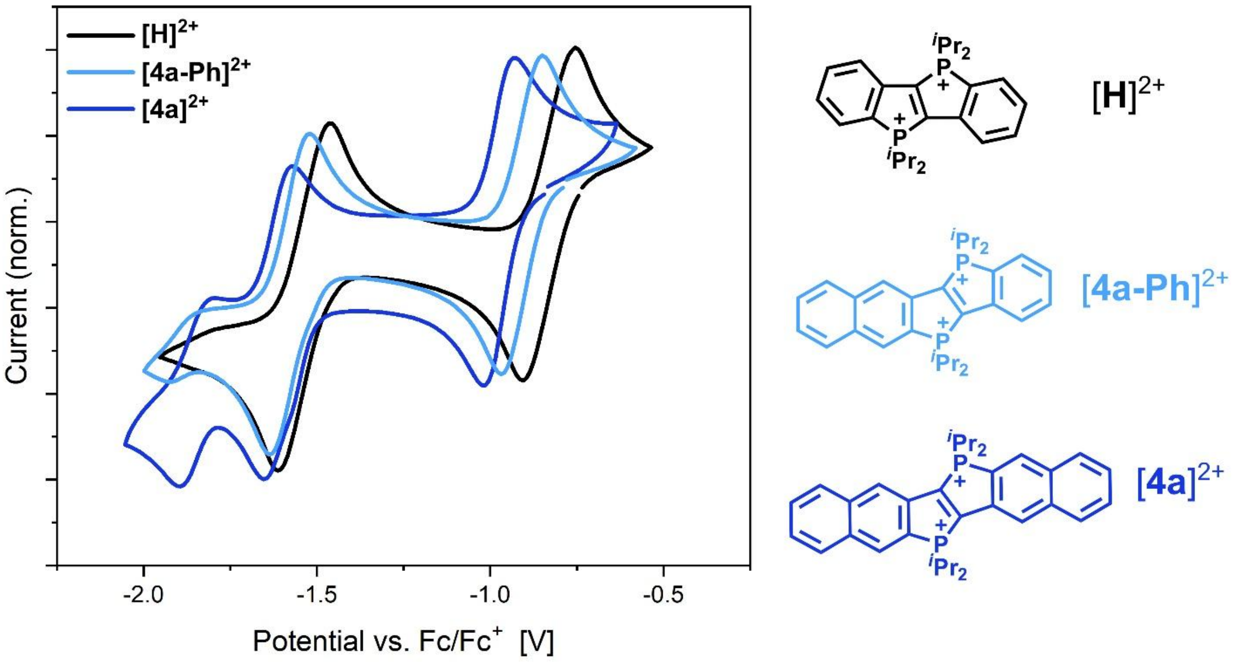

Figure S150. Cyclic voltammograms of [**H**]Cl_2, [**4a-Ph**]Cl_2 and [**4a**]Cl_2 in MeCN/ 0.1 M NBu_4OTf. Potentials are referrenced vs. Fc/Fc^+.

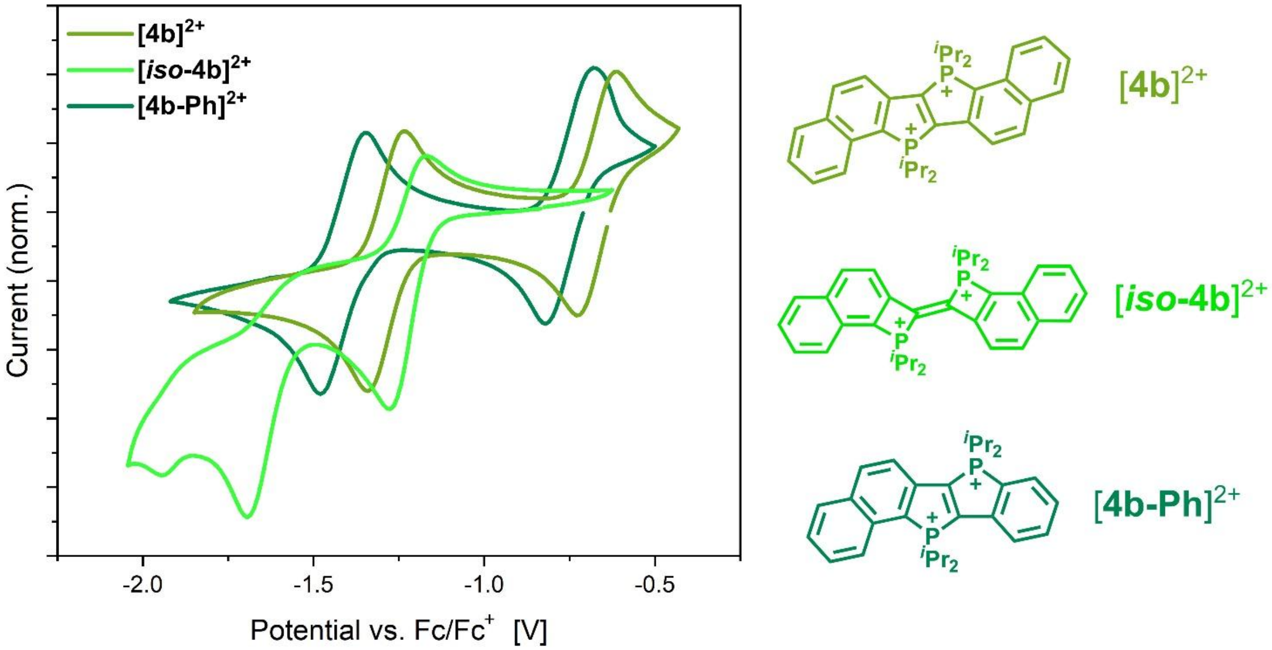

Figure S151. Cyclic voltammograms of [**4b**]Cl_2, [***iso*-4b**]Cl_2 and [**4b-Ph**]Cl_2 in MeCN/ 0.1 M NBu_4OTf. Potentials are referrenced vs. Fc/Fc^+.

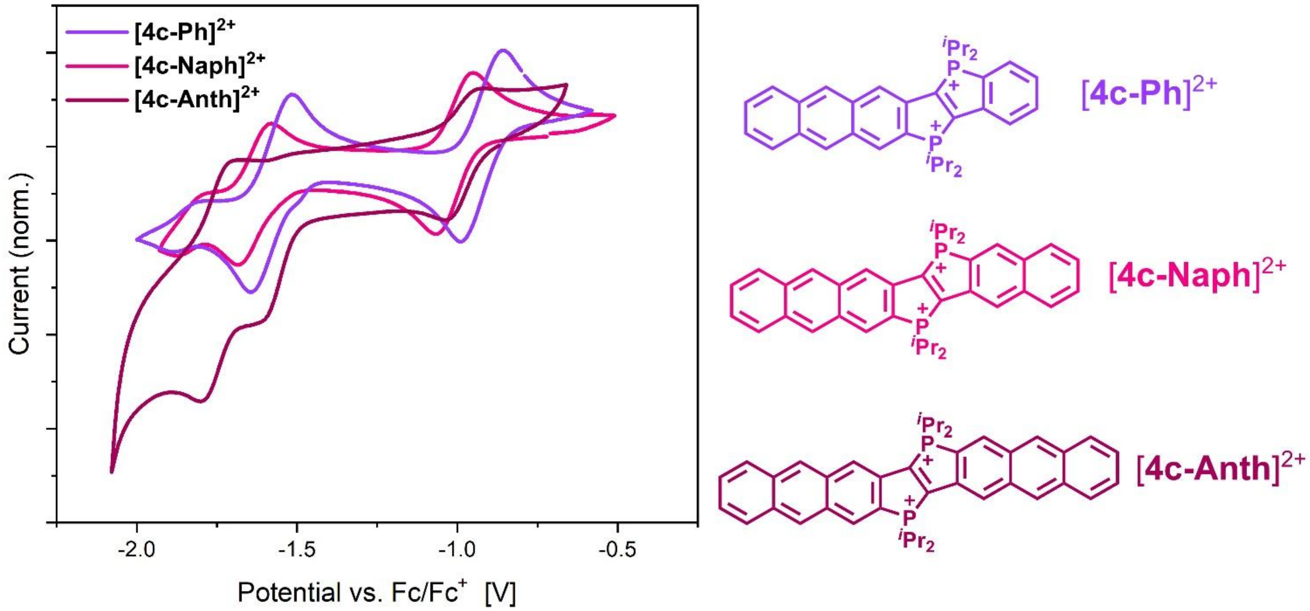

Figure S152. Cyclic voltammograms of [**4c-Ph**]Cl_2, [**4c-Naph**]Cl_2 and [**4c-Anth**]Cl_2 in MeCN/ 0.1 M NBu_4OTf. Potentials are referrenced vs. Fc/Fc^+.

Table S37. Redox potentials (vs. Fc/Fc^+) for [**H**]Cl_2, [**4a-Ph**]Cl_2, [**4a**]Cl_2, [**4b**]Cl_2, [***iso*-4b**]Cl_2, [**4b-Ph**]Cl_2, [**4c-Ph**]Cl_2, [**4c-Naph**]Cl_2 and [**4c-Anth**]Cl_2. All measurements were conducted in MeCN/0.1 M NBu_4OTf.

compound	E(V)[a]
[**H**]$^{2+}$	$E_{1/2}$ = –1.54 V, **$E_{1/2}$ = –0.83 V**
[**4a-Ph**]$^{2+}$	$E_{1/2}$ = –1.58 V, **$E_{1/2}$ = –0.91 V**
[**4a**]$^{2+}$	$E_{0.5}$ = –1.84 V (irrev.), $E_{1/2}$ = –1.61 V, **$E_{1/2}$ = –0.97 V**
[**4b-Ph**]$^{2+}$	$E_{1/2}$ = –1.42 V, **$E_{1/2}$ = –0.75 V**
[**4b**]$^{2+}$	$E_{1/2}$ = –1.29 V, **$E_{1/2}$ = –0.67 V**
[***iso*-4b**]$^{2+}$	E = –1.94 V (irrev.), E = –1.61 V (irrev.), $E_{1/2}$ = –1.22 V[b]
[**4c-Ph**]$^{2+}$	$E_{1/2}$ = –1.58 V, **$E_{1/2}$ = –0.93 V**
[**4c-Nap**]$^{2+}$	$E_{1/2}$ = –1.63 V, **$E_{1/2}$ = –1.01 V**
[**4c-Anth**]$^{2+}$	$E_{1/2}$ = –1.74 V, E = –1.64 V (irrev.), **$E_{1/2}$ = –0.98 V**

[a] values in bold are assigned to the dication/monocation couple. [b] As shown in the article, ring-opening occurs upon chemical reduction of [***iso*-4b**]$^{2+}$. Hence, the individual redox waves cannot be assigned inevitability.

6.1) Counterion effect on the electrochemical oxidation

The cyclic voltammogram of **[4c-Ph]Cl$_2$** exhibits an oxidation signal at **E$_{1/2}$** = +494 mV vs. Fc/Fc$^+$ with a pronounced irreversible character. Comparable signals, though quasi-reversible in nature, are observed for **[4a-Ph]Cl$_2$** (**E$_{1/2}$** = +505 mV vs. Fc/Fc$^+$) and **[H]Cl$_2$** (**E$_{1/2}$** = +474 mV vs. Fc/Fc$^+$), whereas no such signal is detected for **[H](OTf)$_2$**. These findings suggest that the additional oxidation process originates from the chloride counterion rather than from oxidation of the phospholo[3,2-b]phosphole dications. The potential of chloride in pure acetonitrile, determined using Marcus theory and excited state quenching rates of iridium complexes, is in a similar potential region and amounts to **E$_{1/2}$** = +830 mV vs. Fc/Fc$^+$[51] (originally published as **E$_{1/2}$** = +1.46 V vs. NHE)[52]. This ~0.3 V discrepancy might be due to ion-pairing and counterion effects.

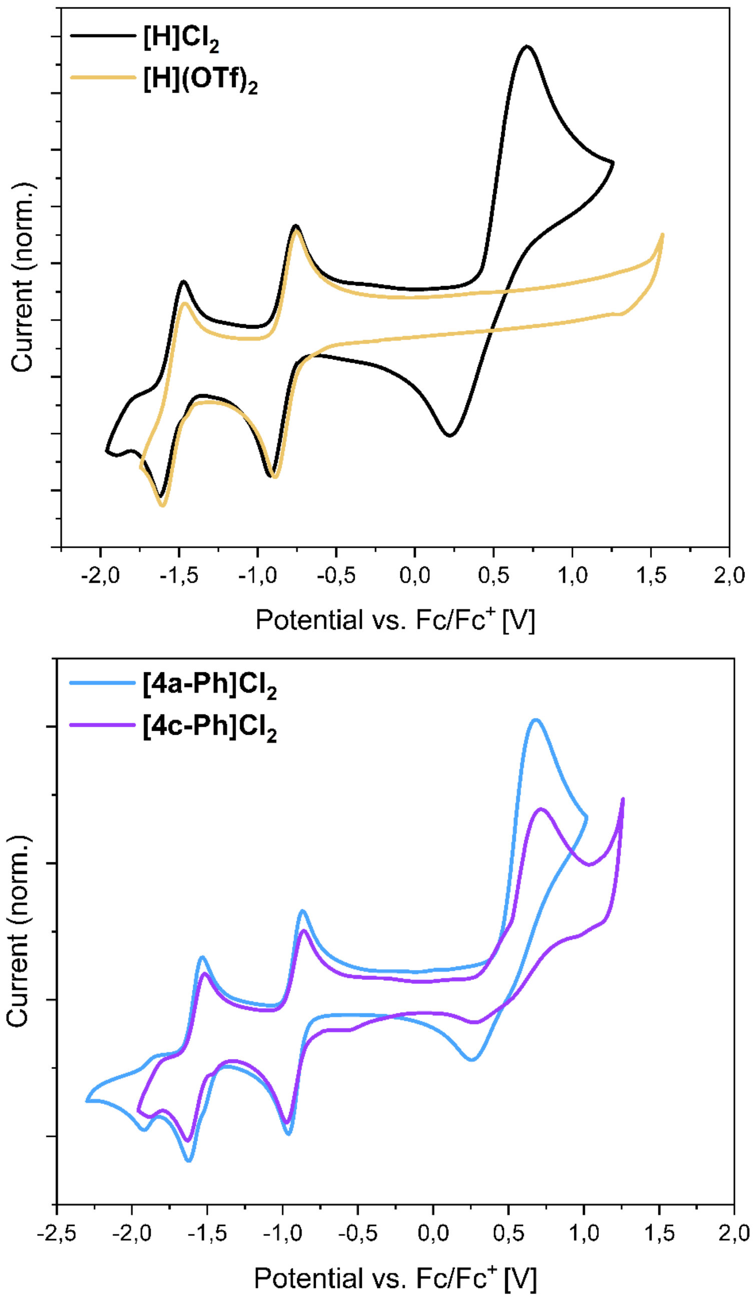

Figure S153. Cyclic voltammograms of [**H**]Cl$_2$, [**H**](OTf)$_2$, [**4a-Ph**]Cl$_2$ and [**4c-Ph**]Cl$_2$ in MeCN/ 0.1 M NBu$_4$OTf. Potentials are referrenced vs. Fc/Fc$^+$.

7) X-Ray Crystal Structure Determinations

Crystal data and details of the structure determinations are compiled in Table S38, Table S39, Table S40 and Table S41. Full shells of intensity data were collected at 120(1) K with an Agilent Technologies Supernova-E CCD diffractometer (Cu-K_α radiation, microfocus X-ray tube, multilayer mirror optics). Detector frames (typically ω-, occasionally φ-scans, scan width 1.0°) were integrated by profile fitting.[40] Data were corrected for air and detector absorption, Lorentz and polarization effects[41,42] and scaled essentially by application of appropriate spherical harmonic functions.[43,44,45] Absorption by the crystal was treated numerically (Gaussian grid).[45,46] An illumination correction was performed as part of the numerical absorption correction.[45]

Using OLEX2,[47] the structures were solved with SHELXT[48] (intrinsic phasing) and refined with SHELXL[49] by full-matrix least squares methods based on F^2 against all unique reflections. All non-hydrogen atoms were given anisotropic displacement parameters. Hydrogen atoms were generally input at calculated positions and refined with a riding model.[50] Split atom models were used to refine disordered groups and/or solvent molecules. When found necessary, suitable geometry and adp restraints were applied.[50]

CCDC 2431371-2431379 and 2470828 contain the supplementary crystallographic data for this paper. These data can be obtained free of charge from the Cambridge Crystallographic Data Centre's and FIZ Karlsruhe’s joint Access Service via https://www.ccdc.cam.ac.uk.

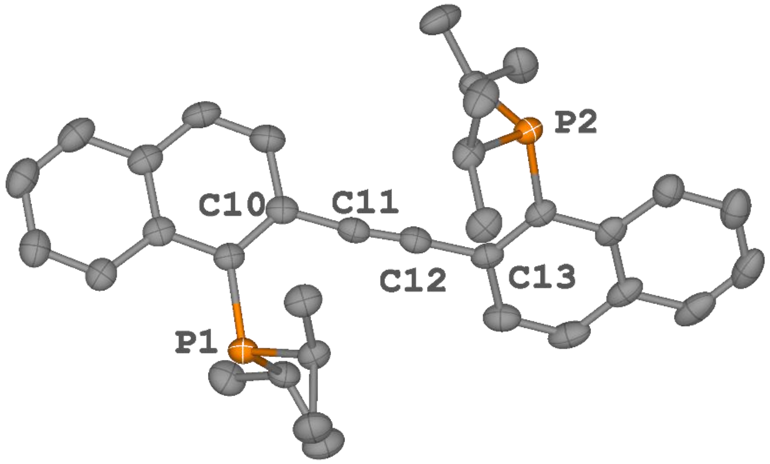

Figure S154. ORTEP plot of the molecular structure of **3b** (thermal ellipsoids set to 50% probability; hydrogen atoms omitted for clarity; only one out of two independent molecules present in the asymmetric unit is show,). Selected bond lengths (Å) and angles (°) [values for the second molecule are given in square brackets]: C13–C12 1.441(6) [1.440(5)], C12–C11 1.208(6) [1.205(6)], C11–C10 1.438(6) [1.435(5)], C11–C12–C13 174.7(4) [174.7(4)], C12–C11–C10 173.2(4) [172.0(4)].

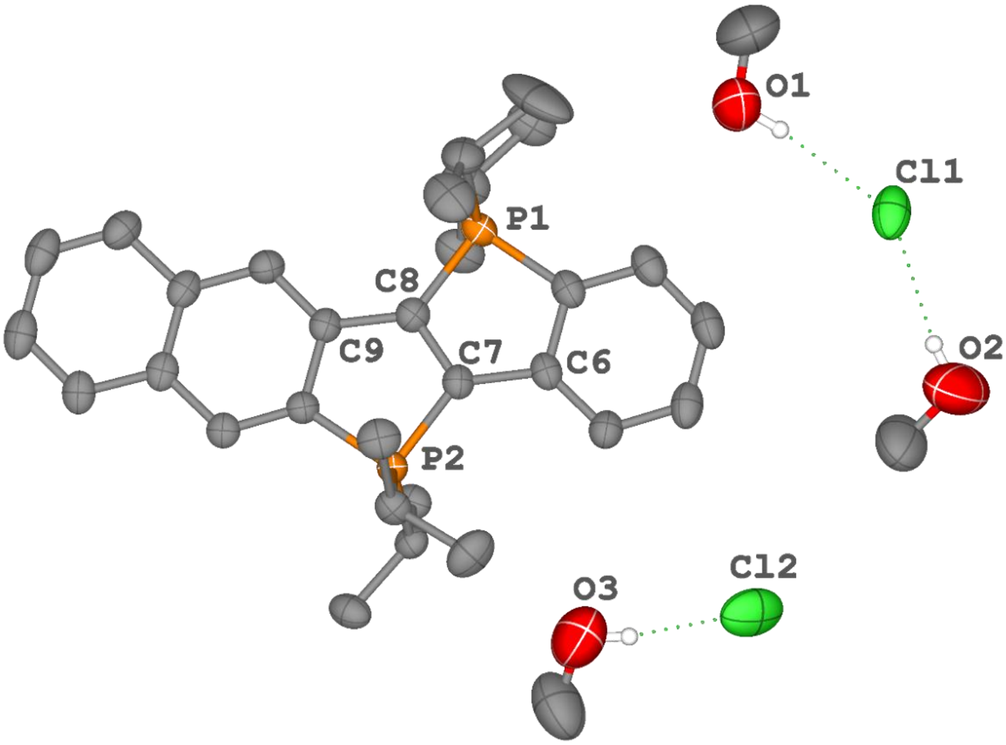

Figure S155. ORTEP plot of the molecular structure of [**4a-Ph**]Cl_2 × 3 MeOH (thermal ellipsoids set to 50% probability; disorder omitted for clarity; hydrogen atoms −except protic hydrogens− omitted for clarity). Selected bond lengths (Å) and angles (°): P2–C7 1.799(4), P1–C8, 1.804(4), C7–C8 1.360(5), C7–C6 1.463(5), C9–C8 1.455(5), C8–C7–P2 109.0(3), C6–C7–P2 134.2(3), C7–C8–P1 108.8(3), C9–C8–P1 133.9(3), C8–C7–C6 116.9(3), C7–C8–C9 117.2(3).

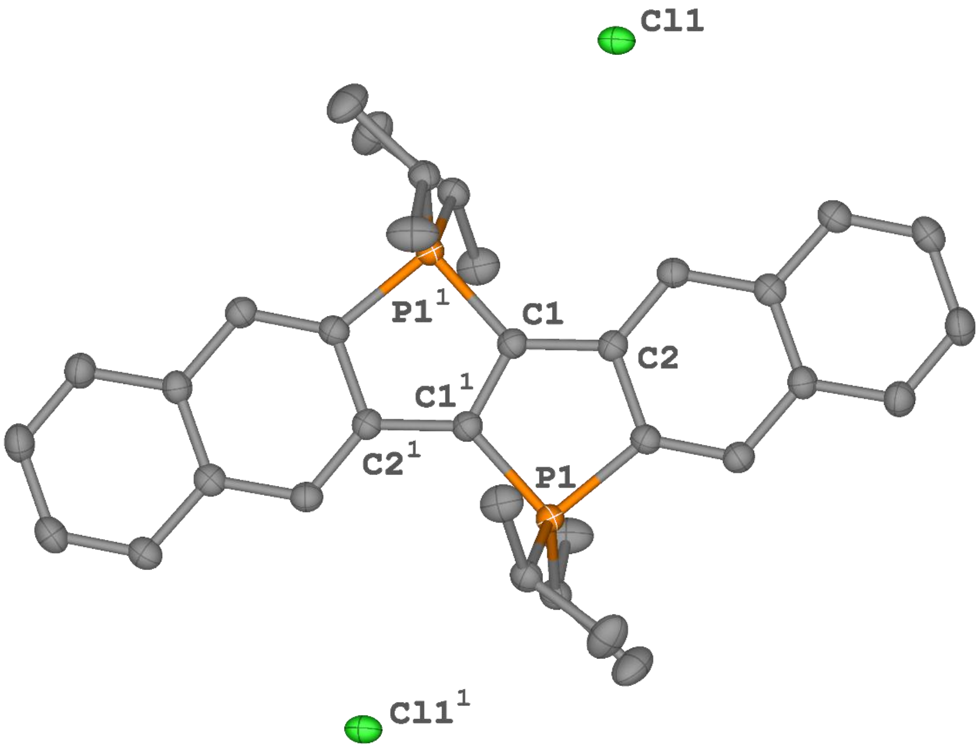

Figure S156. ORTEP plot of the molecular structure of [**4a**]Cl_2 (thermal ellipsoids set to 50% probability; hydrogen atoms omitted for clarity). Selected bond lengths (Å) and angles (°): $P1^{\#1}$–C1 / P1–$C1^{\#1}$ 1.8052(15), C2–C1 / $C2^{\#1}$–$C1^{\#1}$ 1.458(2), C1–$C1^{\#1}$ 1.359(3), C2–C1–$P1^{\#1}$ / $C2^{\#1}$–$C1^{\#1}$–P1 133.50(11), $C1^{\#1}$–C1–$P1^{\#1}$ / C1–$C1^{\#1}$–P1 109.42(14), $C1^{\#1}$–C1–C2 / C1–$C1^{\#1}$–$C2^{\#1}$ 117.06(17).

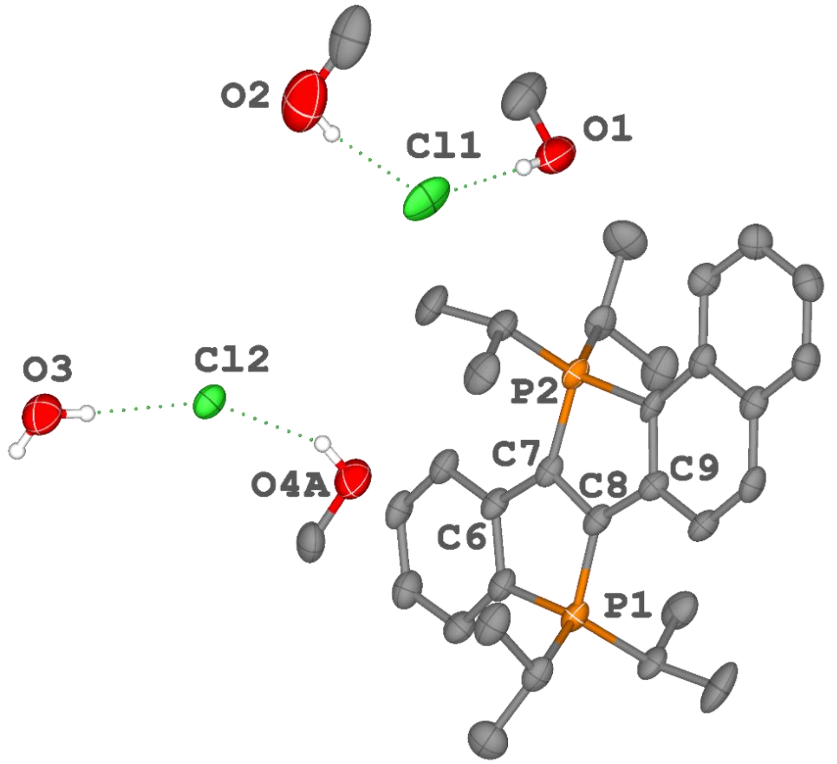

Figure S157. ORTEP plot of the molecular structure of [**4b-Ph**]Cl_2 × 3 MeOH × H_2O (thermal ellipsoids set to 50% probability; disorder omitted for clarity; hydrogen atoms −except protic hydrogens− omitted for clarity). Selected bond lengths (Å) and angles (°): P1–C8 1.803(4), P2–C7 1.801(4), C6–C7 1.466(5), C7–C8 1.365(5), C8–C9 1.456(5), C6–C7–P2 134.3(3), C8–C7–P2 107.7(3), C8–C7–C6 117.8(4), C7–C8–P1 107.9(3), C7–C8–C9 117.2(3), C9–C8–P1 134.9(3).

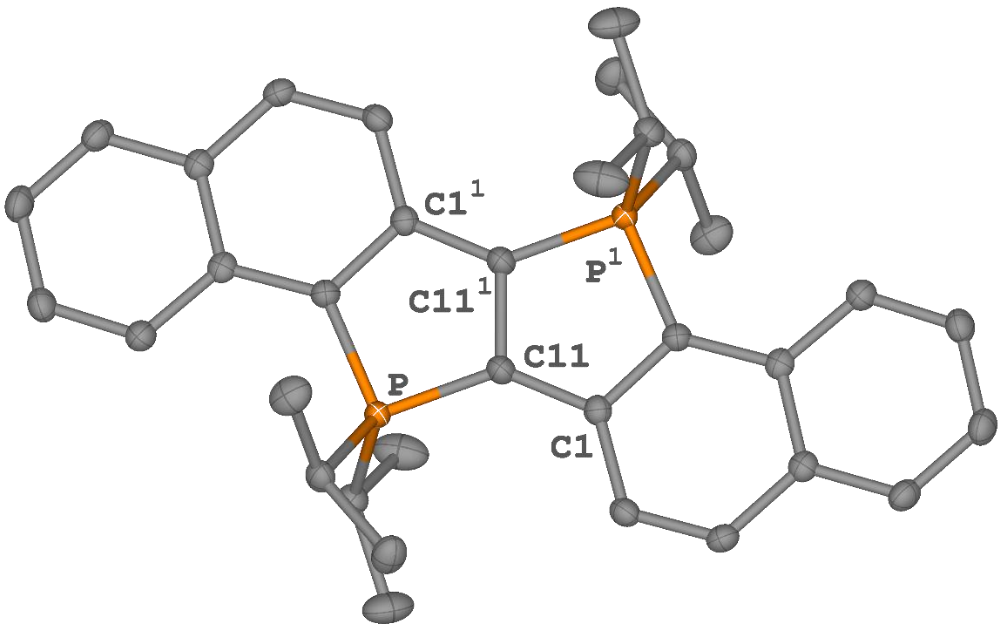

Figure S158. ORTEP plot of the molecular structure of [**4b**]^0 (thermal ellipsoids set to 50% probability; hydrogen atoms omitted for clarity). Selected bond lengths (Å) and angles (°): P–C11 / $P^{\#1}$–$C11^{\#1}$ 1.7428(15), C1–C11 / $C1^{\#1}$–$C11^{\#1}$ 1.408(2), C11–$C11^{\#1}$ 1.472(3), C1–C11–$C11^{\#1}$ / $C1^{\#1}$–$C11^{\#1}$–C11 113.87(16), $C11^{\#1}$–C11–P / C11–$C11^{\#1}$–$P^{\#1}$ 108.55(14).

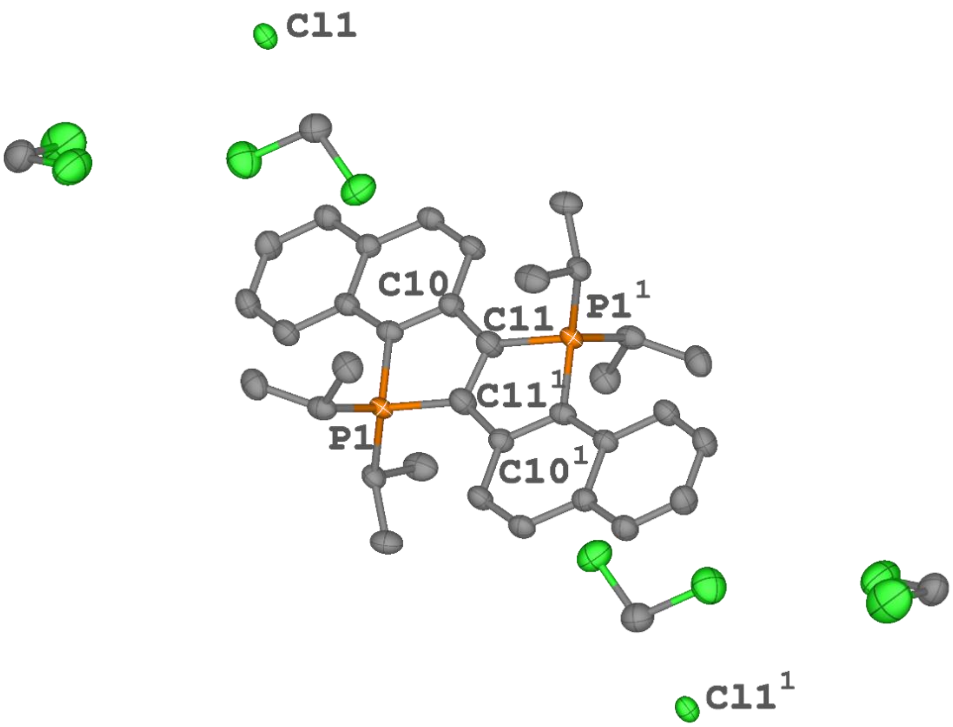

Figure S159. ORTEP plot of the molecular structure of [**4b**]$Cl_2 \times 4\ CH_2Cl_2$ (thermal ellipsoids set to 50% probability; hydrogen atoms omitted for clarity). Selected bond lengths (Å) and angles (°): P1–C11$^{\#1}$ / P1$^{\#1}$–C11 1.817(3), C10–C11 / C10$^{\#1}$–C11$^{\#1}$ 1.465(3), C11–C11$^{\#1}$ 1.355(5), C10–C11–P1$^{\#1}$ / C10$^{\#1}$–C11$^{\#1}$–P1 135.09(19), C11$^{\#1}$–C11–P1$^{\#1}$ / C11–C11$^{\#1}$–P1 107.8(3), C11$^{\#1}$–C11–C10 / C11–C11$^{\#1}$–C10$^{\#1}$ 117.0(3).

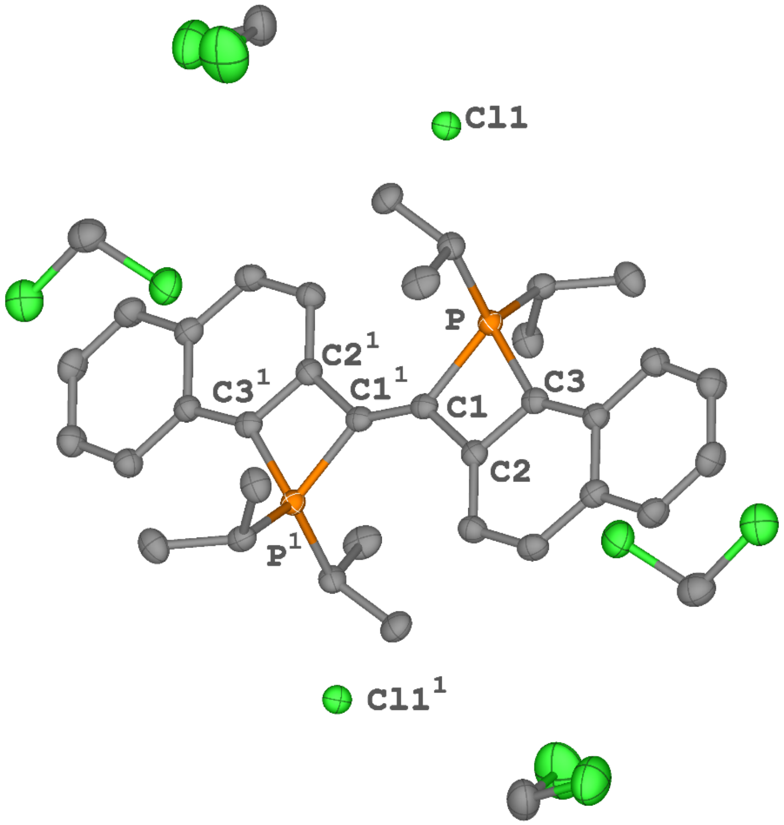

Figure S160. ORTEP plot of the molecular structure of [***iso*-4b**]$Cl_2 \times 4\ CH_2Cl_2$ (thermal ellipsoids set to 50% probability; hydrogen atoms omitted for clarity). Selected bond lengths (Å) and angles (°): P–C1 1.829(2), P–C3 1.792(2), C1–C1$^{\#1}$ 1.336(4), C1–C2 1.478(3), C2–C3 1.393(3), C3–P–C1 76.21(10), C1$^{\#1}$–C1–P / C1–C1$^{\#1}$–P$^{\#1}$ 135.2(2), C1$^{\#1}$–C1–C2 / C1–C1$^{\#1}$–C2$^{\#1}$ 136.1(3), C2–C1–P 88.63(13), C3–C2–C1 102.17(18), C2–C3–P 92.87(15).

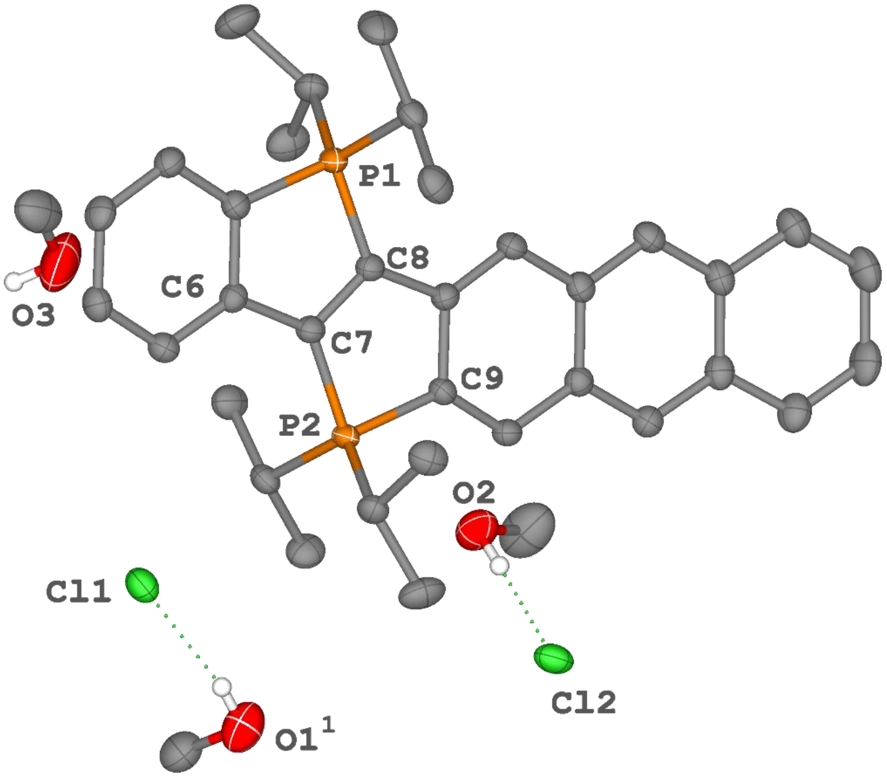

Figure S161. ORTEP plot of the molecular structure of [**4c-Ph**]Cl_2 × 3 MeOH (thermal ellipsoids set to 50% probability; hydrogen atoms −except protic hydrogens− omitted for clarity). Selected bond lengths (Å) and angles (°): P1–C8 1.8015(15), P2–C7 1.8055(14), C6–C7 1.467(2), C7–C8 1.356(2), C8–C9 1.448(2), C6–C7–P2 134.41(11), C8–C7–P2 108.98(11), C8–C7–C6 116.59(13), C7–C8–C9 117.70(13), C7–C8–P1 109.10(11), C9–C8–P1 133.15(11).

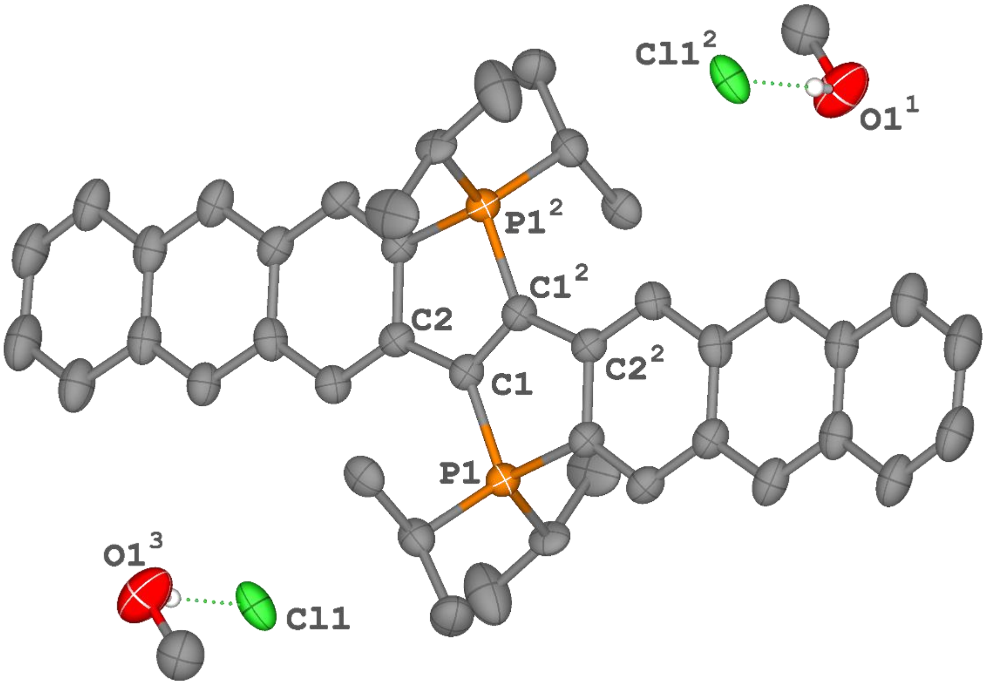

Figure S162. ORTEP plot of the molecular structure of [**4c-Anth**]Cl_2 × 3 MeOH (thermal ellipsoids set to 50% probability; a fully disordered second molecule is omitted for clarity; hydrogen atoms −except protic hydrogens− omitted for clarity). Selected bond lengths (Å) and angles (°) [values for the second molecule are given in square brackets]: P1–C1 1.808(4) [1.797(8)], C1–C1$^{\#1}$ 1.370(7) [1.338(13)], C1–C2 1.452(12) [1.47(2)], C1$^{\#1}$–C1–P1 / C1–C1$^{\#1}$–P1$^{\#1}$ 108.3(4) [110.1(8)], C1$^{\#1}$–C1–C2 / C1–C1$^{\#1}$–C2$^{\#1}$ 118.1(5) [117.5(10)], C2–C1–P1 133.6(4) [132.4(7)].

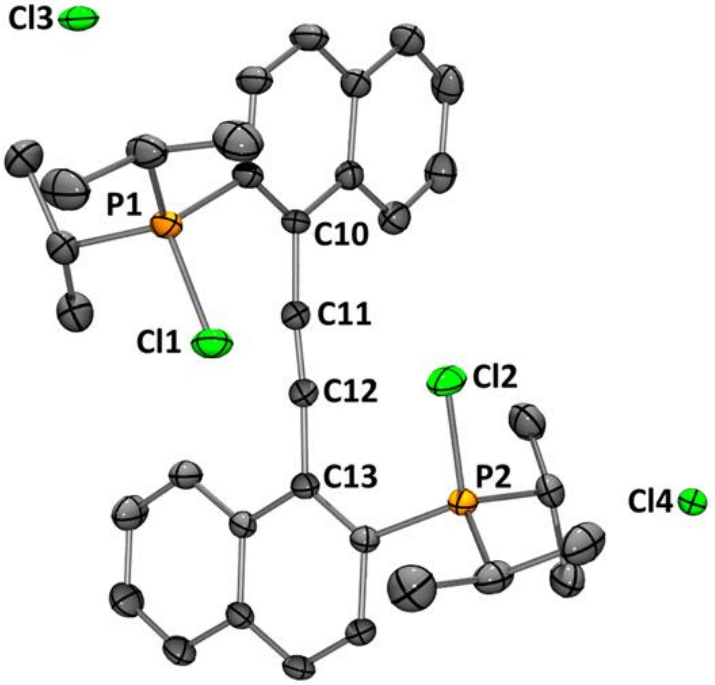

Figure S163. ORTEP plot of the molecular structure of of **[2d-Naph]Cl₂** (thermal ellipsoids set to 50% probability; hydrogen atoms omitted for clarity; only one out of two independent molecules present in the asymmetric unit is show,). Selected bond lengths (Å) and angles (°) [values for the second molecule are given in square brackets]: P1–Cl1 1.9934(10), P2–Cl2 1.9935(11), C10–C11 1.435(4), C11–C12 1.203(4), C12–C13 1.430(4), C10–C11–C12 174.0(3), C11–C12–C13 174.9(3).

Table S38. Crystal data and details of the structure determinations for **3b**, [**4a-Ph**]Cl_2 × 3 MeOH and [**4a**]Cl_2.

compound	**3b**	[**4a-Ph**]Cl_2 × 3 MeOH	[**4a**]Cl_2
Empirical formula	$C_{34}H_{40}P_2$	$C_{33}H_{50}Cl_2O_3P_2$	$C_{34}H_{40}Cl_2P_2$
Formula weight	510.60	627.57	581.50
Temperature [K]	120(1)	120(1)	120(1)
Crystal system	*triclinic*	*monoclinic*	*monoclinic*
Space group (number)	$P1$ (1)	$P2_1/n$ (14)	$P2_1/n$ (14)
a [Å]	10.8371(2)	7.56569(18)	7.24390(10)
b [Å]	11.6905(2)	21.6248(5)	18.9138(2)
c [Å]	12.3737(3)	21.1554(5)	11.37540(10)
α [°]	105.871(2)	90	90
β [°]	90.048(2)	100.276(2)	104.4060(10)
γ [°]	100.228(2)	90	90
Volume [Å^3]	1481.90(5)	3405.63(14)	1509.53(3)
Z	2	4	2
ρ_{calc} [gcm^{-3}]	1.144	1.224	1.279
μ [mm^{-1}]	1.463	2.837	3.090
F(000)	548	1344	616
Radiation	Cu-K_α (λ=1.54184 Å)	Cu-K_α (λ=1.54184 Å)	Cu-K_α (λ=1.54184 Å)
2θ range [°]	7.44 to 142.27 (0.81 Å)	5.89 to 140.87 (0.82 Å)	9.29 to 141.46 (0.82 Å)
Index ranges	−13 ≤ h ≤ 13<br>−14 ≤ k ≤ 14<br>−15 ≤ l ≤ 14	−9 ≤ h ≤ 9<br>−18 ≤ k ≤ 26<br>−25 ≤ l ≤ 25	−8 ≤ h ≤ 8<br>−23 ≤ k ≤ 20<br>−13 ≤ l ≤ 13
Reflections collected	31950	32736	35263
Independent reflections	10602 (R_{int} = 0.0645)	6441 (R_{int} = 0.0806)	2892 (R_{int} = 0.0382)
Completeness to θ	100.0 % (θ = 67.68°)	100.0 % (θ = 67.68°)	99.9 % (θ = 67.68°)
Data / Restraints / Parameters	10602 / 75 / 689	6441 / 22 / 375	2892 / 0 / 176
T_{min}/T_{max}	0.3090 / 0.8460	0.5030 / 0.8820	0.7810 / 0.9990 (gaussian)
Goodness-of-fit on F^2	1.025	1.056	1.092
Final *R* indexes [$I \geq 2\sigma(I)$]	R_1 = 0.0478<br>wR_2 = 0.1225	R_1 = 0.0846<br>wR_2 = 0.2241	R_1 = 0.0338<br>wR_2 = 0.0903
Final *R* indexes [all data]	R_1 = 0.0509<br>wR_2 = 0.1266	R_1 = 0.0978<br>wR_2 = 0.2375	R_1 = 0.0344<br>wR_2 = 0.0908
Largest peak/hole [eÅ^{-3}]	0.68/−0.30	0.94/−0.89	0.19/−0.43
Flack parameter	−0.012(12)	---	---
CCDC number	2431371	2431372	2431373

Table S39. Crystal data and details of the structure determinations for [**4b-Ph**]$Cl_2 \times 3$ MeOH× H_2O, [**4b**]^0 and [**4b**]$Cl_2 \times 4$ CH_2Cl_2.

compound	[**4b-Ph**]$Cl_2 \times 3$ MeOH × H_2O	[**4b**]^0	[**4b**]$Cl_2 \times 4$ CH_2Cl_2
Empirical formula	$C_{33}H_{52}Cl_2O_4P_2$	$C_{34}H_{40}P_2$	$C_{38}H_{48}Cl_{10}P_2$
Formula weight	645.58	510.60	921.20
Temperature [K]	120(1)	120(1)	120(1)
Crystal system	*monoclinic*	*monoclinic*	*monoclinic*
Space group (number)	$P2_1/n$ (14)	$P2_1/n$ (14)	$P2_1/n$ (14)
a [Å]	7.8799(3)	10.67190(10)	14.6337(2)
b [Å]	10.1289(4)	11.97920(10)	10.87690(10)
c [Å]	43.307(2)	11.09950(10)	14.8298(2)
α [°]	90	90	90
β [°]	91.842(5)	104.0550(10)	109.952(2)
γ [°]	90	90	90
Volume [Å^3]	3454.8(3)	1376.49(2)	2218.77(5)
Z	4	2	2
ρ_{calc} [gcm^{-3}]	1.241	1.232	1.379
μ [mm^{-1}]	2.832	1.575	6.635
F(000)	1384	548	952
Radiation	Cu-K_α (λ=1.54184 Å)	Cu-K_α (λ=1.54184 Å)	Cu-K_α (λ=1.54184 Å)
2θ range [°]	8.17 to 134.15 (0.84 Å)	10.32 to 141.78 (0.82 Å)	7.33 to 142.22 (0.81 Å)
Index ranges	−9 ≤ h ≤ 9<br>−12 ≤ k ≤ 12<br>−51 ≤ l ≤ 51	−13 ≤ h ≤ 12<br>−14 ≤ k ≤ 14<br>−13 ≤ l ≤ 13	−17 ≤ h ≤ 17<br>−13 ≤ k ≤ 13<br>−18 ≤ l ≤ 18
Reflections collected	62610	21740	30956
Independent reflections	6168 (R_{int} = 0.1410)	2641 (R_{int} = 0.0745)	4281 (R_{int} = 0.0588)
Completeness to θ	99.9 % (θ = 67.08°)	100.0 % (θ = 67.68°)	100.0 % (θ = 67.68°)
Data / Restraints / Parameters	6168 / 42 / 402	2641 / 0 / 167	4281 / 0 / 230
T_{min}/T_{max}	0.7380 / 1.0000	0.5980 / 1.0000	0.8130 / 1.0000
Goodness-of-fit on F^2	1.044	1.054	1.026
Final *R* indexes [$I \geq 2\sigma(I)$]	R_1 = 0.0668<br>wR_2 = 0.1501	R_1 = 0.0394<br>wR_2 = 0.1068	R_1 = 0.0435<br>wR_2 = 0.1121
Final *R* indexes [all data]	R_1 = 0.0986<br>wR_2 = 0.1708	R_1 = 0.0415<br>wR_2 = 0.1090	R_1 = 0.0504<br>wR_2 = 0.1181
Largest peak/hole [eÅ^{-3}]	0.41/−0.39	0.41/−0.48	0.47/−0.48
CCDC number	2431374	2431376	2431375

Table S40. Crystal data and details of the structure determinations for [***iso*-4b**]Cl$_2$ × 4 CH$_2$Cl$_2$, [**4c-Ph**]Cl$_2$ × 3 MeOH and [**4c-Anth**]Cl$_2$ × 2 MeOH.

compound	[***iso*-4b**]Cl$_2$ × 4 CH$_2$Cl$_2$	[**4c-Ph**]Cl$_2$ × 3 MeOH	[**4c-Anth**]Cl$_2$ × 2 MeOH
Empirical formula	$C_{38}H_{48}Cl_{10}P_2$	$C_{37}H_{52}Cl_2O_3P_2$	$C_{44}H_{52}Cl_2O_2P_2$
Formula weight	921.20	677.62	745.69
Temperature [K]	120(1)	120(1)	120(1)
Crystal system	*monoclinic*	*monoclinic*	*monoclinic*
Space group (number)	$P2_1/n$ (14)	$P2_1/c$ (14)	$P2_1/n$ (14)
a [Å]	15.0324(2)	19.8117(2)	11.48118(8)
b [Å]	10.6843(2)	11.70200(10)	13.37430(9)
c [Å]	15.1691(3)	17.50060(10)	13.10304(12)
α [°]	90	90	90
β [°]	112.535(2)	115.6810(10)	102.6366(8)
γ [°]	90	90	90
Volume [Å^3]	2250.30(7)	3656.50(6)	1963.27(3)
Z	2	4	2
ρ_{calc} [gcm^{-3}]	1.360	1.231	1.261
μ [mm^{-1}]	6.542	2.682	2.530
F(000)	952	1448	792
Radiation	Cu-K_α (λ=1.54184 Å)	Cu-K_α (λ=1.54184 Å)	Cu-K_α (λ=1.54184 Å)
2θ range [°]	7.04 to 141.84 (0.82 Å)	9.04 to 141.86 (0.82 Å)	9.57 to 142.29 (0.81 Å)
Index ranges	−18 ≤ h ≤ 18<br>−13 ≤ k ≤ 13<br>−18 ≤ l ≤ 18	−24 ≤ h ≤ 24<br>−13 ≤ k ≤ 14<br>−21 ≤ l ≤ 21	−14 ≤ h ≤ 13<br>−16 ≤ k ≤ 16<br>−15 ≤ l ≤ 15
Reflections collected	39081	97563	75260
Independent reflections	4305 (R_{int} = 0.0853)	7023 (R_{int} = 0.0553)	3759 (R_{int} = 0.0532)
Completeness to θ	99.9 % (θ = 67.68°)	100.0 % (θ = 67.68°)	99.9 % (θ = 67.68°)
Data / Restraints / Parameters	4305 / 0 / 230	7023 / 0 / 411	3759 / 611 / 466
T_{min}/T_{max}	0.4840 / 0.8410	0.7320 / 1.0000	0.4440 / 1.0000
Goodness-of-fit on F^2	1.024	1.058	1.149
Final *R* indexes [$I≥2σ(I)$]	R_1 = 0.0444<br>wR_2 = 0.1168	R_1 = 0.0348<br>wR_2 = 0.0962	R_1 = 0.0516<br>wR_2 = 0.1355
Final *R* indexes [all data]	R_1 = 0.0500<br>wR_2 = 0.1232	R_1 = 0.0367<br>wR_2 = 0.0977	R_1 = 0.0534<br>wR_2 = 0.1367
Largest peak/hole [eÅ^{-3}]	0.68/−0.68	0.39/−0.30	0.43/−0.24
CCDC number	2431377	2431378	2431379

Table S41. Crystal data and details of the structure determinations for [**2d-Naph**]$Cl_2 \times 6\ CH_2Cl_2$.

compound	[**2d-Naph**]$Cl_2 \times 6\ CH_2Cl_2$
Empirical formula	$C_{40}H_{52}Cl_{16}P_2$
Formula weight	1161.95
Temperature [K]	120(1)
Crystal system	*triclinic*
Space group (number)	P-1
a [Å]	10.3156(2)
b [Å]	11.7343(2)
c [Å]	23.0477(5)
α [°]	85.394(2)
β [°]	79.220(2)
γ [°]	89.6000(10)
Volume [Å^3]	2731.66(9)
Z	2
ρ_{calc} [gcm^{-3}]	1.413
μ [mm^{-1}]	8.144
F(000)	1188.0
Radiation	Cu K_α (λ = 1.54184)
2θ range [°]	7.558 to 142.072
Index ranges	-12 ≤ h ≤ 12<br>-14 ≤ k ≤ 14<br>-28 ≤ l ≤ 26
Reflections collected	65423
Independent reflections	10392 (R_{int} = 0.0908)
Completeness to θ	98.2 % (θ = 71.04°)
Data / Restraints / Parameters	10392/3/536
T_{min}/T_{max}	0.7300 / 1.0000
Goodness-of-fit on F^2	1.041
Final *R* indexes [$I \geq 2\sigma(I)$]	R_1 = 0.0563<br>wR_2 = 0.1502
Final *R* indexes [all data]	R_1 = 0.0644<br>wR_2 = 0.1587
Largest peak/hole [eÅ^{-3}]	1.25/-0.89
CCDC number	2470828

8) Additional Compounds and Spectra

8.1) Synthesis of 2d-Naph and Attempted Oxidation to $[4d]^{2+}$

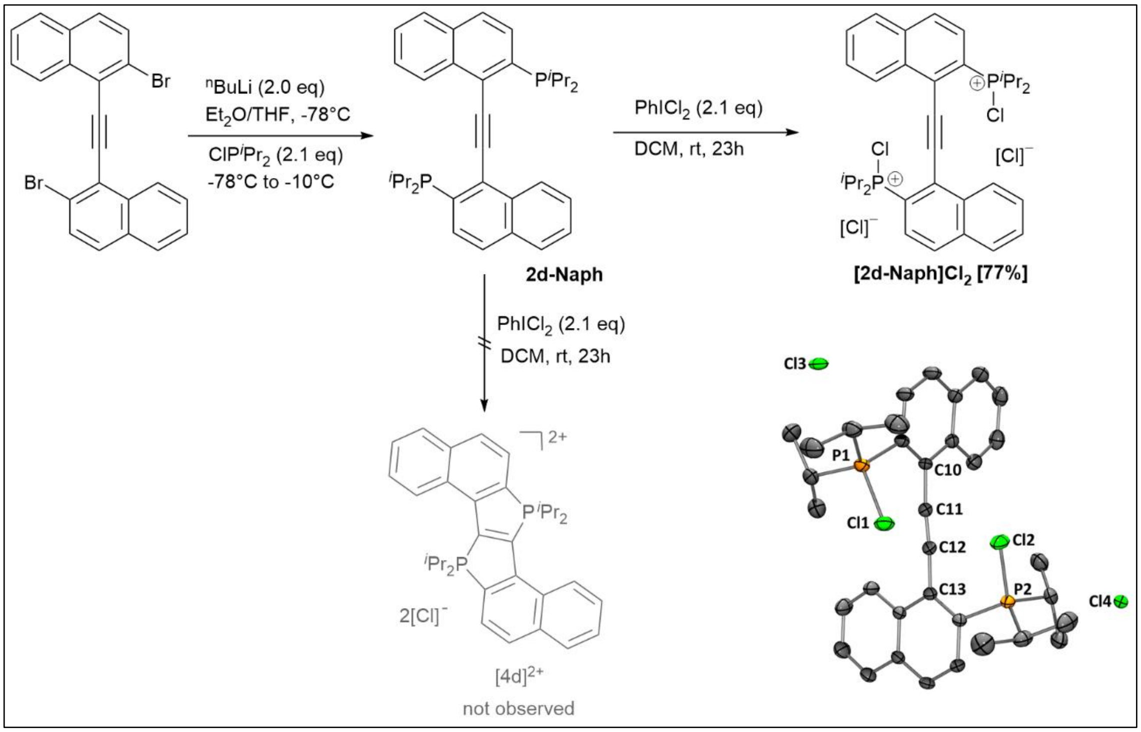

Scheme S6. Synthesis and oxidation of **2d-Naph**.

The oxidation of **2d-Naph** with $PhICl_2$ does not yield the corresponding phosphorus-bridged ladder stilbene. Alternative chlorinating agents such as C_2Cl_6 and [Fc]Cl also fail to induce the cyclization reaction. Instead, chlorination occurs selectively at the phosphorus centers, and no cyclization takes place following the initial monochlorination step, as proposed in mechanism B (Figure 1 in the main manuscript). In the absence of an oxidizing agent, **2d-Naph** was found to be thermally highly stable, for example, refluxing in toluene did not result in either cyclization or decomposition. Upon chlorination, the resulting dichlorinated product **[2d-Naph]Cl₂** was successfully isolated and unequivocally characterized by NMR spectroscopy, mass spectrometry, and single-crystal X-ray diffraction. **[2d-Naph]Cl₂** was synthesized as follows: **2d-Naph** (300 mg, 588 μmol) was dissolved in dry DCM (5 ml) inside the glovebox. $PhICl_2$ (170 mg, 617 μmol) was added and the reaction mixture was stirred at rt for 23 h. Further $PhICl_2$ (170 mg, 617 μmol) was added and the reaction mixture was stirred at rt for additional 23 h until the ^{31}P NMR spectrum showed full conversion. The solvent was removed under reduced pressure and the precipitate was recrystallized from dry DCM. Compound **[2d-Naph]Cl₂** was obtained as a yellow solid (296 mg, 77%). **^{1}H NMR** (600 MHz, CD_2Cl_2): δ (in ppm) = 9.21–9.18 (m, 2H), 8.52 (d, $J_{H\text{–}H}$ = 7.5 Hz, 2H), 8.47–8.45 (m, 2H), 8.15 (d, $J_{H\text{–}H}$ = 7.9 Hz, 2H), 7.88–7.86 (m, 2H), 7.81–7.78 (m, 2H), 4.92–4.88 (m, 4H), 1.51–1.46 (m, 24H). **^{13}C{^{1}H} NMR** (151 MHz, CD_2Cl_2): δ (in ppm) = 136.0 (d, $J_{C\text{–}P}$ = 2.7 Hz, C_q, 2C), 134.6 (d, $J_{C\text{–}P}$ = 10.4 Hz, C_q, 2C), 132.7 (d, $J_{C\text{–}P}$ = 14.9 Hz, CH, 2C), 131.4 (CH, 2C), 130.1 (CH, 2C), 129.8 (CH, 2C), 128.3 (d, $J_{C\text{–}P}$ = 15.9 Hz, CH, 2C), 126.6 (CH, 2C), 125.9 (d, $J_{C\text{–}P}$ = 2.1 Hz, C_q, 2C), 116.8 (d, $J_{C\text{–}P}$ = 72.3 Hz, C_q, 2C), 98.8 (d, $J_{C\text{–}P}$ = 7.3 Hz, C_q, 2C), 31.0 (d, $J_{C\text{–}P}$ = 34.2 Hz, CH, 4C), 17.0 (d, $J_{C\text{–}P}$ = 3.7 Hz, CH_3, 8C). **^{31}P{^{1}H} NMR** (162 MHz, CD_2Cl_2): δ (in ppm) = 107.6 (s). **LIFDI** (pos): *m/z* calcd for $C_{34}H_{39}Cl_2P_2^+$ $[M\text{-}2Cl\text{-}H]^+$ 579.1899, found 579.2125.

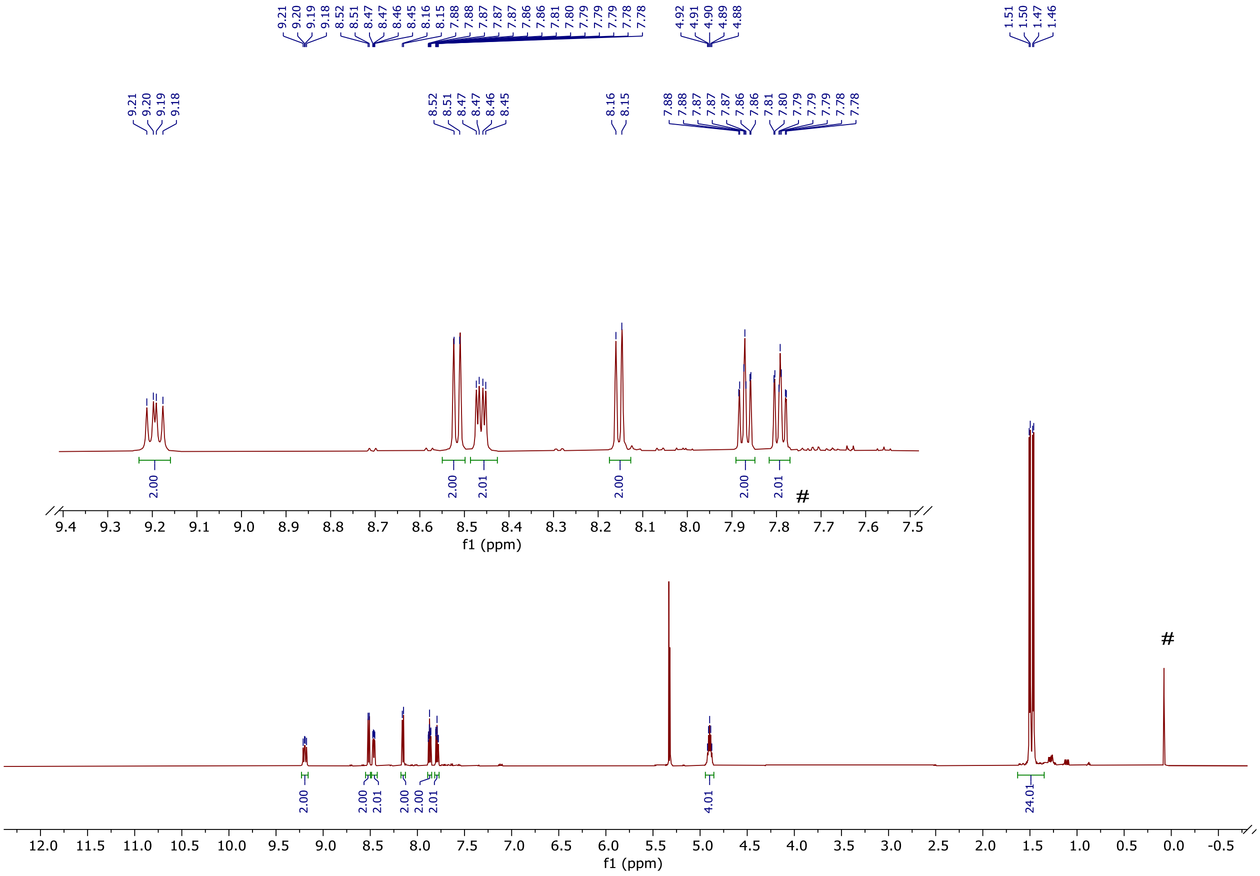

Figure S164. **^{1}H NMR** (600 MHz, CD_2Cl_2, 298K) spectrum of **$[2d\text{-}Naph]^{2+}$**(traces of silicon grease (#) are marked).

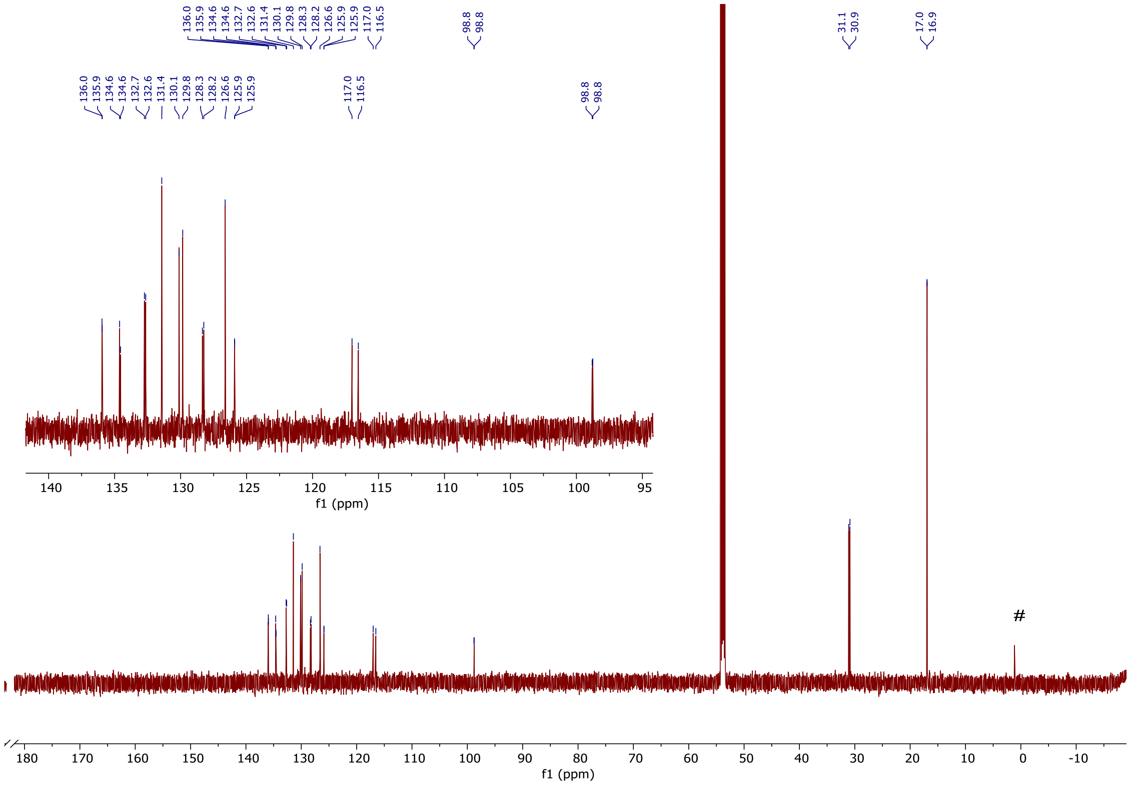

Figure S165. **^{13}C {^{1}H} NMR** (151 MHz, CD_2Cl_2, 298K) spectrum of **$[2d\text{-}Naph]^{2+}$**(traces of silicon grease (#) are marked).

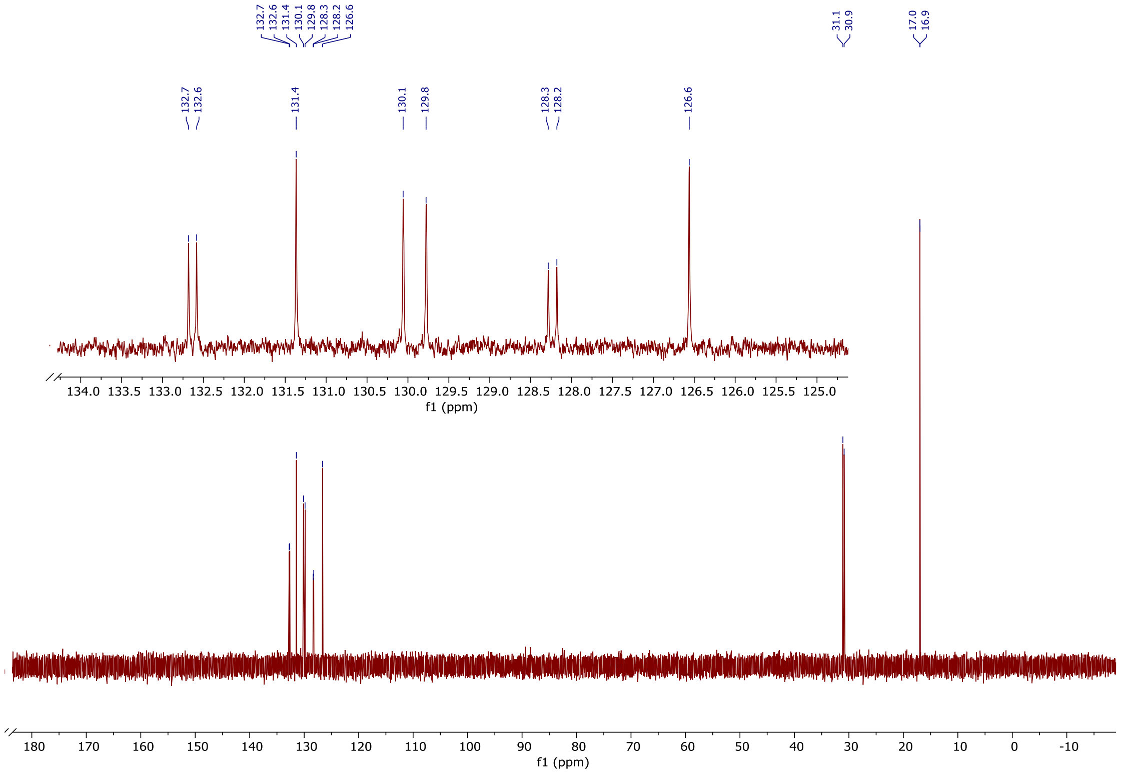

Figure S166.**^{13}C {^{1}H} DEPT-135 NMR** (151 MHz, CD_2Cl_2, 298K) spectrum of **[2d-Naph]$^{2+}$**.

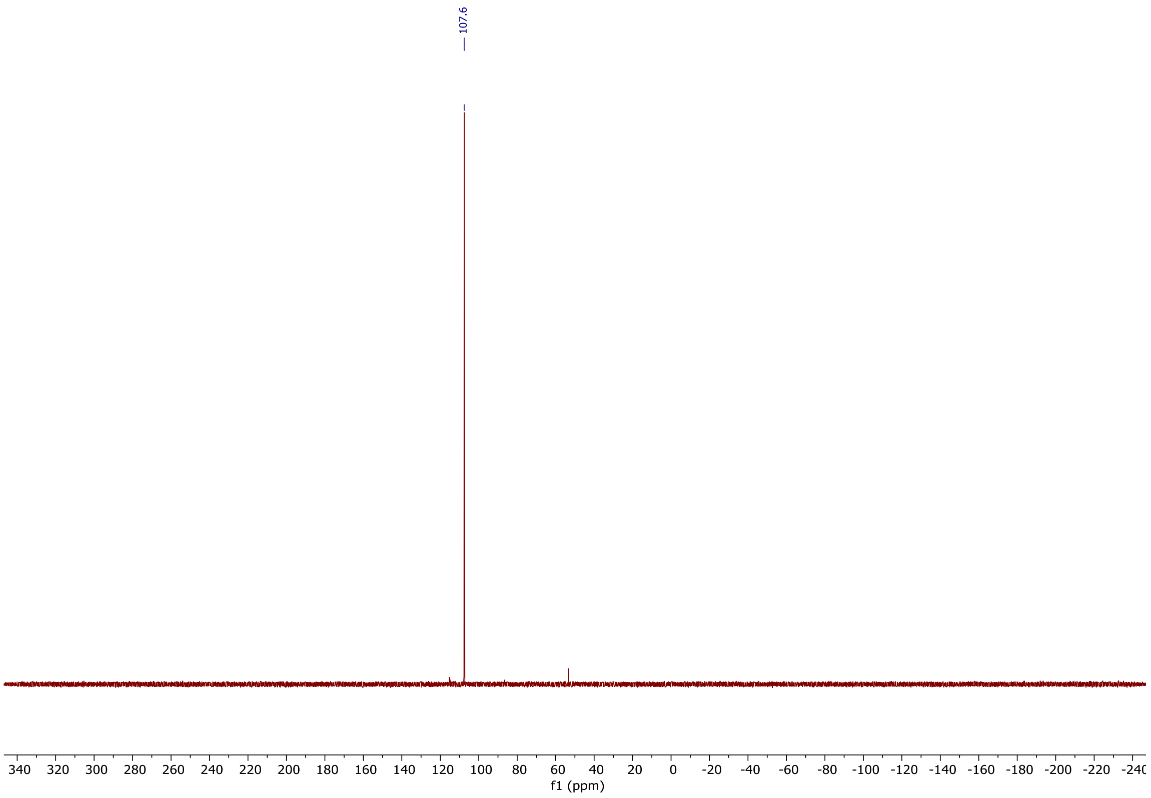

Figure S167. **^{31}P {^{1}H} NMR** (162 MHz, CD_2Cl_2, 298K) spectrum of **[2d-Naph]$^{2+}$**.

9) References

[1] A. E. Brown, B. E. Eichler, *Tetrahedron Lett.* 2011, **52**, 1960-1963**.**

[2] A. Dieudonné-Vatran, M. Azoulay, J.-C. Florent, *Org. Biomol. Chem.* 2012, **10**, 2683-2691.

[3] J. Bucher, T. Wurm, S. Taschinski, E. Sachs, D. Ascough, M. Rudolph, F. Rominger, A. S. K. Hashmi, *Adv. Synth. Catal.* 2017, **359**, 225-233.

[4] C. S. LeHoullier, G. W. Gribble, *J. Org. Chem.* 1983, **48**, 2364-2366.

[5] F. Cottet, E. Castagnetti, M. Schlosser, *Synthesis* 2005, **5**, 798-803.

[6] J. Hellberg, F. Allared, M. Pelcman, *Synth. Commun.* 2003, **33**, 2751-2756**.**

[7] G. E. Martinez, J. W. Nugent, A. R. Fout, *Organometallics* 2018, **37**, 2941-2944.

[8] D. Schaarschmidt, M. Grumbt, A. Hildebrandt, H. Lang, *Eur. J. Org. Chem.* 2014, 6676-6685.

[9] C. G. Newton, E. Braconi, J. Kuziola, M. D. Wodrich, N. Cramer, *Angew. Chem. Int. Ed.* 2018, **57**, 11040-11044.

[10] R. A. Haggam, *Tetrahedron* 2013, **69**, 6488-6494.

[11] W. C. W. Leu, C. S. Hartley, *Org. Lett.* 2013, **15**, 3762-3765

[12] T. Ohashi, M. Watanabe, Tosoh Corporation, patent JP2009155308A, 2009

[13] C. P. Gerlach, F. B. Mccormick, 3M Innovative Properties Company, patent US20060105199A1, 2006.

[14] C. Zarate, M. Nakajima, R. Martin, *J. Am. Chem. Soc.* 2017, **139**, 1191−1197.

[15] W. Zhang, J. M. Ready, *Angew. Chem. Int. Ed.* 2014, **53**, 8980-8984

[16] R. Dorel, P. l. R. McGonigal, A. M. Echavarren, *Angew. Chem. Int. Ed.* 2016, **55**, 11120-11123

[17] M. Montalti, A. Credi, L. Prodi, M. T. Gandolfi, *Handbook of Photochemistry*, 2006.

[18] K. Suzuki, A. Kobayashi, S. Kaneko, K. Takehira, T. Yoshihara, H. Ishida, Y. Shiina, S. Oishi, S-Tobita, *Phys. Chem. Chem. Phys.* 2009, **11**, 9850-9860.

[19] C. Reichardt, *Chem. Rev.* 1994, **94**, 2319-2358.

[20] W. R. Kitzmann, M.-S. Bertrams, P. Boden, A. C. Fischer, R. Klauer, J. Sutter, R. Naumann, C. Förster, G. Niedner-Schatteburg, N. H. Bings, J. Hunger, C.Kerzig, K. Heinze, *J. Am. Chem. Soc.,* 2023, **145**, 16597-16609.

[21] a) R. Isimatsu, S. Matsunami, K. Shinzu, C. Adachi, K.Nakano, T. Imato, *J. Phys. Chem. A*, 2013, **117**, 5607-5612, b) S. Yan, T. Lü, Z.Chen,Y.Wang, J. Xiao, *J. Luninesc.* 2025, **280**, 121110.

[22] M.-S. Bertrams, K. Hermainski, J.-M. Mörsdorf, J. Ballmann, C. Kerzig, *Chem. Sci*., 2023, **14**, 8583-8591.

[23] P. Federmann, H. K. Wagner, P. W. Antoni, J.-M. Mörsdorf, J. L. Pérez Lustres, H. Wadepohl, M. Motzkus, J. Ballmann, *Org. Lett.*, 2019, **21**, 2033-2038.

[24] A. Fukazawa, M. Hara, T. Okamoto, E.-C. Son, C. Xu, K. Tamao and S. Yamaguchi, *Org. Lett.*, 2008, **10**, 913-916.

[25] K. Górski, Ł. W. Ciszewski, A. Wrzosek, A. Szewczyk, A. L. Sobolewski and D. T. Gryko, *Org. Chem. Front.*, 2025, Advance Article, DOI: 10.1039/D5QO00708A.

[26] F. Neese, F. Wennmohs, U. Becker, C. Riplinger, *J. Chem. Phys.* 2020, **152**, article number 224108.

[27] S. Grimme, A. Hansen, S. Ehlert, J.-M. Mewes, *J. Chem. Phys.* 2021, **154** article number 064103.

[28] H. Kruse, S. Grimme, *J. Chem. Phys.* 2012, **136**, article number 154101.

[29] a) E. Caldeweyher, C. Bannwarth, S. Grimme, *J. Chem. Phys.* 2017, **147**, article number 034112; b) E. Caldeweyher, S. Ehlert, A. Hansen, H. Neugebauer, S. Spicher, G. Bannwarth, S. Grimme, *J. Chem. Phys.* 2019;**150**, article number 154122.

[30] M. J. Frisch, G. W. Trucks, H. B. Schlegel, G. E. Scuseria, M. A. Robb, J. R. Cheeseman, G. Scalmani, V. Barone, G. A. Petersson, H. Nakatsuji, X. Li, M. Caricato, A. V. Marenich, J. Bloino, B. G. Janesko, R. Gomperts, B. Mennucci, H. P. Hratchian, J. V. Ortiz, A. F. Izmaylov, J. L. Sonnenberg, D. Williams-Young, F. Ding, F. Lipparini, F. Egidi, J. Goings, B. Peng, A. Petrone, T. Henderson, D. Ranasinghe, V. G. Zakrzewski, J. Gao, N. Rega, G. Zheng, W. Liang, M. Hada, M. Ehara, K. Toyota, R. Fukuda, J. Hasegawa,

M. Ishida, T. Nakajima, Y. Honda, O. Kitao, H. Nakai, T. Vreven, K. Throssell, J. A. Montgomery, Jr., J. E. Peralta, F. Ogliaro, M. J. Bearpark, J. J. Heyd, E. N. Brothers, K. N. Kudin, V. N. Staroverov, T. A. Keith, R. Kobayashi, J. Normand, K. Raghavachari, A. P. Rendell, J. C. Burant, S. S. Iyengar, J. Tomasi, M. Cossi, J. M. Millam, M. Klene, C. Adamo, R. Cammi, J. W. Ochterski, R. L. Martin, K. Morokuma, O. Farkas, J. B. Foresman, D. J. Fox, Gaussian 16 RevC.01, G*aussian, Inc.*, Wallingford CT, 2019.

[31] a) A. D. Becke, *J. Chem. Phys.* 1993, **98**, 5648-5652; b) C. Lee, W. Yang, R. G. Parr, *Phys. Rev. B* 1988, **37**, 785-789; c) P. J. Stephens, F. J. Devlin, C. F. Chabalowski, M. J. Frisch, *J. Phys. Chem.* 1994, **98**, 11623-11627.

[32] F. Weigend, R. Ahlrichs, *Phys. Chem. Chem. Phys.* 2005, **7**, 3297-3305.

[33] S. Grimme, J. Antony, S. Ehrlich, H. Krieg, *J. Chem. Phys*. 2010, **132**, article number 154104.

[34] a) J. Tomasi, B. Mennucci, R. Cammi, R., *Chem. Rev.* 2005, **105**, 2999-3094; b) A. V. Marenich, C. J. Cramer, D. G. Truhlar, *J. Phys. Chem. B* 2009, **113**, 6378-6396.

[35] a) D. Geuenich, K. Hess, F. Koehler, R. Herges, *Chem. Rev.* 2005, **105**, 3758-3772; b) R. Herges, D. Geuenich, *J. Phys. Chem. A* 2001, **105**, 3214-3220.

[36] POV-Ray (ver. 3.7), Persistence of Vision Pty. Ltd., Williamstown, Victoria, Australia, (www.povray.org).

[37] Z. Wang, *Chemistry*, 2024, **6**, 1692-1703.

[38] R. Dennington, T. A. Keith, J. M. Millam, GaussView (ver. 6.1), *Semichem Inc.*, Shawnee Mission, 2016.

[39] Chemcraft - graphical software for visualization of quantum chemistry computations (www.chemcraftprog.com).

[40] K. Kabsch, in *International Tables for Crystallography*, Eds. M. G. Rossmann, E. Arnold, *Vol. F*, Ch. 11.3, Kluwer Academic Publishers, Dordrecht, The Netherlands, 2001.

[41] SAINT, Bruker AXS GmbH, Karlsruhe, Germany 1997-2013 and SAINT V8.40A, Bruker AXS Inc., Madison, Wisconsin, USA, 2018.

[42] *CrysAlisPro*, Rigaku Oxford Diffraction, Rigaku Polska Sp.z o.o., Wrocław, Poland 2015-2024.

[43] R. H. Blessing, *Acta Cryst.* 1995, **A51**, 33-38.

[44] G. M. Sheldrick, *SADABS*, Bruker AXS GmbH, Karlsruhe, Germany 2004-2014.

[45] *SCALE3 ABSPACK, CrysAlisPro*, Rigaku Oxford Diffraction, Rigaku Polska Sp.z o.o., Wrocław, Poland 2015-2024.

[46] W. R. Busing, H. A. Levy, *Acta Cryst.* 1957, **10**, 180-182.

[47] O. V. Dolomanov, L. J. Bourhis, R. J. Gildea, J. A. K. Howard, H. Puschmann, *J. Appl. Cryst.* 2009,**42**, 339-341.

[48] a) G. M. Sheldrick, SHELXT, University of Göttingen and Bruker AXS GmbH, Karlsruhe, Germany, 2012-2018; b) G. M. Sheldrick, *Acta Cryst.* 2015, **A71**, 3-8.

[49] a) G. M. Sheldrick, *SHELXL-20xx*, University of Göttingen and Bruker AXS GmbH, Karlsruhe, Germany 2012-2018; b) G. M. Sheldrick, *Acta Cryst.* 2008, **A64**, 112-122; c) G. M. Sheldrick, *Acta Cryst.* 2015, **C71**, 3-8.

[50] a) J .S. Rollett, in *Crystallographic Computing*, Eds. F. R. Ahmed, S. R. Hall, C. P. Huber, Munksgaard, Copenhagen, Denmark, p. 167, 1970; b) D. Watkin, in *Crystallographic Computing 4*, Eds. N. W. Isaaks, M. R. Taylor, Ch. 8, IUCr and Oxford University Press, Oxford, UK, 1988; c) P. Müller, R. Herbst-Irmer, A. L. Spek, T. R. Schneider, M. R. Sawaya, in *Crystal Structure Refinement*, Ed. P. Müller, Ch. 5, Oxford University Press, Oxford, UK, 2006; d) D. Watkin, *J. Appl. Cryst.* 2008, **41**, 491-522; e) A. Thorn, B. Dittrich, G. M. Sheldrick, *Acta Cryst.* 2012, **A68**, 448-451.

[51] A. M. Deetz, L. Troian-Gautier, S. A. M. Wehlin, E. J. Piechota, G. J. Meyer, *J. Phys. Chem. A.,* 2021, **125**, 9355-9367.

[52] R. Bevernaegie, S. A. Wehlin, E. J. Piechota, M. Abraham, C. Philouze, G. J: Meyer, B. Elijas, L. Troian-Gautier, *J. Am. Chem. Soc*., 2020, **142**, 2732-2737.
